# Single point activation of pyridines enables reductive hydroxymethylation†

**DOI:** 10.1039/d0sc05656a

**Published:** 2020-11-16

**Authors:** Bruno Marinic, Hamish B. Hepburn, Alexandru Grozavu, Mark Dow, Timothy J. Donohoe

**Affiliations:** Chemistry Research Laboratory, University of Oxford Mansfield Road Oxford OX1 3TA UK timothy.donohoe@chem.ox.ac.uk; AstraZeneca Silk Road Macclesfield SK10 2NA UK

## Abstract

The single point activation of pyridines, using an electron-deficient benzyl group, facilitates the ruthenium-catalysed dearomative functionalisation of a range of electronically diverse pyridine derivatives. This transformation delivers hydroxymethylated piperidines in good yields, allowing rapid access to medicinally relevant small heterocycles. A noteworthy feature of this work is that paraformaldehyde acts as both a hydride donor and an electrophile in the reaction, enabling the use of cheap and readily available feedstock chemicals. Removal of the activating group can be achieved readily, furnishing the free NH compound in only 2 steps. The synthetic utility of the method was illustrated with a synthesis of (±)-Paroxetine.

## Abstract 2

Pyridines can be activated at a single point with a new benzyl group, followed by dearomative functionalisation at C3 using formaldehyde.

## Graphical abstract

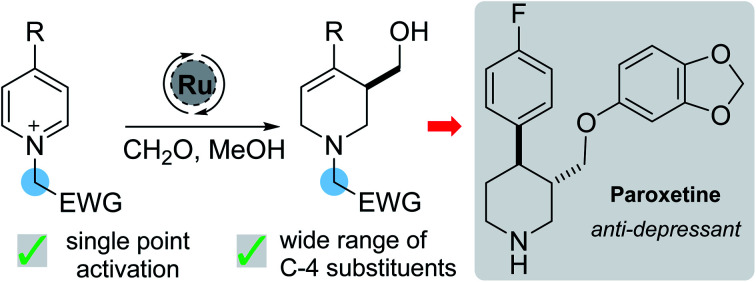

## Introduction

**Scheme 1:**
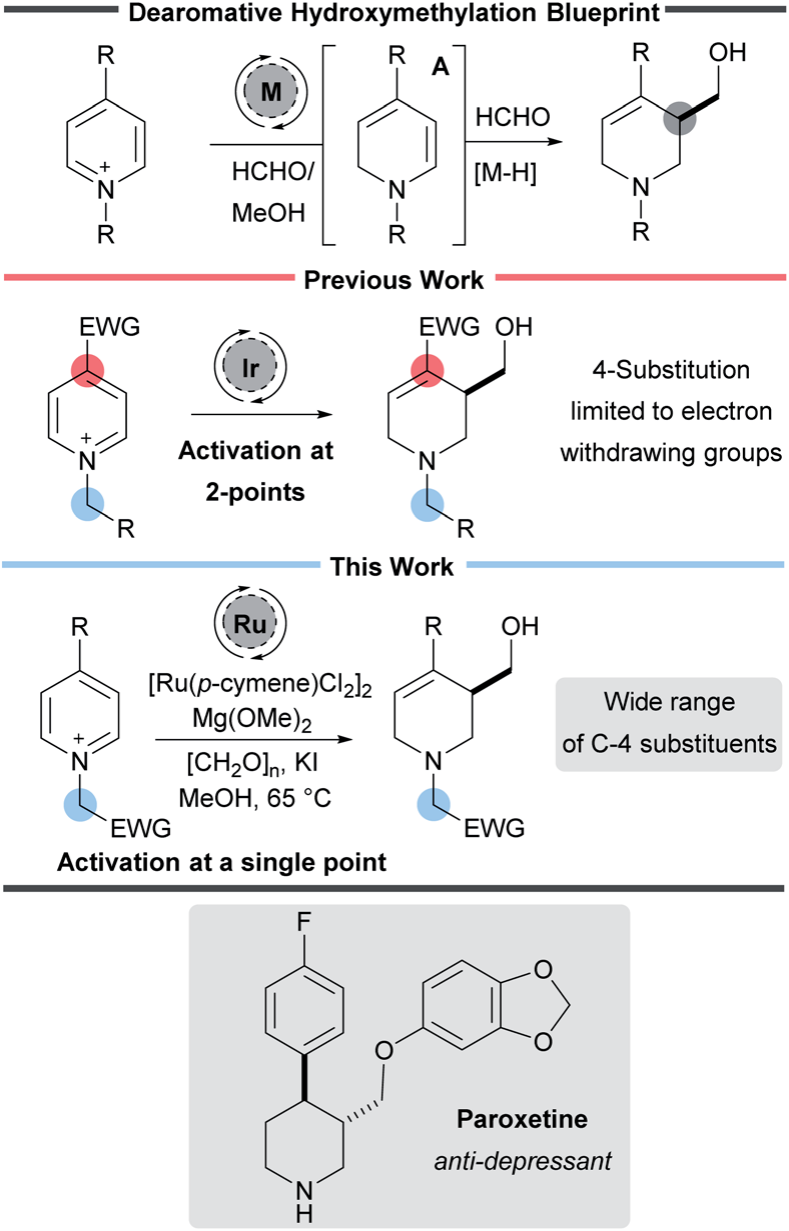
Reaction blueprint and activation strategy.

Nitrogen-containing heterocycles play an essential role in organic chemistry. They are found in a wide range of pharmaceuticals, drug candidates, agrochemicals, natural products, functional materials, and catalysts.^1^ Despite considerable synthetic advances, the pharmaceutical industry still requires processes that allow the facile preparation of aliphatic nitrogen-containing heterocycles along with methods that install small but essential functional groups such as –CH_2_OH and methods which enable the rapid and robust preparation of such useful molecules are of great interest to the synthetic chemistry community (Scheme 1).^2^

Dearomatisation reactions have garnered considerable interest because they represent an expedient way to prepare complex three-dimensional molecular scaffolds from flat and readily available aromatic compounds.^3^ Given the particular relevance of piperidines, and the commercial availability of pyridines, the dearomative pathway represents an attractive approach to preparing saturated azacycles.^4^ In particular dearomative methods that form both C–H and C–C bonds represent an important class of reaction that are particularly versatile and efficient. However, despite their importance,^5–12^ these types of reactions continue to be underrepresented in the literature.

Recently our own laboratory has reported a new iridium-catalysed interrupted transfer hydrogenation process that leads to the reductive hydroxymethylation of pyridines.^13–16^ In addition to forming two new C–H bonds, this process also forms a new C–C bond in a single synthetic step. Mechanistic work has shown that the metal catalyst oxidises formaldehyde in methanol to methyl formate, forming a metal hydride in the process.^13^ Addition of the metal hydride to the C-2 position of the pyridine then forms a key enamine intermediate A. Reaction of the enamine with excess formaldehyde, and subsequent iminium ion reduction then completes the transformation.

As with many dearomative reactions, it is necessary to activate the pyridine substrates to facilitate the initial aromaticity-breaking step. We found that activation could be achieved through a two-point strategy: (i) quaternisation of the pyridine nitrogen to form a highly electron deficient pyridinium salt and (ii) the presence of an electron-withdrawing substituent in the 4-position of the pyridine to further enhance the electron deficiency. The 4-substituent also ensures the initial reduction happens at C-2 exclusively, and prevents over reduction *in situ*.

**Scheme 2:**
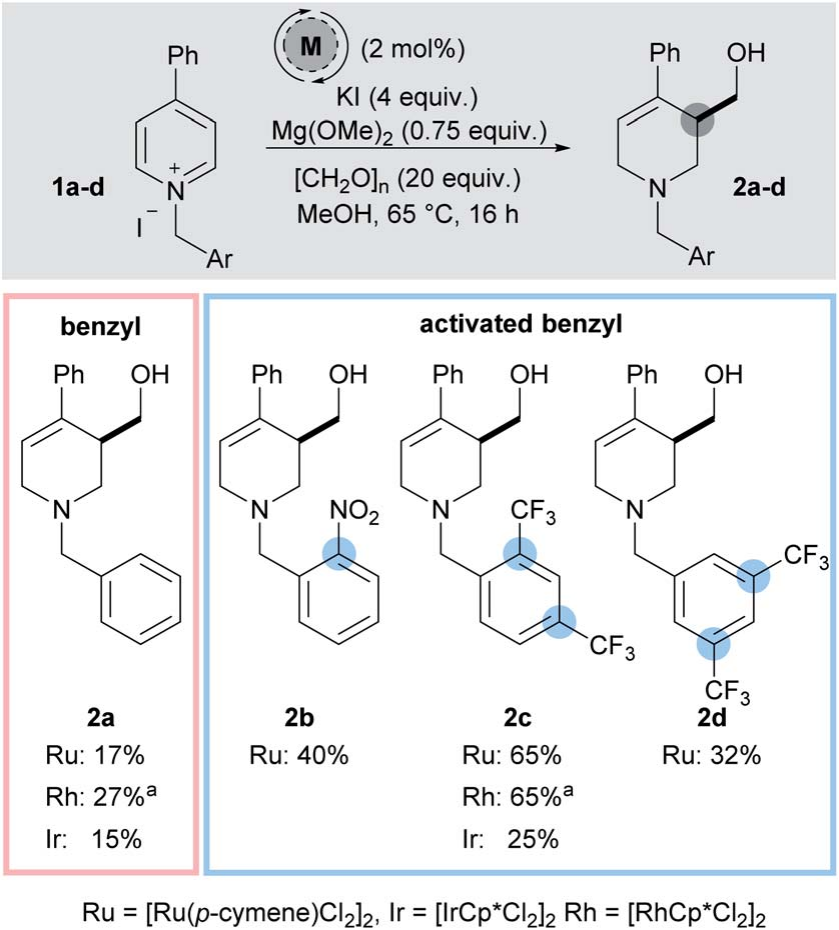
Screening of activating groups. ^*a*^ In the absence of KI.

While our initial work reported 3-hydroxymethylation of pyridines, the requirement of a strongly electron withdrawing group in the 4-position is a significant limitation. Groups such as esters and ketones work well, however any less electron-withdrawing substituents result in either low yielding or completely inactive pyridines. For example 4-phenylpyridine was poorly active under the reaction conditions (giving product 2a in 15% yield using an iridium catalyst and 27% yield using a rhodium catalyst, Scheme 2) and pyridines with alkyl and heteroatom substituents were completely inactive. 4-Aryl substituents are of particular interest as this motif can be found in molecules such as the anti-depressant drug Paroxetine (Scheme 1).

## Results and discussion

In order to address this limitation, we set about developing a single point activation strategy that would enable the use of more electron-rich pyridines, such as 4-phenyl pyridine. From analysis of crude reaction mixtures, it was apparent that the initial dearomative addition of a metal hydride to the pyridine C-2 position was the troublesome step when using less electron-poor substrates. Therefore, our first approach was to utilise metals which have a stronger reductive profile while using *N*-benzyl-4-phenylpyridinium iodide 1a as a test substrate (Scheme 2).^17^ In this case, both rhodium (27%) and ruthenium (17%) were found to give higher yields of 2a than iridium (15%).

To further increase the electron deficiency of the heteroaromatic system, while maintaining a single point of activation (*i.e.* quaternisation), analogous substrates bearing electron-deficient benzyl groups were then prepared and subjected to the metal-catalysed conditions. Using [Ru(*p*-cymene)Cl_2_]_2_ as the pre-catalyst, pleasingly the 2-nitrobenzyl substrate 1b was found to give the product 2b in a higher 40% yield, as did the 2,4-bistrifluoromethyl substrates 1c (2c, 65%) and 3,5-bistrifluoromethyl substrates 1d (2d, 32%). [IrCp*Cl_2_]_2_ was found to generate a far less proficient catalytic species, delivering 2c in only 25% yield. Finally, [RhCp*Cl_2_]_2_ was also found to deliver 2c in a good yield (65%). After deciding that the 2,4-bistrifluoromethyl activating group gave the best yield, substrate 1c was taken as a model substrate and subjected to further optimisation and screening (Table 1). Ru catalysis was studied further because it is significantly less expensive than Rh.

**Table 1:** Screening of reaction conditions

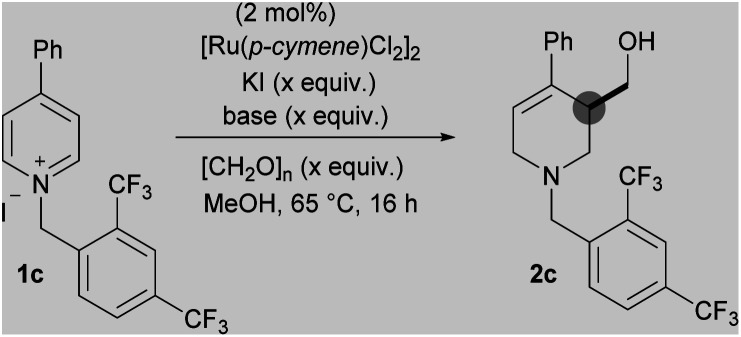					
Entry	KI equiv.	[CH_2_O]_*n*_ equiv.	Base	Base equiv.	Yield of 2c^a^ (%)
1	4	20	Mg(OMe)_2_	0.75	65
2	2	20	Mg(OMe)_2_	0.75	60
3	0	20	Mg(OMe)_2_	0.75	40
4^b^	0	20	Mg(OMe)_2_	0.75	42
5	6	20	Mg(OMe)_2_	0.75	50
6^c^	4	20	Mg(OMe)_2_	0.75	61
7	4	20	KOMe	1.5	24
8	4	20	Mg(OMe)_2_	0.5	64
9	4	20	Mg(OMe)_2_	1	70
10	4	20	Mg(OMe)_2_	2	70
11	4	10	Mg(OMe)_2_	1	65
12	4	30	Mg(OMe)_2_	1	72 (69)^d^

a ^1^H NMR yield using trimethoxybenzene as internal standard.

b Using [Ru(*p*-cymene)I_2_]_2_ as catalyst.

c Using the bromide salt of 1c.

d Isolated yield.

Initially, the role of KI additive, thought to improve the reducing ability of the metal hydride, was investigated; relative to our initial conditions using 4 equivalents,^18,19^ the absence of any KI (40%) or the presence of 2 equivalents (60%) led to a reduced yield of 2c (entry 2 and 3). However, increasing KI loading to 6 equivalents (50%) also led to a decrease in yield (entry 5). Using the bromide salt of 1c as starting material, instead of the iodide salt, also led to a slight decrease in yield (61%, entry 6).

**Scheme 3:**
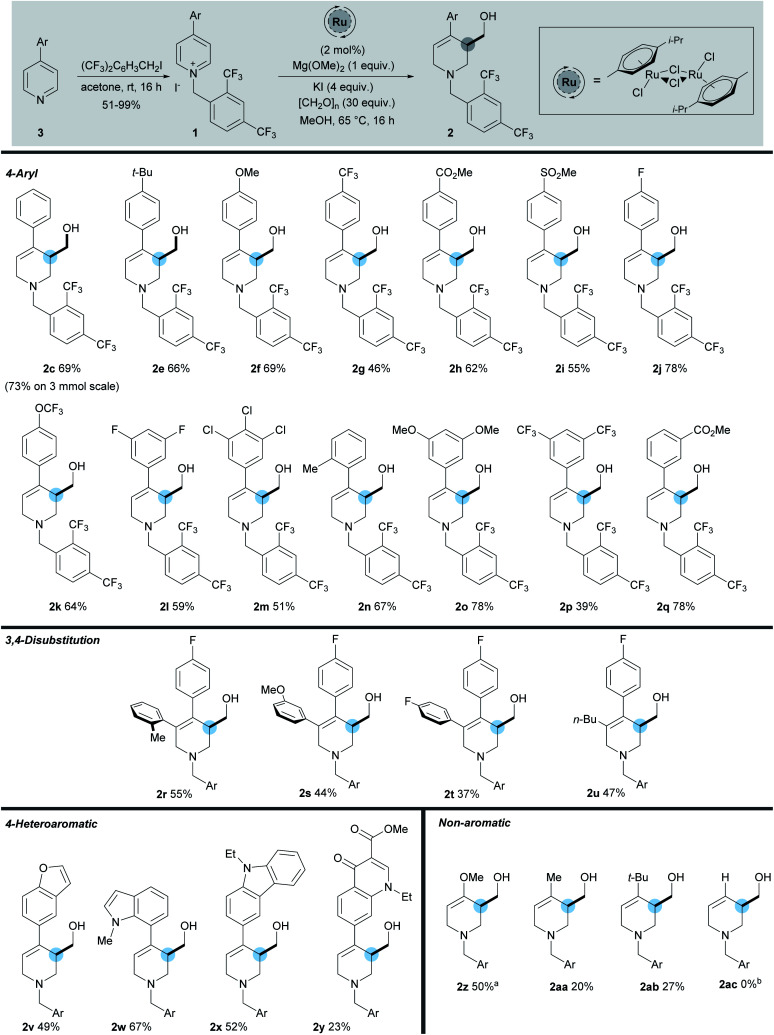
Scope of reaction. ^*a*^ Using [RhCp*Cl_2_]_2_ catalyst, [CH_2_O]_*n*_ (60 equiv.), Mg(OMe)_2_ (1.5 equiv.), 45 °C. ^*b*^ Reaction of 1ac gave a complex mixture of products.

Changing the base to potassium methoxide resulted in a drop in yield to 24%. Decreasing the loading of magnesium methoxide to 0.5 equivalents had a limited effect (64%) but increasing the loading to 1 equivalent increased the yield to 70%; a further increase of base equivalents did not increase the yield (entry 8, 9 and 10). Finally, decreasing the paraformaldehyde equivalents to 10 led to a reduction in yield (65%) while increasing to 30 equivalents increased the yield to 72%, giving an isolated yield of 69% (entries 11 and 12). Note that it was necessary to add excess formaldehyde in order to ensure complete reaction of the enamine formed *in situ*. Although the optimised conditions differ from our initial iridium catalysed work, we believe that mechanistically the reaction process proceeds in a similar fashion (see ESI†).^13^ With the optimal N-activating group and conditions in hand, a range of 4-aryl substituted pyridinium salts were subjected to the reaction conditions (Scheme 3).

The starting materials were prepared in one step from 2,4-bistrifluoromethylbenzyl iodide and the corresponding pyridine. While the model 4-phenylpyridine 1c gave the product 2c in 69% yield, scaling this reaction to 3 mmol, led to the formation of 2c in an increased yield of 73%. Pleasingly we found that a number of both electron-donating (methyl, *tert*-butyl, and methoxy) and electron-withdrawing (trifluoromethyl, fluoro, chloro, methyl ester, and sulfone) substituents were well tolerated on the C-4 aromatic group, giving the products in a range of yields (39–78%). Within the C-4 aromatic group, substitution was tolerated at the *ortho*, *meta*, and *para* positions, together with 3,5-disubstitution and 3,4,5-trisubstitution. Of note, substrate 2j bearing a 4-fluorophenyl group was formed in 78% yield and was used as the key step in the synthesis of (±)-Paroxetine, *vide infra*. In addition, substitution at the 3-position of the pyridine is also tolerated (2r–2u), giving the hydroxymethylated product in reasonable yields (37–55%). In this position aromatic groups bearing *ortho*, *meta*, and *para* substitution along with a *n*-butyl group were all shown to be viable substrates. The activation strategy utilising the 2,4-bistrifluoromethylbenyl group was then extended to enable the use of pyridines substituted at the 4-position with strongly electron-rich heterocycles. These substrates are completely inactive when using the standard *N*-benzyl activating strategy which shows the importance of the electron withdrawing *N*-benzyl derivative described here. Carbazole (2x 52%), indole (2w, 67%), and benzofuran (2v, 49%) C-4 substituents were all used successfully to give the hydroxymethylated products in good yields. These results highlight the ability of this methodology to selectively activate and functionalise pyridines in the presence of other heteroaromatic rings. Furthermore, the *N*-2,4-bistrifluorobenzyl salt of Rosoxacin ester, a commercially available quinoline antibiotic, was subjected to the reaction conditions and the desired product 2y was isolated in 23% yield, showing that late stage derivatisation of important pharmaceutically relevant molecules can be achieved with this methodology. Moreover, the highly electron-rich 4-methoxypyridine was successfully activated using our strategy to give enol ether 2z in 50% yield. While the corresponding 4-alkyl products 2aa and 2ab, bearing a methyl and *tert*-butyl group respectively, were also formed, the isolated yields were only 20% and 27% respectively. However, despite these lower yields, they do demonstrate a proof of concept that simple C-4 alkyl-substituted pyridines can now be utilised in this methodology for the first time. Unfortunately substrates lacking a 4-substituent did not undergo hydroxymethylation successfully and gave complex mixtures of products (2ac); we plan to tackle this issue in our future work.

**Scheme 4:**
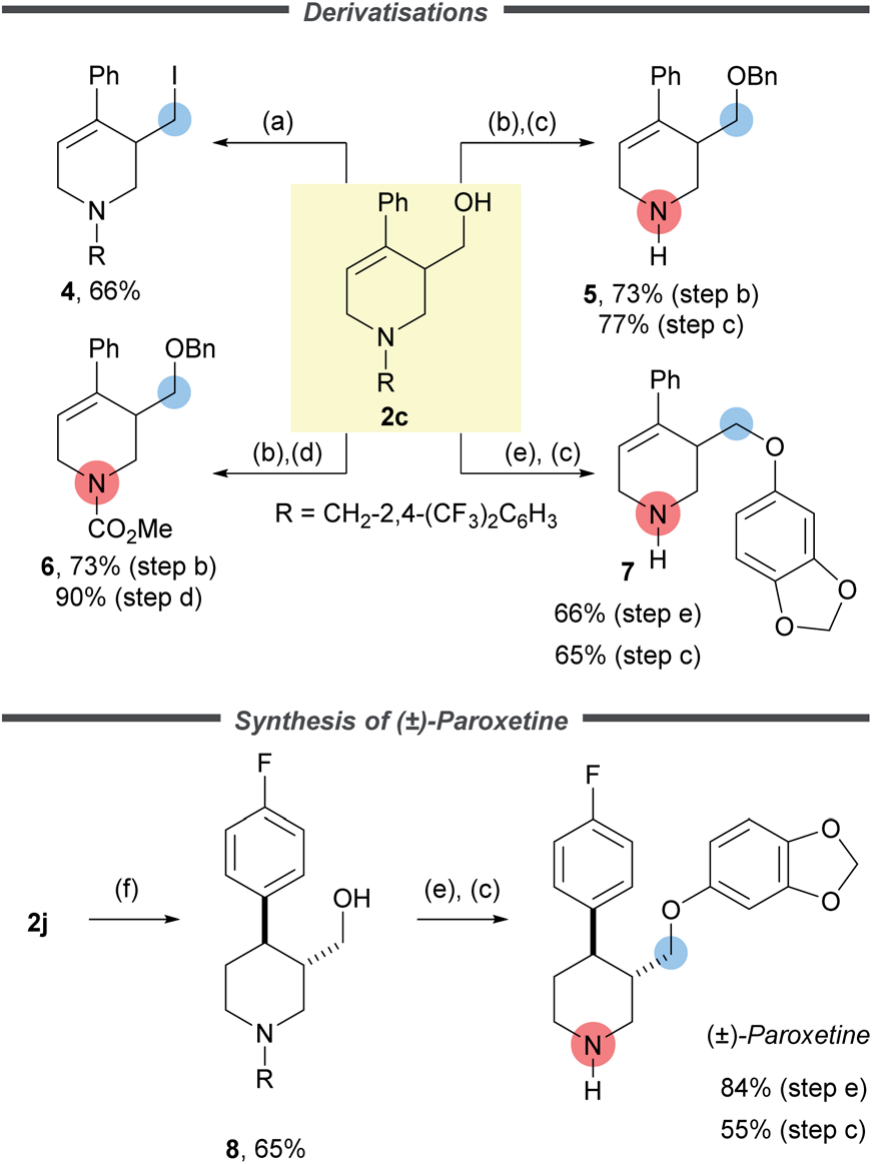
Deprotection and functionalisation of the products. (a) PPh_3_ (2.2 equiv.), I_2_ (2.2 equiv.), imidazole (2.5 equiv.) CH_2_Cl_2_, 0 °C to r.t., 2 h. (b) NaH (10 equiv.), 15-crown-5 (2.0 equiv.), BnBr (10 equiv.), 0 °C to r.t., 2 h. (c) 1-Chloroethyl chloroformate (8.0 equiv.), DCE, 120 °C, 16 h then MeOH, 65 °C, 4 h. (d) Methyl chloroformate (8.0 equiv.), DCE, 120 °C, 16 h. (e) PPh_3_ (1.2 equiv.) sesamol (2.0 equiv.) DIAD (1.2 equiv.), THF, 0 → 50 °C, 2 h. (f) [Ir(cod)(PCy_3_)(py)]PF_6_ (20 mol%), H_2_, CH_2_Cl_2_, r.t., 20 h. R = CH_2_(CF_3_)_2_C_6_H_3_.

To highlight the versatility of the products, further synthetic transformations were undertaken on a representative example 2c (Scheme 4). The newly installed CH_2_OH group was converted to the corresponding alkyl iodide 4*via* an Appel reaction in good yield (66%). Importantly, removal of the activating group was achieved easily through protection of the free –OH group with a benzyl group followed by either treatment with 1-chloroethyl chloroformate and subsequent reaction of the intermediate carbamate with methanol to furnish the free amine 5 in 56% yield over two steps. Alternatively treatment with methyl chloroformate, allowed a protecting group switch to form carbamate 6 in good yield (66%) over two steps. A Mitsunobu reaction of 2c using sesamol, followed by cleavage of the activating group delivered the corresponding amine 7 in 43% yield over two steps. These synthetic manipulations, together with the easy removal of the benzyl derived activating group, enables the wide array of products to be converted into highly useful small molecules.

In order to show the utility of this methodology, we chose to prepare Paroxetine, which is a selective serotonin reuptake inhibitor used as a pharmaceutical drug to treat a variety of conditions.^20^ Starting with commercially available 4-bromopyridine hydrochloride we prepared (±)-Paroxetine using our methodology in only six steps and 20% yield (Scheme 4). Following the preparation of 2j, three simple and efficient transformations gave Paroxetine. The first of these was a key substrate controlled hydrogenation using Crabtree's catalyst to deliver the piperidine 8 (3 : 1 dr, from which the pure *trans* isomer of 8 was isolated in 65% yield). Finally, the synthesis was completed with a Mitsunobu reaction to install the electron rich aryl group (84%) and removal of the activating group using ACE-Cl and methanol (55%). In order to probe the mechanism of the reaction we have subjected 1j to the reaction using [CD_2_O]_*n*_ and observed deuterium incorporation on C-2, C-6 and the exocyclic hydroxymethyl group. This was consistent with our proposed mechanism and also provided a convenient method to access deuterated Paroxetine if so desired (see ESI pp56†).

## Conclusions

In conclusion, we have developed a new catalytic system and shifted the boundaries of the dearomative hydroxymethylation reaction, expanding the scope far beyond what was possible in our initial work. Ranging from 4-arylpyridines to the electron-rich 4-methoxypyridine the hydroxymethylation proceeded in good yields. The key aspect of the work involves the use of a ruthenium catalyst in conjunction with a highly electron-deficient 2,4-bistrifluoromethylbenzyl activating group. This bespoke activating group can be readily removed to furnish the free amine. Finally, we have utilised this highly useful methodology in the synthesis of (±)-Paroxetine and plan to employ it further to make more medicinally relevant molecules and natural products.

## Conflicts of interest

There are no conflicts of interest.

## Supplementary Material

SC-012-D0SC05656A-s001
