# Supplementary material for: Single point activation of pyridines enables reductive hydroxymethylation
Source: Chem Sci. 2020 Nov 16;12(2):742–6. doi: 10.1039/d0sc05656a (PMC8178984; doi:10.1039/d0sc05656a)
Supplement: SC-012-D0SC05656A-s001 [file SC-012-D0SC05656A-s001.pdf]

Electronic Supplementary Material (ESI) for Chemical Science.
This journal is © The Royal Society of Chemistry 2020

Single Point Activation of Pyridines Enables Reductive Hydroxymethylation

Bruno Marinic,[a] Hamish B. Hepburn,[a] Alexandru Grozavu,[a] Mark Dow,[b] and Timothy J. Donohoe*[a]

[a] Chemistry Research Laboratory, University of Oxford, Mansfield Road, Oxford, UK OX1 3TA

[b] AstraZeneca, Silk Road, Macclesfield, UK

Supplementary Information

Contents

General Experimental Techniques S2
- Chemicals and solvents S2
- Glassware and reaction conditions S2
- Analytical techniques S2
- Chromatography S2

The Synthesis of Benzyl Iodides S3
- General Procedure A: Preparation of benzyl iodides S3

The Synthesis of Pyridine Precursors S5
- General Procedure B: Preparation of 4-*Aryl* Pyridines S5
- General Procedure C: Preparation of Bisarylpyridines S10

The Synthesis of Pyridinium Salts S15
- General Procedure D: Preparation of benzyl pyridinium iodide salts. S15

The Synthesis of Tetrahydropyridines S32
- General procedure E: S32

Derivatisation of products S50

Synthesis of Paroxetine S54

Proposed reaction mechanism S56

NMR Spectra S58

General Experimental Techniques

Chemicals and solvents

Unless stated otherwise, all chemicals were purchased from commercial suppliers (Sigma-Aldrich, Fluorochem, Alfa Aesar) and used without further purification. The magnesium methoxide was purchased from Alfa Aesar as a 6-10% w/w solution in methanol and was titrated using EDTA in the presence of Eriochrome Black T as an indicator. Prilled paraformaldehyde (95% purity) was used in all cases.

Glassware and reaction conditions

Reactions were carried out in oven-dried microwave vials under an atmosphere of air unless otherwise stated.

Analytical techniques

^{1}H, ^{19}F and ^{13}C NMR spectra were recorded on a Bruker AVIII400 Spectrometer (400 MHz 376 MHz and 100 MHz respectively) or a Bruker AVII500 (^{1}H: 500 MHz and ^{13}C: 126 MHz) in $CDCl_3$ or DMSO-d_6, and referenced to residual solvent peaks. Chemical shifts δ are quoted in parts per million (ppm) to the nearest 0.01 for ^{1}H and 0.1 for ^{13}C, coupling constants *J* are quoted in Hz to the nearest 0.1 and splitting are recorded as singlet (s), doublet (d), triplet (t), quartet (q), doublet of a doublet (dd), doublet of a triplet (dt), triplet of a doublet (td), triplet of a triplet (tt), doublet of a doublet of a doublet (ddd), doublet of a doublet of a triplet (ddt) and multiplet (m). Assignments were based upon COSY, HSQC and HMBC experiments. Infrared spectra were recorded on a Bruker Tensor 27 FT-IR spectrometer fitted with an Attenuated Total Reflectance (ATR) sampling accessory. Absorption maxima are quoted in wavenumbers (cm^{-1}). High resolution mass spectra were recorded on a Bruker MicroTof (resolution = 10000 FWHM). Melting points (m.p.) were obtained using a Lecia VMGT heated-stage microscope and are uncorrected.

Chromatography

Analytical thin layer chromatography was performed on pre-coated silica gel aluminium sheets from Merck (TLC Silica Gel 60 F_{254s}). Spots were visualized either by the quenching of UV fluorescence or by staining with phosphomolybdic acid, potassium permanganate or vanillin solutions. Preparative flash column chromatography (FCC) was carried out using Geduran Silica Gel 60 (40 µm – 63 µm) from Merck.

The Synthesis of Benzyl Iodides

General Procedure A: Preparation of benzyl iodides

To a solution of the corresponding benzyl chloride or bromide (1.00 equiv.) in acetone (0.5 M) at 0 °C was added NaI (2.00 equiv.) and the reaction mixture was stirred in the dark at room temperature for 16 hours. Reaction was quenched with brine (20 mL) and the aqueous layer extracted with Et_2O (2 x 40 mL). The combined organic layers were dried ($MgSO_4$), filtered and concentrated *in vacuo*. FCC (0-2% Et_2O in Pentane) afforded the benzyl iodides.

2-Nitrobenzyl iodide (S1)

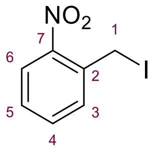

The title compound was prepared by General Procedure A using 2-nitrobenzyl bromide (6.48 g, 30.0 mmol) to give the *iodide* **S1** (7.10 g, 90%) as an orange solid. Spectroscopic data was consistent with that reported in the literature.[1]

^{1}H NMR (400 MHz, $CDCl_3$) δ 8.04 (dd, *J* = 8.2, 1.4 Hz, 1H, C^6**H**), 7.60 – 7.40 (m, 3H, 3 x ArC**H**), 4.79 (s, 2H, C^1**H₂**).

^{13}C NMR (101 MHz, $CDCl_3$) δ 147.5 (C^7), 135.1 (C^2), 134.0 (ArCH), 132.4 (ArCH), 129.2 (ArCH), 125.9 (ArCH), 0.0 (C^1H_2).

2,4-*Din*itrobenzyl iodide (S2)

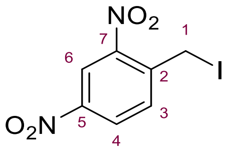

Title compound was prepared by General Procedure A using 2,4-dinitrobenzyl bromide (217 mg, 1.00 mmol) to give the *iodide* **S2** (273 mg 89%) as a yellow solid.

^{1}H NMR (400 MHz, $CDCl_3$) δ 8.90 (d, *J* = 2.4 Hz, 1H, C^6**H**), 8.40 (dd, *J* = 8.5, 2.4 Hz, 1H, C^4**H**), 7.73 (d, *J* = 8.5 Hz, 1H, C^3**H**), 4.84 (s, 2H, 2 x C^1**H_2**).

^{13}C NMR (101 MHz, $CDCl_3$) δ 141.93 (C^2), 133.67(C^6H), 128.01 (C^4H), 121.52 (C^3H), –2.78 (C^1H_2), signal for C^5 and C^7 not observed.

HRMS (ESI): Exact mass calculated for $C_7H_4O_4N_2{}^{127}I$ $[M]^-$: 306.92212, found: 306.92142;

IR (neat) (cm^{-1}): 3124, 2924, 1704, 1603, 1536, 1346, 1198, 1085, 892, 506.

3,5-bis(Trifluoromethyl)benzenyl iodide (S3)

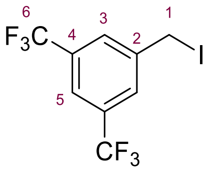

Title compound was prepared using General Procedure A using 3,5-bis(trifluoromethyl)benzyl bromide (0.37 mL, 2.00 mmol) to yield the *iodide* **S3** (700 mg, 99%) as a yellow solid. Spectroscopic data was consistent with that reported in the literature.[2]

^{1}H NMR (400 MHz, $CDCl_3$) δ 7.81 (s, 2H, 2 x C^3**H**), 7.76 (s, 1H, C^5**H**), 4.49 (s, 2H, C^1**H_2**).

[1] M. Alajarin, A. Pastor, R.-A. Orenes, J. W. Steed, and R. Arakawa, *Chem. Eur. J.* **2004** 10, 1383–1397.

[2] D. F. J. Caputo, C. Arroniz, A. B. Dürr, J. J. Mousseau, A. F. Stepan, S. J. Mansfield, and E. A. Anderson, *Chem. Sci.*, 2018,**9**, 5295-5300

^{13}**C NMR** (101 MHz, $CDCl_3$) δ 142.1, (C^2), 132.4 (q, *J* = 33.6 Hz, 2 x C^4), 128.9 (q, *J* = 3.1 Hz, 2 x C^{3}H), 123.1 (q, *J* = 273 Hz, 2 x C$^6F_3$), 122.0 – 121.8 (m, C^{5}H), 1.3 (C$^1H_2$).

2,4-bis(Trifluoromethyl)benzenyl iodide (S4)

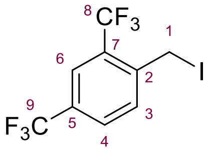

Title compound was prepared using General Procedure A using 2,4-bis(trifluoromethyl)benzyl bromide (0.38 mL, 2.00 mmol) to yield the *iodide* **S4** (700 mg, 99%) as a yellow oil.

HRMS (ESI): Exact mass calculated for $C_9H_5F_6{}^{127}I$ $[M]^+$:

^{1}**H NMR** (500 MHz, $CDCl_3$) δ 7.85 (s, 1H, C^6**H**), 7.76 (d, *J* = 8.3 Hz, 1H, C^4**H**), 7.70 (d, *J* = 8.2 Hz, 1H, C^3**H**), 4.61 (s, 2H, C^1**H$_2$**).

^{13}**C NMR** (126 MHz, $CDCl_3$) δ 142.3 (C^2), 133.6 (C^{3}H), 130.4 (q, *J* = 33.7 Hz, C^5/ C^7), 129.4 (q, *J* = 3.7 Hz, C^{4}H), 128.4 (q, *J* = 31.6 Hz, C^5/C^7), 124.05 – 123.52 (m, C^{6}H), 123.5 (q, *J* = 274.5 Hz, C$^8F_3$/ C$^9F_3$), 123.30 (q, *J* = 272.3 Hz, C$^8F_3$/ C$^9F_3$), –2.53 (q, *J* = 2.5 Hz, C$^1H_2$).

^{19}**F NMR** (377 MHz, $CDCl_3$) δ –60.7, –63.0.

IR (neat) (cm^{-1}): 1628, 1345, 1273, 1175, 1119, 1082, 1053, 913, 844, 671

The Synthesis of Pyridine Precursors

General Procedure B: Preparation of 4-*Aryl* Pyridines

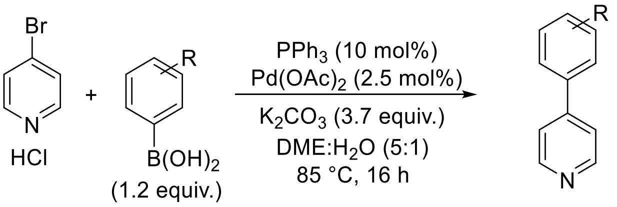

4-Bromopyridine hydrochloride (1.00 equiv.), triphenylphosphine (20 mol%), boronic acid (1.20 equiv.), and potassium carbonate (3.70 equiv.) were added to a 3-necked flask fitted with a reflux condenser. The vessel was evacuated and backfilled with argon three times then dimethoxyethane and water (5:1 0.1 M) were added and solution purged with argon for 10 minutes. Palladium(II) acetate (2.5 mol%) was added and the solution heated at 85 °C for 16 hours. The solution was cooled, diluted with 50 mL of water, and extracted with EtOAc (50 mL) three times. The organic layers were combined, dried ($MgSO_4$), filtered, and concentrated *in vacuo*. The crude material was purified by flash column chromatography to give the product.

4-(4-(*tert*-Butyl)phenyl)pyridine (3e)

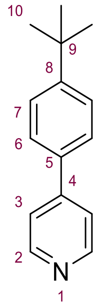

The title compound was prepared according to General Procedure B using 4-*tert*-butylphenylboronic acid (2.14 g, 12.0 mmol) and was purified by FCC (10% - 20% EtOAc in pentane) to give *pyridine* **3e** (1.78 g, 83%) as a cream solid. The spectroscopic data was consistent with previous reports.[3]

4-(4-Methoxyphenyl)pyridine (3f)

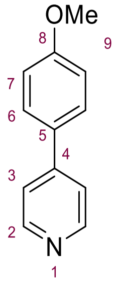

The title compound was prepared according to General Procedure B using 4-methoxyphenylboronic acid (547 mg, 3.60 mmol) and was purified by FCC (10% - 20% EtOAc in pentane) to give *pyridine* **3f** (1.60 g, 76%) as a white solid. The spectroscopic data was consistent with previous reports.[4]

---

[3] S. Jonsson, F. G. J. Odille, P.-O. Norrby, and K. Wärnmark, *Org. Biomol. Chem.*, 2006, **4**, 1927–1948
[4] X. Zhang and A. McNally, *Angew. Chem. Int. Ed.*, 2017, **56**, 9833 –9836

4-(4-Trifluoromethyl)pyridine (3g)

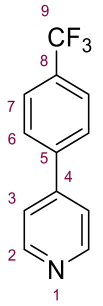

The title compound was prepared according to General Procedure B using 4-trifluoromethylbenzene boronic acid (2.28 g, 12.0 mmol) and was purified by FCC (30% -50% EtOAc in pentane) to give *pyridine* **3g** (1.70 g, 76% yield) as a yellow glass. The spectroscopic data was consistent with previous literature reports.[5]

4-Pyridin-4-yl benzoic acid methyl ester (3h)

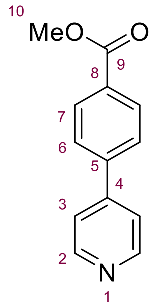

The title compound was prepared according to General Procedure B using 4-methoxycarbonylphenylboronic acid (2.16 g, 12.0 mmol) and purified by FCC (30%-50% EtOAc in pentane) to give *pyridine* **3h** (1.60g, 76% yield) as a white solid. The spectroscopic data was consistent with previous literature reports.[6]

4-(4-Methylsulfonylphenyl)pyridine (3i)

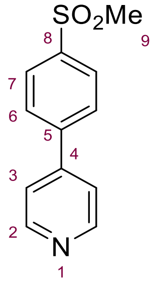

The title compound was prepared according to General Procedure B using 4-methysulfonylphenylboronic acid (2.40 g, 12.0 mmol) and purified by FCC (10%-20% acetone in CH_2Cl_2) to give *pyridine* **3i** (1.56 g, 67% yield) as a white solid. The spectroscopic data was consistent with previous literature reports.[7]

---

[5]H. Y. Lee; X. Jiang, D. Lee, *Org. Lett.* **2009**, 11, 2065 - 2068

[6]J. Malineni, R. L. Jezorek, N. Zhang, and V. Percec, *Synthesis*, 2016, **48**, 2795–2807

[7]F. Pettersson, P. Svensson, S. Waters, N. Waters, and C. Sonesson, *J. Med. Chem.* 2012, ***55***, 3242-3249.

4-(4-Fluorophenyl)pyridine (3j)

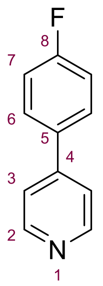

The title compound was prepared according to General Procedure B using 4-fluorobenzene boronic acid (1.68 g, 12.0 mmol) and was purified by FCC (10% - 20% EtOAc in pentane) to give *pyridine* **3j** (1.56 g, 90% yield) as a colourless glass. The spectroscopic data was consistent with previous literature reports.[8]

4-(4-(trifluoromethoxy)phenyl)pyridine (3k)

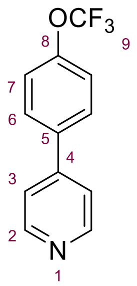

The title compound was prepared according to General Procedure B using 4-trifluoromethoxyphenylboronic acid (2.47 g, 12.0 mmol) and was purified by FCC (10% - 20% EtOAc in pentane) to give *pyridine* **3k** (2.19 g, 92%) as a yellow oil.

HRMS (ESI): Exact mass calculated for $C_{13}H_8F_3NO$ $[M+H]^+$:240.0631, found: 240.0632

^{1}H NMR (400 MHz, $CDCl_3$) δ 8.72 – 8.65 (m, 2H, 2 x C^2**H**), 7.70 – 7.62 (m, 2H, 2 x C^6**H**), 7.51 – 7.45 (m, 2H, 2 x C^3**H**), 7.38 – 7.30 (m, 2H, 2 x C^7**H**).

^{13}C NMR (101 MHz, $CDCl_3$) δ 150.5 (2 x C^2H), 150.1 (C^8), 147.2 (C^4), 136.9 (C^5), 128.7 (2 x C^6H), 121.7 (2 x C^3H), 121.6 (2 x C^7H), 120.6 (q, *J* = 257.7 Hz, C^9F_3).

^{19}F NMR (377 MHz, $CDCl_3$) δ –57.8.

IR (neat) (cm^{-1}): 2982, 1596, 1516, 1488, 1252, 1207, 1154, 1110, 805, 674.

4-(3,5-Difluorophenyl)pyridine (3l)

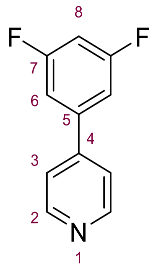

The title compound was prepared according to General Procedure B using 3,5-difluorobenzne boronic acid (1.89 g, 12.0 mmol) and was purified by FCC (10% - 20% EtOAc in pentane) to give *pyridine* **3l** (1.67 g, 87% yield) as a cream solid. The spectroscopic data was consistent with previous literature reports.[4]

[8]S. Panda, A. Coffin, Q. N. Nguyen, D. J. Tantillo, and J. M. Ready, *Angew. Chem. Int. Ed.* **2016**, *55*, 2245-2249

4-(3,4,5-Trichloropheny)pyridine (3m)

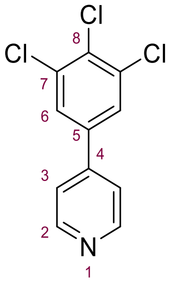

The title compound was prepared using General Procedure B on a 2 mmol scale using 3,4,5-trichlorobenzne boronic acid (540 mg, 2.40 mmol) and purified by FCC (30%-50% EtOAc in pentane) to give *pyridine* **3m** (426 mg, 82% yield) as a white solid. The spectroscopic data was consistent with previous literature reports.[9]

4-(2-Methyphenyl)pyridine (3n)

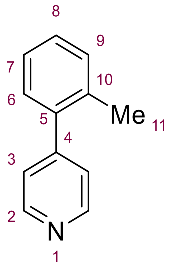

The title compound was prepared according to General Procedure B using 2-methylphenylboronic acid (1.63 g, 12.0 mmol) and purified by FCC (10%-20% EtOAc in pentane) to give *pyridine* **3n** (0.94 g, 55% yield) as a colourless oil. The spectroscopic data was consistent with previous literature reports.[4]

4-(3,5-Dimethoxyphenyl)pyridine (3o)

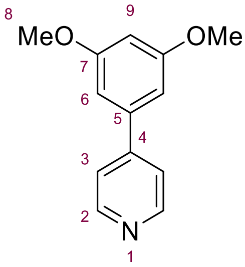

The title compound was prepared according to General Procedure B using 4-trifluoromethoxyphenylboronic acid (2.19 g, 12.0 mmol) and was purified by FCC (20% - 50% EtOAc in pentane) to give *pyridine* **3o** (1.74 g, 81%) as a white solid.

m.p. (EtOAc): 56 – 58 °C

HRMS (ESI): Exact mass calculated for $C_{13}H_{14}NO_2$ $[M+H]^+$: 216.1019, found: 216.1020.

^{1}H NMR (400 MHz, $CDCl_3$) δ 8.68 – 8.62 (m, 2H, 2 x C^2**H**), 7.50 – 7.44 (m, 2H, 2 x C^3**H**), 6.75 (d, *J* = 2.2 Hz, 2H, 2 x C^6**H**), 6.53 (t, *J* = 2.3 Hz, 1H, C^9**H**), 3.85 (s, 6H, 3 x C^8**H_3**).

^{13}C NMR (101 MHz, $CDCl_3$) δ 161.5 (2 x C^7), 150.4 (2 x C^2H), 148.5 (C^4), 140.5 (C^5), 121.9 (2 x C^3H), 105.4 (2 x C^6H), 100.9 (C^9H), 55.6 (2 x C^8H_3).

IR (neat) (cm^{-1}): 2979, 2845, 1586, 1336, 1226, 1198, 1165, 1029, 852, 818.

---

[9]A. Grozavu, H. B. Hepburn, E. P. Bailey, P. J. Lindsay-Scott, T. J. Donohoe, *Chem. Sci.*, 2020, **11**, 8595-8599.

4-(3,5-bis(Trifluoromethyl)phenyl)pyridine (3p)

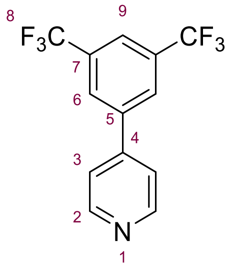

The title compound was prepared according to General Procedure B using 3,5-bis(trifluoromethyl)phenylboronic acid (3.00 g, 12.0 mmol) and purified by FCC (10%-30% EtOAc in pentane) to give *pyridine* **3p** (2.12 g, 73% yield) as a white solid. The spectroscopic data was consistent with previous literature reports[10]

3-Pyridin-4-yl benzoic acid methyl ester (3q)

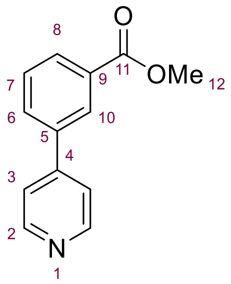

The title compound was prepared according to General Procedure B using 3-methoxycarbonylphenylboronic acid (2.16 g, 12.0 mmol) and purified by FCC (30%-50% EtOAc in pentane) to give *pyridine* **3q** (1.53 g, 72% yield) as a white solid. The spectroscopic data was consistent with previous literature reports.[11]

3-Bromo-4-(4-fluorophenyl)pyridine (S5) and 3,4-bis(4-fluorophenyl)pyridine (3t)

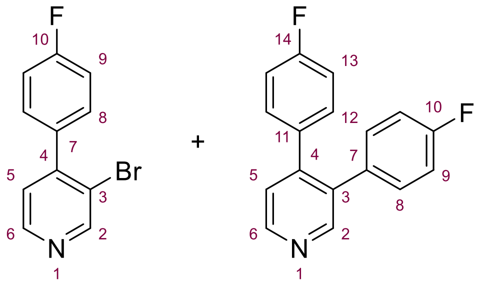

The title compound were prepared according to General Procedure B using 3,4-dibromopyridine (2.37 g, 10.0 mmol) and 4-fluorophenylboronic acid (1.52 g, 10.9 mmol) and was purified by FCC (2% EtOAc in pentane) to give *arylpyridine* **S5** (1.27 g, 51%) as a white solid followed by *bisarylpyridine* **3t** (182 mg, 7% yield) as a white solid.

m.p. (EtOAc): 66 - 68 °C

HRMS (ESI): Exact mass calculated for $C_{11}H_8BrFN$ $[M+H]^+$: 251.9819, found: 251.9821

^{1}H NMR (400 MHz, $CDCl_3$) δ 8.82 (d, *J* = 0.6 Hz, 1H, C^2**H**), 8.55 (d, *J* = 4.9 Hz, 1H, C^5**H**), 7.49 – 7.37 (m, 2H, 2 x C^8**H**), 7.25 (dd, *J* = 4.9, 0.6 Hz, 1H, C^6**H**), 7.22 – 7.11 (m, 2H, 2 x C^9**H**).

^{13}C NMR (101 MHz, $CDCl_3$) δ 163.1 (d, *J* = 249.0 Hz, C^{10}), 152.8, (C^2H), 148.9 (C^4), 148.5 (C^5H), 134.3 (d, *J* = 3.5 Hz, C^7), 130.9 (d, *J* = 8.4 Hz, 2 x C^8H), 125.7 (C^6H), 121.0 (C^3), 115.7 (d, *J* = 21.7 Hz, 2 x C^9H).

[10] Z.-Y. Liu, Z.-H. Wen, and X.-C. Wang, *Angew. Chem. Int. Ed.l*, 2017, **56**, 5817-5820

[11]K. Ohkura, M. Terashima, Y. Kanaoka, and K. Seki, *Chem. Pharm. Bull.* 1993, **41**, 1920-1924.

^{19}**F NMR** (377 MHz, $CDCl_3$) δ –112.3 (tt, *J* = 8.6, 5.4 Hz).
IR (neat) (cm^{-1}): 3048, 3011, 2161, 1970, 1605, 1583, 1510, 1470, 1228, 831.

m.p. (EtOAc): 108 - 110 °C
HRMS (ESI): Exact mass calculated for $C_{17}H_{12}F_2N$ $[M+H]^+$: 268.0932, found: 268.0932
^{1}**H NMR** (400 MHz, $CDCl_3$) δ 8.62 (d, *J* = 5.1 Hz, 1H, C^5**H**), 8.61 (d, *J* = 0.7 Hz, 1H, C^2**H**), 7.31 (dd, *J* = 5.0, 0.7 Hz, 1H, C^6**H**), 7.16 – 7.05 (m, 4H, 2 x C^9**H** + 2 x C^{13}**H**), 7.04 – 6.92 (m, 4H, 2 x C^8**H** + 2 x C^{12}**H**).
^{13}**C NMR** (101 MHz, $CDCl_3$) δ 162.5 (d, *J* = 248.5 Hz, C^{10}/C^{14}), 162.3 (d, *J* = 247.6 Hz, C^{10}/C^{14}), 151.0 (C^2H), 149.0 (C^5H), 146.7 (C^4), 134.8 (C^3), 134.4 (d, *J* = 3.3 Hz, C^7/C^{11}), 133.5 (d, *J* = 3.3 Hz, C^7/C^{11}), 131.4 (d, *J* = 8.0 Hz, C^8H/C^{12}H), 131.0 (d, *J* = 8.3 Hz, C^8H/C^{12}H), 124.5 (C^6H), 115.5 (d, *J* = 22.0 Hz, C^9H + C^{13}H).
^{19}**F NMR** (377 MHz, $CDCl_3$) δ -113.4 – -113.6 (m), -114.4 – -114.6 (m).
IR (neat) (cm^{-1}): 3050, 3011, 2161, 2034, 1978, 1510, 1228, 1215, 828, 814.

General Procedure C: Preparation of Bisarylpyridines

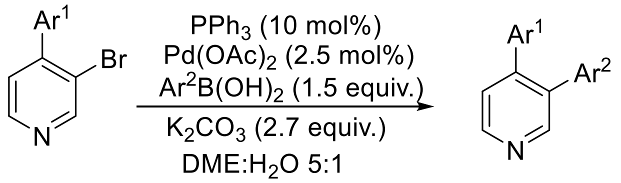

3-Bromo-4-(4-fluorophenyl)pyridine **S5** (1.5 mmol, 1.00 equiv.), triphenylphosphine (10 mol%), potassium carbonate (2.70 equiv.), and arylboronic acid (1.10 equiv.) were dissolved in a 5:1 mixture of DME:H_2O (0.1 M) which was then purged with Ar for 10 minutes. $Pd(OAc)_2$ was added and the solution heated for 16 hours at 85 °C. The solution was cooled, diluted with EtOAC and extracted three times with EtOAC. The combined organic layers were washed with brine, dried ($MgSO_4$), filtered and concentrated *in vacuo*. The crude material was purified by flash column chromatography to give the product.

4-(4-Fluorophenyl)-3-(o-tolyl)pyridine (3r)

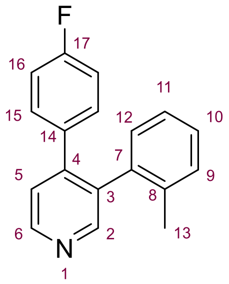

The title compound was prepared according to General Procedure C using 2-methylphenylboronic acid (150 mg, 1.10 mmol) and purified by FCC (10%-20% EtOAc in pentane) to give *pyridine* **3r** (370 mg, 94% yield) as a white solid.
m.p. (EtOAc) 110 - 112 °C;
HRMS (ESI): Exact mass calculated for $C_{18}H_{15}FN$ $[M+H]^+$: 268.1183, found: 268.1182.
^{1}**H NMR** (400 MHz, $CDCl_3$) δ 8.66 (d, *J* = 5.1 Hz, 1H, C^5**H**), 8.54 (d, *J* = 0.6 Hz, 1H, C^2**H**), 7.36 (dd, *J* = 5.1, 0.7 Hz, 1H, C^6**H**), 7.23 (dd, *J* = 7.3, 1.7 Hz, 1H, C^{12}**H**), 7.19 (td, *J* = 7.5, 1.6 Hz, 1H, C^{11}**H**), 7.15 – 7.07 (m, 4H, C^9**H** + C^{10}**H** + 2 x C^{15}**H**), 6.96 – 6.86 (m, 2H, 2 x C^{16}**H**), 1.87 (s, 3H, C^{13}**H$_3$**).
^{13}**C NMR** (101 MHz, $CDCl_3$) δ 162.6 (d, *J* = 248.4 Hz, C^{17}), 151.3 (C^2H), 148.9 (C^5H), 147.4 (C^4), 137.2 (C), 136.2 (C), 135.7 (C), 134.7 (d, *J* = 3.3 Hz, C^{14}), 130.8 (d, *J* = 8.7 Hz, ArCH + 2 x C^{15}H), 130.4 (ArCH), 128.2 (C^{12}H), 126.0 (C^{11}H), 124.0 (C^6H), 115.4 (d, *J* = 21.7 Hz, 2 x C^{16}H), 20.1 ($C^{13}H_3$).
^{19}**F NMR** (377 MHz, $CDCl_3$) δ -113.5 (tt, *J* = 8.8, 5.3 Hz).
IR (neat) (cm^{-1}): 2994, 1602, 1512, 1477, 1218, 831, 779, 756, 746, 729.

4-(4-Fluorophenyl)-3-(3-methoxyphenyl)pyridine (3s)

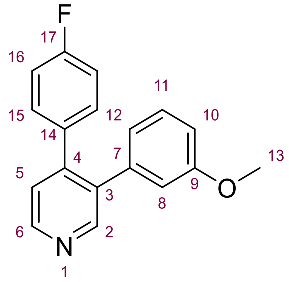

The title compound was prepared according to General Procedure C using 2-methylphenylboronic acid (167 mg, 1.10 mmol) and purified by FCC (10%-30% EtOAc in pentane) to give *pyridine* **3s** (402 mg, 96% yield) as a pale yellow oil.

HRMS (ESI): Exact mass calculated for $C_{18}H_{15}FNO$ $[M+H]^+$: 280.1132, found: 280.1130.

^{1}H NMR (400 MHz, $CDCl_3$) δ 8.65 (d, *J* = 0.7 Hz, 1H, C^2**H**), 8.62 (d, *J* = 5.1 Hz, 1H, C^5**H**), 7.31 (dd, *J* = 5.1, 0.7 Hz, 1H, C^6**H**), 7.20 (dd, *J* = 8.4, 7.5 Hz, 1H, C^{11}**H**), 7.17 – 7.11 (m, 2H, 2 x C^{15}**H**), 7.02 – 6.92 (m, 2H, 2 x C^{16}**H**), 6.83 (ddd, *J* = 8.3, 2.6, 1.0 Hz, 1H, C^{12}**H**), 6.72 (ddd, *J* = 7.6, 1.6, 0.9 Hz, 1H, C^{10}**H**), 6.67 (dd, *J* = 2.6, 1.6 Hz, 1H, C^8**H**), 3.68 (s, 3H, C^{13}**H₃**).

^{13}C NMR (101 MHz, $CDCl_3$) δ 162.6 (d, *J* = 248.2 Hz, C^{17}), 159.6 (C^9), 151.1 (C^2H), 148.9 (C^5H), 146.8 (C^4), 139.0 (C), 135.8 (C), 134.7 (d, *J* = 3.5 Hz, C^{14}), 131.1 (d, *J* = 8.3 Hz, 2 x C^{15}H), 129.6 (C^{11}H), 124.6 (C^6H), 122.4 (C^{10}H), 115.5 (d, *J* = 21.5 Hz, 2 x C^{16}H), 115.5 (C^8H), 113.4 (C^{12}H), 55.3 ($C^{13}H_3$).

^{19}F NMR (377 MHz, $CDCl_3$) δ -113.8 (tt, *J* = 8.5, 5.2 Hz).

IR (neat) (cm^{-1}): 3039, 2938, 1605, 1512, 1472, 1221, 1160, 1046, 1016, 828.

3-Butyl-4-(4-fluorophenyl)pyridine (3u)

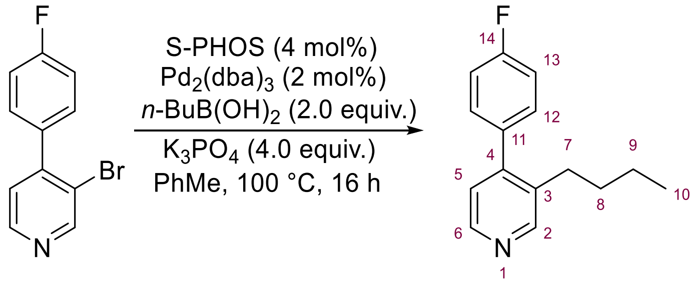

3-Bromo-4-(4-fluorophenyl)pyridine **5S** (1.00 equiv.), S-PHOS (4 mol%), potassium phosphate (4.00 equiv.), and alkylboronic acid (2.00 equiv.) were dissolved in PhMe (8 mL per mmol of aryl bromide) which was then purged with Ar for 10 minutes. $Pd_2(dba)_3$ (2 mol%) was added and the solution heated for 16 hours at 100 °C. The solution was cooled, diluted with EtOAC and extracted three times with EtOAC. The combined organic layers were washed with brine, dried ($MgSO_4$), filtered and concentrated *in vacuo*. The crude material was carried through without further purification.

4-(Benzofuran-5-yl)pyridine (3v)

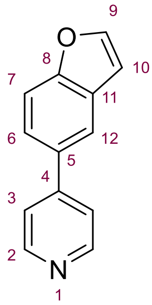

4-Pyridylboronic acid (615 mg, 5.00 mmol), 5-bromobenzofuran (0.75 mL, 6.00 mmol), potassium carbonate (1.80 g, 13.5 mmol), and triphenylphosphine (131 mg, 10 mol%) were dissolved in dioxane:water (25 mL:10 mL). The solution was purged with argon for 10 minutes, then palladium acetate (28 mg, 2.5 mol%) was added. The reaction was heated at 80 °C under argon for 14 hours, then cooled, and diluted with EtOAc (50 mL) and water (50 mL). The solution was separated, and the aqueous layer was extracted with EtOAc (2 x 50 mL, the organic layers were combined, washed with brine, dried ($MgSO_4$), filtered, and concentrated *in vacuo*. The crude material was purified by FCC (50% EtOAc in Pentane) to yield *heterocycle* **3v** (590 mg, 61% yield) as a white solid. The spectroscopic data was consistent with previous literature reports.[12]

1-Methyl-7-(pyridin-4-yl)-1H-indole (3w)

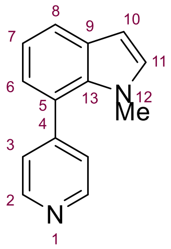

4-Pyridylboronic acid (830 mg, 6.75 mmol), 7-bromo-1-methyl-1H-indole (945 mg, 4.50 mmol), potassium carbonate (1.55 g, 11.25 mmol), and triphenylphosphine (118 mg, 10 mol%) were dissolved in dioxane:water (40 mL:10 mL). The solution was purged with argon for 10 minutes, then palladium acetate (25 mg, 2.5 mol%) was added. The reaction was heated at 80 °C under argon for 14 hours, then cooled, and diluted with EtOAc (50 mL) and water (50 mL). The solution was separated, and the aqueous layer was extracted with EtOAc (2 x 50 mL, the organic layers were combined, washed with brine, dried ($MgSO_4$), filtered, and concentrated *in vacuo*. The crude material was purified by FCC (20%-50% EtOAc in Pentane) to yield *heterocycle* **3w** (500 mg, 53% yield) as a white solid.

m.p. (EtOAc) 130 - 132 °C;

HRMS (ESI): Exact mass calculated for $C_{14}H_{13}N_2$ $[M+H]^+$: 209.1073, found: 209.1074.

^{1}H NMR (400 MHz, $CDCl_3$) δ 8.72 – 8.64 (m, 2H, 2 x C^2**H**), 7.68 (dd, *J* = 7.9, 1.2 Hz, 1H, C^6**H**), 7.43 – 7.36 (m, 2H, 2 x C^3**H**), 7.15 (dd, *J* = 7.9, 7.2 Hz, 1H, C^7**H**), 7.05 – 6.98 (m, 2H, C^8**H** + C^{11}**H**), 6.58 (d, *J* = 3.1 Hz, 1H, C^{10}**H**), 3.36 (s, 3H, C^{12}**H$_3$**).

^{13}C NMR (101 MHz, $CDCl_3$) δ 149.3 (2 x C^{2}H), 148.9 (C), 133.4 (C), 131.4 (C^{11}H), 130.2 (C), 125.3 (2 x C^{3}H), 124.1 (C^{8}H), 123.9 (C), 121.5 (C^{6}H), 119.3 (C^{7}H), 101.7 (C^{10}H), 37.2 (C^{12}H$_3$).

IR (neat) (cm^{-1}): 3012, 2160, 2031, 1978, 1511, 1470, 1229, 831, 822, 792.

[12]M. Hagiwara, A. Kobayashi, T. Hosoya, S. Yoshida, and Y. Sumida (Kyoto University), Composition for Activating Neurogenesis, 2019, EP 3508223 A1

9-Ethyl-3-(pyridin-4-yl)-9H-carbazole (3x)

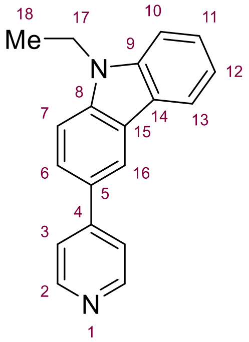

4-Pyridylboronic acid (1.23 g, 10.0 mmol), 3-bromo-9-ethyl-9H-carbazole[13] (3.28 g, 12.0 mmol), potassium carbonate (3.70 g, 27.0 mmol), and triphenylphosphine (262 mg, 10 mol%) were dissolved in dioxane:water (80 mL:20 mL). The solution was purged with argon for 10 minutes, then palladium acetate (56 mg, 2.5 mol%) was added. The reaction was heated at 80 °C under argon for 14 hours, then cooled, and diluted with EtOAc (100 mL) and water (100 mL). The solution was separated, and the aqueous layer was extracted with EtOAc (2 x 50 mL, the organic layers were combined, washed with brine, dried ($MgSO_4$), filtered, and concentrated *in vacuo*. The crude material was purified by FCC (10%-40% EtOAc in Pentane) to yield *heterocycle* **3x** (1.00 g, 30% yield) as a white solid.

m.p. (EtOAc) 161 - 163 °C

HRMS (ESI): Exact mass calculated for $C_{19}H_{17}N_2$ $[M+H]^+$: 273.1386, found: 273.1386.

^{1}H NMR (400 MHz, $CDCl_3$) δ 8.71 – 8.64 (m, 2H, 2 x C^2**H**), 8.40 (d, *J* = 1.8 Hz, 1H, C^{16}**H**), 8.17 (dt, *J* = 7.7, 1.0 Hz, 1H, C^{13}**H**), 7.77 (dd, *J* = 8.5, 1.8 Hz, 1H, C^6**H**), 7.67 – 7.59 (m, 2H, 2 x C^3**H**), 7.55 – 7.41 (m, 3H, C^7**H** + C^{10}**H** + C^{11}**H**), 7.29 (ddd, *J* = 7.9, 7.0, 1.1 Hz, 1H, C^{12}**H**), 4.41 (q, *J* = 7.2 Hz, 2H, C^{17}**H$_2$**), 1.47 (t, *J* = 7.2 Hz, 3H, C^{18}**H$_3$**).

^{13}C NMR (101 MHz, $CDCl_3$) δ 150.3 (C^2H), 149.3 (C^4), 140.6 (C), 140.5 (C), 128.9 (C), 126.4 (C^{11}H), 124.8 (C^6H), 123.8 (C), 123.0 (C), 121.7 (2 x C^3H), 120.7 (C^{13}H), 119.5 (C^{12}H), 119.2 (C^{16}H), 109.1 (C^7H/C^{10}H), 108.9 (C^7H/C^{10}H), 37.9 ($C^{17}H_2$), 14.0 ($C^{18}H_3$).

IR (neat) (cm^{-1}): 2983, 2161, 1594, 1472, 1459, 1232, 1217, 804, 757, 735.

Ethyl α-carbethoxy-β-*m*-bromoanilinoacrylate (S6)

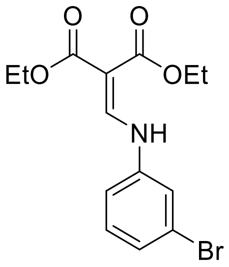

3-Bromoaniline (10 g, 58.1 mmol) and diethyl methoxymethylenemalonate (14 mL, 63.9 mmol) were heated together at 110 °C for 1 hour. The solution was cooled to room temperature which formed a solid, which was broken up and suspended in pentane. The suspension was filtered, washed with pentane, and dried under vacuum to yield *acrylate* **S6** (19.2 g, 97% yield) as a white solid. Spectroscopic data matched those previously reported.[14]

---

[13] L. Przypis Krzysztof and Z. Walczak, *J. Org. Chem.*, 2019, **84**, 2287–2296

[14] D. De, F. M. Krogstad, L. D. Byers, D. J. Krogstad, *J. Med.l Chem.*, 1998, **41**, 4918 – 4926.

7-Bromo-4-oxoquinoline-3-carboxylic acid ethyl ester (S7)

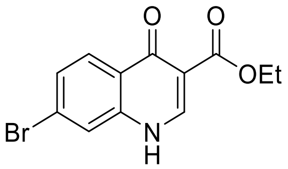

Dowtherm A© (50 mL) was heated to 250 °C and Compound **S6** (10.0 g, 29.0 mmol) was added portionwise. The solution was heated at 250 °C for 1 hour before being cooled slowly to room temperature. The resulting suspension was filtered, washed with pentane and dried under vacuum to yield *heterocycle* **S7** (6.99 g, 79% yield) as a colourless solid. Literature NMR spectra in d^6-DMSO are available. However, in our hands, **S7** was insoluble in DMSO and all other deuterated solvents, no NMR spectra could be obtained and the compound was taken onto to the next step.

***N*-Ethyl-7-bromo-4-oxoquinoline-3-carboxylic acid ethyl ester (S8)**

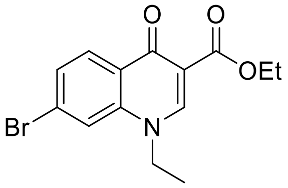

S7 (2.95 g, 10.0 mmol) and potassium carbonate (4.14 g, 30.0 mmol) were suspended in DMF (30 mL) and ethyl iodide (4 mL, 50 mmol) was added. The solution was heated at 80 °C for 5 hours, then poured into water (100 mL). The resulting suspension was filtered and solid was dissolved in CH_2Cl_2 (50 mL), dried ($MgSO_4$), filtered, and concentrated *in vacuo*. The crude material was purified by FCC (50-100% EtOAc in pentane) to give *heterocycle* **S8** (2.37 g, 73% yield) as a brown solid. Spectroscopic data matched those previously reported.[15]

***N*-Ethyl-7-(4-pyridine)-4-oxoquinoline-3-carboxylic acid ethyl ester (3y)**

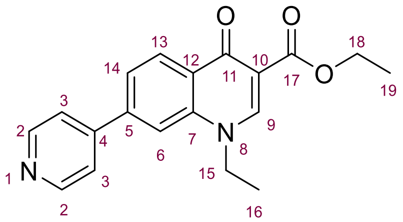

4-Pyridylboronic acid (553 mg, 4.50 mmol), **S8** (969 mg, 3.00 mmol), potassium carbonate (1.04 g, 7.5 mmol), and triphenylphosphine (79 mg, 10 mol%) were dissolved in dioxane:water (25 mL:10 mL). The solution was purged with argon for 10 minutes, then palladium acetate (17 mg, 2.5 mol%) was added. The reaction was heated at 80 °C under argon for 14 hours, then cooled, and diluted with EtOAc (50 mL) and water (50 mL). The solution was separated, and the aqueous layer was extracted with EtOAc (2 x 50 mL, the organic layers were combined, washed with brine, dried ($MgSO_4$), filtered, and concentrated *in vacuo*. The crude material was purified by FCC (0-4% MeOH in CH_2Cl_2) to yield *heterocycle* **3y** (572 mg, 59% yield) as a cream solid. Spectroscopic data matched those previously reported.[9]

---

[15] A. L. Choy, A. Eakin, O. Quiroga, B. Sherer (AstraZeneca AB), Heterocyclic Urea Derivatives and Methods of Use Thereof, 2010, US2010317624 A1.

The Synthesis of Pyridinium Salts

General Procedure D: Preparation of benzyl pyridinium iodide salts.

A mixture of the corresponding pyridine (1.00 equiv.) and benzyl iodide (1.50/2.00 equiv.) in acetone (0.5 M) was stirred in the dark at room temperature for 16 hours. Diethyl ether (10 mL) was added and the resulting suspension was sonicated (15 min) and then filtered. The resultant solid was washed with diethyl ether and dried under vacuum to give the benzyl pyridinium iodide salts as crystalline solids.

***N*-Benzyl-4-phenylpyridinium iodide (S9)**

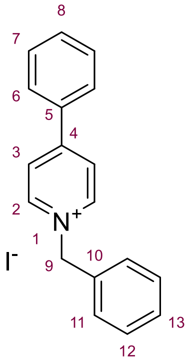

The title compound was prepared by General Procedure D using 4-phenyl pyridine (1.41 g, 9.10 mmol) and benzyl iodide (4.78 g 18.2 mmol) to give salt **S9** (3.46 g, 91%) as a yellow solid. Spectroscopic data was consistent with that reported in the literature.[16]

***N*-(2-Nitrobenzyl)-4-phenylpyridinium iodide (1b)**

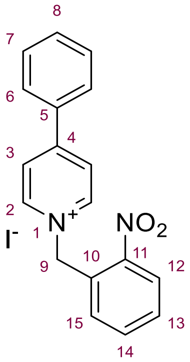

The title compound was prepared by General Procedure D using 4-phenyl pyridine (1.41 g, 9.1 mmol) and 2-nitrobenzyl iodide **S1** (4.78 g 18.2 mmol) to give salt **1b** (3.46 g, 91%) as a yellow solid.

m.p. (acetone) 196-198 °C;

HRMS (ESI): Exact mass calculated for $C_{18}H_{15}O_2N_2$ $[M]^+$: 291.11280, found: 291.11275;

^{1}H NMR (400 MHz, DMSO) δ 9.18 – 9.08 (d, *J*=7.1 Hz, 2H, 2 x C^2**H**), 8.68 – 8.57 (d, *J*=7.1 Hz, 2H, 2 x C^3**H**), 8.28 (dd, *J* = 8.1, 1.4 Hz, 1H, C^{12}**H**), 8.18 – 8.08 (m, 2H, 2 x ArC**H**), 7.86 – 7.62 (m, 5H, 5 x ArC**H**), 7.19 (dd, *J* = 7.7, 1.4 Hz, 1H, C^{15}**H**), 6.24 (s, 2H, C^9**H$_2$**).

^{13}C NMR (101 MHz, DMSO) δ 155.5 (ArC), 147.5 (ArC), 145.6 (2 x C^{2}H), 135.0 (ArCH), 133.5 (ArC), 132.4 (ArCH), 130.4 (ArCH), 130.0 (C^{15}H), 129.8 (2 x ArCH), 129.6 (ArC), 128.3 (2 x ArCH), 125.6 (C^{12}H), 124.9 (2 x C^{3}H), 59.9 (C^{9}H$_2$).

IR (neat) (cm^{-1}): 1637, 1514, 1440, 1347, 1335, 1200, 861, 790, 772, 747.

[16] A. Grozavu, H. B. Hepburn, P. J. Smith, H. K. Potukuchi, P. J. Lindsay-Scott and T. J. Donohoe, *Nat. Chem.*, 2019, **11**, 242-247.

***N*-(2,4-Dinitrobenzyl)-4-phenylpyridinium iodide (S10)**

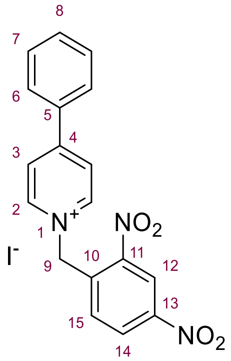

Title compound was prepared by General Procedure D using 4-phenyl pyridine (65.7 mg, 0.42 mmol) and 2,4-bisnitrobenzyl iodide **S2** (261 mg, 0.85 mmol) to give salt **S10** (185 mg, 95%) as a red-brown solid. This compound was found to be highly hydroscopic and water in the atmosphere readily cleaved the bond between N^1 and C^9. Due to this instability full characterisation data was not obtained.

HRMS (ESI): Exact mass calculated for $C_{18}H_{14}O_2N_3[M]^+$ m/z: 336.09788, found 336.09787

^{1}H NMR (400 MHz, DMSO) δ 9.13 (d, *J* = 6.9 Hz, 2H, 2 x C^2**H**), 8.91 (d, *J* = 2.4 Hz, 1H, C^{12}**H**), 8.68 (d, *J* = 7.1 Hz, 2H, 2 x C^3**H**), 8.51 (dd, *J* = 8.6, 2.4 Hz, 1H, C^{14}**H**), 8.18 – 8.10 (m, 2H, 2 x C^7**H**), 7.76 – 7.62 (m, 3H, 2 x C^6**H** + C^8**H**), 7.37 (d, *J* = 8.6 Hz, 1H, C^{15}**H**), 6.34 (s, 2H, C^9**H₂**).

***N*-(2,4-bis(Trifluoromethyl)benzyl)-4-phenylpyridinium iodide (1c)**

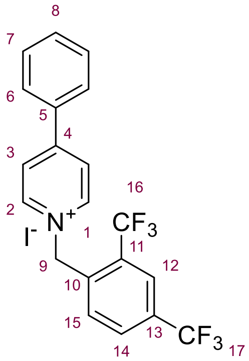

The title compound was prepared by General Procedure D using 4-phenyl pyridine (517 mg, 3.33 mmol) and 2,4-bis(trifluoromethyl)benzyl iodide **S4** (2.37 g, 6.66 mmol) to give the pyridinium **1c** (1.51 g, 89%) as a yellow solid.

m.p. (acetone): 221-223 °C

HRMS (ESI): Exact mass calculated for $C_{20}H_{14}F_6N$ $[M]^+$ m/z: 382.10250, found: 382.10225

^{1}H NMR (400 MHz, DMSO-d_6) δ 9.13 (d, *J* = 6.9 Hz, 2H, 2 x C^2**H**), 8.63 (d, *J* = 7.1 Hz, 2H, 2 x C^3**H**), 8.22 (s, 1H, C^{12}**H**), 8.13 (m, 3H, 2 x C^7**H** + C^{14}**H**), 7.72 – 7.64 (m, 3H, 2 x C^6**H** + C^8**H**), 7.43 (d, *J* = 8.2 Hz, 1H, C^{15}**H**), 6.21 (s, 2H, 2 x C^9**H₂**).

^{13}C NMR (126 MHz, DMSO-d_6) δ 155.9 (C), 145.8 (2 x C^2H), 136.8 (C), 133.3 (C), 132.6 (C^8H), 131.0 (C^{15}H), 130.5 (q, *J* = 3.9 Hz, C^{14}H), 129.9 (q, *J* = 33.5 Hz, C^{11}/C^{13}), 129.8 (2 x C^6H), 128.4 (2 x C^7H), 127.8 (q, *J* = 31.7 Hz, C^{11}/C^{13}), 124.9 (2 x C^3H), 124.0 – 123.5 (m, C^{12}H), 123.22 (q, *J* = 274.6 Hz, $C^{16}F_3$/$C^{17}F_3$), 123.18 (q, *J* = 272.6 Hz, $C^{16}F_3$/$C^{17}F_3$), 58.9 (q, *J* = 3.1 Hz, C^9H_2).

^{19}F NMR (377 MHz, DMSO-d_6) δ –59.0, –61.5.

IR (neat) (cm^{-1}):2984, 2161, 1978, 1639, 1343, 1284, 1270, 1181, 1168, 1137.

N-(3,5-bis(Trifluoromethyl)benzyl)-4-phenylpyridinium iodide (1d)

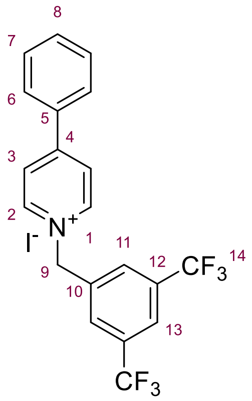

Title compound was prepared by General Procedure D using 4-phenyl pyridine (155 mg, 1.00 mmol) and 3,5-bis(trifluoromethy)benzyl iodide **S3** (708.1 mg, 2.00 mmol) to give the pyridinium **1d** (435 mg, 88%) as a yellow solid.

m.p. (acetone): >350 °C

HRMS (ESI): Exact mass calculated for $C_{20}H_{14}F_6N$ $[M]^+$: 382.1025, found 382.1031

^{1}**H NMR** (400 MHz, DMSO) δ 9.30 (d, *J* = 7.1 Hz, 2H, 2 x C^{2}**H**), 8.57 (d, *J* = 7.1 Hz, 2H, 2 x C^{3}**H**), 8.4 (d, *J* = 1.7 Hz, 2H, 2 x C^{11}**H**), 8.23 (s, 1H, C^{13}**H**), 8.12 – 8.06(m, 2H, 2 x ArC**H**), 7.74 – 7.49 (m, 3H, 3 x ArC**H**), 5.99 (s, 2H, C^{9}**H$_2$**).

^{13}**C NMR** (126 MHz, DMSO) δ 155.3 (C^{4}), 145.0 (2 x C^{2}H), 137.0 (C), 133.5 (C), 132.3 (ArCH), 130.9 (q, *J* = 33.0 Hz, 2 x C^{12}), 130.6 (q, *J* = 3.8 Hz, 2 x C^{11}H), 129.7 (2 x ArCH), 128.3 (2 x ArCH), 125.0 (2 x C^{3}H), 123.7 – 123.2 (m, C^{13}H), 123.1 (q, *J* = 273.1 Hz, 2 x C^{14}F$_3$), 61.0 (C^{9}H$_2$).

^{19}**F NMR** (377 MHz, DMSO) δ –61.2.

IR (neat) (cm^{-1}): 3021, 2960, 1635, 1280, 1167, 1113, 910, 771, 744, 683.

N-(2,4-bis(Trifluoromethyl)benzyl)-4-(4-(*tert*-butyl)phenyl)pyridinium iodide (1e)

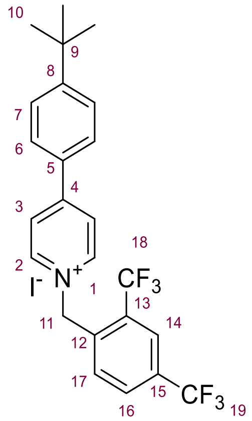

The title compound was prepared by General Procedure D using 4-(4-(*tert*-butyl)phenyl)pyridine **3e** (423 mg, 2.0 mmol) and 2,4-bis(trifluoromethyl)benzyl iodide **S4** (1.06 g, 3.00 mmol) to give the pyridinium **1e** (1.11 g, 98%) as a yellow solid.

m.p. (acetone): 255-257 °C

HRMS (ESI): Exact mass calculated for $C_{24}H_{22}NF_6$ $[M]^+$: 438.16510, found 438.16490

^{1}**H NMR** (400 MHz, DMSO-*d*$_6$) δ 9.10 (d, *J* = 7.0 Hz, 2H, 2 x C^{2}**H**), 8.61 (d, *J* = 7.0 Hz, 2H, 2 x C^{3}**H**), 8.23 (s, 1H, C^{14}**H**), 8.15 (d, *J* = 8.4 Hz, 1H, C^{16}**H**), 8.09 (d, *J* = 8.6 Hz, 2H, 2 x C^{6}**H**), 7.69 (d, *J* = 8.6 Hz, 2H, 2 x C^{7}**H**), 7.44 (d, *J* = 8.2 Hz, 1H, C^{17}**H**), 6.20 (s, 2H, 2 x C^{11}**H**), 1.36 (s, 9H, 3 x C^{10}**H$_3$**).

^{13}**C NMR** (126 MHz, DMSO-*d*$_6$) δ 155.9 (C), 155.7 (C), 145.7 (2 x C^{3}H), 136.8 (C^{12}), 131.1 (C^{17}H), 130.6 – 130.4 (m, C^{16}H+C), 129.95 (q, *J* = 33.4 Hz, C^{13}/C^{15}), 128.3 (2 x C^{6}H), 127.8 (q, *J* = 31.7 Hz, C^{13}/C^{15}), 126.7

(2 x $C^{7}H$), 124.5 (2 x $C^{2}H$), 124.0 – 123.7 (m, $C^{14}H$), 123.18 (q, J = 274.7 Hz, $C^{18}F_3/C^{19}F_3$), 123.15 (q, J = 272.7 Hz, $C^{18}F_3/C^{19}F_3$), 58.8 (q, J = 3.4 Hz, $C^{11}H_2$), 34.9 (C^{9}), 30.8 (3 x $C^{10}H_3$).
^{19}F NMR (377 MHz, DMSO-d_6) δ –59.0, –61.5.
IR (neat) (cm^{-1}): 3659, 2981, 1341, 1270, 1182, 1166, 1131, 1084, 1052, 826.

***N*-(2,4-bis(Trifluoromethyl)benzyl)-4-(4-methoxyphenyl)pyridinium iodide (1f)**

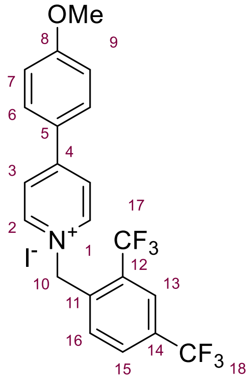

The title compound was prepared by General Procedure D using 4-(4-methoxyphenyl)pyridine **3f** (460 mg, 2.50 mmol) and 2,4-bis(trifluoromethyl)benzyl iodide **S4** (1.46 g, 4.20 mmol) to give the pyridinium **1f** (1.23 g, 92%) as a yellow solid.
m.p. (acetone): 215-217 °C
HRMS (ESI): Exact mass calculated for $C_{21}H_{16}ONF_6$ $[M]^+$: 412.1130, found: 412.1131
^{1}H NMR (400 MHz, DMSO) δ 9.02 (d, J = 7.1 Hz, 2H, 2 x C^{2}**H**), 8.57 (d, J = 7.2 Hz, 2H, 2 x C^{3}**H**), 8.22 (s, 1H, C^{13}**H**), 8.18 (d, J = 9.0 Hz, 2H, 2 x C^{6}**H**), 8.12 (dd, J = 8.2, 2.2 Hz, 1H, C^{15}**H**), 7.38 (d, J = 8.2 Hz, 1H, C^{16}**H**), 7.22 (d, J = 9.0 Hz, 2H, 2 x C^{7}**H**), 6.15 (s, 2H, C^{10}**H_2**), 3.90 (s, 3H, C^{9}**H_3**).
^{13}C NMR (126 MHz, DMSO) δ 163.1 (C^{8}), 155.1 (C^{4}), 145.4 (2 x $C^{2}H$), 137.1 (C^{11}), 130.7 ($C^{16}H$), 130.5 (q, J = 3.9 Hz, $C^{15}H$), 130.4 (2 x $C^{6}H$), 129.8 (q, J = 33.3 Hz, C^{12}/C^{14}), 127.7 (q, J = 31.7 Hz, C^{12}/C^{14}), 125.1 (C^{5}), 124.0 – 123.6 (m, $C^{13}H$), 123.5 (2 x $C^{3}H$), 123.2 (q, J = 274.6 Hz, $C^{17}F_3/C^{18}F_3$), 123.1 (q, J = 272.4 Hz, $C^{17}F_3/C^{18}F_3$), 115.3 (2 x $C^{7}H$), 58.6 (q, J = 3.3 Hz, $C^{10}H_2$), 55.8 ($C^{9}H_3$).
^{19}F NMR (377 MHz, DMSO) δ –59.1, –61.4.
IR (neat) (cm^{-1}): 2981, 1640, 1599, 1344, 1271, 1166, 1122, 1084, 1053, 833.

***N*-(2,4-bis(Trifluoromethyl)benzyl)-4-(4-(trifluoromethyl) phenyl)pyridinium iodide (1g)**

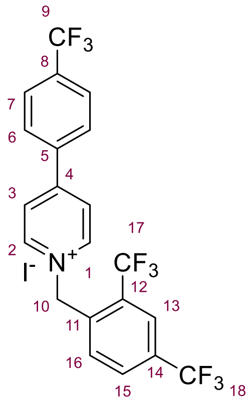

The title compound was prepared by General Procedure D using 4-(4-(trifluoromethyl) phenyl)pyridine **3g** (446 mg, 2.00 mmol) and 2,4-bis(trifluoromethyl)benzyl iodide **S4** (1.06 g, 3.00 mmol) to give the pyridinium **1g** (987 mg, 85%) as a yellow solid.
m.p. (acetone): 226-228 °C
HRMS (ESI): Exact mass calculated for $C_{21}H_{13}NF_9$ $[M]^+$: 450.08900, found 450.08860

^{1}H NMR (400 MHz, DMSO-d_6) δ 9.22 (d, *J* = 7.0 Hz, 2H, 2 x C^2**H**), 8.69 (d, *J* = 7.1 Hz, 2H, 2 x C^3**H**), 8.32 (d, *J* = 8.2 Hz, 2H, 2 x C^6**H**), 8.23 (s, 1H, C^{13}**H**), 8.14 (d, *J* = 8.5 Hz, 1H, C^{15}**H**), 8.05 (d, *J* = 8.3 Hz, 2H, 2 x C^7**H**), 7.45 (d, *J* = 8.2 Hz, 1H, C^{16}**H**), 6.25 (s, 2H, 2 x C^{10}**H$_2$**).
^{13}C NMR (126 MHz, DMSO-d_6) δ 154.5 (C), 146.2 (2 x C^{3}H), 137.5 (C), 136.7 (C), 131.8 (q, *J* = 32.4 Hz, C^8/C^{12}/C^{14}), 131.1 (C^{16}H), 130.5 (d, *J* = 3.8 Hz, C^{15}H), 130.0 (q, *J* = 33.3 Hz, C^8/C^{12}/C^{14}), 129.4 (2 x C^{6}H), 127.9 (q, *J* = 31.7 Hz, 2 x C^{7}H), 126.5 (q, *J* = 3.8 Hz, C^8/C^{12}/C^{14}), 125.9 (2 x C^{2}H), 124.0 – 123.7 (m, C^{13}H), 123.17 (q, *J* = 274.6 Hz, C^{9}F$_3$), 123.15 (q, *J* = 272.4 Hz, 2 x C^{17}F$_3$/C^{18}F$_3$), 59.2 (q, *J* = 3.4 Hz, C^{10}H$_2$).
^{19}F NMR (376 MHz, DMSO-d_6) δ –59.0, –61.5.
IR (neat) (cm^{-1}): 3660, 2981, 1325, 1276, 1167, 1127, 1109, 1084, 1072, 1057.

***N*-(2,4-bis(Trifluoromethyl)benzyl)-4-(4-(methoxycarbonyl) phenyl)pyridinium iodide (1h)**

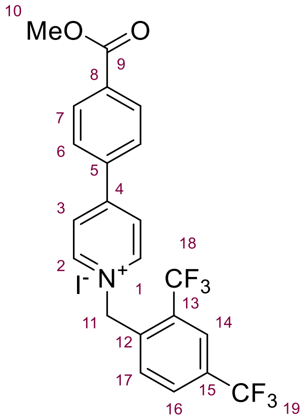

The title compound was prepared by General Procedure D using 4-(4-(methoxycarbonyl) phenyl)pyridine **3h** (426 mg, 2.00 mmol) and 2,4-bis(trifluoromethyl)benzyl iodide **S4** (1.06 g, 3.00 mmol) to give the pyridinium **1h** (1.01 g, 89%) as a yellow solid.
m.p. (acetone): 236-238 °C
HRMS (ESI): Exact mass calculated for $C_{22}H_{16}O_2NF_6$ [M]$^+$: 440.10797, found 440.10834
^{1}H NMR (400 MHz, DMSO-d_6) δ 9.19 (d, *J* = 7.0 Hz, 2H, 2 x C^2**H**), 8.68 (d, *J* = 7.0 Hz, 2H, 2 x C^3**H**), 8.26 (d, *J* = 8.8 Hz, 2H, 2 x C^6**H**), 8.22 (s, 1H, C^{14}**H**), 8.20 (d, *J* = 8.7 Hz, 2H, 2 x C^7**H**), 8.13 (d, *J* = 8.4 Hz, 1H, C^{16}**H**), 7.44 (d, *J* = 8.2 Hz, 1H, C^{17}**H**), 6.24 (s, 2H, 2 x C^{11}**H$_2$**), 3.92 (s, 3H, 3 x C^{10}**H$_3$**).
^{13}C NMR (126 MHz, DMSO) δ 165.6 (C^{9}O), 154.7(C), 146.2 (2 x C^{2}H), 137.7 (C), 136.7 (C), 132.6 (C), 131.0 (C^{17}H), 130.5 (d, *J* = 3.8 Hz, C^{16}H), 130.3 (2 x C^{6}H), 130.0 (q, *J* = 33.3 Hz, C^{13}/C^{15}), 128.9 (2 x C^{7}H), 127.9 (q, *J* = 31.7 Hz, C^{13}/C^{15}), 125.7 (2 x C^{3}H), 124.1 – 123.6 (m, C^{14}H), 123.21 (q, *J* = 274.6 Hz, C^{18}F$_3$/C^{19}F$_3$), 123.17 (q, *J* = 272.7 Hz, C^{18}F$_3$/C^{19}F$_3$), 59.2 (q, *J* = 3.3 Hz, C^{11}H$_2$), 52.7 (C^{10}H$_3$).
^{19}F NMR (377 MHz, DMSO) δ –59.00, –61.46.
IR (neat) (cm^{-1}): 3660, 2981, 1719, 1348, 1277, 1179, 1137, 1118, 1084, 1055.

***N*-(2,4-bis(Trifluoromethyl)benzyl)-4-(4-(methylsulfonyl) phenyl)pyridinium iodide (1i)**

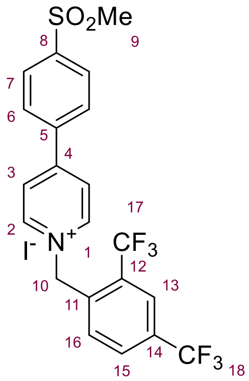

The title compound was prepared by General Procedure D using 4-(4-(methylsulfonyl) phenyl)pyridine **3i** (466 mg, 2.00 mmol) and 2,4-bis(trifluoromethyl)benzyl iodide **S4** (1.06 g, 3.00 mmol) to give the pyridinium **1i** (1.13 g, 96%) as a yellow solid.

m.p. (acetone): 247-249 °C

HRMS (ESI): Exact mass calculated for $C_{21}H_{16}O_2NF_6{}^{32}S$ $[M]^+$: 460.08005, found 460.08010

^{1}H NMR (400 MHz, DMSO-d_6) δ 9.23 (d, J = 6.7 Hz, 2H, 2 x C^2**H**), 8.70 (d, J = 7.1 Hz, 2H, 2 x C^3**H**), 8.36 (d, J = 8.6 Hz, 2H, 2 x C^6**H**), 8.23 (s, 1H, C^{13}**H**), 8.20 (d, J = 8.6 Hz, 2H, 2 x C^7**H**), 8.14 (d, J = 7.5 Hz, 1H, C^{15}**H**), 7.47 (d, J = 8.2 Hz, 1H, C^{16}**H**), 6.26 (s, 2H, 2 x C^{10}**H$_2$**), 3.35 (s, 3H, C^9**H$_3$)**.

^{13}C NMR (126 MHz, DMSO-d_6) δ 154.3 (C), 146.2 (2 x C^{2}H), 143.6 (C), 138.2 (C), 136.6 (C), 131.1 (C^{16}H), 130.5 (d, J = 3.7 Hz, C^{15}H), 130.0 (q, J = 33.3 Hz, C^{12}/C^{14}), 129.5 (2 x C^{6}H), 128.1 (2 x C^{7}H), 127.9 (q, J = 31.7 Hz, C^{12}/C^{14}), 126.0 (2 x C^{3}H), 124.1 – 123.6 (m, C^{13}H), 123.18 (q, J = 274.5 Hz, 2 x C^{17}F$_3$/C^{18}F$_3$), 123.15 (q, J = 272.7 Hz, 2 x C^{17}F$_3$/C^{18}F$_3$), 59.2 (d, J = 3.3 Hz, C^{10}H$_2$), 43.2 (C^{9}H$_3$).

^{19}F NMR (376 MHz, DMSO-d_6) δ–59.0, –61.5.

IR (neat) (cm^{-1}): 2981, 1703, 1639, 1345, 1274, 1172, 1123, 1085, 1054, 961.

***N*-(2,4-bis(Trifluoromethyl)benzyl)-4-(4-fluorophenyl)pyridinium iodide (1j)**

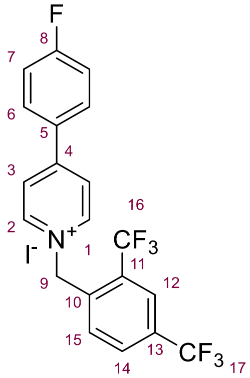

The title compound was prepared by General Procedure D using 4-(4-fluorophenyl)pyridine **3j** (345 mg, 2.00 mmol) and 2,4-bis(trifluoromethyl)benzyl iodide **S4** (1.06 g, 3.00 mmol) to give the pyridinium **1j** (1.00 g, 95%) as a yellow solid.

m.p. (acetone): 225-227 °C

HRMS (ESI): Exact mass calculated for $C_{20}H_{13}NF_7$ $[M]^+$: 400.09307, found 400.09342

^{1}H NMR (400 MHz, DMSO-d_6) δ 9.12 (d, J = 7.1 Hz, 2H, 2 x C^2**H**), 8.61 (d, J = 7.1 Hz, 2H, 2 x C^3**H**), 8.28 – 8.19 (m, 3H, 2 x C^6**H** and C^{12}**H**), 8.12 (dd, J = 8.3, 2.2 Hz, 1H, C^{15}**H**), 7.53 (t, J = 8.8 Hz, 2H, 2 x C^7**H**), 7.41 (d, J = 8.2 Hz, 1H, C^{14}**H**), 6.20 (s, 2H, 2 x C^9**H$_2$**).

^{13}C NMR (126 MHz, DMSO-d_6) δ 164.8 (d, J = 251.9 Hz, C^8), 154.8 (C^4), 145.9 (2 x C^{2}H), 136.8 (C^5), 131.2 (d, J = 9.3 Hz, 2 x C^{6}H), 131.0 (C^{15}H), 130.5 (q, J = 4.0 Hz, C^{14}H), 130.0 (q, J = 33.2 Hz, C^{11}/C^{13}), 129.9 (q, J = 2.9 Hz, C^{10}), 127.9 (q, J = 31.8 Hz, C^{11}/C^{13}), 124.9 (2 x C^{3}H), 123.9-123.8 (m, C^{12}H), 123.21 (q, J = 274.6 Hz, C^{16}F$_3$/C^{17}F$_3$), 123.17 (q, J = 272.7 Hz, C^{16}F$_3$/C^{17}F$_3$), 117.0 (d, J = 22.0 Hz, 2 x C^{7}H), 58.9 (q, J = 2.8 Hz, C^{9}H$_2$).

^{19}F NMR (377 MHz, DMSO-d_6) δ –59.0, –61.5, –107.2 (tt, J = 8.9, 5.2 Hz).

IR (neat) (cm^{-1}): 3009, 2939, 1636, 1497, 1343, 1284, 1265, 1225, 1168, 1136..

***N*-(2,4-bis(Trifluoromethyl)benzyl)-4-(4-(trifluoromethoxy) phenyl)pyridinium iodide (1k)**

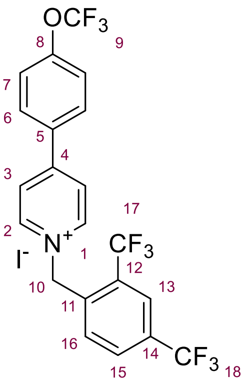

The title compound was prepared by General Procedure D using 4-(4-(trifluoromethoxy) phenyl)pyridine **3k** (482 mg, 2.00 mmol) and 2,4-bis(trifluoromethyl)benzyl iodide **S4** (1.06 g, 3.00 mmol) to give the pyridinium **1k** (1.07 g, 90%) as a yellow solid.

m.p. (acetone): 210-212 °C

HRMS (ESI): Exact mass calculated for $C_{21}H_{13}ONF_9$ $[M]^+$: 466.08479, found 466.08372

^{1}H NMR (400 MHz, DMSO-d_6) δ 9.17 (d, *J* = 6.4 Hz, 2H, 2 x C^2**H**), 8.64 (d, *J* = 6.9 Hz, 2H, 2 x C^3**H**), 8.27 (d, *J* = 8.9 Hz, 2H, 2 x C^6**H**), 8.22 (s, 1H, C^{13}**H**), 8.13 (d, *J* = 8.2 Hz, 1H, C^{15}**H**), 7.67 (d, *J* = 8.3 Hz, 2H, 2 x C^7**H**), 7.45 (d, *J* = 8.2 Hz, 1H, C^{16}**H**), 6.22 (s, 2H, 2 x C^{10}**H$_2$**).

^{13}C NMR (126 MHz, DMSO-d_6) δ 154.5 (C), 151.1 (C), 146.0 (2 x C^{2}H), 136.7 (C), 132.6 (C), 131.1 (C^{16}H), 130.9 (2 x C^{6}H), 130.5 (d, *J* = 3.8 Hz, C^{15}H), 130.0 (q, *J* = 33.3 Hz, C^{12}/C^{14}), 127.9 (q, *J* = 31.6 Hz, C^{12}/C^{14}), 125.3 (2 x C^{3}H), 124.0 – 123.7 (m, C^{13}H), 123.18 (q, *J* = 274.5 Hz, C^{17}F$_3$/C^{18}F$_3$), 123.15 (q, *J* = 272.7 Hz, C^{17}F$_3$/C^{18}F$_3$), 121.9 (2 x C^{7}H), 120.0 (q, *J* = 257.7 Hz, C^{9}F$_3$), 59.1 (q, *J* = 3.3 Hz, C^{10}H$_2$).

^{19}F NMR (376 MHz, DMSO-d_6) δ –56.6, –59.0, –61.5.

IR (neat) (cm^{-1}): 2981, 1638, 1342, 1269, 1212, 1166, 1123, 1084, 1052, 829.

***N*-(2,4-bis(Trifluoromethyl)benzyl)-4-(3,5-difluorophenyl)pyridinium iodide (1l)**

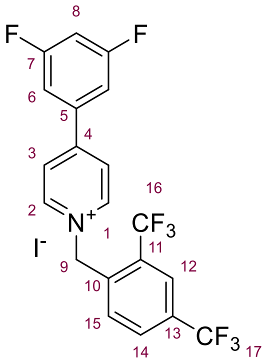

The title compound was prepared by General Procedure D using 4-(3,5-difluorophenyl)pyridine **3l** (382 mg, 2.00 mmol) and 2,4-bis(trifluoromethyl)benzyl iodide **S4** (1.06 g, 3.00 mmol) to give the pyridinium **1l** (714 mg, 65%) as a yellow solid.

m.p. (acetone): 233-235 °C

HRMS (ESI): Exact mass calculated for $C_{20}H_{12}NF_8$ $[M]^+$: 418.08365, found 418.08416

^{1}H NMR (400 MHz, DMSO-d_6) δ 9.22 (d, *J* = 6.9 Hz, 2H, 2 x C^2**H**), 8.69 (d, *J* = 7.1 Hz, 2H, 2 x C^3**H**), 8.23 (s, 1H, C^{12}**H**), 8.12 (d, *J* = 7.6 Hz, 1H, C^{14}**H**), 8.04 – 7.91 (m, 2H, 2 x C^6**H**), 7.64 (tt, *J* = 9.2, 2.3 Hz, 1H, C^8**H**), 7.40 (d, *J* = 8.2 Hz, 1H, C^{15}**H**), 6.22 (s, 2H, 2 x C^9**H**$_2$).

^{13}C NMR (126 MHz, DMSO-d_6) δ 163.1 (dd, *J* = 247.5, 12.4, Hz, 2 x C^7), 153.3 (C^4), 146.2 (2 x C^{2}H), 137.2 – 136.0 (m, C^5+ C^{10}), 130.9, (C^{15}H), 130.5 (C^{14}H), 130.0 (q, *J* = 33.2 Hz, C^{11}/C^{13}), 127.9 (q, *J* = 31.8 Hz,

C^{11}/C^{13}), 125.6 (2 x $C^{3}H$), 123.9 ($C^{12}H$), 123.17 (q, J = 275.0 Hz, $C^{16}F_3/C^{17}F_3$), 123.14 (q, J = 272.4 Hz, $C^{16}F_3/C^{17}F_3$), 112.3 – 111.8 (m, 2 x $C^{6}H$), 107.7 (t, J = 26.0 Hz, $C^{8}H$), 59.2 (q, J = 2.5 Hz, $C^{9}H_2$).
^{19}F NMR (377 MHz, DMSO-d_6) δ –59.0, –61.4, –107.7 (t, J = 8.5 Hz).
IR (neat) (cm^{-1}): 2981, 1339, 1275, 1177, 1117, 1084, 1054, 991, 839.

***N*-(2,4-bis(Trifluoromethyl)benzyl)-4-(3,4,5-trichlorophenyl)pyridinium iodide (1m)**

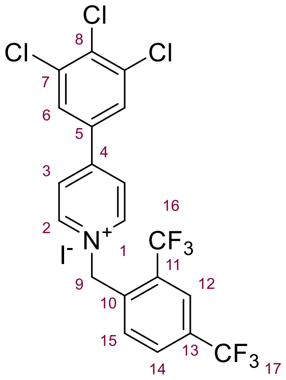

The title compound was prepared by General Procedure D using 4-(3,4,5-trichlorophenyl)pyridine **3m** (186 mg, 0.72 mmol) and 2,4-bis(trifluoromethyl)benzyl iodide **S4** (530 mg 1.50 mmol) to give the pyridinium **1m** (314 mg, 71%) as a yellow solid.
m.p. (acetone): 277-279 °C
HRMS (ESI): Exact mass calculated for $C_{20}H_{11}N^{35}Cl_3F_6$ $[M]^+$: 483.98558, found 483.98567
^{1}H NMR (400 MHz, DMSO-d_6) δ 9.22 (d, J = 7.0 Hz, 2H, 2 x C^{2}**H**), 8.74 (d, J = 7.1 Hz, 2H, 2 x C^{3}**H**), 8.47 (s, 2H, 2 x C^{6}**H**), 8.24 (s, 1H, C^{12}**H**), 8.12 (d, J = 8.2 Hz, 1H, C^{14}**H**), 7.36 (d, J = 8.2 Hz, 1H, C^{15}**H**), 6.22 (s, 2H, 2 x C^{9}**H**$_2$).
^{13}C NMR (126 MHz, DMSO-d_6) δ 152.4 (C^{4}), 146.2 (2 x $C^{2}H$), 136.7 (C), 134.4 (C), 134.2 (C), 133.8 (C), 130.8 ($C^{15}H$), 130.7 – 130.3 (m, $C^{14}H$), 130.0 (q, J = 33.2 Hz, C^{11}/C^{13}), 128.9 (2 x $C^{6}H$), 127.8 (q, J = 31.7 Hz, C^{11}/C^{13}), 125.7 (2 x $C^{3}H$), 124.01 – 123.63 (m, $C^{12}H$), 123.15 (q, J = 270.0 Hz, $C^{16}F_3$ + $C^{17}F_3$), 59.25 (s, $C^{9}H_2$).
^{19}F NMR (377 MHz, DMSO-d_6) δ –59.1, –61.4.
IR (neat) (cm^{-1}): 3659, 2981, 1639, 1381, 1347, 1288, 1274, 1186, 1136, 1117.

***N*-(2,4-bis(Trifluoromethyl)benzyl)-4-(o-tolyl)pyridinium iodide (1n)**

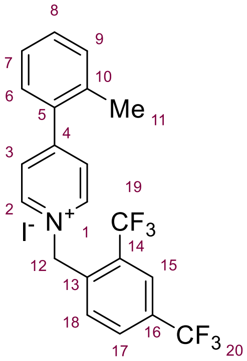

The title compound was prepared by General Procedure D using 4-(o-tolyl)pyridine **3n** (285 mg, 2.00 mmol) and 2,4-bis(trifluoromethyl)benzyl iodide **S4** (1.06 g, 3.00 mmol) to give the pyridinium **1n** (815 mg, 93%) as a yellow solid.
m.p. (acetone): 225-257 °C
HRMS (ESI): Exact mass calculated for $C_{21}H_{16}NF_6$ $[M]^+$: 396.11815, found 396.11719

^{1}H NMR (400 MHz, DMSO-d_6) δ 9.12 (d, *J* = 6.9 Hz, 2H, 2 x C^2**H**), 8.31 (d, *J* = 7.0 Hz, 2H, 2 x C^3**H**), 8.24 (s, 1H, C^{15}**H**), 8.17 (d, *J* = 8.2 Hz, 1H, C^{17}**H**), 7.55 – 7.40 (m, 5H, C^6**H**, C^7**H**, C^8**H**, C^9**H** and C^{18}**H**), 6.23 (s, 2H, 2 x C^{12}**H$_2$**), 2.39 (s, 3H, 3 x C^{11}**H$_3$**).
^{13}C NMR (126 MHz, DMSO-d_6) δ 158.5 (C^4), 145.3 (2 x C^{2}H), 136.5 (C), 135.6 (C), 135.4 (C), 131.5 (ArCH), 131.4 (ArCH), 130.7 (ArCH), 130.6 (C^{17}H), 130.1 (q, *J* = 33.2 Hz, C^{19}/C^{20}), 129.8 (ArCH), 128.5 (2 x C^{3}H), 128.0 (q, *J* = 31.7 Hz, C^{19}/C^{20}), 126.8 (ArCH), 123.9 (C^{15}H), 123.2 (q, *J* = 271.4 Hz, C^{19}F$_3$ + C^{20}F$_3$), 59.2 (s, C^{12}H$_2$), 19.9 (C^{11}H$_3$).
^{19}F NMR (376 MHz, DMSO-d_6) δ –58.9, –61.5.
IR (neat) (cm^{-1}): 2981, 1640, 1345, 1269, 1169, 1119, 1084, 1054, 762.

***N*-(2,4-bis(Trifluoromethyl)benzyl)-4-(3,5-dimethoxyphenyl) pyridinium iodide (1o)**

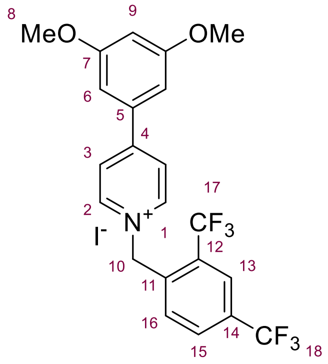

The title compound was prepared by General Procedure D using 4-(3,5-dimethoxyphenyl)pyridine **3o** (430 mg, 2.00 mmol) and 2,4-bis(trifluoromethyl)benzyl iodide **S4** (1.06 g, 3.00 mmol) to give the pyridinium **1o** (1.06 g, 93%) as a yellow solid.
m.p. (acetone): 235-237 °C
HRMS (ESI): Exact mass calculated for $C_{22}H_{18}O_2NF_6$ [M]$^+$: 442.12362, found 442.12302
^{1}H NMR (400 MHz, DMSO-d_6) δ 9.12 (d, *J* = 7.1 Hz, 2H, 2 x C^2**H**), 8.65 (d, *J* = 7.0 Hz, 2H, 2 x C^3**H**), 8.22 (s, 1H, C^{13}**H**), 8.12 (d, *J* = 7.6 Hz, 1H, C^{15}**H**), 7.39 (d, *J* = 8.2 Hz, 1H, C^{16}**H**), 7.25 (d, *J* = 2.2 Hz, 2H, 2 x C^6**H**), 6.82 (t, *J* = 2.2 Hz, 1H, C^9**H**), 6.21 (s, 2H, 2 x C^{10}**H$_2$**), 3.88 (s, 6H, 6 x C^8**H$_3$**).
^{13}C NMR (126 MHz, DMSO-d_6) δ 161.4 (2 x C^{7}H), 155.8 (C^4), 145.8 (2 x C^{2}H), 136.9 (C^{11}), 135.3 (C^5), 130.8, (C^{16}H), 130.5 (d, *J* = 3.8 Hz, C^{15}H), 129.9 (q, *J* = 33.3 Hz, C^{12}/C^{14}), 127.8 (q, *J* = 31.8 Hz, C^{12}/C^{14}), 125.3 (2 x C^{3}H), 124.1 – 123.7 (m, C^{13}H), 123.22 (q, *J* = 274.4, Hz, C^{17}F$_3$/C^{18}F$_3$), 123.18 (q, *J* = 272.1, Hz, C^{17}F$_3$/C^{18}F$_3$), 106.4 (2 x C^{6}H), 104.2 (C^{9}H), 59.0 (q, *J* = 3.3 Hz, C^{10}H$_2$), 55.8 (2 x C^{8}H$_3$).
^{19}F NMR (377 MHz, DMSO-d_6) δ –59.0, –61.4.
IR (neat) (cm^{-1}): 2981, 2889, 1341, 1275, 1182, 1156, 1135, 1115, 1083, 1064.

***N*-(2,4-bis(Trifluoromethyl)benzyl)-4-(3,5-bis(trifluoromethyl) phenyl) pyridinium iodide (1p)**

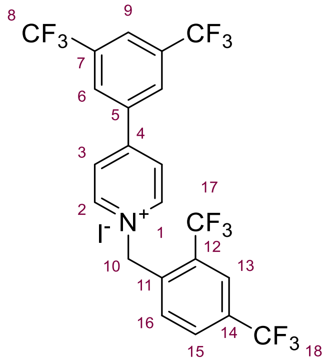

The title compound was prepared by General Procedure D using 4-(3,5-bis(trifluoromethyl) phenyl)pyridine **3p** (285 mg, 1.00 mmol) and 2,4-bis(trifluoromethyl)benzyl iodide **S4** (530 mg, 1.50 mmol) to give the pyridinium **1p** (454 mg, 72%) as a yellow solid.

m.p. (acetone): 259-261 °C

HRMS (ESI): Exact mass calculated for $C_{22}H_{12}NF_{12}$ $[M]^+$: 518.07726, found 518.07581

^{1}H NMR (400 MHz, DMSO-d_6) δ 9.28 (d, *J* = 7.1 Hz, 2H, 2 x C^2**H**), 8.87 (d, *J* = 7.1 Hz, 2H, 2 x C^3**H**), 8.78 (s, 2H, 2 x C^6**H**), 8.45 (s, 1H, C^9**H**), 8.24 (s, 1H, C^{13}**H**), 8.12 (d, *J* = 8.1 Hz, 1H, C^{15}**H**), 7.35 (d, *J* = 8.2 Hz, 1H, C^{16}**H**), 6.28 (s, 2H, 2 x C^{10}**H**).

^{13}C NMR (126 MHz, DMSO-d_6) δ 153.1 (C^4), 146.2 (2 x C^{2}H), 136.9 (C), 136.3 (C), 131.5 (q, *J* = 33.5 Hz, 2 x C^7), 130.6 (C^{16}H), 130.5 (q, *J* = 3.9 Hz, C^{15}H), 130.0 (q, *J* = 33.3 Hz, C^{12}/C^{14}), 129.5 (q, *J* = 4.0 Hz, 2 x C^{6}H), 127.8 (q, *J* = 31.8 Hz, C^{12}/C^{14}), 126.5 (2 x C^{3}H), 125.6 – 125.3 (m, C^{9}H), 123.9 (C^{13}H), 123.19 (q, *J* = 274.6 Hz, C^{17}F$_3$/C^{18}F$_3$), 123.15 (q, *J* = 272.7 Hz, C^{17}F$_3$/C^{18}F$_3$), 123.1 (q, *J* = 273.2 Hz, 2 x C^{8}F$_3$), 59.4 (q, *J* = 3.5 Hz, C^{10}H$_2$).

^{19}F NMR (377 MHz, DMSO-d_6) δ –59.1, –61.1, –61.5.

IR (neat) (cm^{-1}): 2981, 1639, 1345, 1272, 1176, 1122, 1084, 1053, 671.

_N_-(2,4-bis(Trifluoromethyl)benzyl)-4-(3-(methoxycarbonyl) phenyl)pyridinium iodide (1q)

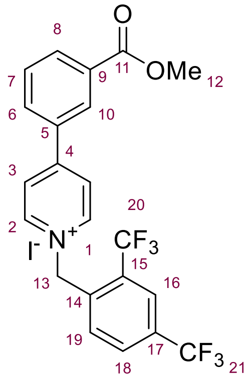

The title compound was prepared by General Procedure D using 4-(3-(methoxycarbonyl) phenyl)pyridine **3q** (427 mg, 2.00 mmol) and 2,4-bis(trifluoromethyl)benzyl iodide **S4** (1.06 g, 3.00 mmol) to give the pyridinium **1q** (1.01 g, 89%) as a yellow solid.

m.p. (acetone): 206-208 °C

HRMS (ESI): Exact mass calculated for $C_{22}H_{16}O_2NF_6$ $[M]^+$: 440.10797, found 440.10828

^{1}H NMR (400 MHz, DMSO-d_6) δ 9.16 (d, *J* = 7.0 Hz, 2H, 2 x C^2**H**), 8.69 (d, *J* = 7.0 Hz, 2H, 2 x C^3**H**), 8.57 (t, *J* = 1.7 Hz, 1H, C^{10}**H**), 8.37 (ddd, *J* = 7.8, 2.1, 1.1 Hz, 1H, C^8**H**), 8.27 – 8.21 (m, 2H, C^6**H** and C^{16}**H**), 8.12 (d, *J* = 8.1 Hz, 1H, C^{18}**H**), 7.84 (t, *J* = 7.7 Hz, 1H, C^7**H**), 7.40 (d, *J* = 8.2 Hz, 1H, C^{19}**H**), 6.23 (s, 2H, 2 x C^{13}**H$_2$**), 3.93 (s, 3H, 3 x C^{12}**H$_3$**).

^{13}C NMR (126 MHz, DMSO-d_6) δ 165.6 (C^{11}), 155.1 (C^4), 146.1 (2 x C^{2}H), 136.9 (C), 134.3 (C), 133.0 (C^{8}H), 132.6 (C^{6}H), 131.2 (C), 130.9 (C^{19}H), 130.5 (d, *J* = 4.7 Hz, C^{18}H), 130.5 (C^{7}H), 130.0 (q, *J* = 33.3 Hz, C^{15}/C^{17}), 128.9 (C^{10}H), 127.9 (q, *J* = 31.7 Hz, C^{15}/C^{17}), 125.7 (2 x C^{3}H), 124.0 – 123.8 (m, C^{16}H), 123.23 (q, *J* = 274.5 Hz, C^{20}F$_3$/C^{21}F$_3$) 123.2 (q, *J* = 272.7 Hz, C^{20}F$_3$/C^{21}F$_3$), 59.1 (q, *J* = 3.6 Hz, C^{13}H$_2$), 52.7 (C^{12}H$_3$).

^{19}F NMR (376 MHz, DMSO-d_6) δ –59.0, –61.5.

IR (neat) (cm^{-1}): 2981, 1707, 1639, 1345, 1273, 1258, 1181, 1119, 1085, 1054.

_N_-(2,4-bis(Trifluoromethyl)benzyl)-4-(4-fluorophenyl)-3-(o-tolyl)pyridinium iodide (1r)

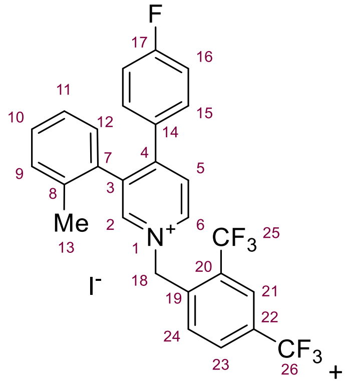

The title compound was prepared by General Procedure D using 4-(4-fluorophenyl)-3-(o-tolyl)pyridine **3r** (263 mg, 1.00 mmol) and 2,4-bis(trifluoromethyl)benzyl iodide **S4** (530 mg, 1.50 mmol) to give the pyridinium **1r** (461 mg, 75%) as a yellow solid.

m.p. (acetone): 252-254 °C

HRMS (ESI): Exact mass calculated for $C_{27}H_{19}NF_7^+$ $[M]^+$: 490.100, found 490.1398

^{1}H NMR (400 MHz, DMSO) δ 9.26 (d, *J* = 1.6 Hz, 1H, C^2**H**), 9.15 (dd, *J* = 6.5, 1.6 Hz, 1H, C^6**H**), 8.40 (d, *J* = 6.4 Hz, 1H, C^5**H**), 8.23 (s, 1H, C^{21}**H**), 8.15 (d, *J* = 8.5 Hz, 1H, C^{23}**H**), 7.52 (d, *J* = 8.2 Hz, 1H, C^{24}**H**), 7.44 – 7.21 (m, 8H, C^9**H**, C^{10}**H**, C^{11}**H**, C^{12}**H**, 2 x C^{15}**H** + 2 x C^{16}**H**), 6.25 (s, 2H, C^2**H$_2$**), 1.89 (s, 3H, C^2**H$_3$**).

^{13}C NMR (126 MHz, DMSO) δ 163.20 (d, *J* = 250.0 Hz, C^{17}), 155.6 (C^4), 146.8 (C^2), 144.3 (C^6), 138.9 (C), 136.9 (C^{19}), 135.7 (C), 133.3 (C), 131.8 (d, *J* = 8.8 Hz 2 x C^{15}H), 131.4 (d, *J* = 3.0 Hz, C^{14}), 130.8 (C^{24}H), 2 x 130.6 (2 x ArCH), 130.5 (q, *J* = 3.6 Hz, C^{23}H), 129.9 (q, *J* = 33.3 Hz, C^{20}/C^{22}), 129.6 (ArCH), 128.7 (C^5), 127.8 (q, *J* = 31.7 Hz, C^{20}/C^{22}), 126.4 (ArCH), 123.9 – 123.6 (m, C^{21}H), 123.2 (q, *J* = 274.4 Hz, $C^{25}F_3$/$C^{26}F_3$), 123.1 (q, *J* = 272.5 Hz, $C^{25}F_3$/$C^{26}F_3$), 116.0 (d, *J* = 22.0 Hz, 2 x C^{16}H), 59.2 (q, *J* = 3.4 Hz, $C^{18}H_2$), 19.3 ($C^{13}H_3$).

^{19}F NMR (377 MHz, DMSO) δ –59.1, –61.4, –109.7 (tt, *J* = 8.7, 5.2 Hz).

IR (neat) (cm^{-1}): 2979, 2932, 1633, 1600, 1483, 1470, 1340, 1281, 1269, 1163.

_N_-(2,4-bis(Trifluoromethyl)benzyl)-4-(4-fluorophenyl)-3-(3-methoxyphenyl)pyridinium iodide (1s)

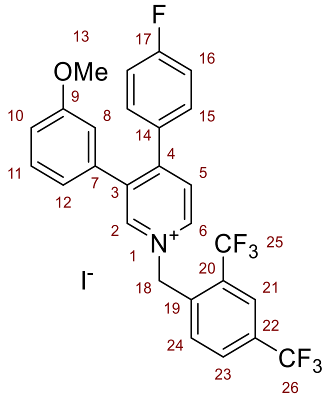

The title compound was prepared by General Procedure D using 4-(4-fluorophenyl)-3-(3-methoxyphenyl)pyridine **3s** (279 mg, 1.00 mmol) and 2,4-bis(trifluoromethyl)benzyl iodide **S4** (530 mg, 1.50 mmol) to give the pyridinium **1s** (495 mg, 78%) as a yellow solid.

m.p. (acetone): 188-190 °C

HRMS (ESI): Exact mass calculated for $C_{27}H_{19}ONF_7^+$ $[M]^+$: 506.1349, found: 506.1347

^{1}H NMR (400 MHz, DMSO) δ 9.34 (d, *J* = 1.5 Hz, 1H, C^2**H**), 9.10 (dd, *J* = 6.5, 1.6 Hz, 1H, C^6**H**), 8.33 (d, *J* = 6.4 Hz, 1H, C^5**H**), 8.24 (s, 1H, C^{21}**H**), 8.13 (d, *J* = 8.0 Hz, 1H, C^{23}**H**), 7.49 (d, *J* = 8.3 Hz, 1H, C^{24}**H**), 7.43 (ddt, *J* = 8.3, 5.2, 2.6 Hz, 2H, 2 x C^{15}**H**), 7.37 – 7.28 (m, 3H, C^{11}**H** + 2 x C^{16}**H**), 7.02 (ddd, *J* = 8.4, 2.6, 0.9 Hz, 1H,

C^{12}**H**), 6.91 (dd, *J* = 2.6, 1.6 Hz, 1H, C^{8}**H**), 6.81 (ddd, *J* = 7.7, 1.7, 1.0 Hz, 1H, C^{10}**H**), 6.26 (s, 2H, C^{18}**H_2**), 3.69 (s, 3H, C^{13}**H_3**).
^{13}C NMR (126 MHz, DMSO) δ 163.1 (d, *J* = 249.2 Hz, C^{17}), 159.4 (C^{9}), 155.3 (C^{4}), 146.5 (C^{2}H), 143.9 (C^{6}H), 139.1 (C), 137.0 (C^{19}), 134.9 (C), 132.0 (d, *J* = 8.9 Hz, 2 x C^{15}H), 131.5 (d, *J* = 3.1 Hz, C^{14}), 130.5 (C^{24}H), 130.4 (C^{23}H), 130.1 (C^{8}H), 129.8 (q, *J* = 33.3 Hz, C^{20}/C^{22}), 129.1 (C^{5}H), 127.7 (q, *J* = 31.9 Hz, C^{20}/C^{22}), 123.9 – 123.5 (m, C^{21}H), 123.21 (q, *J* = 274.5 Hz, $C^{25}F_3$/$C^{26}F_3$), 123.17 (q, *J* = 272.7 Hz, $C^{25}F_3$/$C^{26}F_3$), 121.9 (C^{12}H), 116.0 (d, *J* = 22.1 Hz, 2 x C^{16}H), 115.6 (C^{11}H), 114.6 (C^{10}H), 59.2 (d, *J* = 3.5 Hz, $C^{18}H_2$), 55.3 9 $C^{13}H_3$).
^{19}F NMR (377 MHz, DMSO) δ –59.0, –61.4, –110.1 (tt, *J* = 8.9, 5.4 Hz).
IR (neat) (cm^{-1}): 2922, 1632, 1603, 1341, 1274, 1237, 1180, 1163, 1129, 1086.

***N*-(2,4-bis(Trifluoromethyl)benzyl)-3,4-bis(4-fluorophenyl)pyridinium iodide (1t)**

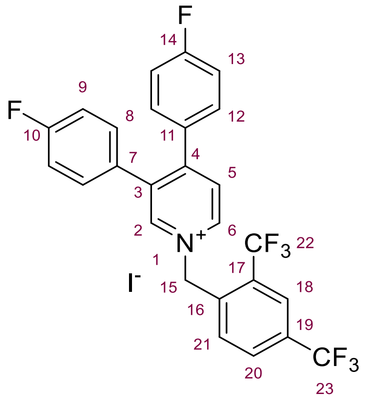

The title compound was prepared by General Procedure D using 3,4-bis(4-fluorophenyl)pyridine **3t** (158 mg, 0.60 mmol) and 2,4-bis(trifluoromethyl)benzyl iodide **S4** (350 mg, 1.00 mmol) to give the pyridinium **1t** (187 mg, 51%) as a yellow solid.
m.p. (acetone): 205-207°C
HRMS (ESI): Exact mass calculated for $C_{26}H_{16}NF_8^+$ $[M]^+$: 494.1150, found 494.1140
^{1}H NMR (400 MHz, DMSO) δ 9.34 (d, *J* = 1.6 Hz, 1H, C^{2}**H**), 9.11 (dd, *J* = 6.5, 1.5 Hz, 1H, C^{6}**H**), 8.34 (d, *J* = 6.4 Hz, 1H, C^{5}**H**), 8.24 (s, 1H, C^{18}**H**), 8.13 (d, *J* = 8.1 Hz, 1H, C^{20}**H**), 7.49 (d, *J* = 8.2 Hz, 1H, C^{21}**H**), 7.44 – 7.38 (m, 2H, 2 x C^{12}**H**), 7.38 – 7.25 (m, 6H, 2 x C^{8}**H** + 2 x C^{9}**H** + 2 x C^{13}**H**), 6.26 (s, 2H, C^{2}**H_2**).
^{13}C NMR (126 MHz, DMSO) δ 163.1 (d, *J* = 249.3 Hz, C^{14}), 162.5 (d, *J* = 247.3 Hz, C^{10}), 155.4 (C^{4}), 146.5 (C^{2}H), 144.0 (C^{6}H), 138.4 (C^{3}), 137.0 (C^{16}), 132.2 (2 x C^{8}H/C^{12}H), 132.1 (2 x C^{8}H/C^{12}H), 131.3 (d, *J* = 3.3 Hz, C^{7}/C^{11}), 130.5 (C), 130.4 (q, *J* = 3.8 Hz, C^{20}H), 130.1 (d, *J* = 3.2 Hz, C^{7}/C^{11}), 129.8 (q, *J* = 33.3 Hz, C^{17}/C^{19}), 129.1 (C^{5}H), 127.7 (q, *J* = 31.9 Hz, C^{17}/C^{19}), 124.0 – 123.6 (m, C^{18}H), 123.21 (q, *J* = 274.7 Hz, $C^{22}F_3$/$C^{23}F_3$), 123.17 (q, *J* = 272.7 Hz, $C^{22}F_3$/$C^{23}F_3$), 116.1 (d, *J* = 22.1 Hz, 2 x C^{13}H), 116.0 (d, *J* = 21.9 Hz, 2 x C^{9}H), 59.2 (q, *J* = 3.4 Hz, $C^{15}H_2$).
^{19}F NMR (377 MHz, DMSO) δ –59.0, –61.4, –110.1 (td, *J* = 9.0, 4.4 Hz), –111.9 (tt, *J* = 9.0, 5.8 Hz).
IR (neat) (cm^{-1}): 2980, 1633, 1603, 1493, 1463, 1342, 1270, 1168, 1138, 1122..

N-(2,4-bis(Trifluoromethyl)benzyl)-3-butyl-4-(4-fluorophenyl)pyridinium iodide (1u)

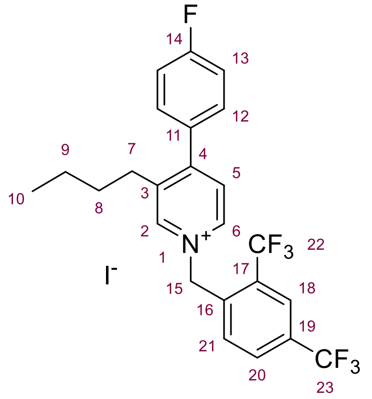

The title compound was prepared by General Procedure D using 3,4-bis(4-fluorophenyl)pyridine **3u** (158 mg, 0.60 mmol) and 2,4-bis(trifluoromethyl)benzyl iodide **S4** (350 mg, 1.00 mmol) to give the pyridinium **1u** (187 mg, 51%) as a white wax.

HRMS (ESI): Exact mass calculated for $C_{24}H_{21}NF_8^+$ $[M]^+$:456.1557, found 456.1552

^{1}H NMR (500 MHz, DMSO) δ 9.16 (d, *J* = 1.6 Hz, 1H, C^2**H**), 8.98 (dd, *J* = 6.3, 1.6 Hz, 1H, C^6**H**), 8.24 (s, 1H, C^{18}**H**), 8.15 (dd, *J* = 8.2, 1.9 Hz, 1H, C^{20}**H**), 8.12 (d, *J* = 6.4 Hz, 1H, C^5**H**), 7.70 – 7.59 (m, 2H, 2 x C^{12}**H**), 7.51 – 7.43 (m, 2H, 2 x C^{13}**H**), 7.39 (d, *J* = 8.2 Hz, 1H, C^{21}**H**), 6.22 (s, 2H, C^2**H$_2$**), 2.84 – 2.76 (m, 2H, C^7**H$_2$**), 1.51 – 1.39 (m, 2H, C^8**H$_2$**), 1.19 (q, *J* = 7.4 Hz, 2H, C^9**H$_2$**), 0.76 (t, *J* = 7.3 Hz, 3H, C^{10}**H$_3$**).

^{13}C NMR (126 MHz, DMSO) δ 163.1 (d, *J* = 248.4 Hz, C^{14}), 157.0 (C^4), 145.9 (C^{2}H), 142.8 (C^{6}H), 140.9 (C^3), 136.8 (C^{16}), 131.8 (d, *J* = 3.3 Hz, C^{11}), 131.1 (d, *J* = 8.9 Hz, 2 x C^{12}H), 130.8 (C^{21}H), 130.4 (q, *J* = 20.7 Hz, C^{20}H), 129.9 (q, *J* = 33.2 Hz, C^{17}/C^{19}), 128.9 (C^{5}H), 127.8 (q, *J* = 31.8 Hz, C^{17}/C^{19}), 124.0 – 123.6 (m, C^{18}H), 123.2 (q, *J* = 274.5 Hz, C^{22}F$_3$/C^{23}F$_3$), 123.1 (q, *J* = 272.7 Hz, C^{22}F$_3$/C^{23}F$_3$), 116.2 (d, *J* = 21.9 Hz, 2 x C^{13}H), 59.3(q, *J* = 3.4 Hz, C^{15}H$_2$), 31.2 (C^{8}H$_2$), 29.5 (C^{7}H$_2$), 21.6 (C^{9}H$_2$), 13.4 (C^{10}H$_3$).

^{19}F NMR (377 MHz, DMSO) δ –59.1, –61.4, –110.8 (tt, *J* = 8.9, 5.3 Hz).

IR (neat) (cm^{-1}): 2981, 1638, 1605, 1500, 1464, 1342, 1276, 1183, 1137, 1123.

4-(Benzofuran-5-yl)-N-(2,4-bis(trifluoromethyl)benzyl)pyridinium iodide (1v)

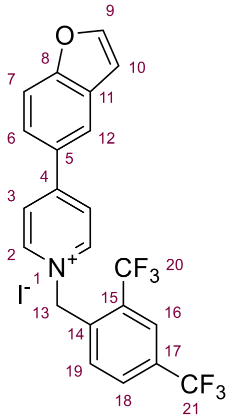

The title compound was prepared by General Procedure D using 4-(benzofuran-5-yl)pyridine **3v** (195 mg, 1.00 mmol) and 2,4-bis(trifluoromethyl)benzyl iodide **S4** (530 mg, 1.50 mmol) to give the pyridinium **1v** (278 mg, 51%) as a pale yellow solid.

m.p. (acetone): 247-249 °C

HRMS (ESI): Exact mass calculated for $C_{22}H_{14}ONF_3^+$ $[M]^+$:422.0974, found 422.0981

^{1}H NMR (500 MHz, DMSO) δ 9.12 (d, *J* = 6.9 Hz, 2H, 2 x C^2**H**), 8.67 (d, *J* = 7.1 Hz, 2H, 2 x C^3**H**), 8.52 (d, *J* = 2.0 Hz, 1H, C^{12}**H**), 8.23 (s, 1H, C^{16}**H**), 8.20 (d, *J* = 2.2 Hz, 1H, C^9**H**), 8.14 (dd, *J* = 8.1, 2.1 Hz, 1H, C^{18}**H**), 8.10 (dd, *J* = 8.7, 2.1 Hz, 1H, C^6**H**), 7.91 (d, *J* = 8.7 Hz, 1H, C^7**H**), 7.43 (d, *J* = 8.2 Hz, 1H, C^{19}**H**), 7.16 (dd, *J* = 2.3, 1.0 Hz, 1H, C^{10}**H**), 6.20 (s, 2H, C^{13}**H$_2$**).

^{13}C NMR (126 MHz, DMSO) δ 156.5 (C), 156.3 (C), 148.2 (C^{9}H), 145.6 (2 x C^{2}H), 136.9 (C), 131.0 (C^{19}H), 130.5 (q, J = 3.8 Hz, C^{18}H), 129.9 (q, J = 33.2 Hz, C^{15}/C^{17}), 128.6 (C), 128.4 (C), 127.8 (q, J = 31.6 Hz, C^{15}/C^{17}), 124.9 (C^{6}H), 124.8 (2 x C^{3}H), 123.9 – 123.6 (m, C^{16}H), 123.19 (q, J = 274.6 Hz, $C^{20}F_3$/$C^{21}F_3$), 123.15 (q, J = 272.7 Hz, $C^{20}F_3$/$C^{21}F_3$), 122.4 (C^{12}H), 112.8 (C^{7}H), 107.3 (C^{10}H), 58.8 (q, J = 3.4 Hz, $C^{13}H_2$).
^{19}F NMR (377 MHz, DMSO) δ –59.0, –61.4.
IR (neat) (cm^{-1}): 3023, 1638, 1343, 1309, 1270, 1193, 1177, 1150, 1134, 1125.

***N*-(2,4-bis(Trifluoromethyl)benzyl)-4-(1-methyl-1H-indol-7-yl)pyridinium iodide (1w)**

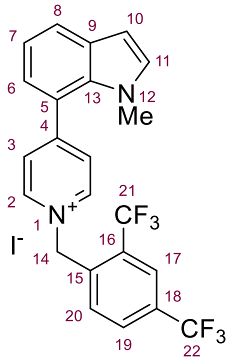

The title compound was prepared by General Procedure D using 4-(1-methyl-1H-indol-7-yl)pyridine **3w** (208 mg, 1.00 mmol) and 2,4-bis(trifluoromethyl)benzyl iodide **S4** (530 mg, 1.50 mmol) to give the pyridinium **1w** (465 mg, 83%) as a pale yellow solid.
m.p. (acetone): 246-248 °C
HRMS (ESI): Exact mass calculated for $C_{23}H_{17}N_2F_6^+$ $[M]^+$: 435.1290, found 432.1288
^{1}H NMR (500 MHz, DMSO) δ 9.12 (d, J = 6.8 Hz, 2H, 2 x C^{2}**H**), 8.35 (d, J = 6.8 Hz, 2H, 2 x C^{3}**H**), 8.25 (s, 1H, C^{17}**H**), 8.20 (dd, J = 8.3, 2.0 Hz, 1H, C^{19}**H**), 7.83 (dd, J = 6.8, 2.1 Hz, 1H, C^{6}**H**), 7.58 (d, J = 8.2 Hz, 1H, C^{20}**H**), 7.47 (d, J = 3.2 Hz, 1H, C^{11}**H**), 7.28 – 7.20 (m, 2H, C^{7}**H** + C^{8}**H**), 6.67 (d, J = 3.2 Hz, 1H, C^{10}**H**), 6.25 (s, 2H, C^{14}**H_2**), 3.48 (s, 3H, C^{12}**H_2**).
^{13}C NMR (126 MHz, DMSO) δ 157.1 (C^{4}), 144.6 (2 x C^{2}H), 136.5 (C), 133.0 (C^{11}H), 132.6 (C), 131.7 (C^{20}H), 130.6 (q, J = 3.8 Hz, C^{19}H), 130.4 (ArC), 130.1 (q, J = 33.2 Hz, C^{16}/C^{18}), 128.7 (2 x C^{3}H), 128.1 (q, J = 31.6 Hz, C^{16}/C^{18}), 125.0 (C^{7}H/C^{8}H), 124.1 – 123.8 (m, C^{17}H), 123.7 (C^{6}H), 123.2 (q, J = 274.6 Hz, $C^{21}F_3$/$C^{22}F_3$), 123.1 (q, J = 272.6 Hz, $C^{21}F_3$/$C^{22}F_3$), 120.4 (ArC), 119.3(C^{7}H/C^{8}H), 101.7 (C^{10}H), 59.1 (q, J = 3.1 Hz, $C^{14}H_2$), 37.5 ($C^{12}H_3$).
^{19}F NMR (377 MHz, DMSO) δ –58.93, –61.46.
IR (neat) (cm^{-1}): 1632, 1341, 1308, 1275, 1172, 1129, 1083, 1052, 796, 723.

***N*-(2,4-bis(Trifluoromethyl)benzyl)-4-(9-ethyl-9H-carbazol-3-yl)pyridinium iodide (1x)**

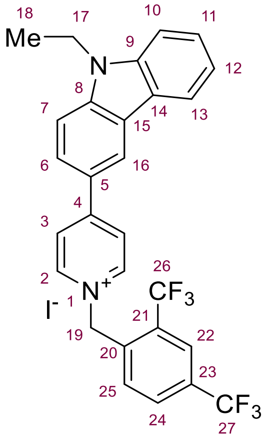

The title compound was prepared by General Procedure D using 4-(9-ethyl-9H-carbazol-3-yl)pyridine **3x** (272 mg, 1.00 mmol) and 2,4-bis(trifluoromethyl)benzyl iodide **S4** (530 mg, 1.50 mmol) to give the pyridinium **1x** (606 mg, 97%) as a yellow solid.

m.p. (acetone): 219-221 °C

HRMS (ESI): Exact mass calculated for $C_{28}H_{21}N_2F_6^+$ $[M]^+$: 499.1603, found 499.1601

^{1}H NMR (400 MHz, DMSO) δ 9.17 (d, *J* = 2.0 Hz, 1H, C^{16}**H**), 9.05 (d, *J* = 7.1 Hz, 2H, 2 x C^{2}**H**), 8.74 (d, *J* = 7.2 Hz, 2H, 2 x C^{3}**H**), 8.34 (dt, *J* = 7.8, 1.0 Hz, 1H, C^{13}**H**), 8.31 (dd, *J* = 8.8, 2.0 Hz, 1H, C^{6}**H**), 8.24 (s, 1H, C^{22}**H**), 8.14 (dd, *J* = 8.3, 2.0 Hz, 1H, C^{24}**H**), 7.89 (d, *J* = 8.8 Hz, 1H, C^{7}**H**), 7.73 (d, *J* = 8.3, 0.9 Hz, 1H, C^{10}**H**), 7.57 (ddd, *J* = 8.3, 7.2, 1.2 Hz, 1H, C^{11}**H**), 7.40 (d, *J* = 8.3 Hz, 1H, C^{25}**H**), 7.34 (ddd, *J* = 7.9, 7.2, 0.9 Hz, 1H, C^{12}**H**), 6.17 (s, 2H, C^{19}**H$_2$**), 4.55 (q, *J* = 7.1 Hz, 2H, C^{17}**H$_2$**), 1.37 (t, *J* = 7.1 Hz, 3H, C^{18}**H$_3$**).

^{13}C NMR (126 MHz, DMSO) δ 156.5 (C^{4}), 145.2 (2 x C^{2}H), 142.2 (2 x C), 140.4 (2 x C), 137.4 (C^{20}), 130.5 (C^{24}H + C^{25}H), 129.8 (q, *J* = 33.3 Hz, C^{21}/C^{23}), 127.7 (q, *J* = 31.8 Hz, C^{21}/C^{23}), 127.0 (C^{11}H), 126.0 (C^{6}H), 123.9 – 123.7 (m, C^{22}H), 123.4 (2 x C^{3}H), 123.22 (q, *J* = 274.9 Hz, C^{26}F$_3$/C^{27}F$_3$), 123.22 (C), 123.17 (q, *J* = 272.7 Hz, C^{26}F$_3$/C^{27}F$_3$), 122.4 (C), 121.8 (C^{16}H), 121.0 (C^{13}H), 120.2 (C^{12}H), 110.5 (C^{7}H), 110.1 (C^{10}H), 58.4 (q, *J* = 3.7 Hz, C^{19}H$_2$), 37.4 (C^{17}H$_2$), 13.8 (C^{18}H$_3$).

^{19}F NMR (377 MHz, DMSO) δ –59.1, –61.4.

IR (neat) (cm^{-1}): 3658, 2980, 2973, 1622, 1590, 1494, 1480, 1343, 1273, 1162.

***N*-(2,4-bis(Trifluoromethyl)benzyl)-4-(3-(ethoxycarbonyl)-1-ethyl-4-oxo-1,4-dihydroquinolin-7-yl)pyridinium iodide (1y)**

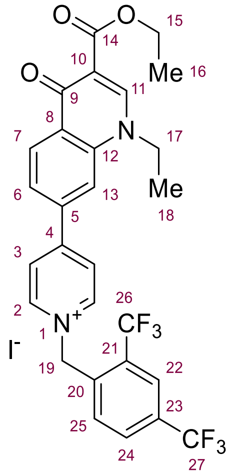

The title compound was prepared by General Procedure D using 4-(3-(ethoxycarbonyl)-1-ethyl-4-oxo-1,4-dihydroquinolin-7-yl)pyridine **3y** (600 mg, 1.90 mmol) and 2,4-bis(trifluoromethyl)benzyl iodide **S4** (1.32 g, 3.70 mmol) to give the pyridinium **1y** (1.13 g, 90%) as an orange solid.

m.p. (acetone): 209-211 °C

HRMS (ESI): Exact mass calculated for $C_{28}H_{23}O_3N_2F_6^+$ $[M]^+$: 549.1607, found 549.1604

^{1}H NMR (400 MHz, DMSO) δ 9.25 (d, *J* = 6.4 Hz, 2H, 2 x C^{2}**H**), 8.82 (d, *J* = 7.8 Hz, 3H, 2 x C^{2}**H** + C^{11}**H**), 8.46 (d, *J* = 8.3 Hz, 1H, C^{7}**H**), 8.39 (s, 1H, C^{13}**H**), 8.25 (s, 1H, C^{22}**H**), 8.18 – 8.10 (m, 2H, C^{6}**H** + C^{24}**H**), 7.42 (d, *J* = 8.2 Hz, 1H, C^{25}**H**), 6.27 (s, 2H, C^{19}**H$_2$**), 4.61 (q, *J* = 7.1 Hz, 2H, C^{17}**H$_2$**), 4.25 (q, *J* = 7.0 Hz, 2H, C^{15}**H$_2$**), 1.43 (t, *J* = 7.0 Hz, 3H, C^{18}**H$_3$**), 1.30 (t, *J* = 7.1 Hz, 3H, C^{16}**H$_3$**).

^{13}C NMR (126 MHz, DMSO) δ 172.2 (C^{9}), 164.4 (C^{14}), 154.8 (C^{4}), 150.0 (C^{11}H), 146.0 (2 x C^{2}H), 139.2 (C), 137.4 (C), 136.8 (C^{20}), 130.8 (C^{25}H), 130.5 (q, *J* = 4.0 Hz, C^{24}H), 130.0 (C^{8}), 129.9 (q, *J* = 33.4 Hz, C^{21}/C^{23}), 128.0 (C^{7}H), 127.8 (q, *J* = 31.7 Hz, C^{21}/C^{23}), 126.3 (2 x C^{3}H), 124.1 (C^{6}H), 124.0 – 123.7 (m, C^{22}H), 123.2 (q, *J* = 274.6 Hz, C^{26}F$_3$/C^{27}F$_3$), 123.1 (q, *J* = 272.5 Hz, C^{26}F$_3$/C^{27}F$_3$), 117.6 (C^{13}H), 111.0 (C^{10}), 59.9 (C^{15}H$_2$), 59.2 (q, *J* = 3.3 Hz, C^{19}H$_2$), 48.1 (C^{17}H$_2$), 14.6 (C^{18}H$_3$), 14.3 (C^{16}H$_3$).

^{19}F NMR (377 MHz, DMSO) δ –59.0, –61.4.

IR (neat) (cm^{-1}): 3433, 1720, 1611, 1472, 1348, 1306, 1275, 1219, 1188, 1171.

N-(2,4-bis(Trifluoromethyl)benzyl)-4-methoxpyridinium iodide (1z)

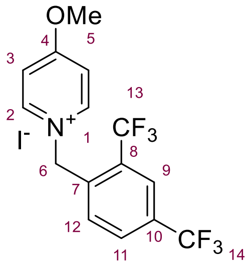

The title compound was prepared by General Procedure D using 4-methoxypyridine (0.2 mL, 2.00 mmol) and 2,4-bis(trifluoromethyl)benzyl iodide **S4** (1.06 g, 3.00 mmol) to give the pyridinium **1z** (853 mg, 92%) as a white solid.

m.p. (acetone): 185-187 °C

HRMS (ESI): Exact mass calculated for $C_{15}H_{12}ONF_6$ $[M]^+$: 336.08176, found 336.08113

^{1}H NMR (400 MHz, DMSO-d_6) δ 8.89 (d, *J* = 7.5 Hz, 2H, 2 x C^2**H**), 8.20 (s, 1H, C^9**H**), 8.11 (d, *J* = 8.2 Hz, 1H, C^{12}**H**), 7.73 (d, *J* = 7.7 Hz, 1H, C^{11}**H**), 7.30 (d, *J* = 8.2 Hz, 2H, 2 x C^3**H**), 6.04 (s, 2H, 2 x C^6**H**), 4.15 (s, 3H, 3 x C^5**H**).

^{13}C NMR (126 MHz, DMSO-d_6) δ 171.4 (C^4), 147.1 (2 x C^{2}H), 137.4 (C^7), 130.6 (C^{12}H), 130.5 (d, *J* = 3.8 Hz, C^{11}H), 129.8 (q, *J* = 33.3 Hz, C^8/C^{10}), 127.7 (q, *J* = 31.8 Hz, C^8/C^{10}), 124.1 – 123.6 (m, C^{9}H), 123.2 (q, *J* = 275.3 Hz, C^{13}F$_3$ + C^{14}F$_3$), 114.0 (2 x C^{3}H), 58.4 (C^{5}H$_3$), 57.9 (q, *J* = 3.5 Hz, C^{6}H$_2$).

^{19}F NMR (377 MHz, DMSO-d_6) δ –59.1, –61.5.

IR (neat) (cm^{-1}): 2981, 1643, 1345, 1269, 1177, 1138, 1085, 1053, 843, 671.

N-(2,4-bis(Trifluoromethyl)benzyl)-4-methylpyridinium iodide (1aa)

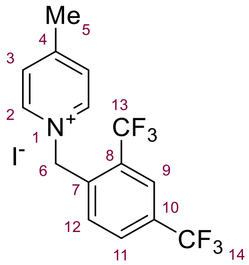

The title compound was prepared by General procedure D using 4-methylpyridine (0.2 mL, 2.00 mmol) and 2,4-bis(trifluoromethyl)benzyl iodide **S4** (1.06 g, 3.00 mmol) to give the pyridinium **1aa** (766 mg, 86%) as a pale brown solid.

m.p. (acetone): 192-194 °C

HRMS (ESI): Exact mass calculated for $C_{15}H_{12}NF_6^+$ $[M]^+$: 320.0870, found 320.0868

^{1}H NMR (400 MHz, DMSO) δ 8.93 (d, *J* = 6.8 Hz, 2H, 2 x C^2**H**), 8.20 (s, 1H, C^9**H**), 8.13 (dd, *J* = 8.1, 2.0 Hz, 1H, C^{11}**H**), 8.08 (d, *J* = 6.1 Hz, 2H, 2 x C^3**H**), 7.38 (d, *J* = 8.2 Hz, 1H, C^{12}**H**), 6.14 (s, 2H, C^6**H$_2$**), 2.67 (s, 3H, C^5**H$_3$**).

^{13}C NMR (126 MHz, DMSO) δ 160.6 (C^4), 144.7 (2 x C^{2}H), 136.6 (C^7), 131.4 (C^{12}H), 130.5 (q, *J* = 3.9 Hz, C^{11}H$_3$), 130.1 (q, *J* = 33.2 Hz, C^8/C^{10}), 128.9 (2 x C^{3}H), 128.0 (q, *J* = 31.8 Hz, C^8/C^{10}), 124.0 – 123.7 (m, C^{9}H), 123.1 (q, *J* = 273.2 Hz, C^{13}F$_3$/C^{14}F$_3$), 58.9 (q, *J* = 3.1 Hz, C^{6}H$_2$), 21.7 (C^{5}H$_3$).

^{19}F NMR (377 MHz, DMSO) δ –59.0, –61.5.

IR (neat) (cm^{-1}): 2981, 1642, 1346, 1282, 1268, 1178, 1139, 1116, 1085, 1053, 670.

N-(2,4-bis(Trifluoromethyl)benzyl)-4-tert-buthoxypyridinium iodide (1ab)

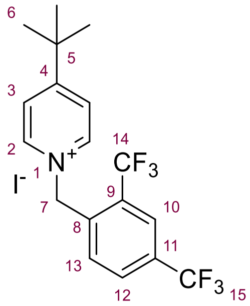

The title compound was prepared by General Procedure D using 4-methylpyridine (0.44 mL, 3.00 mmol) and 2,4-bis(trifluoromethyl)benzyl iodide **S4** (1.59 g, 4.50 mmol) to give the pyridinium **1ab** (1.56 g, 99%) as a white solid.

m.p. (acetone): 251-253 °C

HRMS (ESI): Exact mass calculated for $C_{18}H_{18}NF_6^+$ $[M]^+$: 362.1329, found: 362.1338

^{1}H NMR (400 MHz, DMSO) δ 8.99 (d, *J* = 7.0 Hz, 2H, 2 x C^2**H**), 8.26 (d, *J* = 7.0 Hz, 2H, 2 x C^3**H**), 8.22 (s, 1H, C^{10}**H**), 8.15 (d, *J* = 8.2 Hz, 1H, C^{12}**H**), 7.36 (d, *J* = 8.2 Hz, 1H, C^{13}**H**), 6.16 (s, 2H, C^7**H$_2$**), 1.40 (s, 9H, 3 x C^2**H$_3$**).

^{13}C NMR (126 MHz, DMSO) δ 171.4 (C^4), 145.1 (2 x C^{2}H), 136.6 (C^8), 131.3 (C^{13}H), 130.6 (q, *J* = 3.7 Hz, C^{12}H), 130.0 (q, *J* = 33.2 Hz, C^9/C^{11}), 127.98 (q, *J* = 31.8 Hz, C^9/C^{11}), 125.6 (2 x C^{2}H), 124.0 – 123.63 (m, C^{10}H), 123.2 (d, *J* = 274.9 Hz, C^{14}F$_3$ + C^{15}F$_3$), 58.8 (q, *J* = 3.3 Hz, C^{7}H$_2$), 36.6 (C^5), 29.54 (3 x C^{6}H$_3$).

^{19}F NMR (377 MHz, DMSO) δ –59.0, –61.5.

IR (neat) (cm^{-1}): 2972, 1642, 1340, 1271, 1177, 1164, 1129, 1084, 1051, 847.

N-(2,4-bis(trifluoromethyl)benzyl)pyridinium (1ac)

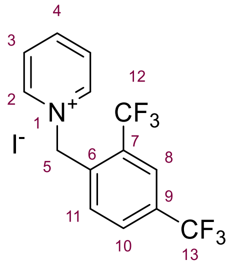

The title compound was prepared by General Procedure D using pyridine (81 µL, 1.00 mmol) and 2,4-bis(trifluoromethyl)benzyl iodide **S4** (365 mg, 1.20 mmol) to give the pyridinium **1ac** (311 mg, 76%) as a pale yellow solid.

m.p. (acetone): 199-201 °C

HRMS (ESI): Exact mass calculated for $C_{14}H_{10}NF_6^+$ $[M]^+$: 306.0712, found: 306.0710

^{1}H NMR (400 MHz, DMSO) δ 9.09 (dd, *J* = 6.7, 1.4 Hz, 2H, 2 x C^2**H**), 8.73 (tt, *J* = 7.8, 1.3 Hz, 1H, C^4**H**), 8.28 – 8.22 (m, 2H, 2 x C^3**H**), 8.20 (d, *J* = 1.9 Hz, 1H, C^8**H**), 8.14 (dd, *J* = 8.2, 2.0 Hz, 1H, C^{10}**H**), 7.44 (d, *J* = 8.2 Hz, 1H, C^{11}**H**), 6.22 (s, 2H, C^5**H$_2$**).

^{13}C NMR (101 MHz, DMSO) δ 147.1 (C^{4}H), 145.8 (C^{2}H), 136.2 (C^6), 131.9 (C^{11}H), 130.7 (C^{10}H), 130.3 (q, *J* = 33.3 Hz, C^7/C^9), 128.8, 128.2 (q, *J* = 31.7 Hz, C^7/C^9), 124.3 – 123.6 (m, C^{8}H), 123.2 (q, *J* = 273.7 Hz, C^{12}F$_3$ + C^{13}F$_3$), 59.9 (q, *J* = 3.2 Hz, C^{5}H$_2$).

^{19}F NMR (377 MHz, DMSO) δ –59.0, –61.6.

IR (neat) (cm^{-1}): 3076, 2160, 2033, 1482, 1348, 1274, 1177, 1142, 1122, 1055.

The Synthesis of Tetrahydropyridines

General procedure E:

Substrate (1.00 equiv.), KI (4.00 equiv.), paraformaldehyde (30.0 equiv.), and [Ru(*p*-cymene)Cl_2]$_2$ (2 mol%) was added to a microwave vial. MeOH (0.2 M) and Mg(OMe)$_2$ (1.00 equiv, 0.89M in MeOH) was added and the solution heated at 65 °C for 16 hours. The solution concentrated under reduce pressure and re-dissolved in CH_2Cl_2 (30 m L), brine (50 mL) and water (50 mL). The solution was separated, and the aqueous layer was extracted with CH_2Cl_2 (2 x 30 mL). The organic layers were combined, dried ($MgSO_4$), filtered, and concentrated *in vacuo*. The crude material was purified by flash column chromatography (FCC) to furnish amines **2b-2ab**.

Reaction to form **2k** was also performed in an rbf with a reflux condenser attached and gave exactly the same yield on 0.50 mmol scale.

(*N*-Benzyl-4-phenyl-1,2,3,6-tetrahydropyridin-3-yl)methanol (2a)

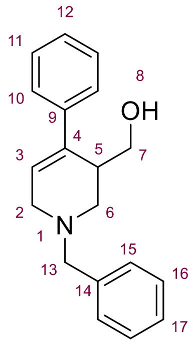

Salt **1a** (187 mg, 0.50 mmol), KI (332 mg, 2.00 mmol), paraformaldehyde (300 mg, 10.0 mmol), and [Ru(*p*-cymene)Cl_2]$_2$ (6.1 mg, 2 mol%) was added to a microwave vial. MeOH (2.08 mL, 0.2 M) and Mg(OMe)$_2$ (0.42mL, 0.89M in MeOH) was added and the solution heated at 65 °C for 16 hours. The solution concentrated under reduce pressure and re-dissolved in CH_2Cl_2 (30 m L), brine (50 mL) and water (50 mL). The solution was separated, and the aqueous layer was extracted with CH_2Cl_2 (2 x 30 mL). The organic layers were combined, dried ($MgSO_4$), filtered, and concentrated *in vacuo*. The product was purified by FCC (10%-20% EtOAc in pentane) to give *amine* **2b** (23 mg, 17%) as an yellow oil. Spectroscopic data was consistent with that reported in the literature.[16]

(*N*-(2-Nitrobenzyl)-4-phenyl-1,2,3,6-tetrahydropyridin-3-yl)methanol (2b)

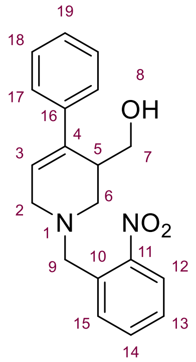

Salt **1b** (209 mg, 0.50 mmol), KI (332 mg, 2.00 mmol), paraformaldehyde (300 mg, 10.0 mmol), and [Ru(*p*-cymene)Cl_2]$_2$ (6.1 mg, 2 mol%) was added to a microwave vial. MeOH (2.08 mL, 0.2 M) and Mg(OMe)$_2$ (0.42mL, 0.89M in MeOH) was added and the solution heated at 65 °C for 16 hours. The solution concentrated under reduce pressure and re-dissolved in CH_2Cl_2 (30 m L), brine (50 mL) and water (50 mL). The solution was separated, and the aqueous layer was extracted with CH_2Cl_2 (2 x 30 mL). The organic layers were combined, dried ($MgSO_4$), filtered, and concentrated *in vacuo*. The product was purified by FCC (20%-50% EtOAc in pentane) to give *amine* **2b** (65 mg, 40%) as an orange solid.

m.p.(EtOAc) 124-126 °C;
HRMS (ESI): Exact mass calculated for $C_{19}H_{21}O_3N_2$ $[M+H]^+$: 325.15467, found: 325.15454;
^{1}H NMR (400 MHz, $CDCl_3$) δ 7.89 (d, *J* = 7.6 Hz, 1H, C^{12}**H**), 7.64 – 7.54 (m, 2H, 2 x ArC**H**), 7.45 (ddd, *J* = 8.6, 6.0, 2.9 Hz, 1H, ArC**H**), 7.41 – 7.19 (m, 5H, 5 x ArC**H**), 6.02 (dd, *J* = 4.7, 2.2 Hz, 1H, C^3**H**), 4.00 (d, *J* = 14.1 Hz, 1H, C^9**H**), 3.88 (d, *J* = 14.0 Hz, 1H, C^9**H₂**), 3.67 (dd, *J* = 10.5, 3.1 Hz, 1H, C^7**H₂**), 3.60 (ddd, *J* = 10.6, 5.2, 1.3 Hz, 1H, C^7**H₂**), 3.38 (dd, *J* = 16.9, 4.7 Hz, 1H, C^2**H₂**), 3.17 (dt, *J* = 11.2, 1.6 Hz, 1H, C^6**H₂**), 2.92 (dt, *J* = 16.9, 2.4 Hz, 1H, C^2**H₂**), 2.87 (br s, 1H, C^5**H**), 2.69 (dd, *J* = 11.5, 3.3 Hz, 1H, C^6**H₂**).
^{13}C NMR (101 MHz, $CDCl_3$) δ 149.9 (ArC), 139.7 (ArC), 136.3 (ArC), 133.0 (ArC), 133.0 (ArCH), 131.4 (ArCH), 128.6 (2 x ArCH), 128.6 (ArCH), 127.5 (ArCH), 126.1 (2 x ArCH), 124.9 (ArCH), 124.1 (C^{3}H), 64.9 (C^{7}H), 59.1 (C^{9}H), 54.9 (C^{6}H), 53.8 (C^{2}H), 39.2 (C^{5}H).
IR (neat) (cm^{-1}): 3311, 2928, 2852, 2811, 1726, 1523, 1350, 1127, 1071, 991.

(N-(2,4-bis(Trifluoromethyl)benzyl)-4-phenyl-1,2,3,6-tetrahydropyridin-3-yl)methanol (2c)

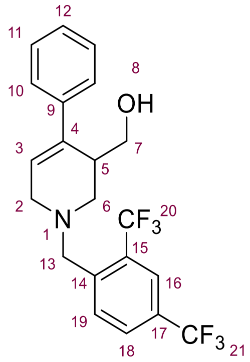

The title compound was prepared by general procedure E using salt **1c** (255 mg, 3.00 mmol). Purification by FCC (10%-30% EtOAc in pentane) gave *amine* **2c** (916 mg, 73%) as an orange oil.
HRMS (ESI): Exact mass calculated for $C_{21}H_{20}F_6NO$ $[M+H]^+$: 416.14436, found: 416.14505
^{1}H NMR (400 MHz, $CDCl_3$) δ 7.93 (d, *J* = 1.8 Hz, 1H, C^{16}**H**), 7.91 (d, *J* = 8.3 Hz, 1H, C^{18}**H**), 7.83 (d, *J* = 8.3, 1.9 Hz, 1H, C^{19}**H**), 7.41 – 7.23 (m, 5H, 5 x Ar**H**), 6.08 (dd, *J* = 4.7, 2.2 Hz, 1H, C^3**H**), 4.02 (s, 1H, O^8**H**), 3.87 (s, 2H, C^{13}**H₂**), 3.75 (dd, *J* = 10.3, 2.7 Hz, 1H, C^7**H₂**), 3.67 (ddd, *J* = 10.3, 4.1, 1.6 Hz, 1H, C^7**H₂**), 3.43 (dd, *J* = 16.9, 4.8 Hz, 1H, C^2**H₂**), 3.16 (dt, *J* = 11.2, 1.6 Hz, 1H, C^6**H₂**), 2.98 (dt, *J* = 16.9, 2.5 Hz, 1H, C^2**H₂**), 2.89 (brs, 1H, C^5**H**), 2.78 (ddd, *J* = 11.1, 3.9, 1.5 Hz, 1H, C^6**H₂**).
^{13}C NMR (101 MHz, $CDCl_3$) δ 141.2 (C^{14}), 139.7 (C), 136.4 (C), 131.5 (C^{19}H), 129.8 (q, *J* = 31.7 Hz, C^{15}+C^{17}), 129.0 (C^{18}H), 128.7 (2 x ArCH), 127.7 (ArCH), 126.2 (2 x ArCH), 124.1 (C^{3}H), 123.7 (q, *J* = 274.0 Hz, C^{20}F₃/C^{21}F₃), 123.5 (q, *J* = 272.4 Hz, C^{20}F₃/C^{21}F₃), 123.5 – 123.2 (m, C^{16}H), 65.4 (C^{7}H₂), 58.0 (q, *J* = 2.2 Hz, C^{13}H₂), 55.7 (C^{6}H₂), 53.8 (C^{2}H₂), 39.1 (C^{5}H).
^{19}F NMR (376 MHz, $CDCl_3$) δ –59.20, –62.86.
IR (neat) (cm^{-1}): 3376, 2996, 1346, 1283, 1276, 1171, 1126, 1085, 1037, 918.

(N-(3,5-bis(Trifluoromethyl)benzyl)-4-phenyl-1,2,3,6-tetrahydropyridin-3-yl)methanol (2d)

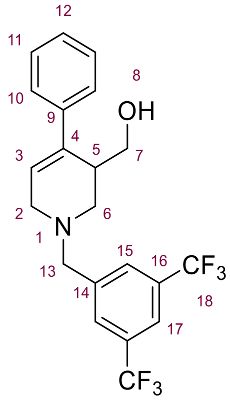

The title compound was prepared by general procedure E using salt **1d** (255 mg, 0.50 mmol). Purification by FCC (20% EtOAc in pentane) gave *amine* **2d** (66 mg, 32%) as an orange oil.

HRMS (ESI): Exact mass calculated for $C_{21}H_{20}F_6NO$ $[M+H]^+$: 416.14436, found: 416.14499

^{1}H NMR (400 MHz, $CDCl_3$) δ 7.83 (s, 3H), 7.40 – 7.23 (m, 5H), 6.06 (dd, *J* = 4.7, 2.2 Hz, 1H), 3.84 (s, 1H), 3.81 (d, *J* = 13.5 Hz, 1H), 3.75 (dd, *J* = 10.2, 2.8 Hz, 1H), 3.73 (d, *J* = 13.5 Hz, 1H), 3.67 (ddd, *J* = 10.3, 4.3, 1.5 Hz, 1H), 3.42 – 3.32 (m, 1H), 3.17 (dt, *J* = 11.1, 1.6 Hz, 1H), 2.94 (dt, *J* = 16.6, 2.4 Hz, 1H), 2.93 – 2.86 (m, 1H), 2.72 (ddd, *J* = 11.1, 3.8, 1.4 Hz, 1H).

^{13}C NMR (101 MHz, $CDCl_3$) δ 140.4 (C^{14}), 139.7 (C), 136.6 (C), 132.0 (q, *J* = 33.3 Hz, 2 x C^{16}), 129.1 (2 x C^{15}H), 128.7 (2 x ArCH), 127.7 (ArCH), 126.2 (2 x ArCH), 124.0 (C^3H), 123.5 (q, *J* = 277.6 Hz, 2 x $C^{18}F_3$), 121.9 – 121.5 (m, C^{17}H), 65.3 (C^7H_2), 62.0 ($C^{13}H_2$), 55.4 (C^6H_2), 53.5 (C^2H_2), 39.0 (C^5H).

^{19}F NMR (377 MHz, $CDCl_3$) δ –62.79 (d, *J* = 2.6 Hz).

IR (neat) (cm^{-1}): 3376, 2921, 1352, 1276, 1168, 1124, 846, 756, 699, 682.

(*N*-(2,4-bis(Trifluoromethyl)benzyl)-4-(4-(tert-butyl)phenyl)-1,2,3,6-tetrahydropyridin-3-yl)methanol (2e)

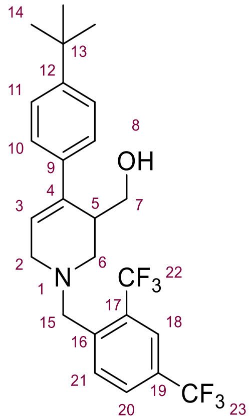

The title compound was prepared by General procedure E using salt **1e** (283 mg, 0.50 mmol). Purification by FCC (10%-30% EtOAc in pentane) gave *amine* **2e** (157 mg, 66%) as an white solid.

m.p. (acetone): 66-68°C

HRMS (ESI): Exact mass calculated for $C_{25}H_{28}F_6NO$ $[M+H]^+$: 472.20696, found: 472.20642

^{1}H NMR (400 MHz, $CDCl_3$) δ 7.95 – 7.88 (m, 2H, C^{18}**H** + C^{21}**H**), 7.83 (dd, *J* = 8.3, 2.4 Hz, 1H, C^{20}**H**), 7.36 (d, *J* = 8.7 Hz, 2H, 2 x C^{10}**H**), 7.30 (d, *J* = 8.7 Hz, 2H, 2 x C^{11}**H**), 6.06 (dd, *J* = 4.8, 2.2 Hz, 1H, C^3**H**), 4.02 (s, 1H, O**H**), 3.86 (s, 2H, C^{15}**H₂**), 3.75 (dd, *J* = 10.3, 2.7 Hz, 1H, C^7**H₂**), 3.69 (ddd, *J* = 10.3, 4.1, 1.5 Hz, 1H, C^7**H₂**), 3.42 (dd, *J* = 16.9, 4.7 Hz, 1H, C^2**H₂**), 3.15 (dt, *J* = 11.1, 1.6 Hz, 1H, C^6**H₂**), 2.97 (dt, *J* = 16.9, 2.5 Hz, 1H, C^2**H₂**), 2.89 (s, 1H, C^5**H**), 2.76 (ddd, *J* = 11.1, 3.9, 1.4 Hz, 1H, C^6**H₂**), 1.32 (s, 9H, 3 x C^{14}**H₃**).

^{13}C NMR (126 MHz, $CDCl_3$) δ 150.7 (C^{12}), 141.2 (C), 136.5 (C), 136.0 (C), 131.5 (C^{21}H), 130.0 (q, *J* = 33.4 Hz, C^{17}/C^{19}), 129.7 (q, *J* = 31.2 Hz, C^{17}/C^{19}), 129.1 (q, *J* = 3.8 Hz, C^{20}H), 125.8 (2 x C^{11}H), 125.6 (2 x C^{10}H), 123.7 (q, *J* = 274.2 Hz, $C^{22}F_3/C^{23}F_3$), 123.5 (q, *J* = 272.3 Hz, $C^{22}F_3/C^{23}F_3$), 123.4 (C^3H), 123.4 – 123.2 (m, C^{18}H), 65.5 (C^7H_2), 58.0 (q, *J* = 2.2 Hz, C^{15}H), 55.8 (C^6H_2), 53.8 (C^2H_2), 38.8 (C^5H), 34.6 (C^{13}), 31.5 (3 x $C^{14}H_3$).

^{19}F NMR (377 MHz, $CDCl_3$) δ –59.2, –62.9.

IR (neat) (cm^{-1}):3036, 2830, 1344, 1273, 1171, 1121, 1083, 1055, 827, 673.

(N-(2,4-bis(Trifluoromethyl)benzyl)-4-(4-methoxyphenyl)-1,2,3,6-tetrahydropyridin-3-yl)methanol (2f)

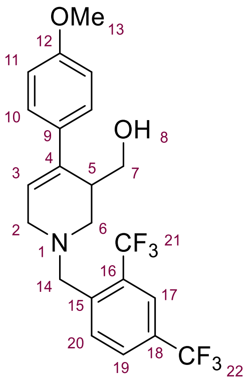

The title compound was prepared by General procedure E using salt **1f** (270 mg, 0.50 mmol). Purification by FCC (5%-30% EtOAc in pentane) gave *amine* **2f** (154 mg, 69%) as an orange oil.

HRMS (ESI): Exact mass calculated for $C_{22}H_{22}F_6NO_2$ $[M+H]^+$446.1549:, found: 446.1549

^{1}H NMR (400 MHz, $CDCl_3$) δ 7.93 (s, 1H, C^{17}**H**), 7.90 (d, *J* = 8.2 Hz, 1H, C^{20}**H**), 7.83 (dd, *J* = 8.3, 1.9 Hz, 1H, C^{19}**H**), 7.30 (d, *J* = 8.9 Hz, 2H, 2 x C^{10}**H**), 6.87 (d, *J* = 8.8 Hz, 2H, 2 x C^{11}**H**), 6.00 (dd, *J* = 4.6, 2.2 Hz, 1H, C^{3}**H**), 4.06 (s, 1H, O**H**), 3.86 (s, 2H, C^{14}**H$_2$**), 3.81 (s, 3H, C^{13}**H$_3$**), 3.75 (dd, *J* = 10.2, 2.7 Hz, 1H, C^{7}**H$_2$**), 3.67 (ddd, *J* = 10.2, 4.0, 1.6 Hz, 1H, C^{7}**H$_2$**), 3.41 (dd, *J* = 16.8, 4.7 Hz, 1H, C^{2}**H$_2$**), 3.14 (dt, *J* = 11.1, 1.6 Hz, 1H, C^{6}**H$_2$**), 2.96 (dt, *J* = 16.8, 2.5 Hz, 1H, C^{2}**H$_2$**), 2.85 (s, 1H, C^{5}**H**), 2.76 (ddd, *J* = 11.1, 3.8, 1.5 Hz, 1H, C^{6}**H$_2$**).

^{13}C NMR (126 MHz, $CDCl_3$) δ 159.3 (C^{12}), 141.2 (C^{15}), 135.7 (C), 132.1 (C), 131.5 (C^{20}H), 130.1 (q, *J* = 33.5 Hz, C^{16}/C^{18}), 129.7 (q, *J* = 31.2 Hz, C^{16}/C^{18}), 129.0 (q, *J* = 4.1 Hz, C^{19}H), 127.2 (2 x C^{10}H), 123.7 (q, *J* = 273.2 Hz, C^{21}F$_3$/C^{22}F$_3$), 123.5 (q, *J* = 272.3 Hz, C^{21}F$_3$/C^{22}F$_3$), 123.3 (m, C^{17}H), 122.6 (C^{3}H), 114.0 (2 x C^{11}H), 65.5 (C^{7}H$_2$), 58.0 (C^{14}H$_2$), 55.8 (C^{6}H$_2$), 55.4 (C^{13}H$_3$), 53.8 (C^{2}H$_2$), 38.9 (C^{5}H).

^{19}F NMR (377 MHz, $CDCl_3$) δ –59.2, –62.8.

IR (neat) (cm^{-1}): 2981, 1640, 1599, 1344, 1271, 1166, 1122, 1084, 1053, 833, 671.

(*N*-(2,4-bis(Trifluoromethyl)benzyl)-4-(4-(trifluoromethyl)phenyl)-1,2,3,6-tetrahydropyridin-3-yl)methanol (2g)

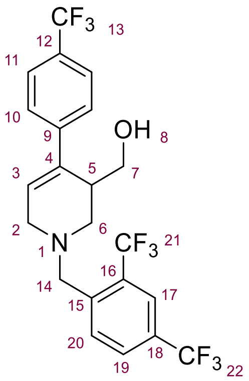

The title compound was prepared by General procedure E using salt **1g** (289 mg, 0.50 mmol). Purification by FCC (10%-20% EtOAc in pentane) gave *amine* **2g** (112 mg, 46%) as a yellow oil.

HRMS (ESI): Exact mass calculated for $C_{22}H_{19}F_9NO$ $[M+H]^+$: 484.1317, found: 484.1321

^{1}H NMR (500 MHz, $CDCl_3$) δ 7.94 (s, 1H, C^{17}**H**), 7.88 (d, *J* = 8.2 Hz, 1H, C^{20}**H**), 7.84 (dd, *J* = 8.4, 1.9 Hz, 1H, C^{19}**H**), 7.59 (d, *J* = 8.2 Hz, 2H, 2 x C^{11}**H**), 7.47 (d, *J* = 8.1 Hz, 2H, 2 x C^{10}**H**), 6.15 (dd, *J* = 4.7, 2.2 Hz, 1H, C^{3}**H**), 3.96 (s, 1H, O**H**), 3.87 (s, 2H, C^{14}**H$_2$**), 3.74 (dd, *J* = 10.4, 2.6 Hz, 1H, C^{7}**H$_2$**), 3.63 (ddd, *J* = 10.4, 4.1, 1.6 Hz, 1H, C^{7}**H$_2$**), 3.45 (dd, *J* = 17.3, 4.8 Hz, 1H, C^{2}**H$_2$**), 3.17 (dt, *J* = 11.2, 1.5 Hz, 1H, C^{6}**H$_2$**), 3.00 (dt, *J* = 17.2, 2.5 Hz, 1H, C^{2}**H$_2$**), 2.89 (brs, 1H, C^{5}**H**), 2.78 (ddd, *J* = 11.1, 3.8, 1.6 Hz, 1H, C^{6}**H$_2$**).

^{13}C NMR (126 MHz, $CDCl_3$) δ 143.2 (C), 140.9 (C), 135.5 (C), 131.5 (C^{20}H), 130.2 (q, *J* = 33.2 Hz, C^{12}/C^{16}/C^{18}), 129.8 (q, *J* = 31.3 Hz, C^{12}/C^{16}/C^{18}), 129.7 (q, *J* = 32.4 Hz, C^{12}/C^{16}/C^{18}), 129.1 (q, *J* = 3.9 Hz, C^{19}H), 126.5 (2 x C^{10}H), 126.2 (C^{3}H), 125.7 (q, *J* = 3.8 Hz, 2 x C^{11}H), 124.3 (q, *J* = 272.0 Hz, $C^{13}F_3$/$C^{21}F_3$/$C^{22}F_3$), 123.7 (q, *J* = 274.3 Hz, $C^{13}F_3$/$C^{21}F_3$/$C^{22}F_3$), 123.5 (q, *J* = 272.3 Hz, $C^{13}F_3$/$C^{21}F_3$/$C^{22}F_3$), 123.5 – 123.4 (m, C^{17}H), 65.2 ($C^{7}H_2$), 57.9 ($C^{14}H_2$), 55.6 ($C^{6}H_2$), 53.7 ($C^{2}H_2$), 38.9 (C^{5}H).
^{19}F NMR (377 MHz, $CDCl_3$) δ –59.2, –62.5, –62.9.
IR (neat) (cm^{-1}): 3320, 1346, 1325, 1275, 1166, 1116, 1083, 1069, 1055, 672.

Methyl-4-(1-(2,4-bis(trifluoromethyl)benzyl)-3-(hydroxymethyl)-1,2,3,6-tetrahydropyridin-4-yl)benzoate (2h)

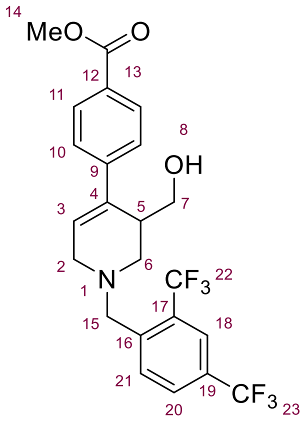

The title compound was prepared by General procedure E using salt **1h** (284 mg, 0.50 mmol). Purification by FCC (10-30% EtOAc in pentane) gave *amine* **2h** (148 mg, 62%) as an off-white solid.

m.p. (acetone): 145-147 °C

HRMS (ESI): Exact mass calculated for $C_{23}H_{22}F_6NO$ $[M+H]^+$: 474.1498, found:474.1501

^{1}H NMR (400 MHz, $CDCl_3$) δ 8.01 (d, *J* = 8.5 Hz, 2H, 2 x C^{11}**H**), 7.94 (d, *J* = 1.9 Hz, 1H, C^{18}**H**), 7.89 (d, *J* = 8.2 Hz, 1H, C^{21}**H**), 7.83 (dd, *J* = 8.3, 1.8 Hz, 1H, C^{20}**H**), 7.44 (d, *J* = 8.5 Hz, 2H, 2 x C^{10}**H**), 6.19 (dd, *J* = 4.7, 2.3 Hz, 1H, C^{3}**H**), 3.96 (s, 1H, O**H**), 3.91 (s, 3H, C^{14}**H₃**), 3.87 (s, 2H, C^{15}**H₂**), 3.75 (dd, *J* = 10.4, 2.6 Hz, 1H, C^{7}**H₂**), 3.64 (ddd, *J* = 10.4, 4.2, 1.6 Hz, 1H, C^{7}**H₂**), 3.45 (dd, *J* = 17.3, 4.7 Hz, 1H, C^{2}**H₂**), 3.17 (dt, *J* = 11.2, 1.5 Hz, 1H, C^{6}**H₂**), 2.99 (dt, *J* = 17.3, 2.5 Hz, 1H, C^{2}**H₂**), 2.92 (s, 1H, C^{5}**H**), 2.78 (ddd, *J* = 11.3, 3.9, 1.5 Hz, 1H, C^{6}**H₂**).

^{13}C NMR (126 MHz, $CDCl_3$) δ 167.0 (C^{13}O), 144.1 (C), 141.0 (C), 135.7 (C), 131.5 (C^{21}H), 130.2 (q, *J* = 33.5 Hz, C^{17}/C^{19}), 130.0 (2 x C^{11}H), 129.8 (q, *J* = 31.0 Hz, C^{17}/C^{19}), 129.3 (C), 129.1 (q, *J* = 3.8 Hz, C^{20}H), 126.1 (2 x C^{10}H + C^{3}H), 123.7 (q, *J* = 274.3 Hz, $C^{22}F_3$/$C^{23}F_3$), 123.5 (q, *J* = 272.3 Hz, $C^{22}F_3$/$C^{23}F_3$), 123.5 – 123.2 (m, C^{18}H), 65.3 ($C^{7}H_2$), 57.9 (q, *J* = 2.0 Hz, $C^{15}H_2$), 55.6 ($C^{6}H_2$), 53.8 ($C^{2}H_2$), 52.3 ($C^{14}H_3$), 38.8 (C^{5}H).
^{19}F NMR (376 MHz, $CDCl_3$) δ –59.2, –62.8.
IR (neat) (cm^{-1}): 3522,1709, 1630, 1607, 1344, 1283, 1266, 1176, 1170, 1127.

(N-(2,4-bis(Trifluoromethyl)benzyl)-4-(4-(methylsulfonyl)phenyl)-1,2,3,6-tetrahydropyridin-3-yl)methanol (2i)

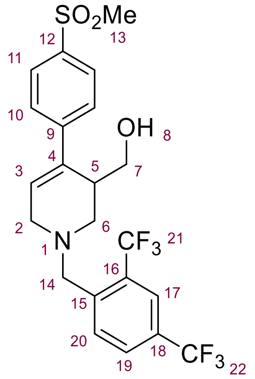

The title compound was prepared by General procedure E using salt **1i** (294 mg, 0.50 mmol). Purification my FCC (10%-50% EtOAc in pentane) gave *amine* **2i** (136 mg, 55%) as a yellow solid.

m.p. (EtOAc): 95-97 °C

HRMS (ESI): Exact mass calculated for $C_{22}H_{22}F_6NOS$ $[M+H]^+$: 494.1195, found: 494.1208

^{1}H NMR (500 MHz, $CDCl_3$) δ 7.94 (d, *J* = 1.8 Hz, 1H, C^{17}**H**), 7.91 (d, *J* = 8.4 Hz, 2H, 2 x C^{11}**H**), 7.87 (d, *J* = 8.2 Hz, 1H, C^{20}**H**), 7.84 (dd, *J* = 8.2, 1.8 Hz, 1H, C^{19}**H**), 7.55 (d, *J* = 8.5 Hz, 2H, 2 x C^{10}**H**), 6.21 (dd, *J* = 4.8, 2.2 Hz, 1H, C^3**H**), 3.92 (s, 1H, O**H**), 3.88 (s, 2H, C^{14}**H_2**), 3.74 (dd, *J* = 10.4, 2.6 Hz, 1H, C^7**H_2**), 3.62 (ddd, *J* = 10.5, 4.3, 1.6 Hz, 1H, C^7**H_2**), 3.47 (dd, *J* = 17.4, 4.8 Hz, 1H, C^2**H_2**), 3.18 (dt, *J* = 11.2, 1.5 Hz, 1H, C^6**H_2**), 3.06 (s, 3H, C^{13}**H_3**), 3.01 (dt, *J* = 17.5, 2.5 Hz, 1H, C^2**H_2**), 2.90 (s, 1H, C^5**H**), 2.78 (ddd, *J* = 11.2, 3.9, 1.5 Hz, 1H, C^6**H_2**).

^{13}C NMR (126 MHz, $CDCl_3$) δ 145.2 (C), 140.8 (C), 139.4 (C), 135.2 (C), 131.5 (C^{20}H), 130.3 (q, *J* = 33.6 Hz, C^{16}/C^{18}), 129.8 (q, *J* = 31.1 Hz, C^{16}/C^{18}), 129.1 (q, *J* = 3.8 Hz, C^{19}H), 127.9 (2 x C^{11}H), 127.4 (C^3H), 127.1 (2 x C^{10}H), 123.7 (q, *J* = 274.2 Hz, $C^{21}F_3$/$C^{22}F_3$), 123.6 – 123.2 (m, C^{17}H), 123.5 (q, *J* = 272.3 Hz, $C^{21}F_3$/$C^{22}F_3$), 65.2 (C^7H_2), 57.9 (q, *J* = 2.0 Hz, $C^{14}H_2$), 55.5 (C^6H_2), 53.7 (C^2H_2), 44.7 ($C^{13}H_3$), 38.9 (C^5H).

^{19}F NMR (377 MHz, $CDCl_3$) δ –59.1, –62.9.

IR (neat) (cm^{-1}): 3326, 2927, 1620, 1346, 1301, 1275, 1171, 1124, 1084, 1055.

(*N*-(2,4-bis(Trifluoromethyl)benzyl)-4-(4-fluorophenyl)-1,2,3,6-tetrahydropyridin-3-yl)methanol (2j)

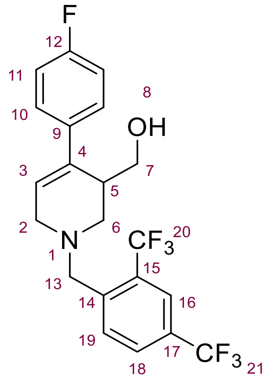

The title compound was prepared by General procedure E using salt **1j** (264 mg, 0.50 mmol). Purification by FCC (5%-20% EtOAc in pentane) gave *amine* **2j** (169 mg, 78%) as a yellow oil.

HRMS (ESI): Exact mass calculated for $C_{21}H_{19}F_7NO$ $[M+H]^+$: 434.1349, found: 434.1349

^{1}H NMR (500 MHz, $CDCl_3$) δ 7.93 (d, *J* = 1.9 Hz, 1H, C^{16}**H**), 7.88 (d, *J* = 8.2 Hz, 1H, C^{19}**H**), 7.83 (d, *J* = 8.2 Hz, 1H, C^{18}**H**), 7.37 – 7.28 (m, 2H, 2 x C^{10}**H**), 7.07 – 6.98 (m, 2H, 2 x C^{11}**H**), 6.02 (dd, *J* = 4.8, 2.2 Hz, 1H, C^3**H**), 4.08 (s, 1H, O**H**), 3.86 (s, 2H, C^{13}**H_2**), 3.74 (dd, *J* = 10.4, 2.6 Hz, 1H, C^7**H_2**), 3.63 (ddd, *J* = 10.4, 4.0, 1.7 Hz, 1H, C^7**H_2**), 3.41 (dd, *J* = 17.0, 4.8 Hz, 1H, C^2**H**), 3.15 (dt, *J* = 11.2, 1.5 Hz, 1H, C^6**H**), 2.96 (dt, *J* = 17.0, 2.5 Hz, 1H, C^2**H**), 2.83 (s, 1H, C^5**H**), 2.77 (ddd, *J* = 11.1, 3.8, 1.6 Hz, 1H, C^6**H**).

^{13}C NMR (126 MHz, $CDCl_3$) δ 162.5 (d, *J* = 246.5 Hz, C^{12}F), 141.0 (C), 135.7 (d, *J* = 3.3 Hz, C^{9}), 135.5 (C), 131.5 (C^{19}H) 130.1 (q, *J* = 33.6 Hz, C^{15}/C^{17}), 129.8 (q, *J* = 31.0 Hz, C^{15}/C^{17}), 129.1 (q, *J* = 3.7 Hz, C^{18}H), 127.8 (d, *J* = 8.0 Hz, 2 x C^{10}H), 124.1 (C^{3}H), 123.7 (q, *J* = 274.2 Hz, C^{20}F$_3$/C^{21}F$_3$), 123.5 (q, *J* = 272.3 Hz, C^{20}F$_3$/C^{21}F$_3$), 123.4 (dd, *J* = 6.2, 3.8 Hz, C^{16}H), 115.5 (d, *J* = 21.3 Hz, 2 x C^{11}H), 65.3 (C^{7}H$_2$), 58.0 (q, *J* = 2.0 Hz, C^{13}H$_2$), 55.7 (C^{6}H$_2$), 53.7 (C^{2}H$_2$), 39.1 (C^{5}H).
IR (neat) (cm^{-1}): 3279, 2922, 1509, 1345, 1275, 1165, 1122, 1083, 1055, 841.

(*N*-(2,4-bis(Trifluoromethyl)benzyl)-4-(4-(trifluoromethoxy)phenyl)-1,2,3,6-tetrahydropyridin-3-yl)methanol (2k)

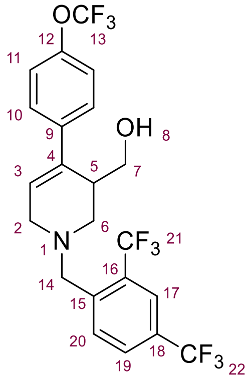

The title compound was prepared by General procedure E using salt **1k** (297 mg, 0.50 mmo). Purification by FCC (5%-20% EtOAc in pentane) gave *amine* **2k** (162 mg, 64%) as a yellow oil.
HRMS (ESI): Exact mass calculated for $C_{22}H_{19}F_9NO_2$ [M+H]$^+$: 500.1267, found: 500.1259
^{1}H NMR (400 MHz, $CDCl_3$) δ 7.93 (s, 1H, C^{17}**H**), 7.89 (d, *J* = 8.2 Hz, 1H, C^{20}**H**), 7.83 (dd, *J* = 8.2, 1.8 Hz, 1H, C^{19}**H**), 7.38 (d, *J* = 8.8 Hz, 2H, 2 x C^{10}**H**), 7.18 (dd, *J* = 8.9, 1.0 Hz, 2H, C^{11}**H**), 6.10 – 6.04 (m, 1H, C^{3}**H**), 4.00 (s, 1H, O**H**), 3.86 (s, 2H, C^{14}**H$_2$**), 3.74 (dd, *J* = 10.4, 2.6 Hz, 1H, C^{7}**H$_2$**), 3.64 (ddd, *J* = 10.4, 4.1, 1.7 Hz, 1H, C^{7}**H$_2$**), 3.43 (dd, *J* = 17.1, 4.7 Hz, 1H, C^{2}**H$_2$**), 3.16 (dt, *J* = 11.1, 1.5 Hz, 1H, C^{6}**H$_2$**), 2.97 (dt, *J* = 17.1, 2.4 Hz, 1H, C^{2}**H$_2$**), 2.84 (s, 1H, C^{5}**H**), 2.77 (ddd, *J* = 11.1, 3.9, 1.5 Hz, 1H, C^{6}**H$_2$**).
^{13}C NMR (126 MHz, $CDCl_3$) δ 148.7 (q, *J* = 2.0 Hz, C^{12}), 141.0 (C), 138.4 (C), 135.3 (C), 131.5 (C^{20}H), 130.2 (q, *J* = 33.6 Hz, C^{16}/C^{18}), 129.8 (q, *J* = 31.1 Hz, C^{16}/C^{18}), 129.1 (q, *J* = 3.5 Hz, C^{19}H), 127.6 (2 x C^{10}H), 125.0 (C^{3}H), 123.7 (q, *J* = 274.2 Hz, C^{21}F$_3$/C^{22}F$_3$), 123.5 (q, *J* = 272.3 Hz, C^{21}F$_3$/C^{22}F$_3$), 123.5 – 123.3 (m, C^{17}H), 121.1 (2 x C^{11}H), 120.6 (q, *J* = 257.1 Hz, C^{13}F$_3$), 65.3 (C^{7}H$_2$), 57.9 (q, *J* = 2.3 Hz, C^{14}H$_2$), 55.7, (C^{6}H$_2$), 53.7 (C^{2}H$_2$), 39.0 (C^{5}H).
^{19}F NMR (377 MHz, $CDCl_3$) δ –57.9, –59.2, –62.7.
IR (neat) (cm^{-1}): 3245, 2922, 1345, 1275, 1170, 1123, 1083, 1055, 804, 673.

(N-(2,4-bis(Trifluoromethyl)benzyl)-4-(3,5-difluorophenyl)-1,2,3,6-tetrahydropyridin-3-yl)methanol (2l)

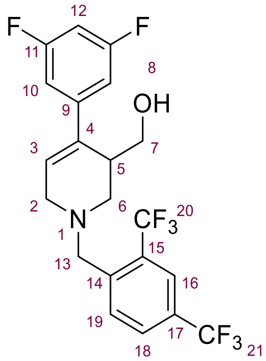

The title compound was prepared by General procedure E using salt **1l** (273 mg, 0.50 mmol). Purification by FCC (5%-15% EtOAc in pentane) gave *amine* **2l** (132 mg, 59%) as a pale orange oil.

HRMS (ESI): Exact mass calculated for $C_{21}H_{18}F_8NO$ $[M+H]^+$: 452.1255, found: 452.1247

^{1}H NMR (400 MHz, $CDCl_3$) δ 7.94 (s, 1H, $C^{16}H$), 7.91 – 7.79 (m, 2H, $C^{18}H$ + $C^{19}H$), 6.93 – 6.84 (m, 2H, 2 x $C^{10}H$), 6.72 (tt, *J* = 8.8, 2.3 Hz, 1H, $C^{12}H$), 6.13 (dd, *J* = 4.8, 2.3 Hz, 1H, C^3H), 3.86 (s, 2H, $C^{13}H_2$), 3.75 (dd, *J* = 10.4, 2.6 Hz, 1H, C^7H_2), 3.65 (ddd, *J* = 10.4, 4.1, 1.7 Hz, 1H, C^7H_2), 3.43 (dd, *J* = 17.3, 4.7 Hz, 1H, C^2H_2), 3.16 (dt, *J* = 10.9, 1.4 Hz, 1H, C^6H_2), 2.98 (dt, *J* = 17.4, 2.4 Hz, 1H, C^2H_2), 2.83 – 2.70 (m, 2H, C^6H_2 + C^5H).

^{13}C NMR (126 MHz, $CDCl_3$) δ 163.32 (dd, *J* = 247.9, 13.3 Hz, 2 x $C^{11}F$), 143.1 (t, *J* = 9.2 Hz, C^9), 140.8 (C^{14}), 134.7 (t, *J* = 2.5 Hz, C^4), 131.5 ($C^{19}H$), 130.2 (q, *J* = 33.5 Hz, C^{15}/C^{17}), 129.8 (q, *J* = 31.2 Hz, C^{15}/C^{17}), 129.1 (q, *J* = 3.8 Hz, $C^{18}H$), 126.2 (C^3H), 123.7 (q, *J* = 274.2 Hz, $C^{20}F_3$/$C^{21}F_3$), 123.5 (q, *J* = 272.4 Hz, $C^{20}F_3$/$C^{21}F_3$), 123.7 – 123.2 (m, $C^{16}H$), 109.5 – 108.5 (m, 2 x $C^{10}H$), 102.9 (t, *J* = 25.4 Hz, $C^{12}H$), 65.2 (C^7H_2), 57.9 (q, *J* = 2.0 Hz, $C^{13}H_2$), 55.4 (C^6H_2), 53.6 (C^2H_2), 38.8 (C^5H).

^{19}F NMR (377 MHz, $CDCl_3$) δ –59.2, –62.9, –109.9 (t, *J* = 8.7 Hz).

IR (neat) (cm^{-1}): 3264, 2972, 2848, 1345, 1275, 1171, 1118, 1083, 1055, 988.

(N-(2,4-bis(Trifluoromethyl)benzyl)-4-(3,4,5-trichlorophenyl)-1,2,3,6-tetrahydropyridin-3-yl)methanol (2m)

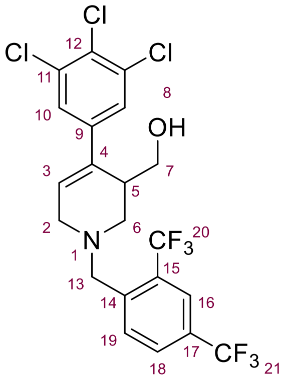

The title compound was prepared by General procedure E using salt **1m** (287 mg, 0.47 mmol). Purification my FCC (5%-20% EtOAc in pentane) gave the *amine* **2m** (124 mg, 51%) as an orange solid.

m.p. (EtOAc): 66-68 °C

HRMS (ESI): Exact mass calculated for $C_{21}H_{17}Cl_3F_6NO$ $[M+H]^+$: 518.0274, found: 518.0274

^{1}H NMR (400 MHz, $CDCl_3$) δ 7.94 (s, 1H, $C^{16}H$), 7.89 – 7.79 (m, 2H, $C^{18}H$ + $C^{19}H$), 7.37 (s, 2H, 2 x $C^{10}H$), 6.11 (dd, *J* = 4.7, 2.3 Hz, 1H, C^3H), 3.86 (s, 2H, $C^{16}H_2$), 3.74 (dd, *J* = 10.4, 2.4 Hz, 1H, C^7H_2), 3.62 (ddd, *J* = 10.5, 4.0, 1.8 Hz, 1H, C^7H_2), 3.43 (dd, *J* = 17.3, 4.7 Hz, 1H, C^2H_2), 3.20 – 3.10 (m, 1H, C^6H_2), 2.97 (dt, *J* = 17.4, 2.4 Hz, 1H, C^2H_2), 2.80 – 2.68 (m, 2H, C^6H_2 + C^5H).

^{13}C NMR (126 MHz, $CDCl_3$) δ 140.7 (C^{14}), 139.8 (C), 134.5 (2 x C^{11}), 133.9 (C), 131.5 ($C^{19}H$), 130.3 (C), 130.3 (q, *J* = 33.6 Hz, C^{15}/ C^{17}), 129.8 (q, *J* = 31.2 Hz, C^{15}/ C^{17}), 129.1 (q, *J* = 3.8 Hz, $C^{18}H$), 126.8 (C^3H), 126.5 (2 x $C^{10}H$), 123.7 (d, *J* = 274.3 Hz, $C^{20}F_3$/ $C^{21}F_3$), 123.7 – 123.2 (m, $C^{16}H$), 123.4 (d, *J* = 272.3 Hz, $C^{20}F_3$/ $C^{21}F_3$), 65.1 (C^7H_2), 57.9 (q, *J* = 2.4 Hz, $C^{13}H_2$), 55.4 (C^6H_2), 53.6 (C^2H_2), 38.8 (C^5H).

^{19}F NMR (377 MHz, $CDCl_3$) δ –59.2, –62.9.

IR (neat) (cm^{-1}): 3260, 2981, 1640, 1345, 1269, 1169, 1119, 1084, 1054, 762

(*N*-(2,4-bis(Trifluoromethyl)benzyl)-4-(o-tolyl)-1,2,3,6-tetrahydropyridin-3-yl)methanol (2n)

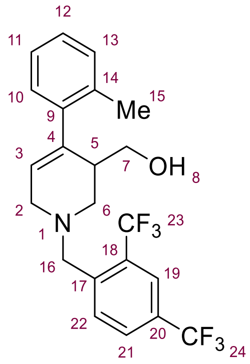

The title compound was prepared by General procedure E using salt **1n** (262 mg, 0.50 mmol). Purification by FCC (5%-20% EtOAc in pentane) gave amine **2n** (144 mg, 67%) as a yellow oil.

HRMS (ESI): Exact mass calculated for $C_{22}H_{22}F_6NO$ $[M+H]^+$: 430.1600, found: 430.1605

^{1}H NMR (400 MHz, $CDCl_3$) δ 7.88 – 7.81 (m, 2H, C^{19}**H** + C^{22}**H**), 7.77 (dd, *J* = 8.2, 1.9 Hz, 1H, C^{21}**H**), 7.14 – 7.02 (m, 4H, 4 x Ar**H**), 5.63 – 5.57 (m, 1H, C^3**H**), 3.80 (s, 2H, C^{16}**H$_2$**), 3.61 (dd, *J* = 10.4, 2.7 Hz, 1H, C^7**H$_2$**), 3.52 (ddd, *J* = 10.4, 3.6, 1.8 Hz, 1H, C^7**H$_2$**), 3.32 (dd, *J* = 16.4, 4.5 Hz, 1H, C^2**H$_2$**), 3.03 (dt, *J* = 11.1, 1.6 Hz, 1H, C^6**H$_2$**), 2.87 (dt, *J* = 16.4, 2.4 Hz, 1H, C^2**H$_2$**), 2.77 (ddd, *J* = 11.2, 4.0, 1.6 Hz, 1H, C^6**H$_2$**), 2.41 (s, 1H, C^5**H**), 2.23 (s, 3H, C^{15}**H$_3$**).

^{13}C NMR (126 MHz, $CDCl_3$) δ 141.1 (C), 141.0 (C), 137.4 (C), 135.8 (C), 131.6 (C^{22}H), 130.3 (ArCH), 130.1 (q, *J* = 33.5 Hz, C^{18}/C^{20}), 129.8 (q, *J* = 31.1 Hz, C^{18}/C^{20}), 129.1 (C^{21}H), 129.1 (ArCH), 127.3 (ArCH), 125.7 (ArCH), 125.3 (C^{3}H), 123.7 (q, *J* = 274.2 Hz, C^{23}F$_3$/ C^{24}F$_3$), 123.5 (q, *J* = 272.3 Hz, C^{23}F$_3$/ C^{24}F$_3$), 123.4 – 123.2 (m, C^{19}H), 65.0 (C^{7}H$_2$), 58.1 (q, *J* = 2.4 Hz, C^{16}H$_2$), 55.9 (C^{6}H$_2$), 53.3 (C^{2}H$_2$), 41.4 (C^{5}H), 19.8 (C^{15}H$_3$).

^{19}F NMR (377 MHz, $CDCl_3$) δ –59.1, –62.9.

IR (neat) (cm^{-1}): 3658, 2980, 2973, 1345, 1274, 1169, 1123, 1083, 1055, 760.

(*N*-(2,4-bis(Trifluoromethyl)benzyl)-4-(3,5-dimethoxyphenyl)-1,2,3,6-tetrahydropyridin-3-yl)methanol (2o)

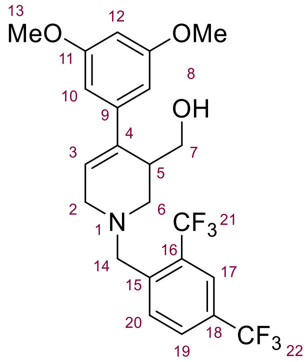

The title compound was prepared by General procedure E using salt **1o** (285 mg, 0.50 mmol). Purification by FCC (10%-30% EtOAc in pentane) gave the *amine* **2o** (186 mg, 78%) as a viscous red oil.

HRMS (ESI): Exact mass calculated for $C_{23}H_{24}F_6NO_3$ $[M+H]^+$: 476.1655, found: 476.1653

^{1}H NMR (500 MHz, $CDCl_3$) δ 7.93 (s, 1H, C^{17}**H**), 7.89 (d, *J* = 8.2 Hz, 1H, C^{19}**H**), 7.83 (dd, *J* = 8.2, 1.9 Hz, 1H, C^{20}**H**), 6.50 (d, *J* = 2.2 Hz, 2H, 2 x C^{10}**H**), 6.40 (t, *J* = 2.2 Hz, 1H, C^{12}**H**), 6.07 (dd, *J* = 4.8, 2.2 Hz, 1H, C^3**H**), 4.07 (s, 1H, **OH**), 3.85 (s, 2H, C^{14}**H**), 3.79 (s, 6H, 2 x C^{13}**H$_3$**), 3.74 (dd, *J* = 10.3, 2.7 Hz, 1H, C^7**H$_2$**), 3.67 (ddd, *J* = 10.3, 4.0, 1.5 Hz, 1H, C^7**H$_2$**), 3.41 (dd, *J* = 17.1, 4.6 Hz, 1H, C^2**H$_2$**), 3.14 (dt, *J* = 11.1, 1.5 Hz, 1H, C^6**H$_2$**), 2.96 (dt, *J* = 17.0, 2.5 Hz, 1H, C^2**H$_2$**), 2.82 (brs, 1H, C^5**H**), 2.76 (ddd, *J* = 11.1, 3.9, 1.5 Hz, 1H, C^6**H$_2$**).

^{13}C NMR (126 MHz, $CDCl_3$) δ 161.0 (2 x C^{11}), 142.0 (C), 141.1 (C), 136.5 (C), 131.5 (C^{19}H), 130.1 (q, *J* = 33.4 Hz, C^{16}/C^{18}), 129.7 (q, *J* = 31.2 Hz, C^{16}/C^{18}), 129.1 (q, *J* = 3.9 Hz, C^{20}H), 124.4 (C^{3}H), 123.7 (q, *J* =

274.1 Hz, $C^{21}F_3/C^{22}F_3$), 123.5 (q, J = 272.4 Hz, $C^{21}F_3/C^{22}F_3$), 123.5 – 123.2 (m, $C^{17}H$), 104.7 (2 x $C^{10}H$), 99.5 ($C^{12}H$), 65.5 ($C^{7}H_2$), 58.0 ($C^{14}H_2$), 55.7 ($C^{6}H_2$), 55.5 (2 x $C^{13}H_3$), 53.7 ($C^{2}H_2$), 39.1 ($C^{5}H$).
^{19}F NMR (377 MHz, $CDCl_3$) δ –59.2, –62.9.
IR (neat) (cm^{-1}): 3298, 2937, 1592, 1345, 1275, 1155, 1121, 1083, 1055, 842.

(*N*-(2,4-bis(Trifluoromethyl)benzyl)-4-(3,5-bis(trifluoromethyl)phenyl)-1,2,3,6-tetrahydropyridin-3-yl)methanol (2p)

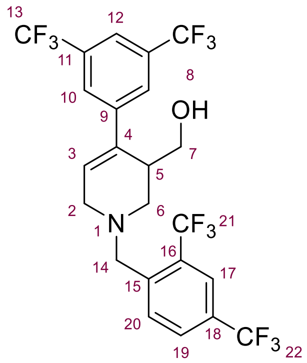

The title compound was prepared by General procedure E using salt **1p** (323 mg, 0.50 mmol). Purification by FCC (0%-20% EtOAc in pentane) gave *amine* **2p** (107 mg, 39%) as an orange oil.
HRMS (ESI): Exact mass calculated for $C_{23}H_{18}F_{12}NO$ $[M+H]^+$: 552.1191, found 552.1186
^{1}H NMR (500 MHz, $CDCl_3$) δ 7.95 (s, 1H, $C^{17}\mathbf{H}$), 7.89 – 7.83 (m, 2H, $C^{19}\mathbf{H}$ + $C^{20}\mathbf{H}$), 7.79 (s, 3H, 2 x $C^{10}\mathbf{H}$ + $C^{12}\mathbf{H}$), 6.21 (dd, J = 4.8, 2.2 Hz, 1H, $C^{3}\mathbf{H}$), 3.89 (s, 2H, $C^{14}\mathbf{H_2}$), 3.76 (dd, J = 10.6, 2.6 Hz, 1H, $C^{7}\mathbf{H_2}$), 3.61 (ddd, J = 10.6, 4.3, 1.7 Hz, 1H, $C^{7}\mathbf{H_2}$), 3.47 (dd, J = 17.4, 4.7 Hz, 1H, $C^{2}\mathbf{H_2}$), 3.20 (dt, J = 11.2, 1.5 Hz, 1H, $C^{6}\mathbf{H_2}$), 3.01 (dt, J = 17.4, 2.5 Hz, 1H, $C^{2}\mathbf{H_2}$), 2.90 (s, 1H, $C^{5}\mathbf{H}$), 2.80 (ddd, J = 11.3, 3.8, 1.6 Hz, 1H, $C^{6}\mathbf{H_2}$).
^{13}C NMR (126 MHz, $CDCl_3$) δ 141.9 (C^{9}), 140.7 (C^{15}), 134.5 (C^{4}), 132.1 (q, J = 33.1 Hz, 2 x C^{11}), 131.5 ($C^{20}H$), 130.3 (q, J = 33.5 Hz, C^{16}/C^{18}), 129.9 (q, J = 31.3 Hz, C^{16}/C^{18}), 129.1 (q, J = 3.7 Hz, $C^{19}H$), 127.8 ($C^{3}H$), 126.4 (2 x $C^{10}H$), 123.7 (q, J = 274.1 Hz, $C^{21}F_3/C^{22}F_3$), 123.7 – 123.2 (m, $C^{17}H$), 123.4 (q, J = 272.8 Hz, 2 x $C^{13}F_3$ + $C^{21}F_3/C^{22}F_3$), 121.6 – 121.2 (m, $C^{12}H$), 65.0 ($C^{7}H_2$), 57.9 (d, J = 2.0 Hz, $C^{14}H_2$), 55.5 ($C^{6}H_2$), 53.6 ($C^{2}H_2$), 38.9 ($C^{5}H$).
^{19}F NMR (377 MHz, $CDCl_3$) δ –59.2, –62.85, –62.87.
IR (neat) (cm^{-1}): 3659, 2980, 2972, 2889, 1382, 1275, 1164, 1125, 1083, 965, 954.

Methyl-3-(1-(2,4-bis(Trifluoromethyl)benzyl)-3-(hydroxymethyl)-1,2,3,6-tetrahydropyridin-4-yl)benzoate (2q)

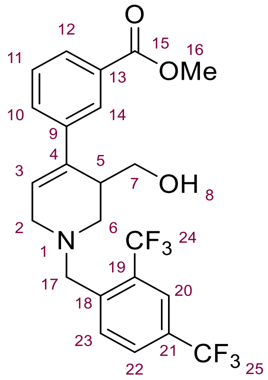

The title compound was prepared by General procedure E using salt **1q** (284 mg, 0.50 mmol). Purification by FCC (10-30% EtOAc in pentane) gave *amine* **2q** (184 mg, 78%) as a yellow oil.
HRMS (ESI): Exact mass calculated for $C_{23}H_{22}F_6NO$ $[M+H]^+$: 474.1498, found:474.1503
^{1}H NMR (400 MHz, $CDCl_3$) δ 8.04 (t, J = 1.8 Hz, 1H, $C^{14}\mathbf{H}$), 7.95 (dt, J = 9.5, 1.5 Hz, 2H, $C^{12}\mathbf{H}$ + $C^{20}\mathbf{H}$), 7.89 (d, J = 8.2 Hz, 1H, $C^{23}\mathbf{H}$), 7.83 (dd, J = 8.3, 1.9 Hz, 1H, $C^{22}\mathbf{H}$), 7.56 (ddd, J = 7.8, 1.9, 1.2 Hz, 1H, $C^{10}\mathbf{H}$), 7.41 (t, J = 7.8 Hz, 1H, $C^{11}\mathbf{H}$), 6.14 (dd, J = 4.7, 2.2 Hz, 1H, $C^{3}\mathbf{H}$), 4.03 (s, 1H, O**H**), 3.92 (s, 3H, $C^{16}\mathbf{H_3}$), 3.87

(s, 2H, $C^{17}\mathbf{H_2}$), 3.75 (dd, *J* = 10.4, 2.6 Hz, 1H, $C^{7}\mathbf{H_2}$), 3.63 (ddd, *J* = 10.4, 4.0, 1.6 Hz, 1H, $C^{7}\mathbf{H_2}$), 3.44 (dd, *J* = 17.0, 4.7 Hz, 1H, $C^{2}\mathbf{H_2}$), 3.17 (dt, *J* = 11.2, 1.5 Hz, 1H, $C^{6}\mathbf{H_2}$), 2.98 (dt, *J* = 16.9, 2.4 Hz, 1H, $C^{2}\mathbf{H_2}$), 2.93 (s, 1H, $C^{5}\mathbf{H}$), 2.79 (ddd, *J* = 11.1, 3.8, 1.5 Hz, 1H, $C^{6}\mathbf{H_2}$).
^{13}C NMR (126 MHz, $CDCl_3$) δ 167.2 ($C^{15}O$), 141.0 (C), 140.0 (C), 135.7 (C), 131.5 ($C^{23}H$), 130.8 ($C^{10}H$), 130.6 (C), 130.1 (q, *J* = 33.5 Hz, C^{19}/C^{21}), 129.8 (q, *J* = 31.0 Hz, C^{19}/C^{21}), 129.1 (q, *J* = 3.7 Hz, $C^{22}H$), 128.82 ($C^{11}H/C^{12}H$), 128.76 ($C^{11}H/C^{12}H$), 127.3 ($C^{14}H$), 125.2 ($C^{3}H$), 123.7 (d, *J* = 274.2 Hz, $C^{24}F_3/C^{25}F_3$), 123.5 (d, *J* = 272.3 Hz, $C^{24}F_3/C^{25}F_3$), 123.5 – 123.3 (m, $C^{20}H$), 65.3 ($C^{7}H_2$), 58.0 (d, *J* = 2.3 Hz, $C^{17}H_2$), 55.7 ($C^{6}H_2$), 53.7 ($C^{2}H_2$), 52.3 ($C^{16}H_3$), 38.9 ($C^{5}H$).
^{19}F NMR (376 MHz, $CDCl_3$) δ –59.2, –62.8.
IR (neat) (cm^{-1}): 3302, 2954, 1722, 1345, 1275, 1170, 1122, 1083, 1054, 757.

(*N*-(2,4-bis(Trifluoromethyl)benzyl)-4-(4-fluorophenyl)-5-(o-tolyl)-1,2,3,6-tetrahydropyridin-3-yl)methanol (2r)

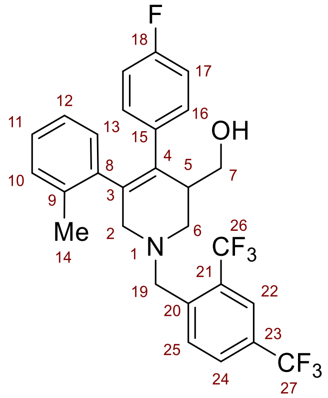

The title compound was prepared by General procedure E using salt **1r** (309 mg, 0.50 mmol). Purification by FCC (20% EtOAc in pentane) gave *amine* **2r** (145 mg, 55%) as a yellow oil.
HRMS (ESI): Exact mass calculated for $C_{28}H_{24}F_7NO$ $[M+H]^+$: 524.1819, found: 524.1818
^{1}H NMR (500 MHz, DMSO) δ 8.19 (t, *J* = 8.5 Hz, 1H, $C^{25}\mathbf{H}$), 8.11 (dd, *J* = 8.1, 2.2 Hz, 1H, $C^{24}\mathbf{H}$), 8.00 (d, *J* = 2.0 Hz, 1H, $C^{22}\mathbf{H}$), 7.23 – 6.57 (m, 9H, 9 x ArC**H**), 4.55 – 4.46 (m, 1H, O**H**), 3.04 – 3.80 (m, 2H, $C^{19}\mathbf{H}_2$), 3.62 – 3.50 (m, 1H, $C^{7}\mathbf{H}_2$), 3.38 (d, *J* = 16.8 Hz, 1H, $C^{2}\mathbf{H}_2$), 3.29 – 3.08 (m, 2H, $C^{6}\mathbf{H}_2$ + $C^{7}\mathbf{H}_2$), 2.93 – 2.66 (m, 2H, $C^{2}\mathbf{H}_2$ + $C^{5}\mathbf{H}$), 2.62 (td, *J* = 11.2, 3.9 Hz, 1H, $C^{6}\mathbf{H}_2$), 2.24 (s, 1H, $C^{14}\mathbf{H}_3$), 1.89 (s, 2H, $C^{14}\mathbf{H}_3$).
^{13}C NMR (126 MHz, DMSO) δ 160.6 (d, *J* = 243.3 Hz, C^{18}), 160.5 (d, *J* = 243.0 Hz, C^{18}), 142.9 (C^{20}), 139.5 (C), 139.2 (C), 136.9 (d, *J* = 3.1 Hz, C^{15}), 136.8 (d, *J* = 3.2 Hz, C^{15}), 134.4 (C), 134.0 (C), 133.2 (C), 132.7 (C), 131.8 (q, *J* = 2.8 Hz, $C^{25}H$), 130.4 (d, *J* = 7.9 Hz, 2 x $C^{16}H$), 2 x 129.9 (ArCH), 2 x 129.7 (ArCH), 129.6 – 129.3 (m, $C^{24}H$), 129.2 (C), 128.1 (q, *J* = 32.1 Hz, C^{21}/C^{23}), 128.0 (q, *J* = 33.2 Hz, C^{21}/C^{23}), 2 x 126.9 (ArCH), 125.6 (ArCH), 125.4 (ArCH), 123.6 (q, *J* = 274.3 Hz, $C^{26}F_3/C^{27}F_3$), 123.5 (q, *J* = 272.3 Hz, $C^{26}F_3/C^{27}F_3$), 122.8 – 122.6 (m, $C^{22}H$), 114.5 (d, *J* = 21.1 Hz, 2 x $C^{17}H$), 114.4 (d, *J* = 21.0 Hz, 2 x $C^{17}H$), 79.2 (C), 61.4 ($C^{7}H_2$), 61.3 ($C^{7}H_2$), 58.3 ($C^{2}H_2$), 57.4 ($C^{2}H_2$), 56.9 ($C^{19}H_2$), 56.8 ($C^{19}H_2$), 51.7 ($C^{6}H_2$), 51.4 ($C^{6}H_2$), 2 x 43.7 ($C^{5}H$), 19.8 ($C^{14}H_3$), 18.9 ($C^{14}H_3$).
Due to presence of rotamers that could not be resolved using VT NMR, doubling of majority of the peaks is observed in the ^{13}C NMR.
^{19}F NMR (377 MHz, DMSO) δ -63.9, -67.6.
IR (neat) (cm^{-1}): 3403, 3063, 2849, 1345, 1275, 1171, 1124, 1054, 835, 757.

(*N*-(2,4-bis(Trifluoromethyl)benzyl)-4-(4-fluorophenyl)-5-(3-methoxyphenyl)-1,2,3,6-tetrahydropyridin-3-yl)methanol (2s)

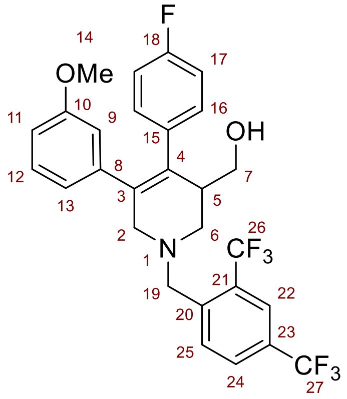

The title compound was prepared by General procedure E using salt **1s** (317 mg, 0.50 mmol). Purification by FCC (10-30% EtOAc in pentane) gave *amine* **2s** (117 mg, 44%) as a yellow oil.

HRMS (ESI): Exact mass calculated for $C_{28}H_{25}F_7NO_2$ $[M+H]^+$: 540.1768, found: 540.1766

^{1}H NMR (400 MHz, $CDCl_3$) δ 7.95 (m, 2H, C^{22}**H** + C^{24}**H**), 7.87 (d, *J* = 8.6 Hz, 1H, C^{25}**H**), 7.09 – 6.97 (m, 3H, C^{12}**H** + 2 x C^{16}**H**), 6.91 – 6.79 (m, 2H, 2 x C^{17}**H**), 6.65 (ddd, *J* = 8.3, 2.6, 1.0 Hz, 1H, C^{11}**H**), 6.58 (ddd, *J* = 7.6, 1.6, 1.0 Hz, 1H, C^{13}**H**), 6.50 (dd, *J* = 2.6, 1.6 Hz, 1H, C^{9}**H**), 4.16 (s, 1H, O**H**), 3.97 – 3.84 (m, 2H, C^{19}**H₂**), 3.72 (d, *J* = 16.6 Hz, 1H, C^{2}**H₂**), 3.67 (dd, *J* = 10.4, 2.6 Hz, 1H, C^{7}**H₂**), 3.61 (s, 3H, C^{14}**H₃**), 3.57 (ddd, *J* = 10.4, 3.9, 1.6 Hz, 1H, C^{7}**H₂**), 3.18 (dt, *J* = 11.2, 1.5 Hz, 1H, C^{6}**H₂**), 3.00 (dd, *J* = 16.6, 2.4 Hz, 1H, C^{2}**H₂**), 2.91 – 2.83 (m, 1H, C^{6}**H₂**), 2.74 (brs, 1H, C^{5}**H**).

^{13}C NMR (126 MHz, $CDCl_3$) δ 161.7 (d, *J* = 245.7 Hz, C^{18}), 159.3 (C^{10}), 141.0 (C^{8}), 140.9 (C^{20}), 136.3 (d, *J* = 3.3 Hz, C^{15}), 136.0 (C^{4}), 133.1 (C^{3}), 131.4 (C^{14}H), 131.0 (d, *J* = 7.8 Hz, 2 x C^{16}H), 130.2 (q, *J* = 33.5 Hz, C^{21}/C^{23}), 129.8 (q, *J* = 31.0 Hz, C^{21}/C^{23}), 129.1 (C^{11}H + C^{25}H), 123.7 (q, *J* = 274.2 Hz, $C^{26}F_3$/$C^{27}F_3$), 123.7 – 123.3 (m, C^{22}H), 123.5 (q, *J* = 272.3 Hz, $C^{26}F_3$/$C^{27}F_3$), 121.9 (C^{11}H), 115.3 (C^{9}H), 115.1 (d, *J* = 21.4 Hz, 2 x C^{17}H), 112.6 (C^{13}H), 65.2 ($C^{7}H_2$), 58.3 ($C^{2}H_2$), 58.0 (q, *J* = 2.2 Hz, $C^{19}H_2$), 55.7 ($C^{6}H_2$), 55.2 ($C^{14}H_3$), 42.6 (C^{5}H).

^{19}F NMR (377 MHz, $CDCl_3$) δ –59.2, –62.8, –114.7 - –116.1 (m).

IR (neat) (cm^{-1}): 3372, 2921, 1601, 1508, 1345, 1275, 1169, 1124, 1083, 1053.

(*N*-(2,4-bis(Trifluoromethyl)benzyl)-4,5-bis(4-fluorophenyl)-1,2,3,6-tetrahydropyridin-3-yl)methanol (2t)

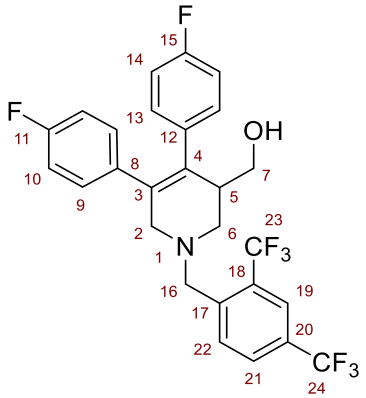

The title compound was prepared by General procedure E using salt **1t** (155 mg, 0.25 mmol). Purification by FCC (10-20% EtOAc in pentane) gave *amine* **2t** (49 mg, 37%) as a yellow oil.

HRMS (ESI): Exact mass calculated for $C_{27}H_{22}F_8NO$ $[M+H]^+$: 528.1568, found: 528.1564

^{1}H NMR (400 MHz, $CDCl_3$) δ 7.98 – 7.91 (m, 2H, C^{19}**H** + C^{22}**H**), 7.88 (d, *J* = 8.9 Hz, 1H, C^{21}**H**), 7.03 – 6.96 (m, 2H, 2 x C^{9}**H**/C^{13}**H**), 6.97 – 6.91 (m, 2H, 2 x C^{9}**H**/C^{13}**H**), 6.88 – 6.83 (m, 2H, 2 x C^{10}**H**/C^{14}**H**), 6.83 – 6.76 (m, 2H, 2 x C^{10}**H**/C^{14}**H**), 4.12 (d, *J* = 5.2 Hz, 1H, O**H**), 3.97 – 3.84 (m, 2H, C^{16}**H₂**), 3.69 (d, *J* = 14.5 Hz, 1H,

C²H₂), 3.67 – 3.63 (m, 1H, C^7**H₂**), 3.55 (ddd, *J* = 10.4, 3.9, 1.7 Hz, 1H, C^7**H₂**), 3.19 (dt, *J* = 11.2, 1.5 Hz, 1H, C^6**H₂**), 2.98 (dd, *J* = 16.6, 2.5 Hz, 1H, C^2**H₂**), 2.86 (ddd, *J* = 11.2, 4.0, 1.5 Hz, 1H, C^6**H₂**), 2.73 (s, 1H, C^5**H**).
^{13}C NMR (126 MHz, $CDCl_3$) δ 161.7 (d, *J* = 246.6 Hz, C^{11}/C^{15}), 161.7 (d, *J* = 246.1 Hz, C^{11}/C^{15}), 140.8 (C^{17}), 136.0 (d, *J* = 3.4 Hz, C^8/C^{12}), 135.5 (d, *J* = 3.4 Hz, C^8/C^{12}), 135.1 (C^4), 133.6 (C^3), 131.4 (C^{22}H), 131.1 (app, *J* = 7.5 Hz, , 2 x C^{10}H + 2 x C^{14}H), 130.2 (q, *J* = 33.5 Hz, C^{18}/C^{20}), 129.9 (q, *J* = 31.3 Hz, C^{18}/C^{20}), 129.1 (q, *J* = 3.7 Hz, C^{21}H), 123.7 (q, *J* = 274.2 Hz, $C^{23}F_3$/$C^{24}F_3$), 123.5 (m, C^{19}H), 123.5 (q, *J* = 272.3 Hz, $C^{23}F_3$/$C^{24}F_3$), 115.2 (d, *J* = 21.2 Hz, 2 x C^9H/C^{13}H), 115.1 (d, *J* = 21.3 Hz, 2 x C^9H/C^{13}H), 65.1 (C^7H_2), 58.4 (C^2H_2), 58.0 ($C^{16}H_2$), 55.7 (C^6H_2), 42.6 (C^5H).
^{19}F NMR (377 MHz, $CDCl_3$) δ –59.2, –62.8, –114.6 - –115.1 (m), –115.1 - –115.6 (m).
IR (neat) (cm^{-1}): 3375, 2923, 1508, 1346, 1275, 1159, 1124, 1054, 833, 674

(*N*-(2,4-bis(Trifluoromethyl)benzyl)-5-butyl-4-(4-fluorophenyl)-1,2,3,6-tetrahydropyridin-3-yl)methanol (2u)

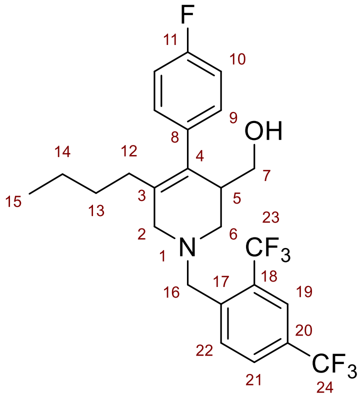

The title compound was prepared by General procedure E using salt **1u** (58 mg, 0.10 mmol). Purification by FCC (10-20% EtOAc in pentane) gave *amine* **2u** (23 mg, 47%) as a yellow oil.
HRMS (ESI): Exact mass calculated for $C_{25}H_{27}F_7NO$ $[M+H]^+$: 490.1975, found:490.1972
^{1}H NMR (400 MHz, $CDCl_3$) δ 7.93 (s, 1H, C^{19}**H**), 7.90 (d, *J* = 8.3 Hz, 1H, C^{22}**H**), 7.88 – 7.81 (m, 1H, C^{21}**H**), 7.17 – 7.07 (m, 2H, 2 x C^9**H**), 7.11 – 6.98 (m, 2H, C^{10}**H**), 4.23 (s, 1H, OH), 3.91 – 3.78 (m, 2H, C^{16}**H₂**), 3.60 (dd, *J* = 10.3, 2.4 Hz, 1H, C^7**H₂**), 3.52 (ddd, *J* = 10.3, 3.6, 1.8 Hz, 1H, C^7**H₂**), 3.28 (d, *J* = 15.9 Hz, 1H, C^2**H₂**), 3.06 (dt, *J* = 11.0, 1.5 Hz, 1H, C^6**H₂**), 2.82 (dd, *J* = 15.8, 2.2 Hz, 1H, C^2**H₂**), 2.77 – 2.69 (m, 1H, C^6**H₂**), 2.39 (s, 1H, C^5**H**), 1.98 – 1.87 (m, 1H, C^{12}**H₂**), 1.82 (m, 1H, C^{12}**H₂**), 1.36 – 1.21 (m, 2H, C^{13}**H₂**), 1.25 – 1.02 (m, 2H, C^{14}**H₂**), 0.75 (t, *J* = 7.2 Hz, 3H, C^{15}**H₃**).
^{13}C NMR (126 MHz, $CDCl_3$) δ 161.9 (d, *J* = 245.2 Hz, C^{11}), 141.1 (C^{17}), 136.9 (d, *J* = 3.3 Hz, C^8), 134.7 (C^4), 131.4 (C^{22}H), 130.8 (C^3), 130.6 (d, *J* = 7.7 Hz, 2 x C^9H), 130.1 (q, *J* = 33.6 Hz, C^{18}/C^{20}), 129.7 (q, *J* = 31.1 Hz, C^{18}/C^{20}), 129.1 (q, *J* = 3.7 Hz, C^{21}H), 123.7 (q, *J* = 274.2 Hz, $C^{23}F_3$/$C^{24}F_3$), 123.5 (q, *J* = 272.3 Hz, $C^{23}F_3$/$C^{24}F_3$), 123.6 – 123.1 (m, C^{19}H), 115.3 (d, *J* = 21.2 Hz, 2 x C^{10}H), 65.1 (C^7H_2), 58.1 (q, *J* = 2.0 Hz, $C^{16}H_2$), 56.4 (C^6H_2), 56.3 (C^2H_2), 42.8 (C^5H), 31.9 ($C^{12}H_2$), 30.9 ($C^{13}H_2$), 22.6 ($C^{14}H_2$), 14.0 ($C^{15}H_3$).
^{19}F NMR (377 MHz, $CDCl_3$) δ –59.2, –62.8, –114.9 - –118.9 (m).
IR (neat) (cm^{-1}): 3338, 2970, 1345, 1276, 1161, 1129, 1032, 951, 816, 674.

(4-(Benzofuran-5-yl)-*N*-(2,4-bis(trifluoromethyl)benzyl)-1,2,3,6-tetrahydropyridin-3-yl)methanol (2v)

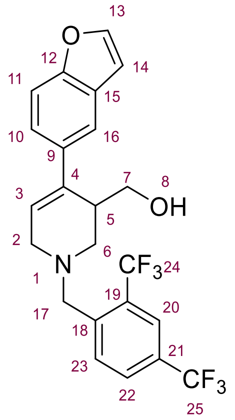

The title compound was prepared by General procedure E using salt **1**v (258 mg, 0.47 mmol). Purification by FCC (20% EtOAc in pentane) gave *amine* **2v** (49%) as an orange oil.

HRMS (ESI): Exact mass calculated for $C_{23}H_{20}F_6NO_2$ $[M+H]^+$: 456.1393, found: 456.1387

^{1}H NMR (400 MHz, $CDCl_3$) δ 7.94 (s, 1H, C^{20}**H**), 7.91 (d, *J* = 8.1 Hz, 1H, C^{23}**H**), 7.84 (dd, *J* = 8.2, 1.9 Hz, 1H, C^{22}**H**), 7.62 (d, *J* = 2.2 Hz, 1H, C^{13}**H**), 7.58 (d, *J* = 1.8 Hz, 1H, C^{16}**H**), 7.46 (dt, *J* = 8.6, 0.8 Hz, 1H, C^{11}**H**), 7.31 (dd, *J* = 8.6, 1.9 Hz, 1H, C^{10}**H**), 6.74 (dd, *J* = 2.2, 1.0 Hz, 1H, C^{14}**H**), 6.05 (dd, *J* = 4.7, 2.2 Hz, 1H, C^3**H**), 4.14 (s, 1H, O**H**), 3.88 (s, 2H, C^{17}**H₂**), 3.75 (dd, *J* = 10.3, 2.7 Hz, 1H, C^7**H₂**), 3.66 (ddd, *J* = 10.3, 3.9, 1.6 Hz, 1H, C^7**H₂**), 3.44 (dd, *J* = 16.7, 4.7 Hz, 1H, C^2**H₂**), 3.18 (dt, *J* = 11.2, 1.6 Hz, 1H, C^6**H₂**), 2.99 (dt, *J* = 16.8, 2.4 Hz, 1H, C^2**H₂**), 2.93 (brs, 1H, C^5**H**), 2.82 (ddd, *J* = 11.1, 3.9, 1.6 Hz, 1H, C^6**H₂**).

^{13}C NMR (101 MHz, $CDCl_3$) δ 154.7 (C^{12}), 145.6 (C^{13}H), 141.2 (C^{18}), 136.7 (C), 135.0 (C), 131.5 (C^{23}H), 130.0 (d, *J* = 33.0 Hz, C^{19}/C^{21}), 129.4 (d, *J* = 31.2 Hz, C^{19}/C^{21}), 129.1 – 128.9 (m, C^{22}H), 127.8 (C^{15}), 123.9 (C^3H), 123.7 (d, *J* = 274.1 Hz, $C^{24}F_3$/$C^{25}F_3$), 123.5 (q, *J* = 272.4 Hz, $C^{24}F_3$/$C^{25}F_3$), 123.5 – 123.2 (m, C^{20}H), 123.0 (C^{10}H), 118.9 (C^{16}H), 111.4 (C^{11}H), 106.8 (C^{14}H), 65.4 (C^7H_2), 58.0 (d, *J* = 2.1 Hz, $C^{17}H_2$), 55.8 (C^6H_2), 53.8, (C^2H_2), 39.7 (C^5H).

^{19}F NMR (377 MHz, $CDCl_3$) δ –59.2, –62.8.

IR (neat) (cm^{-1}): 3325, 2923, 1630, 1345, 1274, 1170, 1122, 1083, 1055, 672.

(*N*-(2,4-bis(Trifluoromethyl)benzyl)-4-(1-methyl-1H-indol-7-yl)-1,2,3,6-tetrahydropyridin-3-yl)methanol (2w)

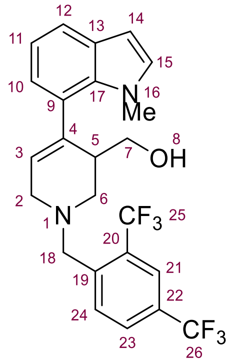

The title compound was prepared by General procedure E using salt **1w** (141 mg, 0.25 mmol). Purification by FCC (5%-30% EtOAc in pentane) gave the *amine* **2w** (78 mg, 67%) as a pale yellow solid.

m.p. (EtOAc): 90-92°C

HRMS (ESI): Exact mass calculated for $C_{24}H_{23}F_6N_2O$ $[M+H]^+$: 469.1709, found: 469.1706

^{1}H NMR (500 MHz, DMSO) δ 8.15 (d, *J* = 8.2 Hz, 1H, C^{24}**H**), 8.06 (d, *J* = 8.3 Hz, 1H, C^{23}**H**), 7.97 (s, 1H, C^{21}**H**), 7.45 (dd, *J* = 7.9, 1.2 Hz, 1H, C^{12}**H**), 7.19 (d, *J* = 3.2 Hz, 1H, C^{15}**H**), 6.97 (t, *J* = 7.5 Hz, 1H, C^{11}**H**), 6.81 (d, *J* = 7.2 Hz, 1H, C^{10}**H**), 6.44 (d, *J* = 3.2 Hz, 1H, C^{14}**H**), 5.70 (t, *J* = 3.4 Hz, 1H, C^3**H**), 4.12 (t, *J* = 5.1 Hz, 1H,

OH), 3.91 (s, 2H, $C^{18}H_2$), 3.82 (s, 3H, $C^{16}H_3$), 3.54 (td, J = 9.6, 5.6 Hz, 1H, C^7H_2), 3.41 – 3.28 (m, 2H, C^7H_2 + C^2H_2), 3.08 (m, 1H, C^2H) 3.03 (dd, J = 11.1, 3.7 Hz, 1H, C^6H_2), 2.74 (dd, J = 11.2, 4.1 Hz, 1H, C^6H_2), 2.65 (s, 1H, C^5H). C^2H at 3.08 hidden underneath the water peak, proven by 2D NMR (COSY)
^{13}C NMR (126 MHz, DMSO) δ 142.5 (C^{19}), 135.2 (C), 133.2 (C), 131.7 ($C^{24}H$), 130.9 ($C^{10}H$), 129.4 (C), 128.8 (q, J = 4.6 Hz, $C^{23}H$), 128.0 (q, J = 30.7 Hz C^{20} + C^{22}), 126.1 (C^3H), 125.6 (C), 123.1 (q, J = 272.9 Hz, $C^{25}F_3/C^{26}F_3$), 122.2 (m, $C^{15}H$ + $C^{21}H$), 119.0 ($C^{12}H$), 118.1 ($C^{11}H$), 100.4 ($C^{14}H$), 61.2 (C^7H_2), 57.0 ($C^{18}H_2$), 52.3 (C^2H_2), 51.7 (C^6H_2), 44.1 (C^5H), 35.4 ($C^{16}H_3$). 1 of $C^{25}F_3/C^{26}F_3$ not observed
^{19}F NMR (471 MHz, DMSO) δ –58.3, –61.4.
IR (neat) (cm^{-1}): 3056, 2882, 1630, 1345, 1301, 1275, 1171, 1122, 1083, 1053, 796.

(*N*-(2,4-bis(Trifluoromethyl)benzyl)-4-(9-ethyl-9H-carbazol-3-yl)-1,2,3,6-tetrahydropyridin-3-yl)methanol (2x)

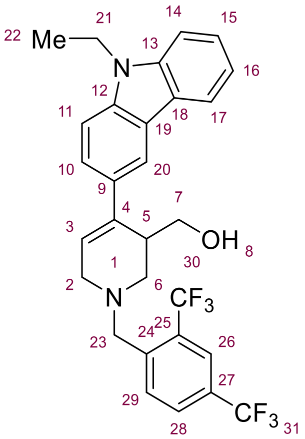

The title compound was prepared by General procedure E using salt **1x** (313 mg, 0.50 mmol). Purification by FCC (10%-50% EtOAc in pentane) gave *amine* **2x** (147 mg, 52%) as an orange solid
m.p. (EtOAc): 107-109°C
HRMS (ESI): Exact mass calculated for $C_{29}H_{27}F_6N_2O$ $[M+H]^+$: 533.2022, found:533.2015
^{1}H NMR (500 MHz, $CDCl_3$) δ 8.12 – 8.04 (m, 2H, $C^{17}H$ + $C^{20}H$), 7.97 – 7.91 (m, 2H, $C^{26}H$ + $C^{29}H$), 7.85 (dd, J = 8.2, 2.3 Hz, 1H, $C^{28}H$), 7.53 – 7.44 (m, 2H, $C^{11}H$ + $C^{14}H$), 7.43 – 7.35 (m, 2H, $C^{10}H$ + $C^{15}H$), 7.23 (ddd, J = 7.9, 7.1, 1.0 Hz, 1H, $C^{16}H$), 6.11 (dd, J = 4.8, 1.9 Hz, 1H, C^3H), 4.37 (q, J = 7.2 Hz, 2H, $C^{21}H_2$), 3.90 (s, 2H, $C^{23}H_2$), 3.80 (dd, J = 10.3, 2.6 Hz, 1H, C^7H_2), 3.73 (ddd, J = 10.3, 3.9, 1.6 Hz, 1H, C^7H_2), 3.52 – 3.44 (m, 1H, C^2H_2), 3.25 – 3.19 (m, 1H, C^6H_2), 3.12 – 2.98 (m, 2H, C^2H_2 + C^5H), 2.87 (ddd, J = 11.1, 3.8, 1.5 Hz, 1H, C^6H_2), 1.44 (t, J = 7.2 Hz, 3H, $C^{22}H_3$).
^{13}C NMR (126 MHz, $CDCl_3$) δ 141.3 (C^{24}), 140.5 (C^{12}), 139.7 (C), 137.1 (C), 131.5 ($C^{29}H$), 130.8 (C), 130.0 (q, J = 33.5 Hz, C^{25}/C^{27}), 129.7 (q, J = 31.1 Hz, C^{25}/C^{27}), 129.1 (q, J = 3.8 Hz, $C^{28}H$), 125.9 ($C^{11}H$), 124.3 ($C^{14}H$), 123.7 (q, J = 274.2 Hz, $C^{30}F_3/C^{31}F_3$), 123.5 (q, J = 272.3 Hz, $C^{30}F_3/C^{31}F_3$), 123.5 – 123.2 (m, $C^{26}H$), 123.2 (C), 123.1 (C), 122.8 (C^3H), 120.6 ($C^{17}H$), 119.0 ($C^{16}H$), 118.1 ($C^{20}H$), 108.7 ($C^{10}H/C^{15}H$), 108.5 ($C^{10}H/C^{15}H$), 65.7 (C^7H_2), 58.1 (q, J = 2.4 Hz, $C^{23}H_2$), 56.0 (C^6H_2), 54.0 (C^2H_2), 39.5 (C^5H), 37.8 ($C^{21}H_2$), 14.0 ($C^{22}H_3$).
^{19}F NMR (377 MHz, $CDCl_3$) δ –59.2, –62.8.
IR (neat) (cm^{-1}): 3353, 2971, 2885, 1634, 1379, 1160, 1130, 1108, 951, 817.

Methyl 7-(*N*-(2,4-bis(Trifluoromethyl)benzyl)-3-(hydroxymethyl)-1,2,3,6-tetrahydropyridin-4-yl)-1-ethyl-4-oxo-1,4-dihydroquinoline-3-carboxylate (2y)

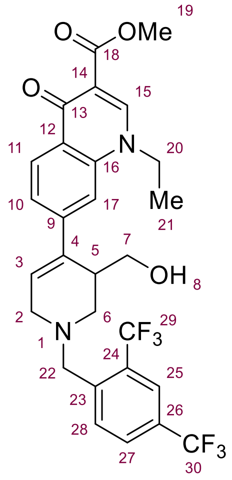

The title compound was prepared by General procedure E using salt **1y** (169 mg, 0.25 mmol). Purification by FCC (2% MeOH in CH_2Cl_2) gave *amine* **2y** (33 mg, 23%) as a pale brown solid.

m.p. (CH_2Cl_2)**:** 129-131 °C

HRMS (ESI): Exact mass calculated for $C_{28}H_{27}F_6N_2O_4$ $[M+H]^+$: 569.1870, found: 569.1870

^{1}H NMR (500 MHz, $CDCl_3$) δ 8.53 (s, 1H, C^{18}**H**), 8.48 (d, *J* = 8.3 Hz, 1H, C^{11}**H**), 7.94 (d, *J* = 1.8 Hz, 1H, C^{25}**H**), 7.90 (d, *J* = 8.5 Hz, 1H, C^{28}**H**), 7.84 (dd, *J* = 8.2, 1.9 Hz, 1H, C^{27}**H**), 7.42 (d, *J* = 9.3 Hz, 2H, C^{10}**H** + C^{17}**H**), 6.18 (dd, *J* = 4.9, 2.2 Hz, 1H, C^{3}**H**), 4.33 – 4.24 (m, 2H, C^{20}**H₂**), 3.93 (s, 3H, C^{19}**H₃**), 3.90 (s, 2H, 22**H₂**), 3.76 (dd, *J* = 10.5, 2.6 Hz, 1H, C^{7}**H₂**), 3.63 (ddd, *J* = 10.4, 4.4, 1.4 Hz, 1H, C^{7}**H₂**), 3.48 (dd, *J* = 17.4, 4.8 Hz, 1H, C^{2}**H₂**), 3.21 (d, *J* = 11.2 Hz, 1H, C^{6}**H₂**), 3.03 (d, *J* = 17.3 Hz, 1H, C^{2}**H₂**), 2.95 (s, 1H, C^{5}**H**), 2.86 – 2.80 (m, 1H, C^{6}**H₂**), 1.55 (t, *J* = 7.2 Hz, 3H, C^{21}**H₃**).

^{13}C NMR (126 MHz, $CDCl_3$) δ 174.2 (C^{13}), 166.8 (C^{18}), 149.2 (C^{9}H), 144.7 (C^{15}), 140.8 (C^{23}), 139.1 (C^{16}), 136.1 (C^{4}), 131.6 (C^{28}H), 130.2 (q, *J* = 33.6 Hz, C^{24}/C^{26}), 129.8 (q, *J* = 31.1 Hz, C^{24}/C^{26}), 129.1 (q, *J* = 3.7 Hz, C^{27}H), 128.5 (C^{11}H), 127.0 (C^{3}H + C^{12}), 123.7 (q, *J* = 274.2 Hz, $C^{29}F_3$/$C^{30}F_3$), 123.6 – 123.4 (m, C^{25}H), 123.4 (q, *J* = 272.5 Hz, $C^{29}F_3$/$C^{30}F_3$), 123.3 (C^{10}H), 113.4 (C^{17}H), 111.0 (C^{14}), 65.1 ($C^{7}H_2$), 57.9 ($C^{22}H_2$), 55.4 ($C^{6}H_2$), 53.7 ($C^{2}H_2$), 52.3 ($C^{19}H_3$), 49.0 ($C^{20}H_2$), 39.3 (C^{5}H), 14.7 ($C^{21}H_3$).

^{19}F NMR (377 MHz, $CDCl_3$) δ –59.2, –62.9.

IR (neat) (cm^{-1}): 3373, 2360, 1721, 1614, 1468, 1346, 1276, 1165, 1129, 756.

***N*-(2,4-bis(Trifluoromethyl)benzyl)-4-methoxy-1,2,3,6-tetrahydropyridin-3-yl)methanol (2z)**

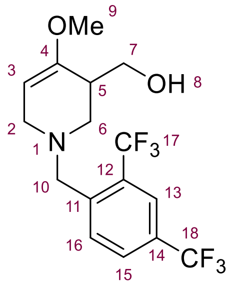

N-(2,4-bis(Trifluoromethyl)benzyl)-4-methoxpyridinium iodide **1z** (116 mg, 0.25 mmol, 1.00 equiv.), paraformaldehyde (450 mg, 60.0 equiv.), and $[RhCp*Cl_2]_2$ (3.1 mg, 2 mol%) was added to a microwave vial. MeOH (0.83 mL, 0.2 M) and $Mg(OMe)_2$ (0.42 mL, 1.5 equiv, 0.89M in MeOH) was added and the solution heated at 45 °C for 16 hours. The solution concentrated under reduce pressure and re-dissolved in CH_2Cl_2 (30 m L), brine (50 mL) and water (50 mL). The solution was separated, and the aqueous layer was extracted with CH_2Cl_2 (2 x 30 mL). The organic layers were combined, dried

($MgSO_4$), filtered, and concentrated *in vacuo*. The crude material was purified by FCC (5%-30% EtOAc in Pentane) to furnish the *amine* **2z** (46 mg, 50%) as a yellow oil.

HRMS (ESI): Exact mass calculated for $C_{16}H_{18}F_6NO_2$ $[M+H]^+$: 370.1236, found: 340.1240.

^{1}H NMR (400 MHz, $CDCl_3$) δ 7.90 (s, 1H, C^{13}**H**), 7.88 (d, *J* = 8.1 Hz, 1H, C^{16}**H**), 7.80 (dd, *J* = 8.2, 2.4 Hz, 1H, C^{15}**H**), 4.72 (dd, *J* = 4.6, 2.4 Hz, 1H, C^{3}**H**), 3.92 (s, 1H, O**H**), 3.85 (ddd, *J* = 10.2, 3.8, 1.3 Hz, 1H, C^{7}**H_2**), 3.80 (s, 2H, C^{10}**H_2**), 3.75 (dd, *J* = 10.2, 3.6 Hz, 1H, C^{7}**H_2**), 3.56 (s, 3H, C^{9}**H_3**), 3.24 (ddt, *J* = 14.5, 4.6, 1.3 Hz, 1H, C^{2}**H_2**), 2.93 – 2.84 (m, 2H, C^{2}**H_2** + C^{6}**H_2**), 2.71 (ddd, *J* = 11.1, 4.4, 1.2 Hz, 1H, C^{6}**H_2**), 2.37 – 2.30 (brs, 1H, C^{5}**H**).

^{13}C NMR (101 MHz, $CDCl_3$) δ 153.6 (C^{4}), 141.3 (C^{11}), 131.2 (C^{16}H), 129.8 (d, *J* = 33.5 Hz, C^{12}/C^{14}), 129.5 (d, *J* = 30.9 Hz, C^{12}/C^{14}), 128.8 (q, *J* = 4.5 Hz, C^{15}H), 123.6 (q, *J* = 274.2 Hz, $C^{17}F_3$/$C^{18}F_3$), 123.4 (q, *J* = 272.0 Hz, $C^{17}F_3$/$C^{18}F_3$), 123.3 – 122.9 (m, C^{13}H), 93.0 (C^{3}H), 65.0 ($C^{7}H_2$), 57.5 (d, *J* = 2.1 Hz, $C^{10}H_2$), 55.2 ($C^{6}H_2$), 54.5 ($C^{9}H_3$), 51.8 ($C^{2}H_2$), 40.1 (C^{5}H).

^{19}F NMR (377 MHz, $CDCl_3$) δ –59.3, –62.9.

IR (neat) (cm^{-1}):

(*N*-(2,4-bis(Trifluoromethyl)benzyl)-4-methyl-1,2,3,6-tetrahydropyridin-3-yl)methanol (2aa)

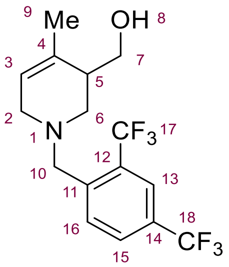

The title compound was prepared by General procedure E using salt **1aa** (224 mg, 0.50 mmol). Purification by FCC (10%-20% EtOAc in pentane) gave *amine* **2aa** (35 mg mg, 20%) as a yellow oil.

HRMS (ESI): Exact mass calculated for $C_{16}H_{18}F_6NO$ $[M+H]^+$: 354.1285, found: 354.1287.

^{1}H NMR (400 MHz, $CDCl_3$) δ 7.90 (s, 1H, C^{13}**H**), 7.84 (d, *J* = 8.4 Hz, 1H, C^{16}**H**), 7.80 (dd, *J* = 8.5, 1.8 Hz, 1H, C^{15}**H**), 5.70 – 5.45 (m, 1H, C^{3}**H**), 4.36 (s, 1H, O**H**), 3.86 (ddd, *J* = 10.3, 3.4, 1.8 Hz, 1H, C^{7}**H_2**), 3.80 – 3.74 (m, 3H, C^{7}**H_2** + C^{10}**H_2**), 3.26 – 3.12 (m, 1H, C^{2}**H_2**), 2.95 (dt, *J* = 11.2, 1.6 Hz, 1H, C^{6}**H_2**), 2.74 (dq, *J* = 15.9, 2.4 Hz, 1H, C^{2}**H_2**), 2.63 (ddd, *J* = 11.1, 4.0, 1.8 Hz, 1H, C^{6}**H_2**), 2.10 (s, 1H, C^{5}**H**), 1.78 (q, *J* = 1.9 Hz, 3H, C^{9}**H_3**).

^{13}C NMR (126 MHz, $CDCl_3$) δ 141.1 (C^{11}), 132.3 (C^{4}), 131.5 (C^{16}H), 130.0 (q, *J* = 33.5 Hz, C^{12}/C^{14}), 129.7 (q, *J* = 31.0 Hz, C^{12}/C^{14}), 129.0 (q, *J* = 3.7 Hz, C^{15}H), 123.7 (q, *J* = 274.0 Hz, $C^{17}F_3$/$C^{18}F_3$), 123.5 (q, *J* = 272.4 Hz, $C^{17}F_3$/$C^{18}F_3$), 123.4 – 123.1 (m, C^{13}H), 121.7 (C^{3}H), 65.1 ($C^{7}H_2$), 58.1 (d, *J* = 2.3 Hz, $C^{10}H_2$), 56.1 ($C^{6}H_2$), 53.3 ($C^{2}H_2$), 41.5 (C^{5}H), 21.2 ($C^{9}H_3$).

^{19}F NMR (377 MHz, $CDCl_3$) δ –59.2, –62.9.

IR (neat) (cm^{-1}):

(*N*-(2,4-bis(Trifluoromethyl)benzyl)-4-(tert-butyl)-1,2,3,6-tetrahydropyridin-3-yl)methanol (2ab)

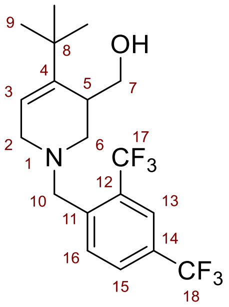

The title compound was prepared by General procedure E using salt **1ab** (245 mg, 0.50 mmol). Purification by FCC (10%-20% EtOAc in pentane) gave *amine* **2ab** (53 mg mg, 27%) as a yellow oil.

HRMS (ESI): Exact mass calculated for $C_{19}H_{24}F_6NO$ $[M+H]^+$: 396.1743, found: 396.1757

^{1}H NMR (500 MHz, $CDCl_3$) δ 7.91 (s, 1H, C^{13}**H**), 7.85 (d, *J* = 8.2 Hz, 1H, C^{16}**H**), 7.81 (dd, *J* = 8.2, 1.8 Hz, 1H, C^{15}**H**), 5.66 (dd, *J* = 4.1, 2.4 Hz, 1H, C^{3}**H**), 4.78 (s, 1H, O**H**), 3.92 (ddd, *J* = 10.6, 3.8, 2.2 Hz, 1H, C^{7}**H₂**), 3.82 (dd, *J* = 10.6, 2.2 Hz, 1H, $C^{7}\mathbf{H_2}$), 3.78 (s, 2H, $C^{10}\mathbf{H_2}$), 3.36 (dd, *J* = 16.4, 4.1 Hz, 1H, $C^{2}\mathbf{H_2}$), 2.98 (ddd, *J* = 10.8, 2.1, 1.0 Hz, 1H, $C^{6}\mathbf{H_2}$), 2.78 (dt, *J* = 16.4, 2.2 Hz, 1H, $C^{2}\mathbf{H_2}$), 2.53 (dt, *J* = 10.7, 2.7 Hz, 1H, $C^{6}\mathbf{H_2}$), 2.41 (s, 1H, C^{5}**H**), 1.12 (s, 9H, 3 x $C^{9}\mathbf{H_3}$).

^{13}C NMR (126 MHz, $CDCl_3$) δ 143.4 (C^4), 141.1 (C^{11}), 131.5 (C^{16}H), 130.0 (q, *J* = 32.3 Hz, C^{12}/C^{14}), 129.8 (q, *J* = 32.3 Hz, C^{12}/C^{14}), 129.0 (q, *J* = 3.7 Hz, C^{15}H), 123.7 (q, *J* = 274.2 Hz, $C^{17}F_3$/$C^{18}F_3$), 123.5 (q, *J* = 272.3 Hz, $C^{17}F_3$/$C^{18}F_3$), 123.3 (m, C^{13}H), 119.5 (C^3H), 67.3 (C^7H_2), 58.0 (q, *J* = 2.0 Hz, $C^{10}H_2$), 57.6 (C^6H_2), 53.9(C^2H_2), 37.4 (C^5H), 35.5 (C^8), 30.3 (3 x C^9H_3).

^{19}F NMR (377 MHz, $CDCl_3$) δ –59.1, –62.9.

IR (neat) (cm^{-1}): 3324, 2962, 1345, 1275, 1169, 1122, 1083, 1056, 1029, 672.

Derivatisation of products

N-(2,4-bis(Trifluoromethyl)benzyl)-3-(iodomethyl)-4-phenyl-1,2,3,6-tetrahydropyridine (4)

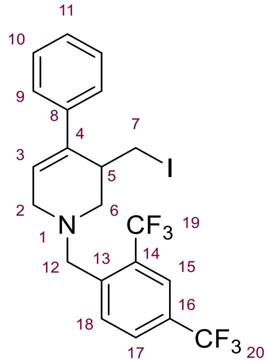

Amine **2c** (83 mg, 0.20 mmol), PPh_3 (115 mg, 0.44 mmol) and imidazole (34 mg, 0.50 mmol) were dissolved in CH_2Cl_2 (4 mL). The solution was cooled to 0 °C, I_2 (112 mg, 0.44 mmol) was added and the reaction was allowed to warm to rt. After 2 h the reaction was quenched with an *aq. sat.* solution of $Na_2S_2O_3$ (5 mL). The solution was separated, and the aqueous layer was extracted with CH_2Cl_2 (2 x 5 mL). The organic layers were combined, dried ($MgSO_4$), filtered, and concentrated *in vacuo*. The crude material was purified by FCC (0%-2% Et_2O in pentane) to give *iodide* **4** (69 mg, 66%) as a yellow oil.
HRMS (ESI): Exact mass calculated for $C_{21}H_{19}F_6NO^{127}I$ [M+H]+: 526.0461, found: 526.0454.
^{1}H NMR (400 MHz, $CDCl_3$) δ 8.02 (d, *J* = 8.2 Hz, 1H, C^{18}**H**), 7.92 (s, 1H, C^{15}**H**), 7.84 (d, *J* = 8.2 Hz, 1H, C^{17}**H**), 7.40 – 7.27 (m, 5H, 5 x ArC**H**), 6.02 (dd, *J* = 4.2, 2.6 Hz, 1H, C^3**H**), 3.94 – 3.81 (m, 2H, C^{12}**H₂**), 3.45 (dd, *J* = 11.0, 9.7 Hz, 1H, C^7**H₂**), 3.30 (dd, *J* = 17.4, 4.4 Hz, 1H, C^2**H₂**), 3.24 – 3.13 (m, 2H, C^6**H₂** + C^7**H₂**), 3.04 (d, *J* = 11.2 Hz, 1H, C^5**H**), 2.98 (dt, *J* = 17.3, 2.5 Hz, 1H, C^2**H₂**), 2.62 (ddd, *J* = 11.5, 3.7, 1.6 Hz, 1H, C^6**H₂**).
^{13}C NMR (126 MHz, $CDCl_3$) δ 142.2 (C^{13}), 139.1 (C), 137.6 (C), 131.6 (C^{18}H), 129.7 (q, *J* = 33.9 Hz, C^{14}/C^{16}), 129.5 (q, *J* = 31.8 Hz, C^{14}/C^{16}), 129.0 (C^{17}H), 128.9 (2 x ArCH), 127.8 (ArCH), 125.8 (2 x ArCH), 124.4 (C^3H), 123.8 (q, *J* = 274.1 Hz, $C^{19}F_3$/$C^{20}F_3$), 123.6 (q, *J* = 272.3 Hz, $C^{19}F_3$/$C^{20}F_3$), 123.2 (C^{15}H), 57.5 ($C^{12}H_2$), 54.5 (C^2H_2), 53.7 (C^6H_2), 41.3 (C^5H), 9.5 (C^7H_2).
^{19}F NMR (377 MHz, $CDCl_3$) δ –59.5, –62.8.
IR (neat) (cm^{-1}): 2808, 2161, 1348, 1292, 1279, 1167, 1122, 1084, 1057, 757.

3-((Benzyloxy)methyl)-N-(2,4-bis(trifluoromethyl)benzyl)-4-phenyl-1,2,3,6-tetrahydropyridine (S11)

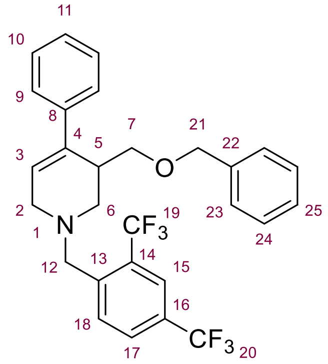

Amine **2c** (83 mg, 0.20 mmol), 15-crown-5 (80 µL, 0.40 mmol) and benzyl bromide (0.24 mL, 2.00 mmol) were dissolved in THF (1 mL). The solution was cooled to 0 °C and NaH (64 mg, 2.0 mmol) was added portion wise. The reaction mixture was allowed to warm to rt and stir for 2 hours. The reaction was quenched with water (5 mL), the aq layer extracted with CH_2Cl_2 (2 x 5 mL), dried ($MgSO_4$) and concentrated *in vacuo*. The crude material was purified by FCC (0%-4% Et_2O in pentane) to give *amine* **S11** (72 mg, 73%) as a white solid.

m.p. (EtOAc): 78-80 °C
HRMS (ESI): Exact mass calculated for $C_{28}H_{26}F_6NO$ $[M+H]^+$: 506.1911, found: 506.1913.
^{1}H NMR (400 MHz, $CDCl_3$) δ 7.94 (d, *J* = 8.2 Hz, 1H, C^{18}**H**), 7.89 (s, 1H, C^{15}**H**), 7.69 (dd, *J* = 7.8, 1.5 Hz, 1H, C^{17}**H**), 7.41 – 7.12 (m, 10H, 10 x ArC**H**), 6.01 (ddd, *J* = 4.5, 2.5, 0.8 Hz, 1H, C^3**H**), 4.45 (d, *J* = 11.8 Hz, 1H, C^{21}**H$_2$**), 4.32 (d, *J* = 11.8 Hz, 1H, C^{21}**H$_2$**), 3.90 – 3.75 (m, 2H, C^{12}**H$_2$**), 3.71 (dd, *J* = 10.0, 9.1 Hz, 1H, C^7**H$_2$**), 3.45 – 3.28 (m, 2H, C^2**H$_2$** + C^7**H$_2$**), 3.18 (ddd, *J* = 11.1, 2.4, 1.2 Hz, 1H, C^6**H$_2$**), 3.09 (dt, *J* = 9.8, 3.1 Hz, 1H, C^5**H**), 3.01 (dt, *J* = 17.1, 2.5 Hz, 1H, C^2**H$_2$**), 2.54 (dd, *J* = 11.2, 3.6 Hz, 1H, C^6**H$_2$**).
^{13}C NMR (126 MHz, $CDCl_3$) δ 142.7 (C^{13}), 140.0 (C^{22}), 138.4 (C^5), 136.2 (C^4), 131.2 (C^{18}H), 129.5 (q, *J* = 33.3 Hz, C^{14}/C^{16}), 129.3 (q, *J* = 31.3 Hz, C^{14}/C^{16}), 128.7 (C^{17}H+ ArCH), 128.5 (2 x ArCH), 127.7 (4 x ArCH), 127.4 (ArCH), 125.8 (2 x ArCH), 124.6 (C^{3}H), 123.8 (q, *J* = 274.2 Hz, C^{19}F$_3$/C^{20}F$_3$), 123.7 (q, *J* = 272.2 Hz, C^{19}F$_3$/C^{20}F$_3$), 123.1 – 122.9 (m, C^{15}H), 73.1 (C^{21}H$_2$), 70.8 (C^{7}H$_2$), 57.6 (C^{12}H$_2$), 53.8 (C^{2}H$_2$), 52.0 (C^{6}H$_2$), 38.8 (C^{5}H).
^{19}F NMR (377 MHz, $CDCl_3$) δ –59.8, –62.7.
IR (neat) (cm^{-1}): 2857, 1348, 1280, 1166, 1118, 1085, 1056, 755, 695, 674.

3-((Benzyloxy)methyl)-4-phenyl-1,2,3,6-tetrahydropyridine (5)

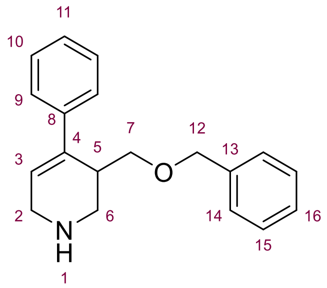

Amine **S11** (25 mg, 0.05 mmol) added to a microwave vial, dissolved in DCE (0.13 mL), 1-chloroethyl chloroformate (31 µL, 0.40 mmol) was added and the reaction was sealed and heated to 120 °C for 16 h. The reaction was allowed to cool to rt, the solvent was switched to MeOH (1 mL) and heated to reflux for 4 hours. Purification by FCC (0%-10% MeOH in CH_2Cl_2) gave *amine* **5** (11 mg mg, 77%) as a yellow oil
HRMS (ESI): Exact mass calculated for $C_{19}H_{22}NO$ $[M+H]^+$: 280.1696, found: 280.1696.
^{1}H NMR (400 MHz, $CDCl_3$) δ 7.45 – 7.16 (m, 10H, 10 x ArC**H**), 5.96 (d, *J* = 3.4 Hz, 1H, C^3**H**), 4.46 (s, 2H, C^{12}**H$_2$**), 3.85 – 3.71 (m, 2H, C^6**H$_2$**), 3.68 (dd, *J* = 12.5, 2.8 Hz, 1H, C^2**H$_2$**), 3.60 (dd, *J* = 9.6, 7.0 Hz, 1H, C^7**H$_2$**), 3.49 (dd, *J* = 9.6, 3.4 Hz, 1H, C^7**H$_2$**), 3.27 (dd, *J* = 12.7, 4.8 Hz, 1H, C^2**H$_2$**), 3.14 (s, 1H, C^5**H**).
^{13}C NMR (101 MHz, $CDCl_3$) δ 139.0 (C), 137.9 (C), 137.5 (C), 128.8 (2 x ArCH), 128.5 (2 x ArCH), 128.1 (ArCH), 127.9 (ArCH), 127.8 (2 x ArCH), 126.2 (2 x ArCH), 121.1 (C^{3}H), 73.3 (C^{12}H$_2$), 69.9 (C^{7}H$_2$), 44.4 (C^{2}H$_2$), 43.1 (C^{6}H$_2$), 35.2 (C^{5}H).
IR (neat) (cm^{-1}): 3415, 2922, 2853, 1659, 1639, 1593, 1454, 1092, 1073, 1027.

3-((Benzyloxy)methyl)-*N*-(2,4-bis(trifluoromethyl)benzyl)-4-phenyl-1,2,3,6-tetrahydropyridine (6)

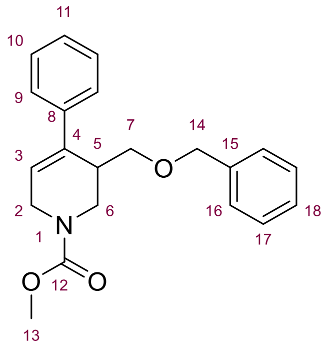

Amine **S11** (25 mg, 0.05 mmol) added to a microwave vial, dissolved in DCE (0.13 mL), methyl chloroformate (31 µL, 0.40 mmol) was added and the reaction was sealed and heated to 120 °C for 16 h. Afterwards the reaction was allowed to cool to rt, diluted with CH_2Cl_2 (5 mL) and quenched with

water (5 mL) The layers were separated, the aqueous layer was extracted with CH_2Cl_2 (2 x 5 mL), dried ($MgSO_4$) and concentrated *in vacuo*. The crude material was purified by FCC (5%-30% EtOAc in pentane) to give *carbamate* **6** (16 mg, 90%) as a pale yellow oil.
HRMS (ESI): Exact mass calculated for $C_{21}H_{23}NO_3{}^{23}Na$ $[M+Na]^+$: 360.1568, found: 360.1570.
^{1}H NMR (500 MHz, DMSO) δ 7.45 – 7.20 (m, 10H, 10 x ArC**H**), 6.07 (t, *J* = 3.4 Hz, 1H, C^3**H**), 4.44 (d, *J* = 12.2 Hz, 1H, C^{14}**H$_2$**), 4.38 (d, *J* = 12.1 Hz, 1H, C^{14}**H$_2$**), 4.29 – 4.19 (m, 2H, C^2**H$_2$** + C^7**H$_2$**), 3.85 (dt, *J* = 19.1, 2.7 Hz, 1H, C^2**H$_2$**), 3.66 (s, 3H, C^{13}**H$_3$**), 3.31 (ddd, *J* = 9.7, 4.0, 1.0 Hz, 1H, C^6**H$_2$**), 3.27 (t, *J* = 9.2 Hz, 1H, C^6**H$_2$**), 3.20 (ddd, *J* = 13.0, 3.7, 0.9 Hz, 1H, C^7**H$_2$**), 3.10 (brs, 1H, C^5**H**).
^{13}C NMR (126 MHz, DMSO) δ 155.3 (C^{12}), 139.0 (C), 138.1 (C), 135.5 (C), 127.9 (2 x ArCH), 127.6 (2 x ArCH), 126.8 (2 x ArCH), 126.7 (2 x ArCH), 125.0 (2 x ArCH), 122.5 (C^{3}H), 71.8 (C^{14}H$_2$), 69.1 (C^{7}H$_2$), 51.7 (C^{13}H$_3$), 43.2 (C^{2}H$_2$), 41.8 (C^{6}H$_2$), 36.9 (C^{5}H).
IR (neat) (cm^{-1}): 3414, 2920, 2852, 1754, 1696, 1445, 1265, 1209, 1095, 769.

3-((Benzo[d][1,3]dioxol-5-yloxy)methyl)-*N*-(2,4-bis(trifluoromethyl)benzyl)-4-phenyl-1,2,3,6-tetrahydropyridine (S12)

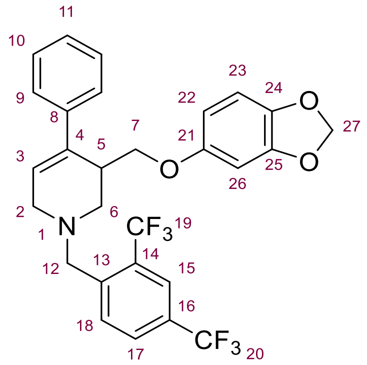

Amine **2c** (62 mg, 0.15 mmol), PPh_3 (47 mg, 0.18 mmol) and sesamol (41 mg, 0.30 mmol) were dissolved in THF (1.5 mL). The solution was cooled to 0°C and DIAD (35 µL, 0.21 mmol) was added dropwise. The reaction was then heated to 50 °C and stirred for 16 h. The reaction was evaporated, the crude redissolved in CH_2Cl_2 (20 mL), washed with NaOH (2 x 20 mL, 1.0 M) and dried ($MgSO_4$). The crude material was purified by FCC (0%-5% EtOAc in pentane) to give *amine* **S12** (54 mg, 66%) as a white solid.
m.p. (EtOAc): 110-112 ^{o}C
HRMS (ESI): Exact mass calculated for $C_{28}H_{24}F_6NO_3$ $[M+H]^+$: 536.1655, found: 536.1655.
^{1}H NMR (500 MHz, $CDCl_3$) δ 7.87 – 7.82 (m, 2H, C^{15}**H** + C^{18}**H**), 7.44 – 7.39 (m, 2H, C^{11}**H** + C^{17}**H**), 7.38 – 7.32 (m, 2H, 2 x C^9**H**/C^{10}**H**), 7.32 – 7.27 (m, 2H, 2 x C^9**H**/C^{10}**H**), 6.65 (d, *J* = 8.5 Hz, 1H, C^{23}**H**), 6.27 (d, *J* = 2.5 Hz, 1H, C^{26}**H**), 6.14 (dd, *J* = 8.5, 2.5 Hz, 1H, C^{22}**H**), 6.11 (dd, *J* = 4.6, 2.3 Hz, 1H, C^3**H**), 5.90 (dd, *J* = 9.8, 1.4 Hz, 2H, C^{27}**H$_2$**), 4.09 (dd, *J* = 10.3, 8.8 Hz, 1H, C^7**H$_2$**), 3.87 (d, *J* = 14.5 Hz, 1H, C^{12}**H$_2$**), 3.80 (d, *J* = 15.5 Hz, 1H, C^{12}**H$_2$**), 3.77 – 3.73 (m, 1H, C^7**H$_2$**), 3.46 (dd, *J* = 17.2, 4.6 Hz, 1H, C^2**H$_2$**), 3.20 (s, 1H, C^5**H**), 3.16 (dd, *J* = 11.1, 1.4 Hz, 1H, C^6**H$_2$**), 3.12 (dt, *J* = 17.1, 2.4 Hz, 1H, C^2**H$_2$**), 2.53 – 2.40 (m, 1H, C^6**H$_2$**).
^{13}C NMR (126 MHz, $CDCl_3$) δ 154.4 (C^{21}), 148.4 (C^{25}), 142.4 (C^{13}), 141.7 (C^{24}), 139.7 (C^8), 135.7 (C^4), 131.0 (C^{18}H), 129.3 (q, *J* = 33.3 Hz, C^{14}/C^{16}), 129.2 (q, *J* = 31.3 Hz, C^{14}/C^{16}), 128.8 (ArCH), 128.7 – 128.5 (m, C^{17}H), 127.7 (ArCH), 125.7 (ArCH), 125.2 (C^{3}H), 123.8 (q, *J* = 274.1 Hz, C^{19}F$_3$/C^{20}F$_3$), 123.6 (q, *J* = 272.2 Hz, C^{19}F$_3$/C^{20}F$_3$), 123.1 – 122.8 (m, C^{15}H), 107.9 (C^{23}H), 105.3 (C^{22}H), 101.3 (C^{27}H$_2$), 97.9 (C^{26}H), 68.3 (C^{7}H$_2$), 57.1 (q, *J* = 2.1 Hz, C^{12}H$_2$), 54.2 (C^{2}H$_2$), 50.8 (C^{6}H$_2$), 38.3 (C^{5}H).
^{19}F NMR (377 MHz, $CDCl_3$) δ –59.8, –62.8.
IR (neat) (cm^{-1}): 2898, 1629, 1490, 1343, 1271, 1173, 1159, 1119, 1101, 1095.

3-((Benzo[d][1,3]dioxol-5-yloxy)methyl)-4-phenyl-1,2,3,6-tetrahydropyridine (7)

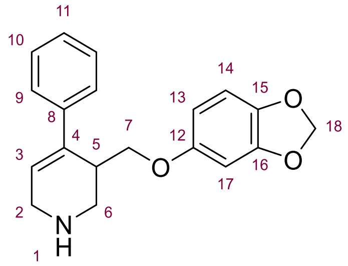

Amine **S12** (54 mg, 0.10 mmol) added to a microwave vial, dissolved in DCE (0.25 mL), 1-chloroethyl chloroformate (86 µL, 0.80 mmol) was added and the reaction was sealed and heated to 120 °C for 16 h. The reaction was allowed to cool to rt, the solvent was switched to MeOH (1 mL) and heated to reflux for 4 hours. Purification by FCC (1%-10% MeOH in CH_2Cl_2) gave *amine* **7** (20 mg mg, 65%) as a yellow oil

HRMS (ESI): Exact mass calculated for $C_{19}H_{20}NO_3$ $[M+H]^+$: 310.1438, found: 310.1437.

^{1}H NMR (400 MHz, $CDCl_3$) δ 7.41 – 7.28 (m, 5H, 5 x ArC**H**), 6.63 (d, *J* = 8.5 Hz, 1H, C^{14}**H**), 6.42 (d, *J* = 2.4 Hz, 1H, C^{17}**H**), 6.23 (dd, *J* = 8.5, 2.5 Hz, 1H, C^{13}**H**), 6.14 – 6.08 (m, 1H, C^3**H**), 5.87 (s, 2H, C^{18}**H₂**), 3.94 (t, *J* = 9.1 Hz, 1H, C^7**H₂**), 3.82 – 3.72 (m, 1H, C^7**H₂**), 3.63 – 3.49 (m, 2H, C^2**H₂**), 3.47 – 3.39 (m, 1H, C^6**H₂**), 3.15 – 3.05 (m, 2H, C^5**H** + C^6**H₂**), 2.92 (s, 1H, N**H**).

^{13}C NMR (101 MHz, $CDCl_3$) δ 154.4 (C^{12}), 148.3 (C^{16}), 141.8 (C^{15}), 140.5 (C^8), 136.2 (C^4), 128.7 (2 x ArCH), 127.5 (ArCH), 127.0 (C^3H), 126.0 (2 x ArCH), 108.0 (C^{14}H), 106.0 (C^{13}H), 101.2 ($C^{18}H_2$), 98.3 (C^{17}H), 69.0 (C^7H_2), 45.7 (C^2H_2), 45.2 (C^6H_2), 36.4 (C^5H).

IR (neat) (cm^{-1}): 2919, 2160, 2027, 1977, 1630, 1486, 1179, 1035, 757, 698.

Synthesis of Paroxetine

(*trans-N*-(2,4-bis(Trifluoromethyl)benzyl)-4-(4-fluorophenyl)piperidin-3-yl)methanol (8)

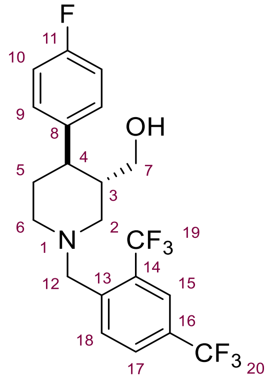

A microwave vial was charged with *amine* **2j** (43 mg, 0.10 mmol) and Crabtree's catalyst (16 mg, 20 mol%) evacuated and back filled with argon. Degassed CH_2Cl_2 (2 mL) were added and the reaction mixture was purged with hydrogen for 5 minutes. The reaction was left to stir for 20 h at room temperature under an atmosphere of hydrogen. The d.r. of the crude was 3:1 *trans*:*cis*, however, purification by FCC (0%-10% EtOAc in pentane) gave a single diastereoisomer of *amine* **8** (28 mg, 65%) as a yellow oil

HRMS (ESI): Exact mass calculated for $C_{21}H_{21}F_7NO$ $[M+H]^+$: 436.1506, found: 436.1503.

^{1}H NMR (400 MHz, $CDCl_3$) δ 8.07 (d, *J* = 8.2 Hz, 1H, C^{18}**H**), 7.89 (s, 1H, C^{15}**H**), 7.80 (d, *J* = 8.2 Hz, 1H, C^{17}**H**), 7.23 – 7.15 (m, 2H, 2 x C^{9}**H**), 7.06 – 6.95 (m, 2H, 2 x C^{10}**H**), 3.77 (s, 2H, C^{12}**H$_2$**), 3.40 (dd, *J* = 10.8, 3.1 Hz, 1H, C^{7}**H$_2$**), 3.25 (dd, *J* = 10.8, 6.6 Hz, 1H, C^{7}**H$_2$**), 3.18 – 3.10 (m, 1H, C^{2}**H$_2$**), 2.91 (d, *J* = 11.3 Hz, 1H, C^{6}**H$_2$**), 2.37 (td, *J* = 11.0, 4.9 Hz, 1H, C^{4}**H**), 2.19 (td, *J* = 11.2, 3.5 Hz, 1H, C^{6}**H$_2$**), 2.14 – 1.97 (m, 2H, C^{2}**H$_2$** + C^{3}**H**), 1.92 – 1.74 (m, 2H, C^{5}**H$_2$**).

^{13}C NMR (126 MHz, $CDCl_3$) δ 161.7 (d, *J* = 244.3 Hz, C^{11}), 142.9 (C^{13}), 140.0 (d, *J* = 3.3 Hz, C^{8}), 130.9 (C^{16}H), 129.4 (q, *J* = 33.2 Hz, C^{14}/C^{16}), 129.3 (q, *J* = 31.3 Hz, C^{14}/C^{16}), 128.9 (d, *J* = 7.7 Hz, 2 x C^{9}H), 128.7 (q, *J* = 3.8 Hz, C^{17}H), 123.8 (d, *J* = 274.2 Hz, C^{19}F$_3$/C^{20}F$_3$), 123.7 (d, *J* = 272.1 Hz, C^{19}F$_3$/C^{20}F$_3$), 123.3 – 122.9 (m, C^{15}H), 115.6 (d, *J* = 21.2 Hz, 2 x C^{10}H), 64.0 (C^{7}H$_2$), 58.4 (q, *J* = 2.5 Hz, C^{12}H$_2$), 57.7 (C^{6}H$_2$), 54.4 (C^{2}H$_2$), 44.5 (C^{3}H), 44.3 (C^{4}H), 34.7 (C^{5}H).

^{19}F NMR (376 MHz, $CDCl_3$) δ –60.0, –62.7, –115.6 to –118.8 (m).

IR (neat) (cm^{-1}): 3257, 2922, 1510, 1345, 1275, 1168, 1122, 1083, 1053, 831.

Relative stereochemistry determined using NOESY experiment:

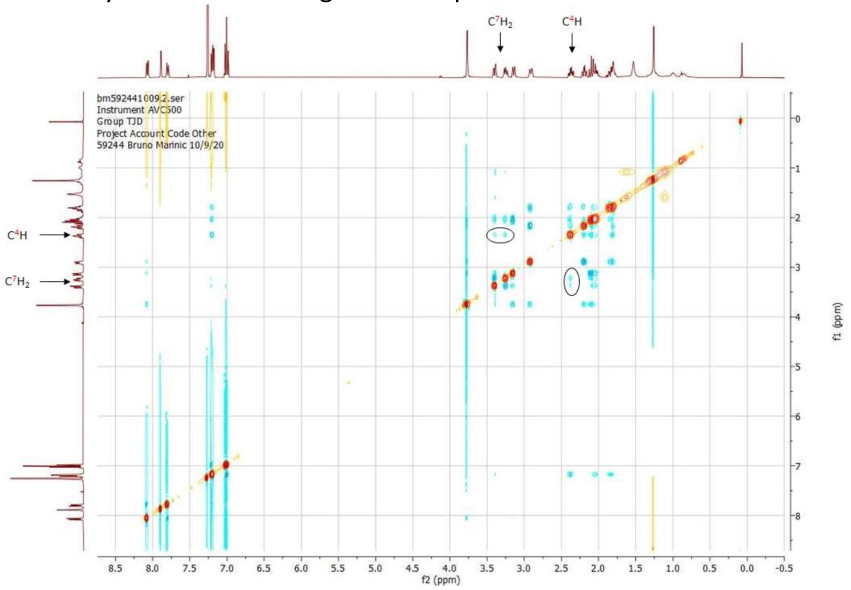

***trans*-3-((Benzo[d][1,3]dioxol-5-yloxy)methyl)-*N*-(2,4-bis(trifluoromethyl)benzyl)-4-(4-fluorophenyl)piperidine (S13)**

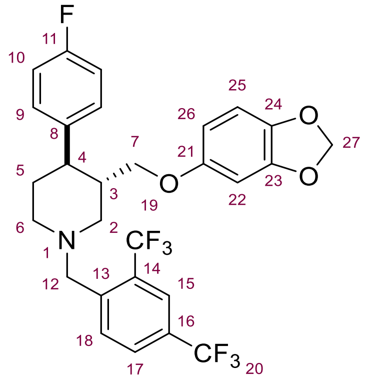

Amine **8** (45 mg, 0.10 mmol), PPh_3 (32 mg, 0.12 mmol) and sesamol (28 mg, 0.20 mmol) were dissolved in THF (1 mL). The solution was cooled to 0°C and DIAD (47 µL, 0.12 mmol) was added dropwise. The reaction was then heated to 50 °C and stirred for 16 h. The reaction was evaporated, the crude redissolved in CH_2Cl_2 (20 mL), washed with NaOH (2 x 20 mL, 1.0 M) and dried ($MgSO_4$). The crude material was purified by FCC (0%-20% Et_2O in pentane) to give *amine* **S13** (46mg, 84%) as a white solid.
m.p. (EtOAc): 100-102 °C
HRMS (ESI): Exact mass calculated for $C_{28}H_{25}F_7NO_3$ $[M+H]^+$: 556.1717, found: 556.1713.
^{1}H NMR (400 MHz, $CDCl_3$) δ 8.08 (d, *J* = 8.2 Hz, 1H, C^{18}**H**), 7.89 (s, 1H, C^{15}**H**), 7.81 (dd, *J* = 8.2, 1.9 Hz, 1H, C^{17}**H**), 7.24 – 7.14 (m, 2H, 2 x C^{9}**H**), 7.04 – 6.94 (m, 2H, 2 x C^{10}**H**), 6.61 (d, *J* = 8.4 Hz, 1H, C^{25}**H**), 6.31 (d, *J* = 2.5 Hz, 1H, C^{22}**H**), 6.10 (dd, *J* = 8.5, 2.5 Hz, 1H, C^{26}**H**), 5.87 (s, 2H, C^{27}**H**), 3.85 – 3.72 (m, 2H, C^{12}**H**), 3.57 (dd, *J* = 9.5, 2.4 Hz, 1H, C^{7}**H$_2$**), 3.50 – 3.41 (m, 1H, C^{7}**H$_2$**), 3.26 – 3.15 (m, 1H, C^{2}**H$_2$**), 2.92 (ddt, *J* = 11.3, 4.0, 2.4 Hz, 1H, C^{6}**H$_2$**), 2.51 (td, *J* = 11.1, 4.9 Hz, 1H, C^{4}**H**), 2.32 – 2.16 (m, 3H, C^{2}**H$_2$** + C^{5}**H** + C^{6}**H$_2$**), 1.95 – 1.76 (m, 2H, C^{5}**H$_2$**).
^{13}C NMR (126 MHz, $CDCl_3$) δ 161.7 (d, *J* = 244.3 Hz, C^{11}), 154.4 (C^{21}), 148.3 (C^{23}), 142.9 (C^{13}), 141.8 (C^{24}), 139.7 (d, *J* = 3.2 Hz, C^{8}), 130.9 (C^{18}H), 129.4 (q, *J* = 33.3 Hz, C^{14}/C^{16}), 129.3 (q, *J* = 31.3 Hz, C^{14}/C^{16}), 129.0 (d, *J* = 7.7 Hz, 2 x C^{9}H), 128.7 (q, *J* = 3.8 Hz, C^{17}H), 123.8 (q, *J* = 274.2 Hz, C^{19}F$_3$/C^{20}F$_3$), 123.7 (q, *J* = 272.2 Hz, C^{19}F$_3$/C^{20}F$_3$), 123.4 – 123.0 (m, C^{15}H), 115.6 (d, *J* = 21.2 Hz, 2 x C^{10}H), 108.0 (C^{25}H), 105.7 (C^{26}H), 101.2 (C^{27}H$_2$), 98.1 (C^{22}H), 69.7 (C^{7}H$_2$), 58.3 (q, *J* = 2.2 Hz, C^{12}H$_2$), 58.0 (C^{2}H$_2$), 54.3 (C^{6}H$_2$), 44.1 (C^{4}H), 42.4 (C^{3}H), 34.6 (C^{5}H$_2$).
^{19}F NMR (377 MHz, $CDCl_3$) δ –60.0, –62.7, –115.6 to –117.2 (m).
IR (neat) (cm^{-1}): 2918, 2160, 2026, 1503, 1348, 1280, 1198, 1164, 1120, 833.

(±)-*Paroxetine*

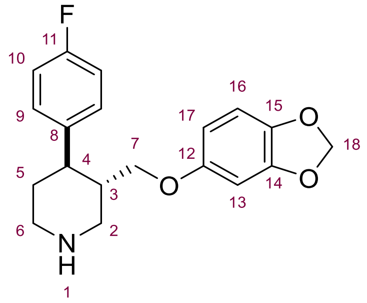

Amine **S13** (54 mg, 0.04 mmol) added to a microwave vial, dissolved in DCE (0.13 mL), 1-chloroethyl chloroformate (37 µL, 0.35 mmol) was added and the reaction was sealed and heated to 120 °C for 16 h. The reaction was allowed to cool to rt, the solvent was switched to MeOH (1 mL) and heated to

reflux for 4 hours. Purification by FCC (1%-10% 7N NH_3 MeOH in CH_2Cl_2) gave *Paroxetine* (8 mg, 55%) as a colourless oil. The spectroscopic data was consistent with previous reports.[17]

^{1}H NMR (400 MHz, $CDCl_3$) δ 7.21 – 7.12 (m, 2H, 2 x C^9**H**), 7.03 – 6.92 (m, 2H, 2 x C^{10}**H**), 6.62 (d, *J* = 8.4 Hz, 1H, C^{16}**H**), 6.33 (d, *J* = 2.5 Hz, 1H, C^{13}**H**), 6.12 (dd, *J* = 8.5, 2.5 Hz, 1H, C^{17}**H**), 5.87 (s, 2H, C^{18}**H_2**), 3.57 (dd, *J* = 9.4, 3.0 Hz, 1H, C^7**H_2**), 3.48 – 3.39 (m, 2H, C^2**H_2** + C^7**H_2**), 3.20 (dt, *J* = 11.6, 3.0 Hz, 1H, C^6**H_2**), 2.80 – 2.73 (m, 1H, C^6**H_2**), 2.69 (dd, *J* = 12.2, 10.9 Hz, 1H, C^2**H_2**), 2.59 (td, *J* = 11.7, 4.2 Hz, 1H, C^4**H**), 2.08 (tdt, *J* = 10.9, 6.9, 3.4 Hz, 1H C^3**H**), 1.87 – 1.77 (m, 1H, C^5**H_2**), 1.76 – 1.60 (m, 1H, C^5**H_2**).

^{13}C NMR (101 MHz, $CDCl_3$) δ 161.67 (d, *J* = 245.0 Hz, C^{11}), 154.6 (C^{12}), 148.3 (C^{14}), 141.7 (C^{15}), 140.1 (d, *J* = 2.9 Hz, C^8), 128.9 (d, *J* = 7.7 Hz, 2 x C^9H), 115. 6(d, *J* = 21.0 Hz, C^{10}H), 108.0 (C^{16}H), 105.8 (C^{17}H), 101.2 ($C^{18}H_2$), 98.1 (C^{13}H), 69.7 (C^7H_2), 50.5 (C^2H_2), 47.2 (C^6H_2), 44.7 (C^4H), 43.1 (C^3H), 35.5 (C^5H_2).

^{19}F NMR (377 MHz, $CDCl_3$) δ –116.6.

Proposed reaction mechanism

The mechanism of the reaction is thought to proceed via the same pathway we previously reported.[16] Oxidation of paraformaldehyde to methyl formate forms the ruthenium hydride. Reduction can then occur on either C-6 or C-2, with the former forming the inactive enamine **A'** which can be re-oxidised back to the starting material. Reduction at C-2 forms the reactive enamine **A** that then goes on to trap formaldehyde to give iminium **B**. Another reduction at C-6 then generates the final product. The formaldehyde thus serves as the electrophile and the source of hydride in the reaction.

Metal-hydride formation:

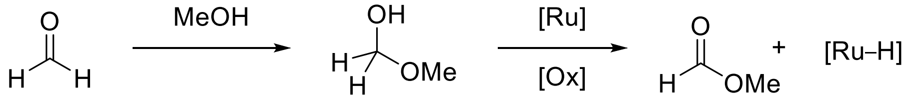

Reaction mechanism:

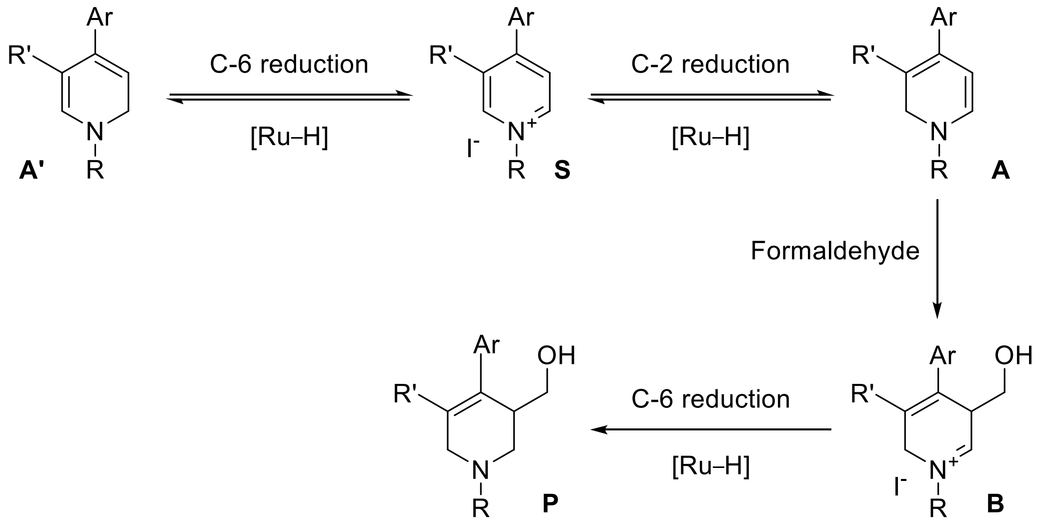

---

[17] S. Krautwald, M. A. Schafroth, D. Sarlah, and E. M. Carreira, *J. Am. Chem. Soc.*, 2014, **136**, 3020–3023.

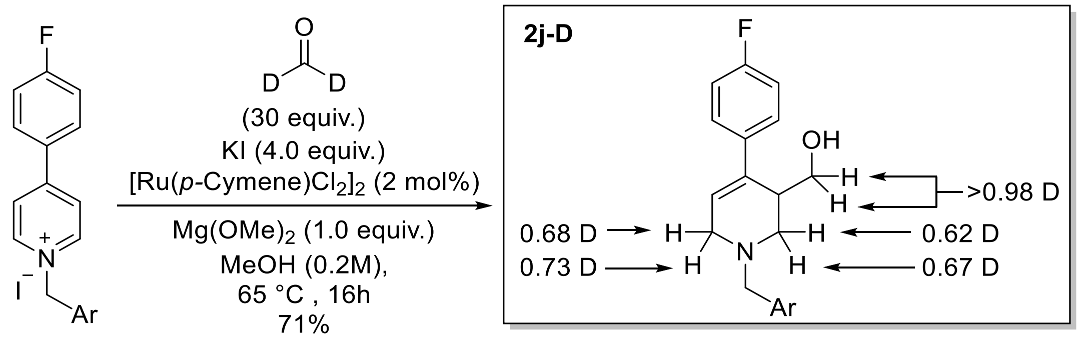

The title compound **2j-D** was prepared by General procedure E using salt **1j** (264 mg, 0.50 mmol) and deuterated paraformaldehyde (480 mg, 15 mmol). Purification by FCC (5%-20% EtOAc in pentane) gave *amine* **2j** (155 mg, 71%) as a yellow oil. Complete deuterium incorporation was observed for the hydroxymethyl group and significant levels of incorporation at the C-2 and C-6 positions. These observations are consistent with the mechanism described above.

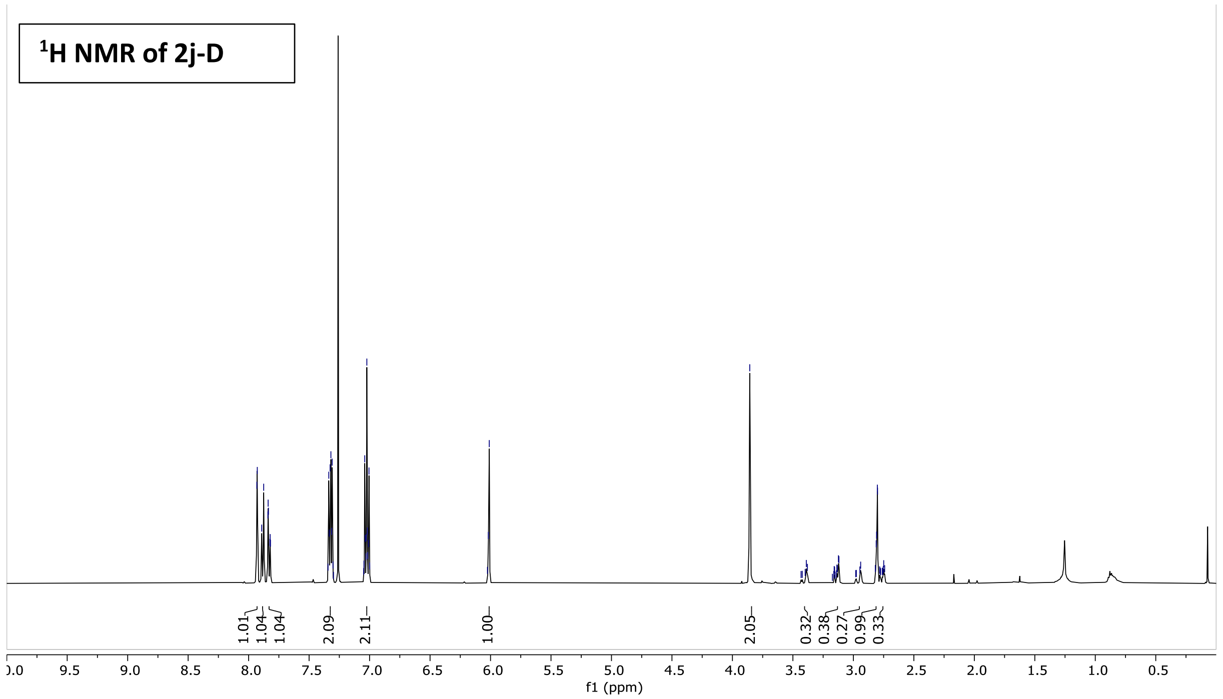

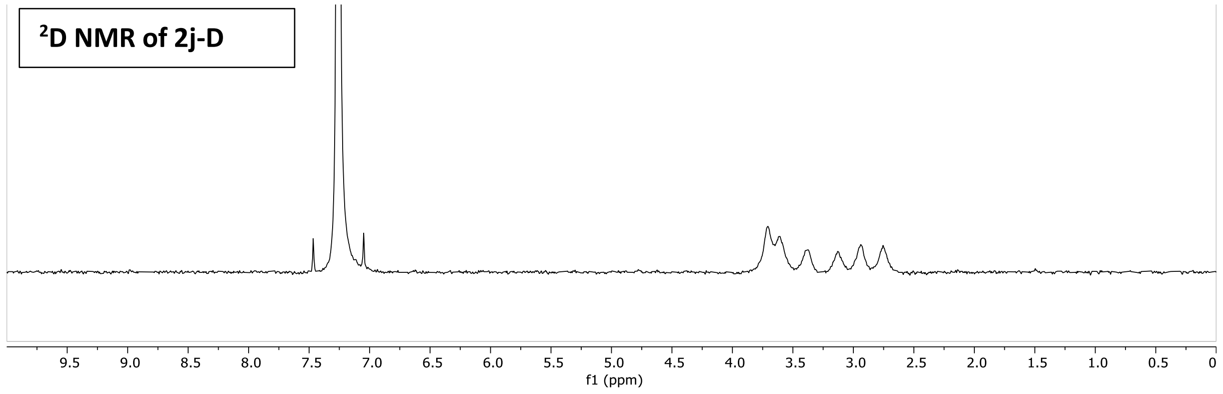

NMR Spectra

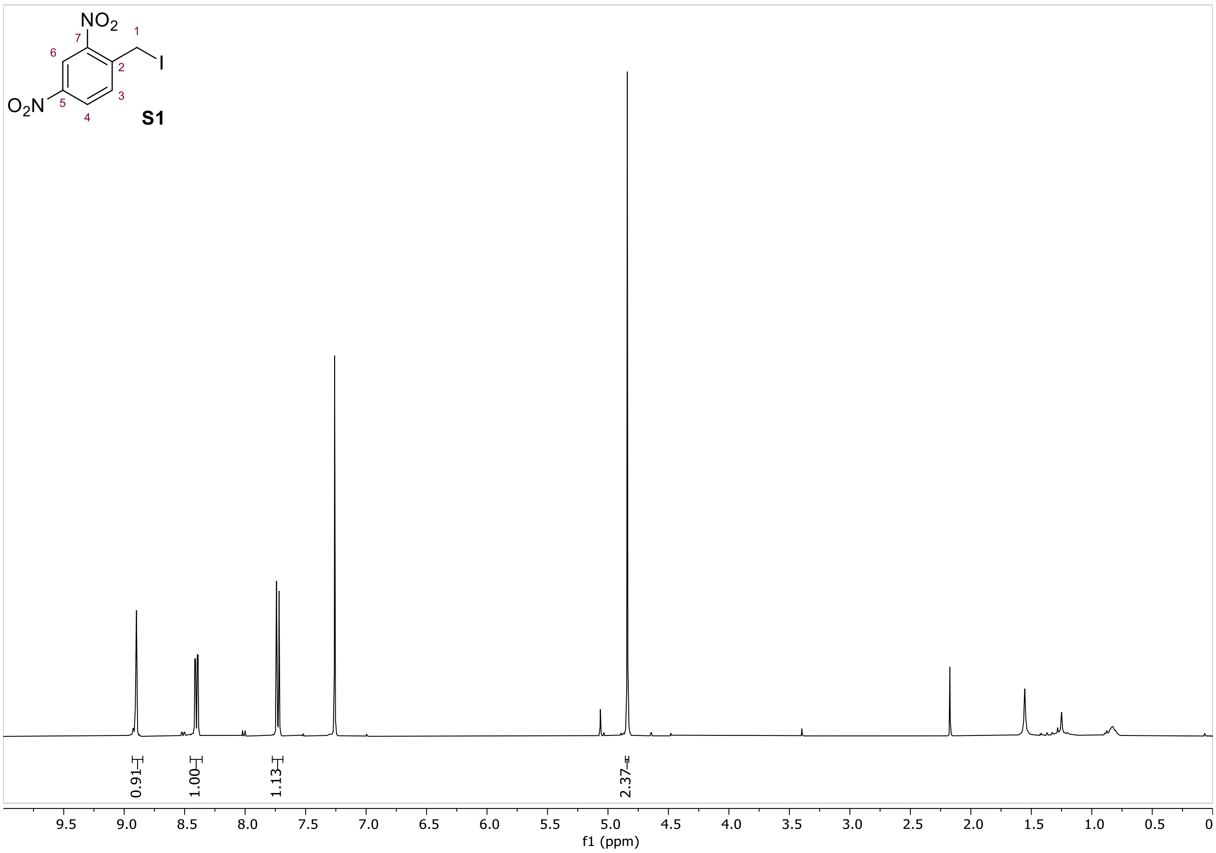
NO2
O2N
1
2
3
4
5
6
7
I
S1
0.91
1.00
1.13
2.37
9.5
9.0
8.5
8.0
7.5
7.0
6.5
6.0
5.5
5.0
4.5
4.0
3.5
3.0
2.5
2.0
1.5
1.0
0.5
0
f1 (ppm)

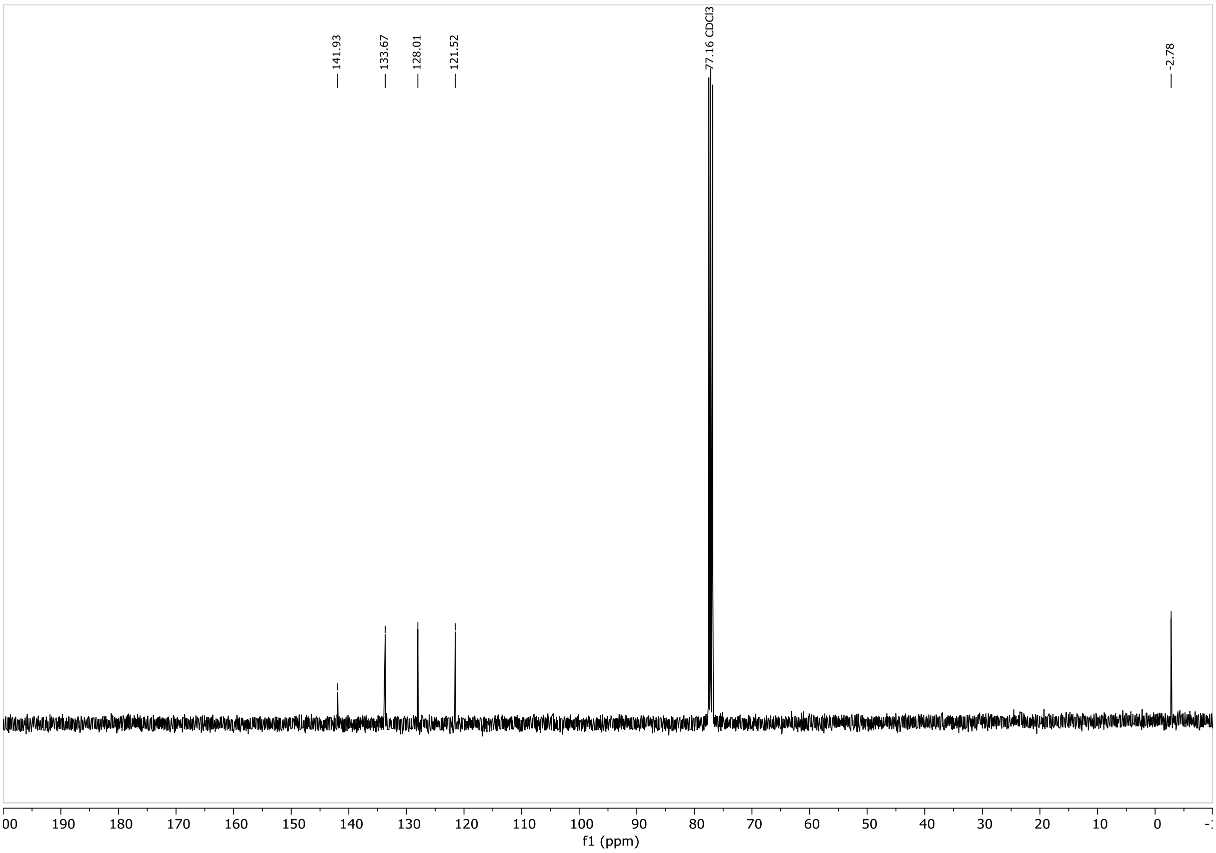
141.93
133.67
128.01
121.52
77.16 CDCl3
-2.78
190
180
170
160
150
140
130
120
110
100
90
80
70
60
50
40
30
20
10
0
f1 (ppm)

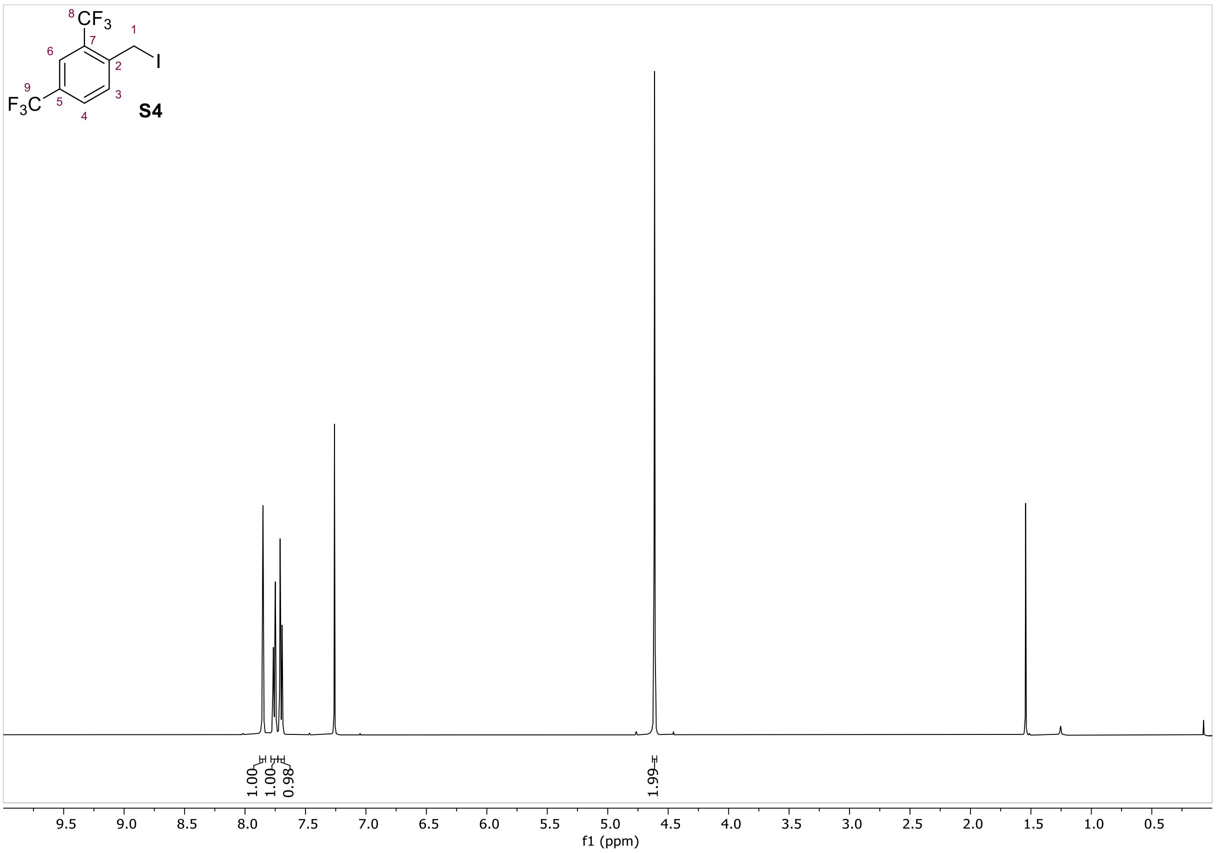

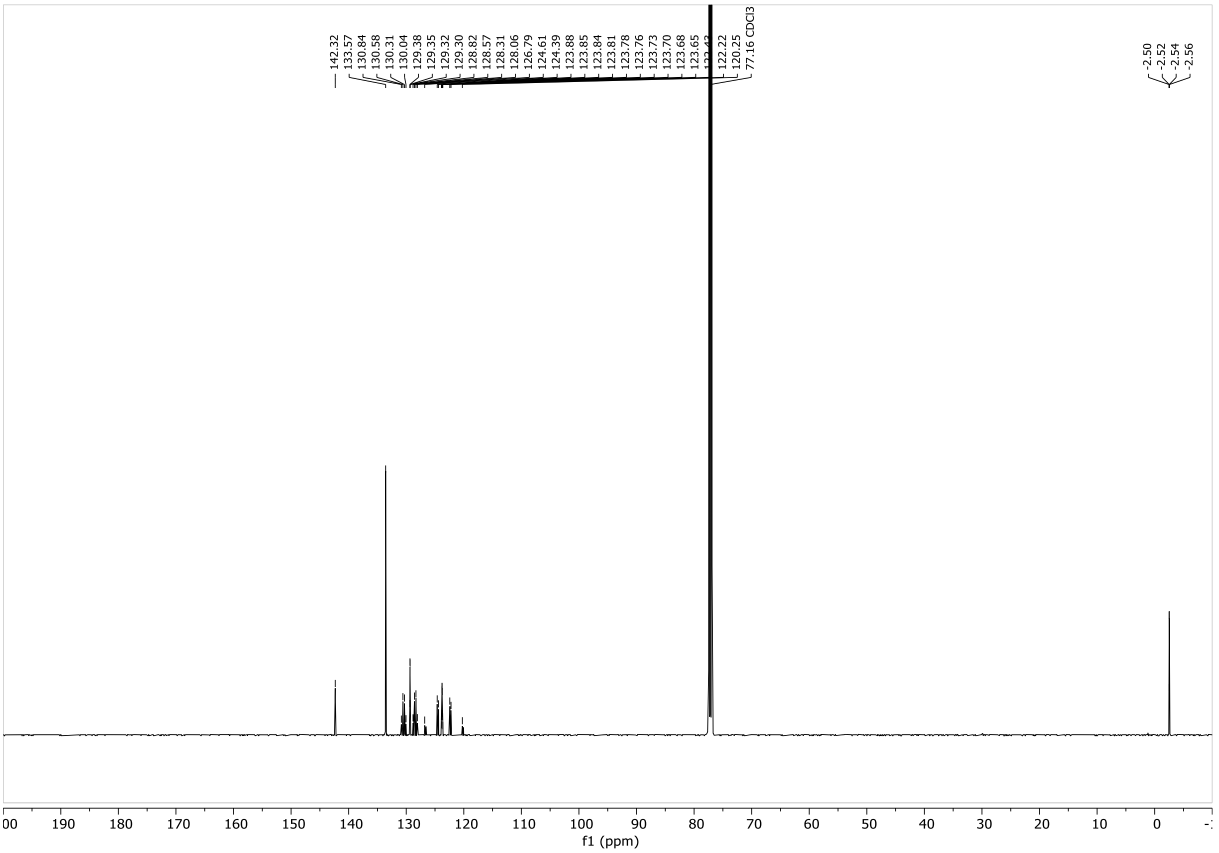

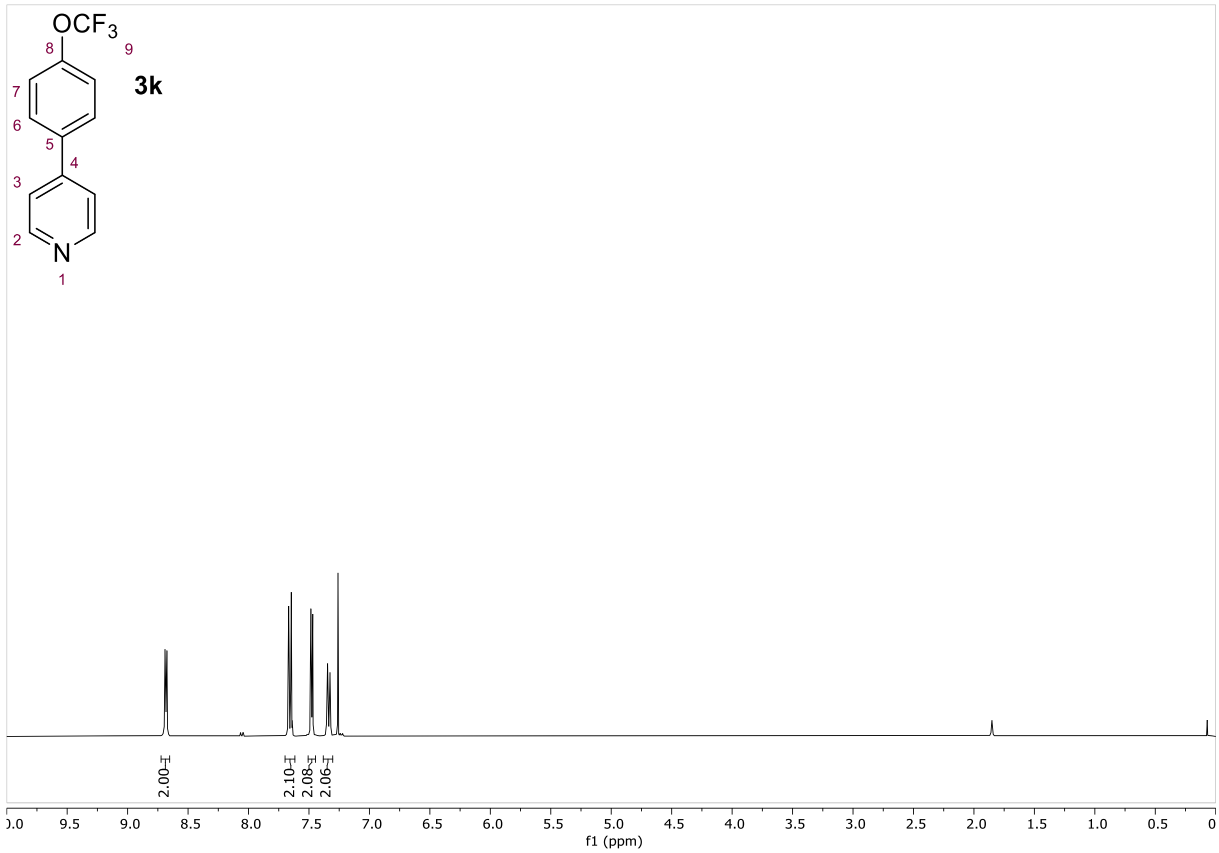
OCF3
3k
2.00
2.10
2.08
2.06
f1 (ppm)

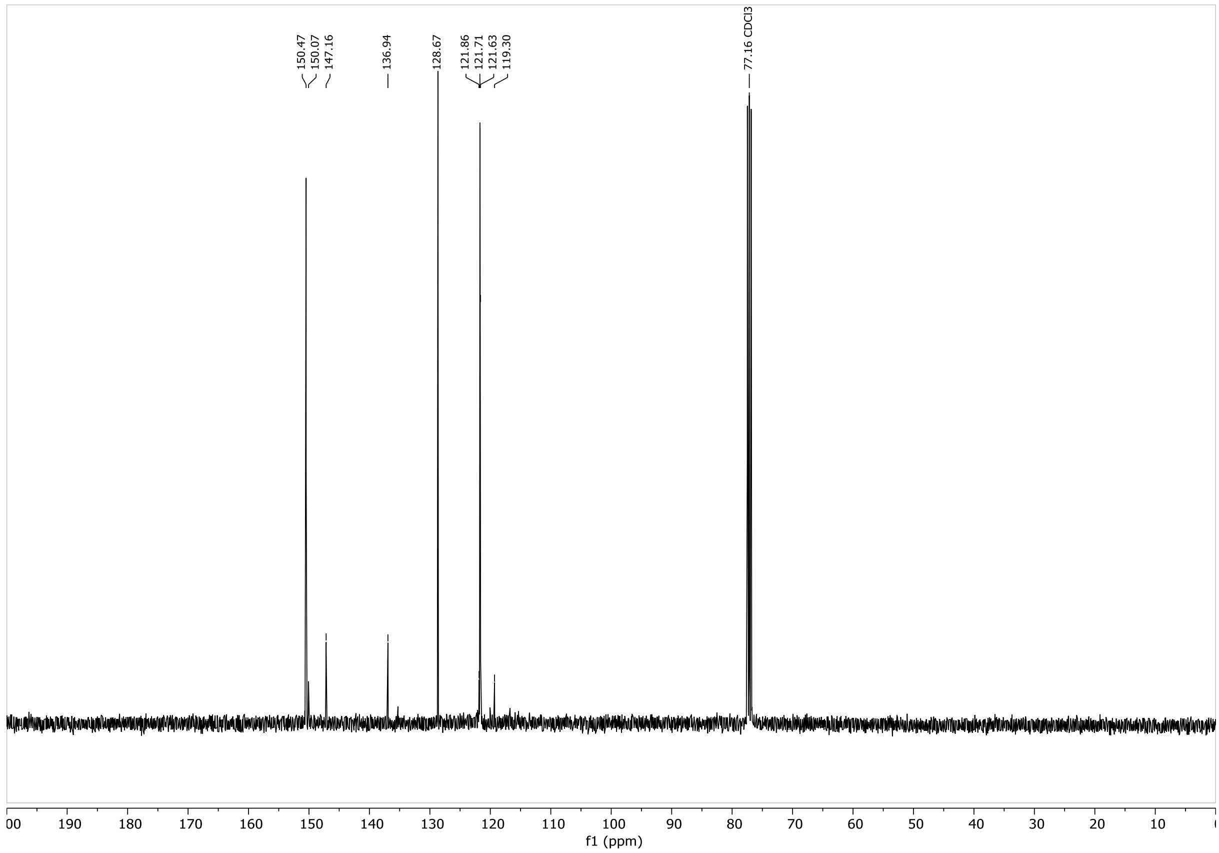
150.47
150.07
147.16
136.94
128.67
121.86
121.71
121.63
119.30
77.16 CDCl3
f1 (ppm)

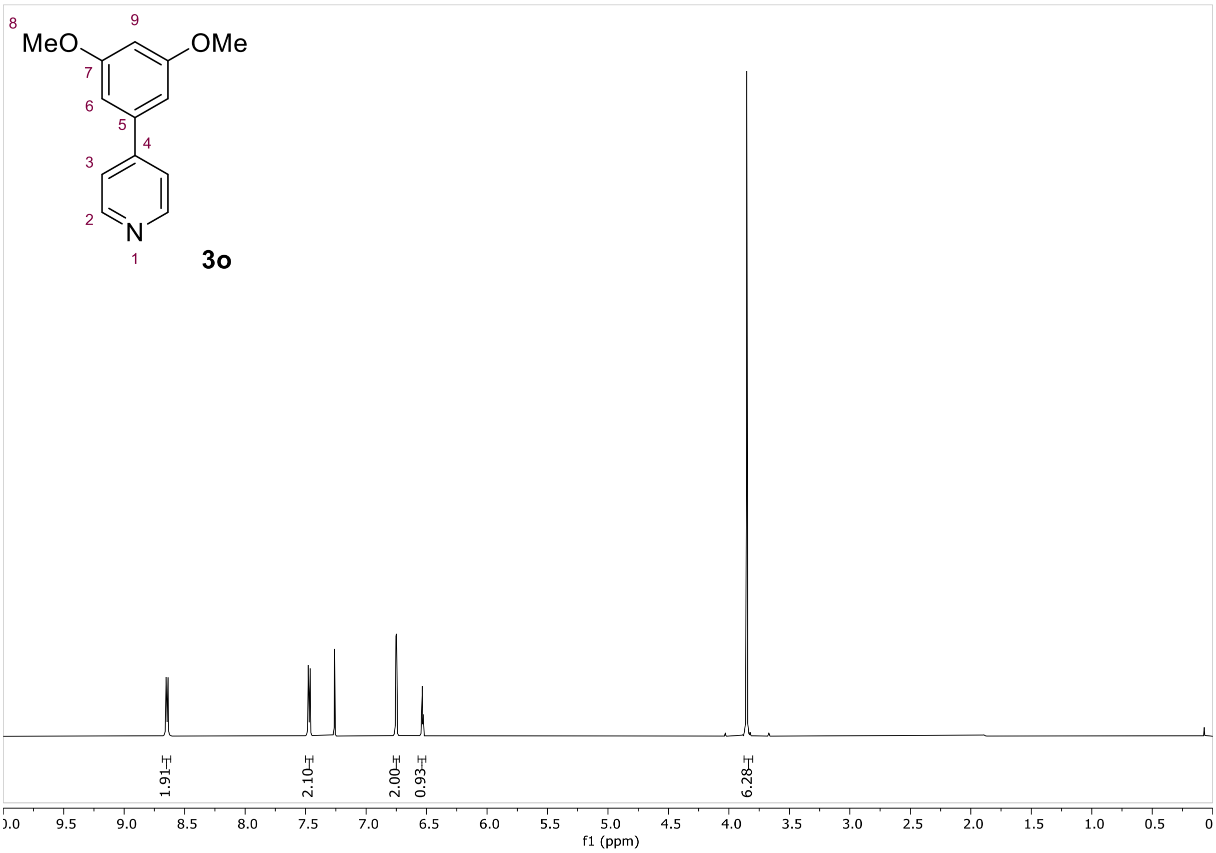
MeO
OMe
N
3o
1
2
3
4
5
6
7
8
9
1.91
2.10
2.00
0.93
6.28
f1 (ppm)

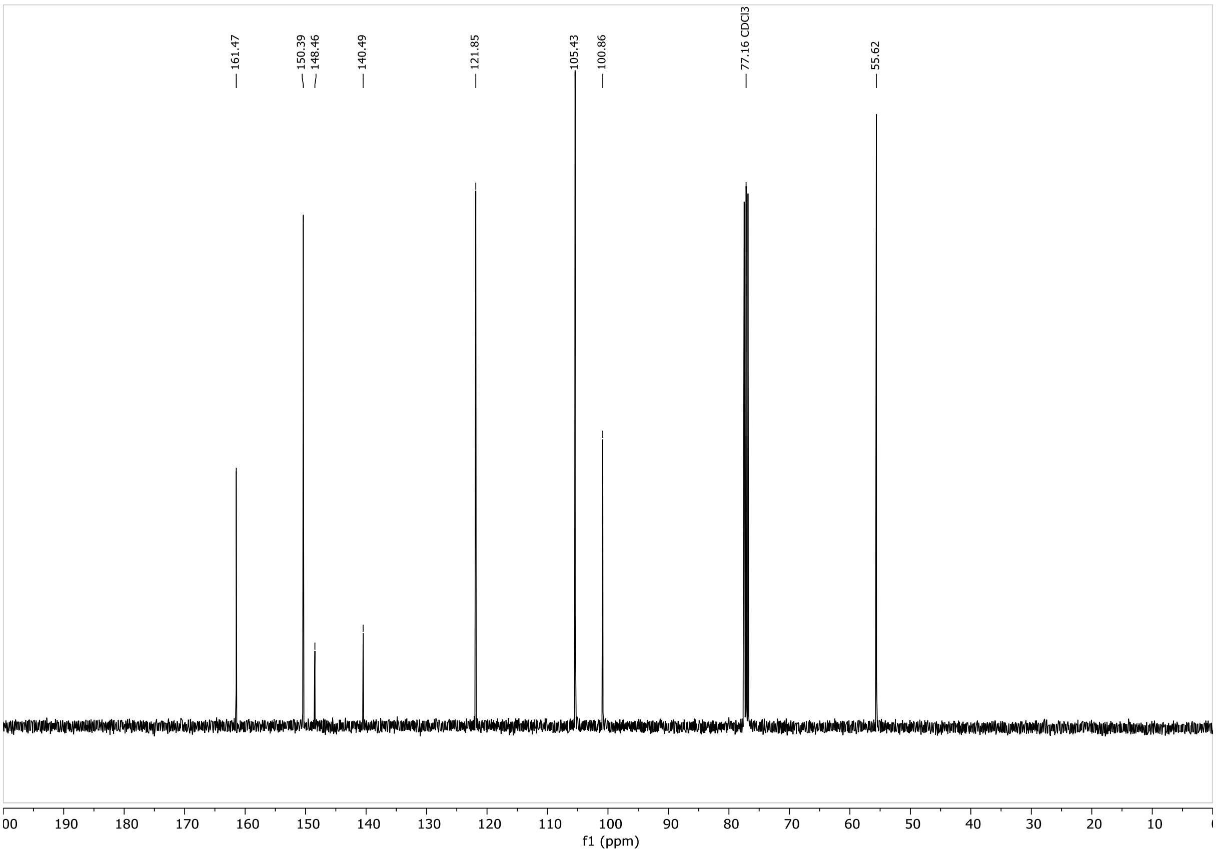
161.47
150.39
148.46
140.49
121.85
105.43
100.86
77.16 CDCl3
55.62
f1 (ppm)

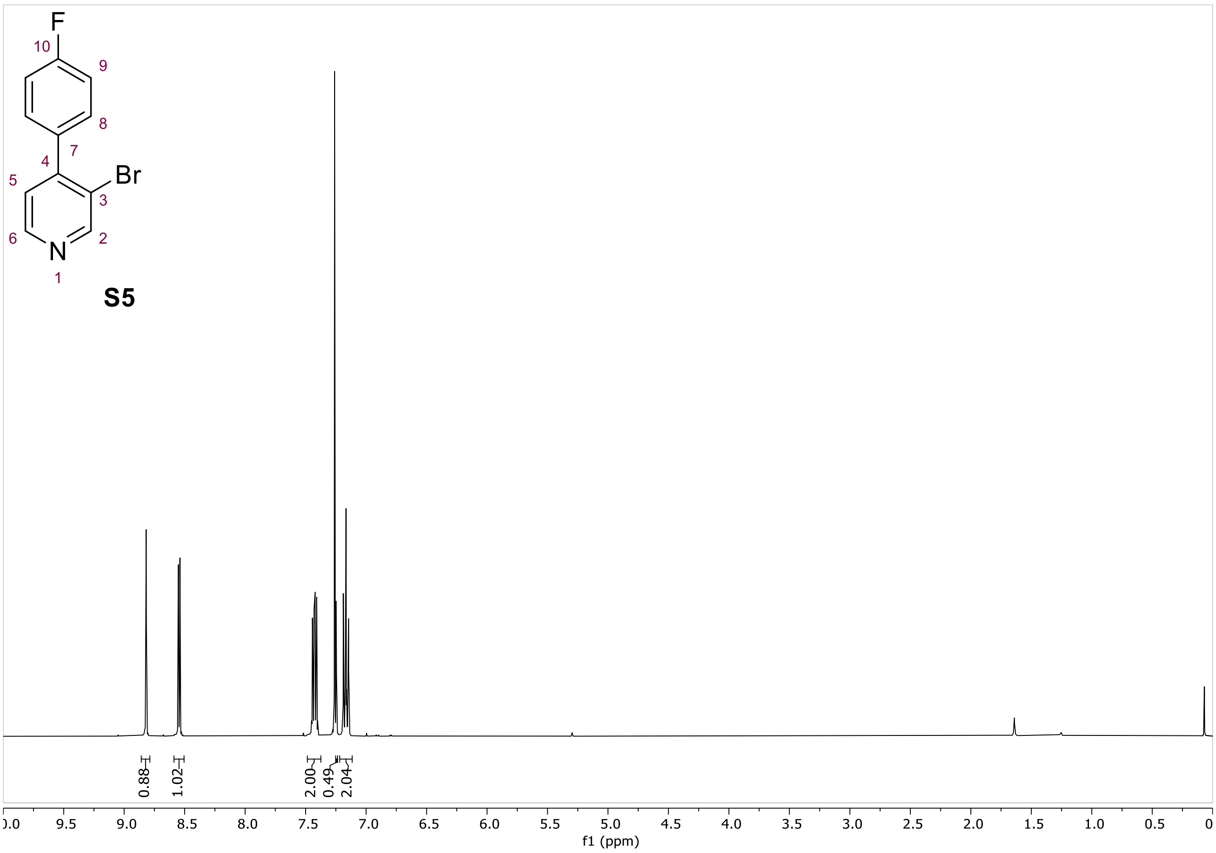
F
Br
N
1
2
3
4
5
6
7
8
9
10
S5
0.88
1.02
2.00
0.49
2.04
f1 (ppm)

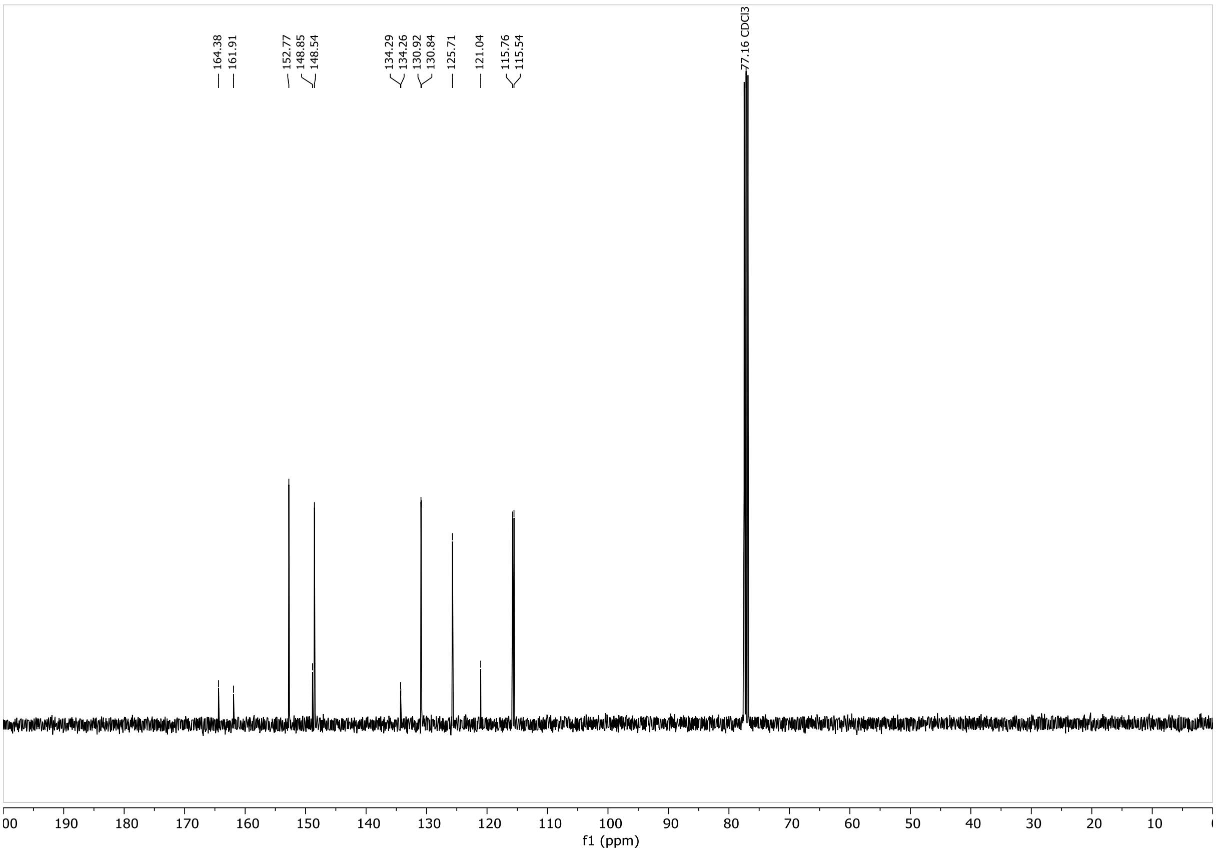
164.38
161.91
152.77
148.85
148.54
134.29
134.26
130.92
130.84
125.71
121.04
115.76
115.54
77.16 CDCl3
f1 (ppm)

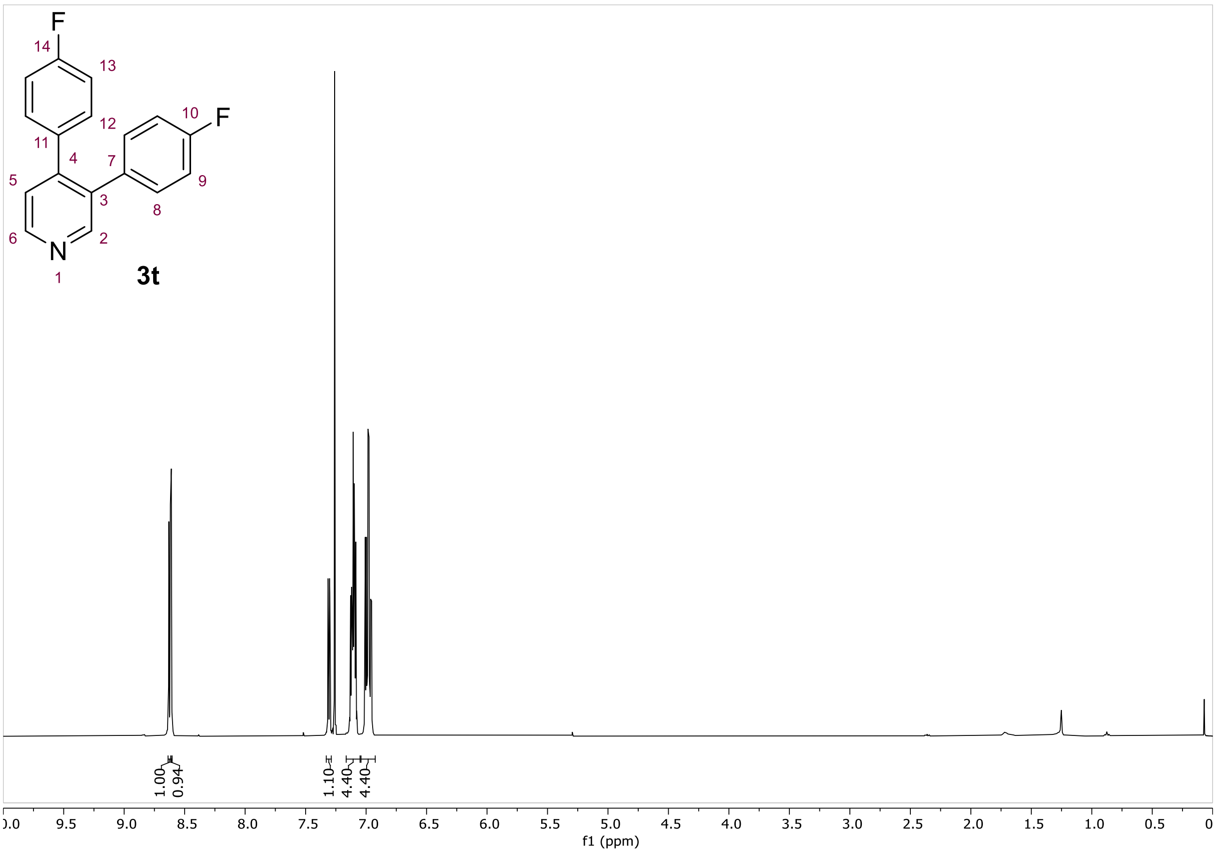

3t
f1 (ppm)

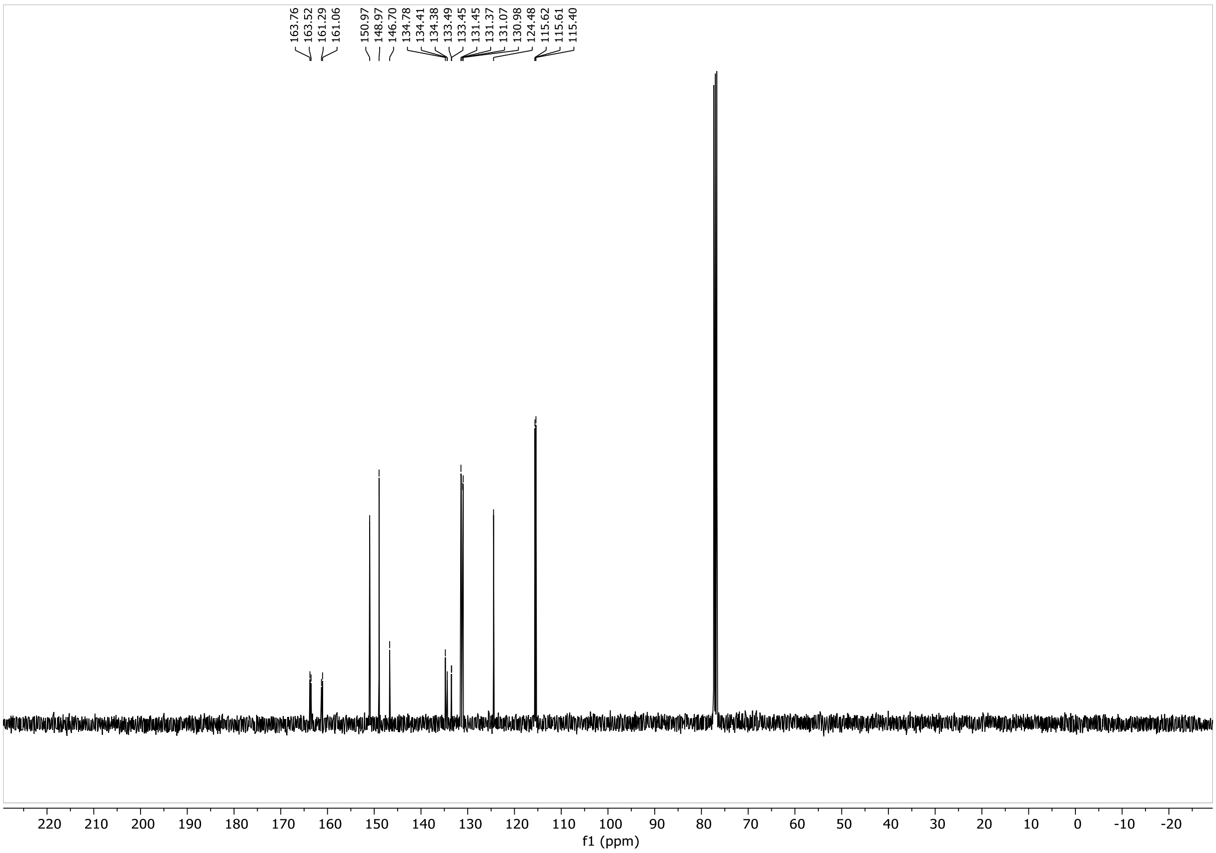

163.76
163.52
161.29
161.06
150.97
148.97
146.70
134.78
134.41
134.38
133.49
133.45
131.45
131.37
131.07
130.98
124.48
115.62
115.61
115.40
f1 (ppm)

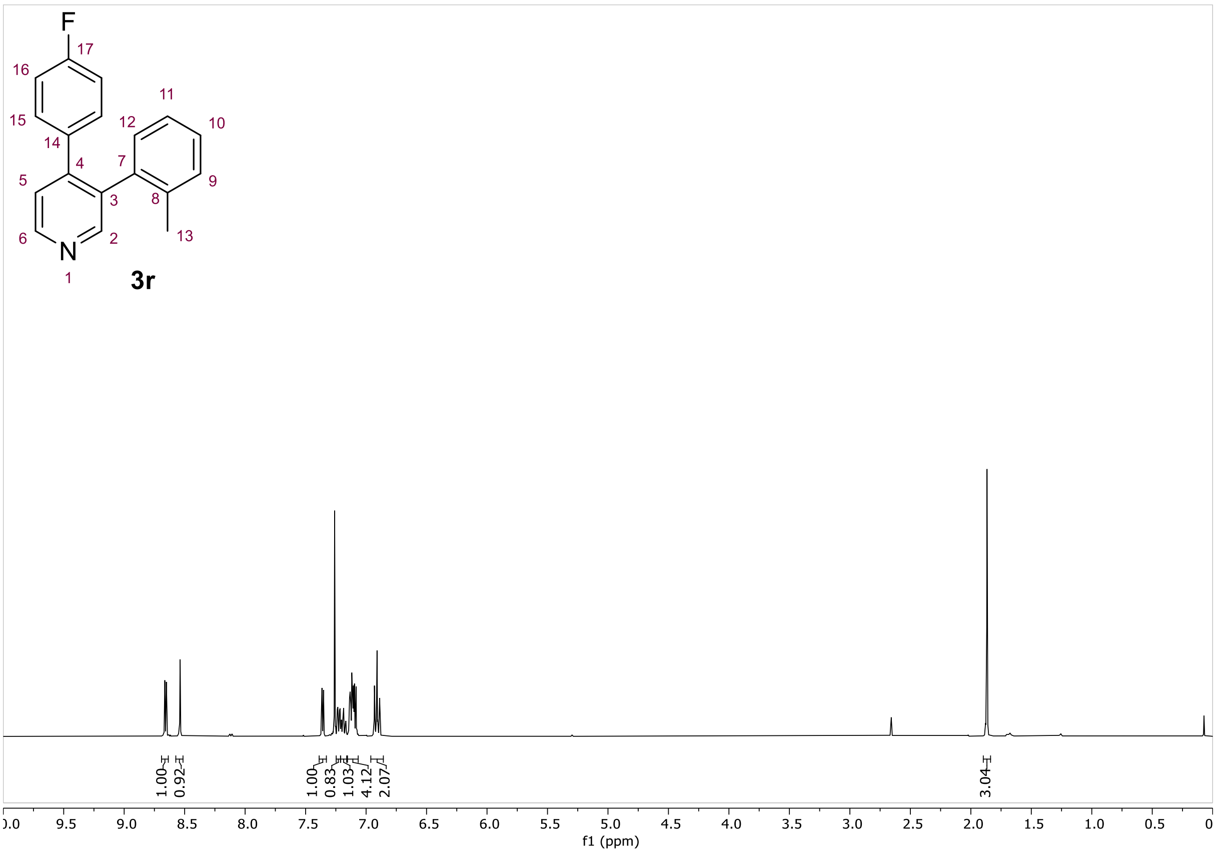

F
17
16
15
14
4
5
6
N
1
2
3
7
8
9
10
11
12
13
3r
1.00
0.92
1.00
0.83
1.03
4.12
2.07
3.04
0.0
9.5
9.0
8.5
8.0
7.5
7.0
6.5
6.0
5.5
5.0
4.5
4.0
3.5
3.0
2.5
2.0
1.5
1.0
0.5
0
f1 (ppm)

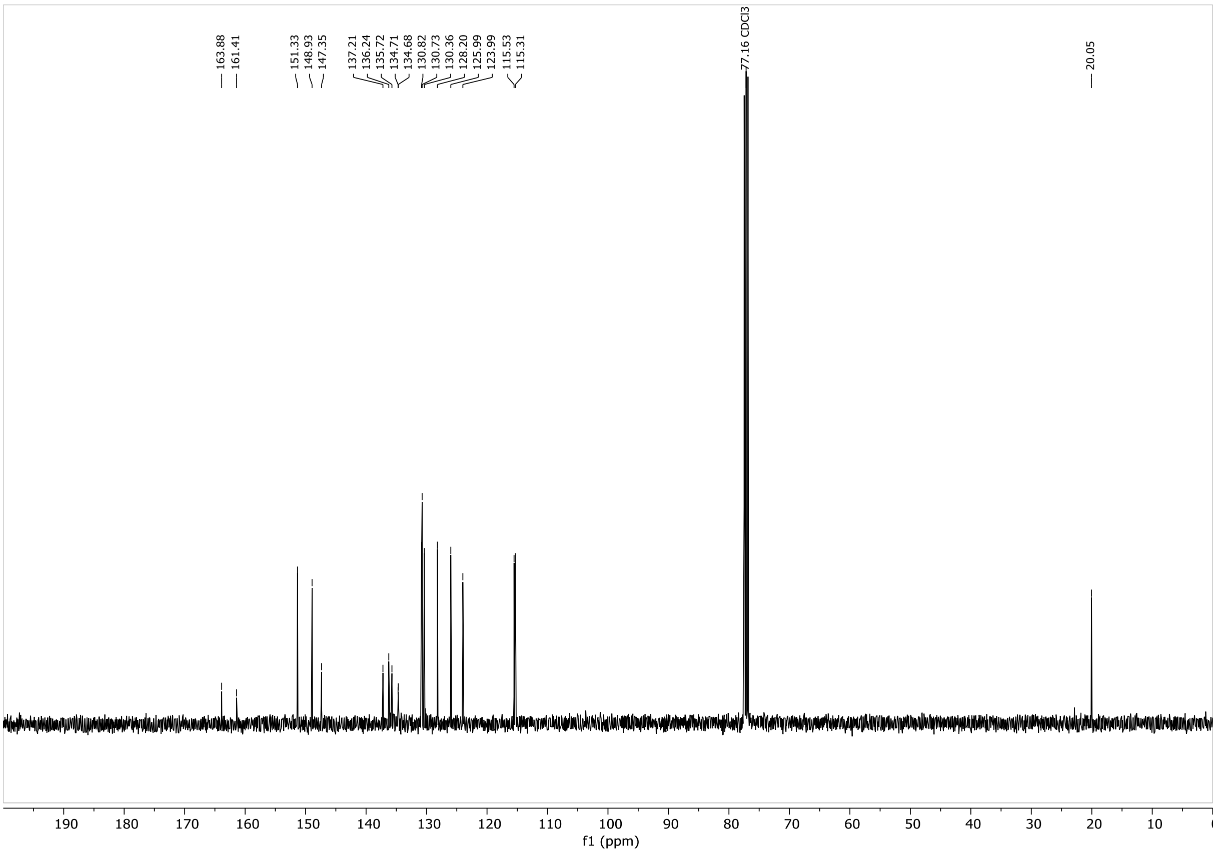

163.88
161.41
151.33
148.93
147.35
137.21
136.24
135.72
134.71
134.68
130.82
130.73
130.36
128.20
125.99
123.99
115.53
115.31
77.16 CDCl3
20.05
190
180
170
160
150
140
130
120
110
100
90
80
70
60
50
40
30
20
10
f1 (ppm)

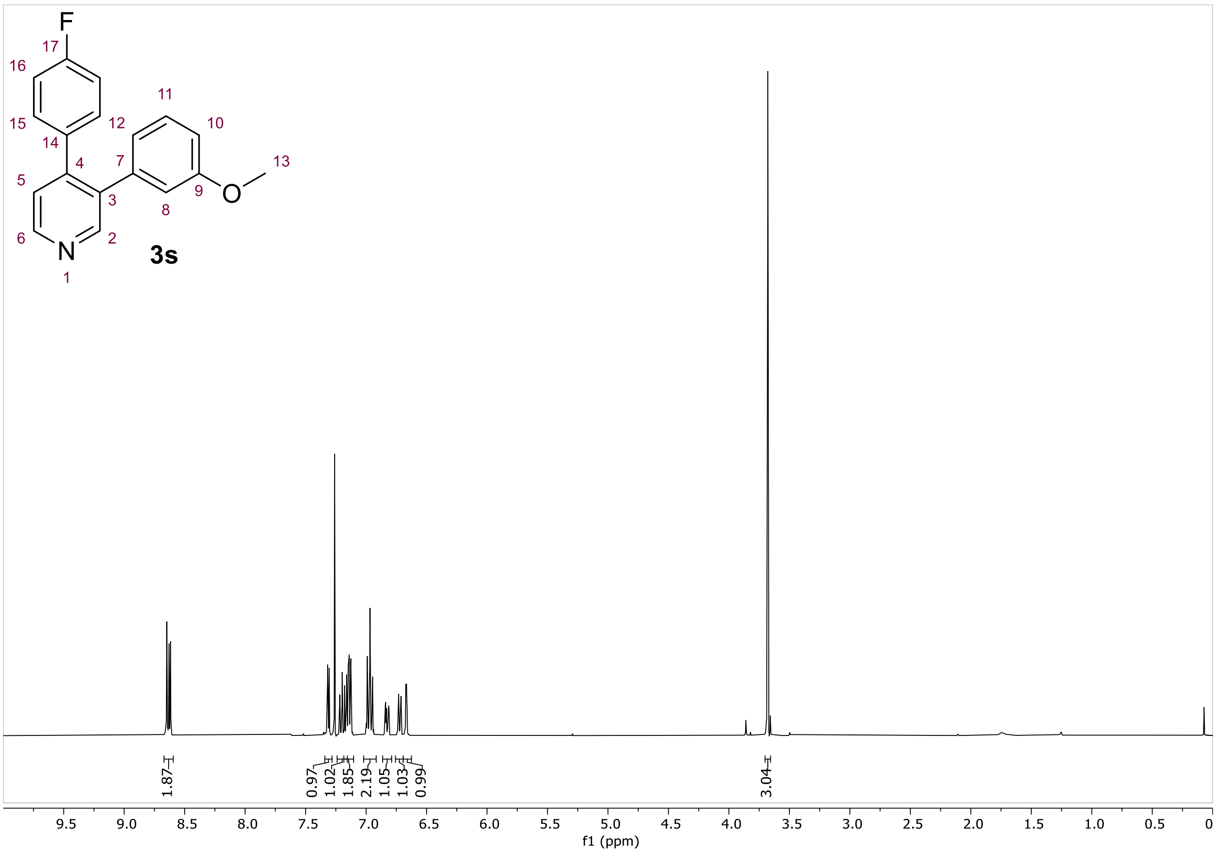

3s
1.87
0.97
1.02
1.85
2.19
1.05
1.03
0.99
3.04
f1 (ppm)

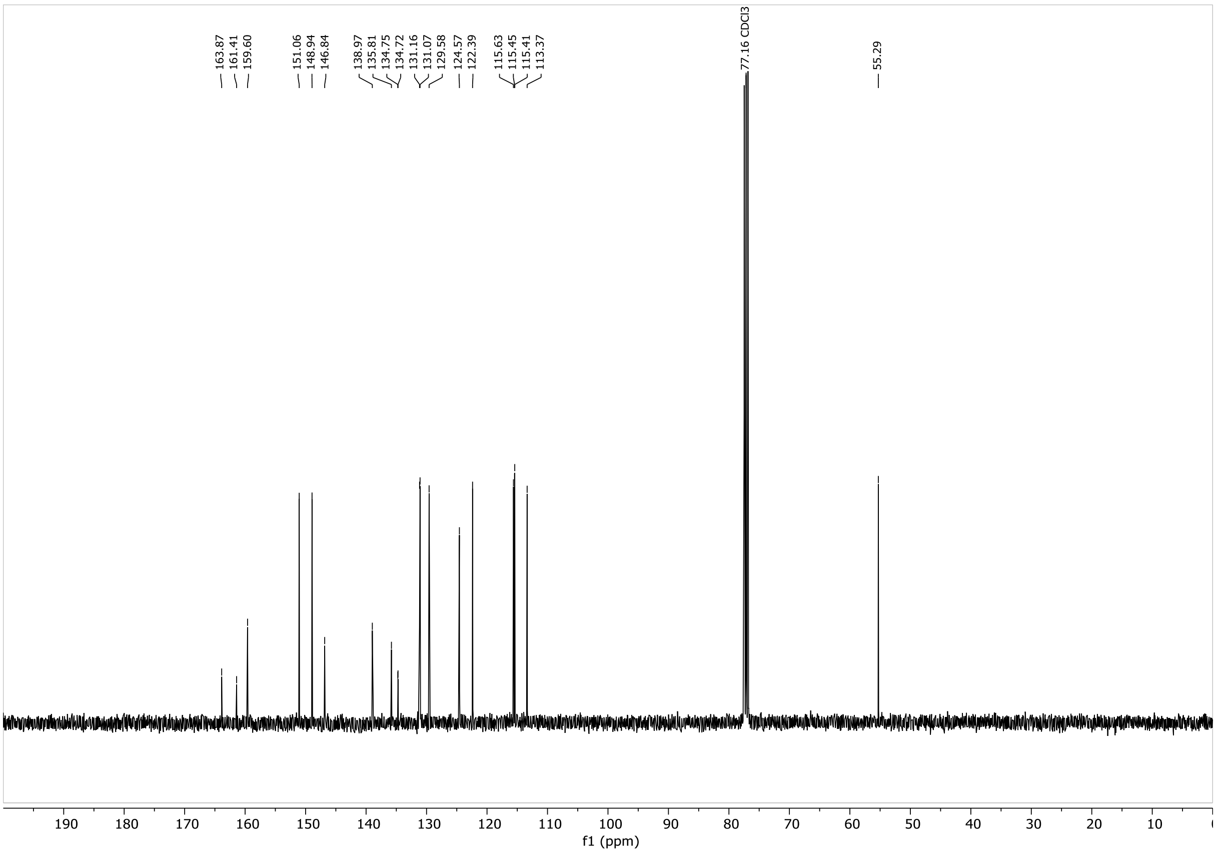

163.87
161.41
159.60
151.06
148.94
146.84
138.97
135.81
134.75
134.72
131.16
131.07
129.58
124.57
122.39
115.63
115.45
115.41
113.37
77.16 CDCl3
55.29
f1 (ppm)

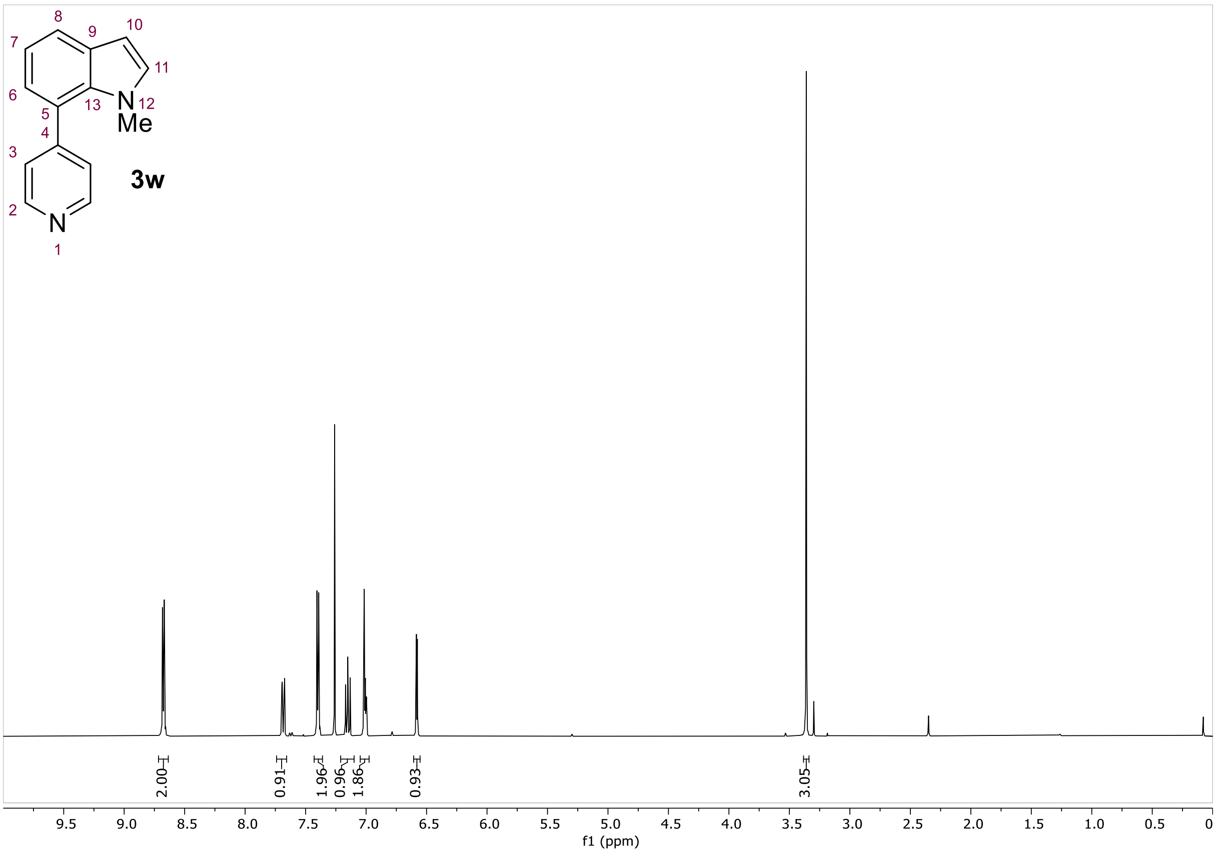

3w
2.00
0.91
1.96
0.96
1.86
0.93
3.05
9.5
9.0
8.5
8.0
7.5
7.0
6.5
6.0
5.5
5.0
4.5
4.0
3.5
3.0
2.5
2.0
1.5
1.0
0.5
0
f1 (ppm)

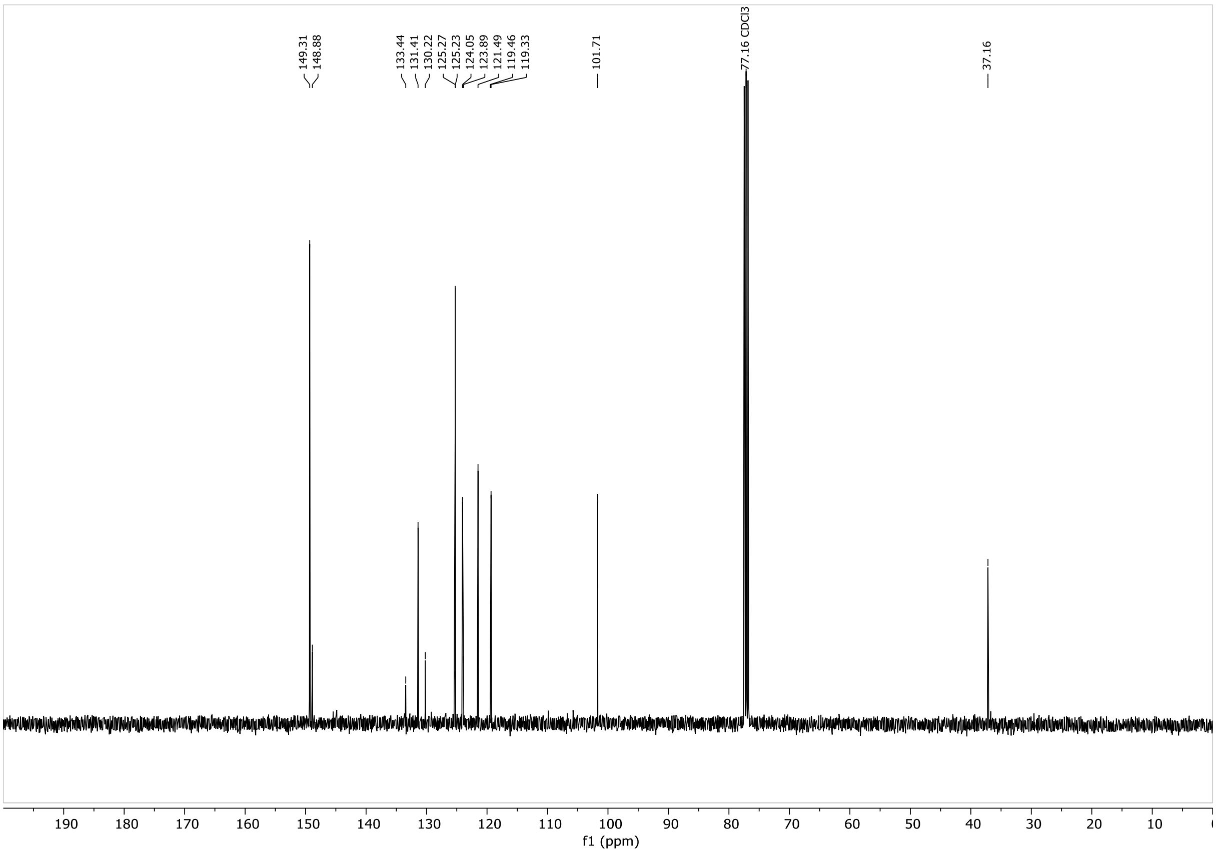

149.31
148.88
133.44
131.41
130.22
125.27
125.23
124.05
123.89
121.49
119.46
119.33
101.71
77.16 CDCl3
37.16
190
180
170
160
150
140
130
120
110
100
90
80
70
60
50
40
30
20
10
f1 (ppm)

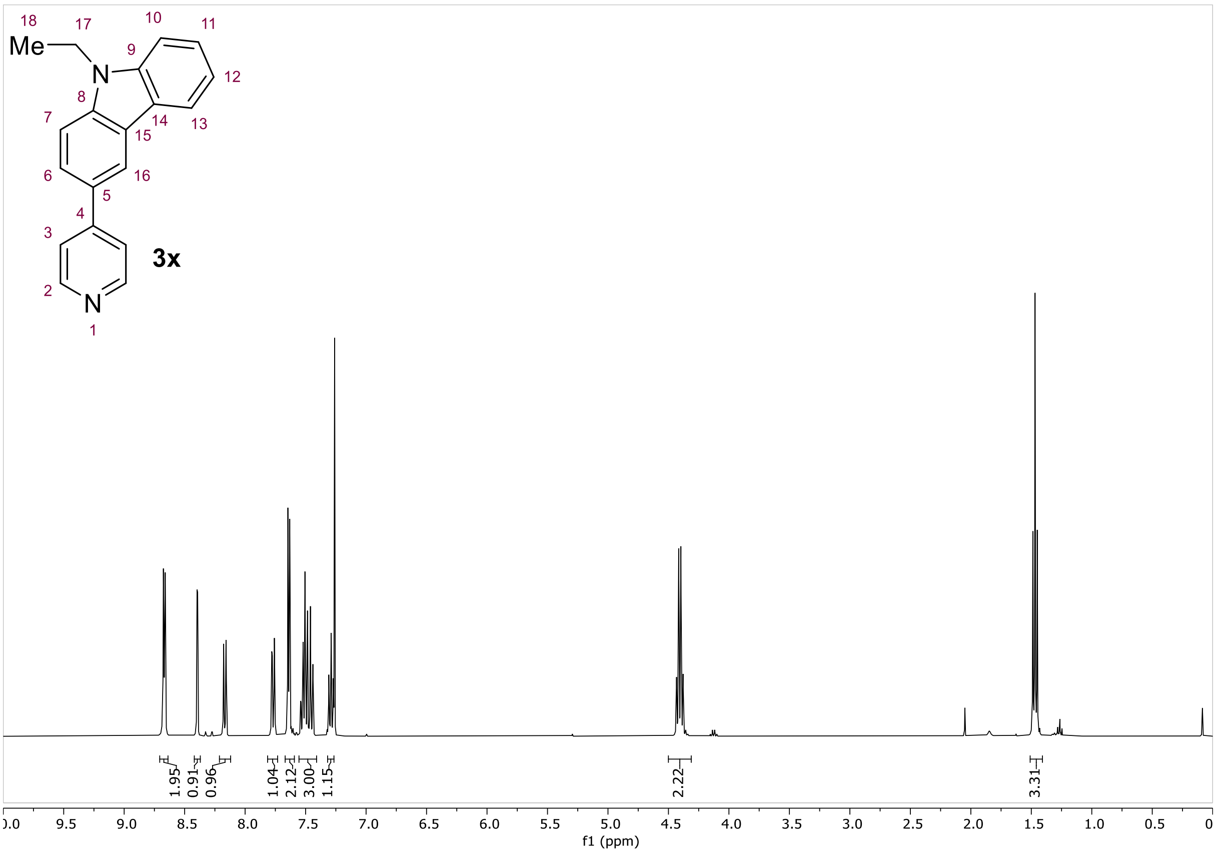

18
17
10
11
Me
N
9
12
8
7
14
13
15
6
16
5
4
3
3x
2
N
1
1.95
0.91
0.96
1.04
2.12
3.00
1.15
2.22
3.31
f1 (ppm)

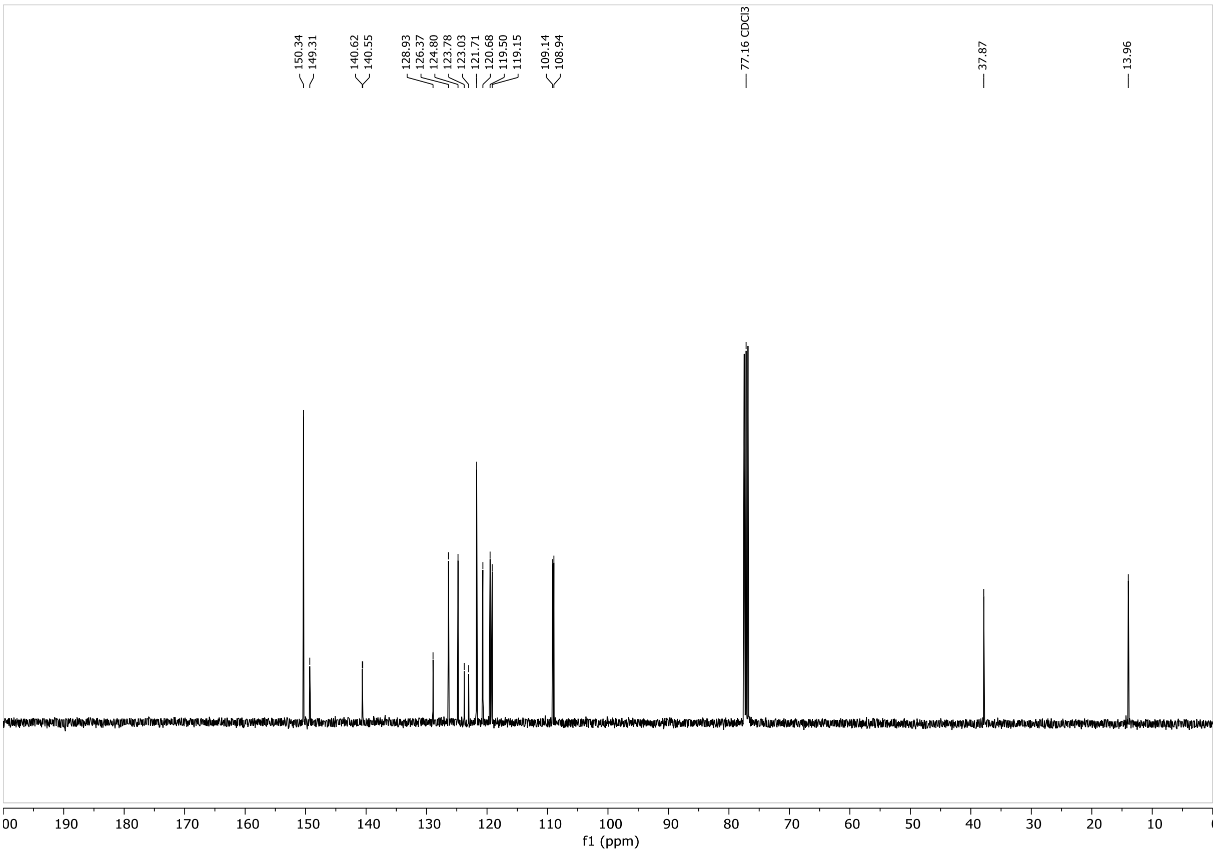

150.34
149.31
140.62
140.55
128.93
126.37
124.80
123.78
123.03
121.71
120.68
119.50
119.15
109.14
108.94
77.16 CDCl3
37.87
13.96
f1 (ppm)

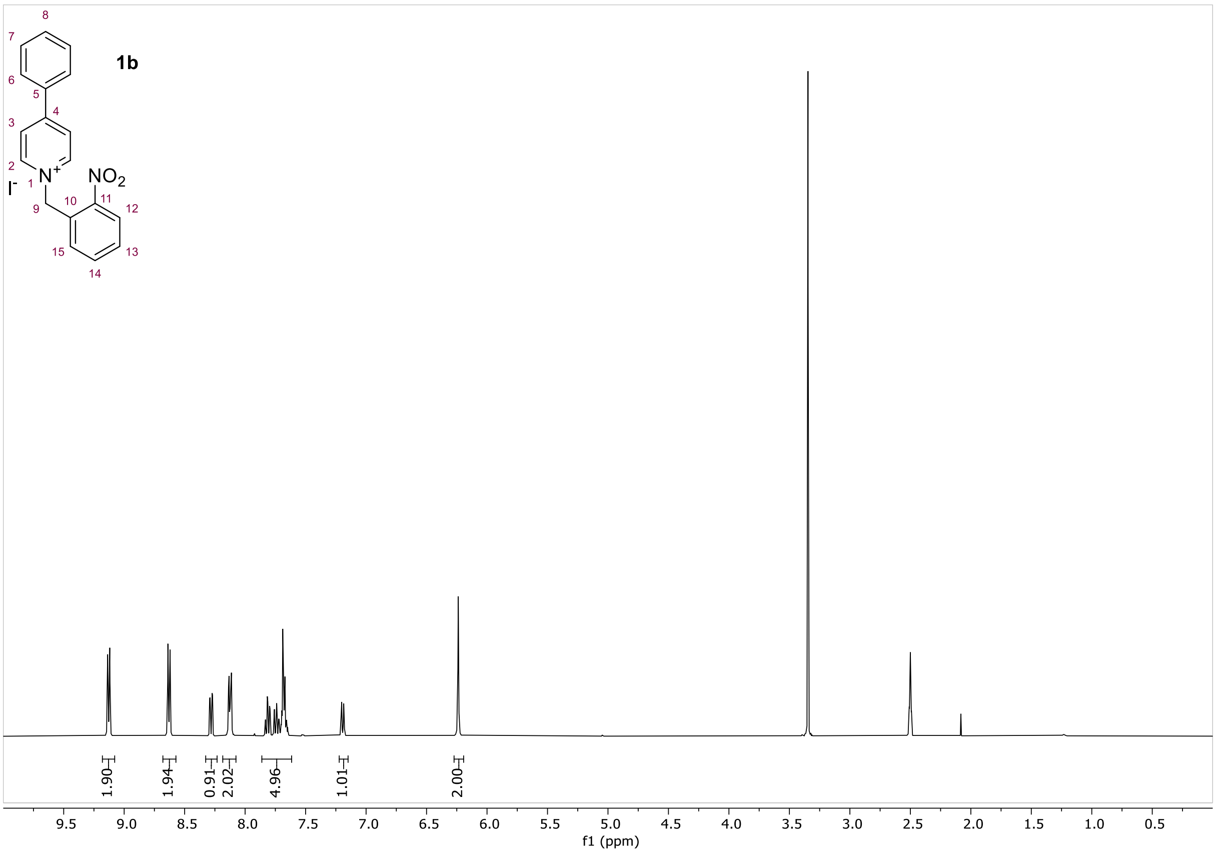
1b
NO2
I-
1.90
1.94
0.91
2.02
4.96
1.01
2.00
f1 (ppm)

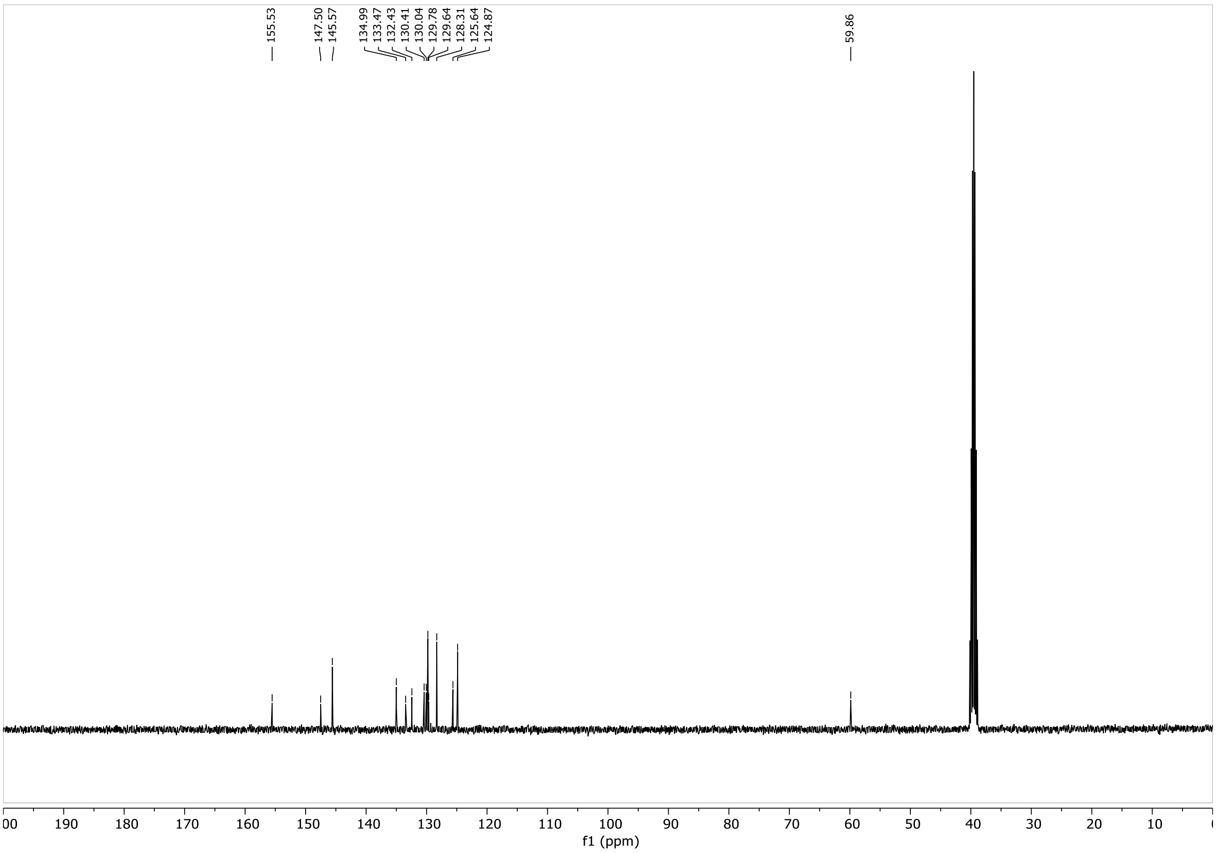
155.53
147.50
145.57
134.99
133.47
132.43
130.41
130.04
129.78
129.64
128.31
125.64
124.87
59.86
f1 (ppm)

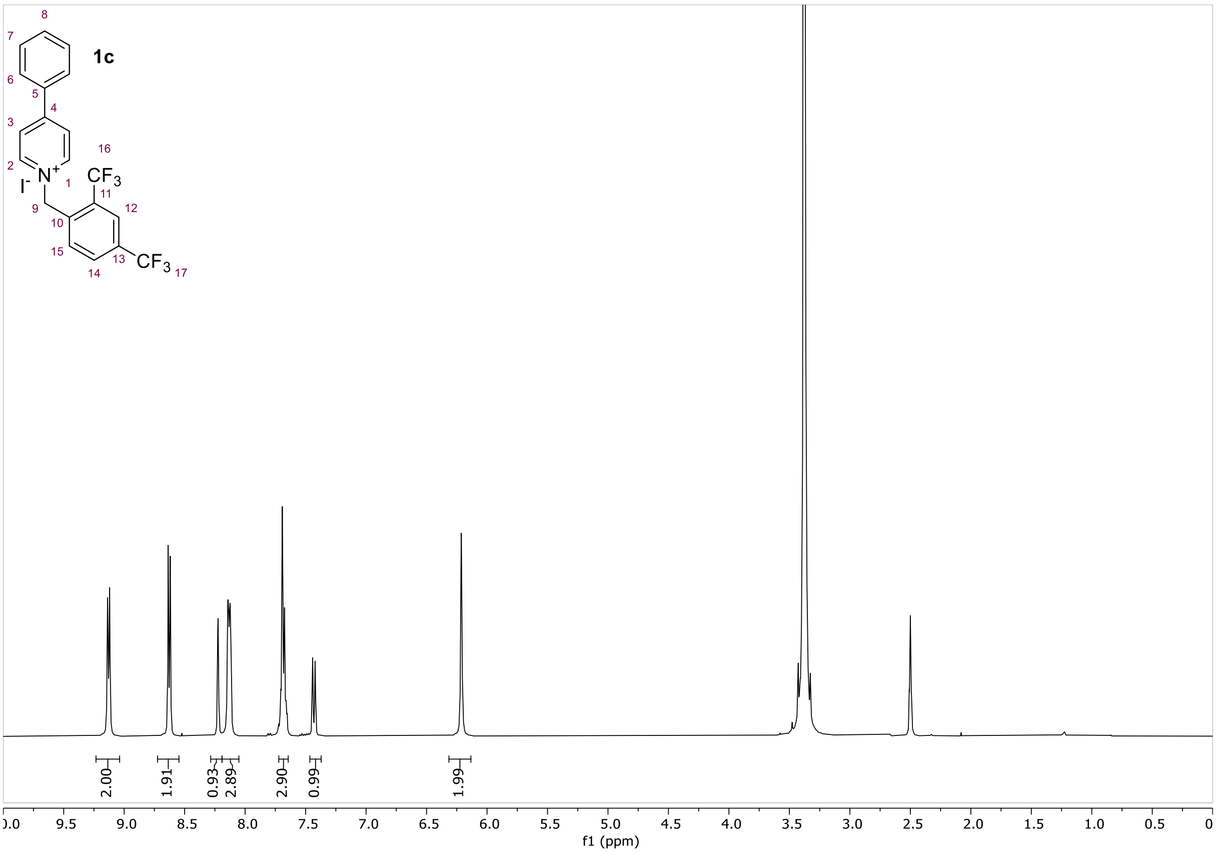
1c
CF3
CF3
I-
N+
2.00
1.91
0.93
2.89
2.90
0.99
1.99
f1 (ppm)

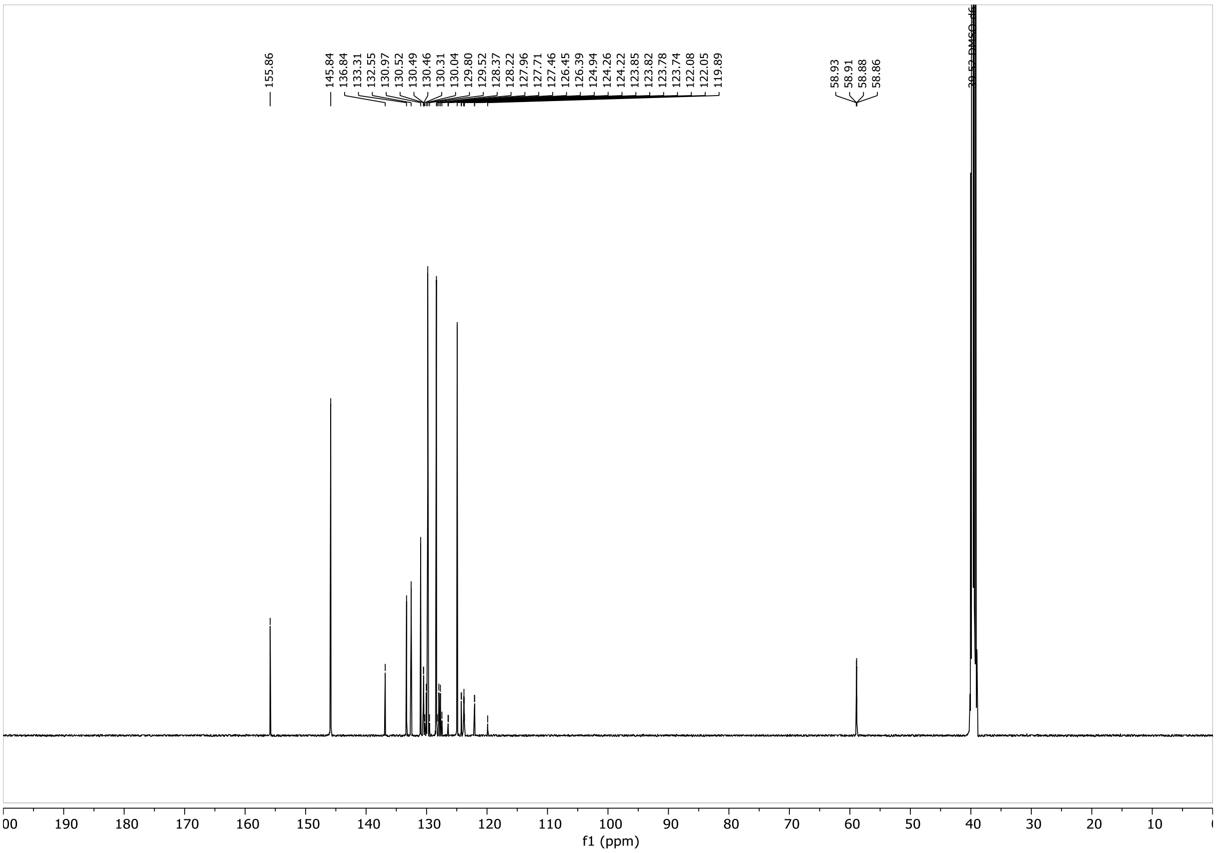
155.86
145.84
136.84
133.31
132.55
130.97
130.52
130.49
130.46
130.31
130.04
129.80
129.52
128.37
128.22
127.96
127.71
127.46
126.45
126.39
124.94
124.26
124.22
123.85
123.82
123.78
123.74
122.08
122.05
119.89
58.93
58.91
58.88
58.86
f1 (ppm)

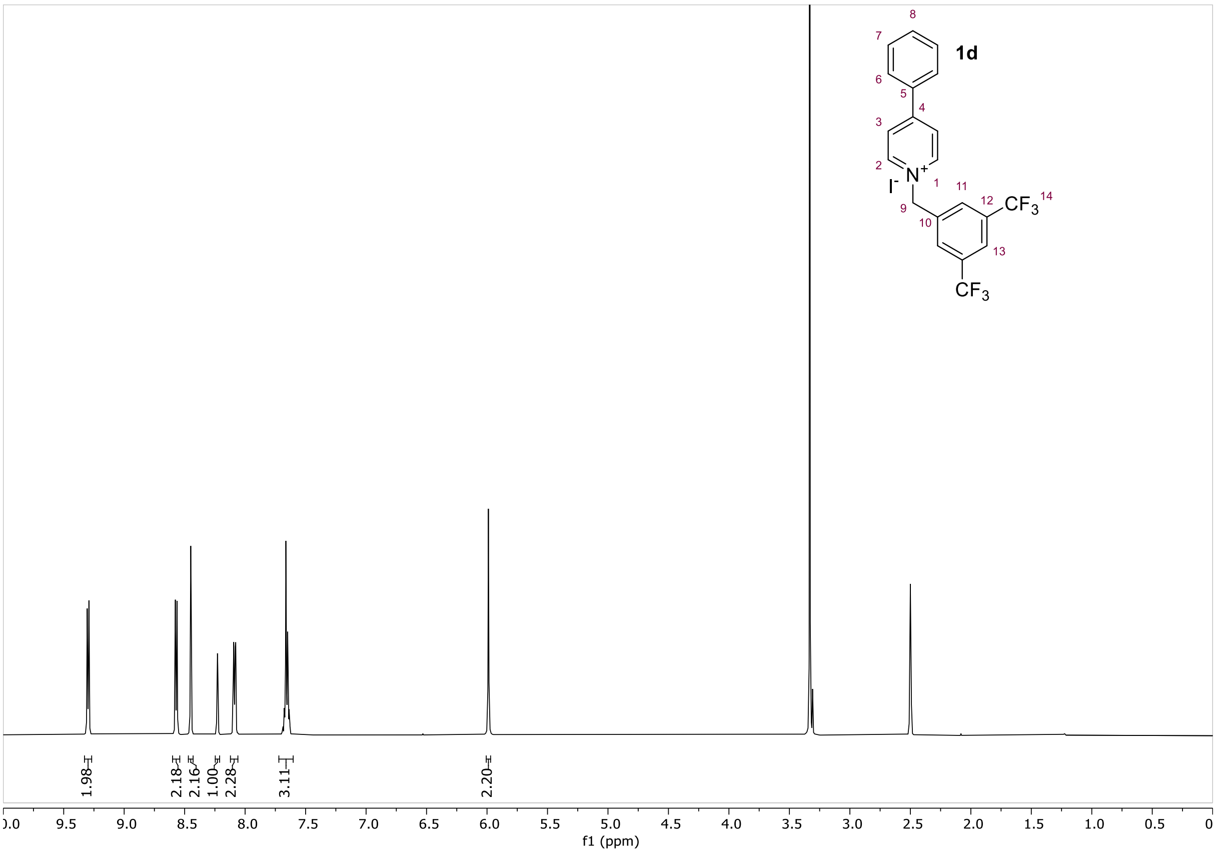
1d
1.98
2.18
2.16
1.00
2.28
3.11
2.20
f1 (ppm)

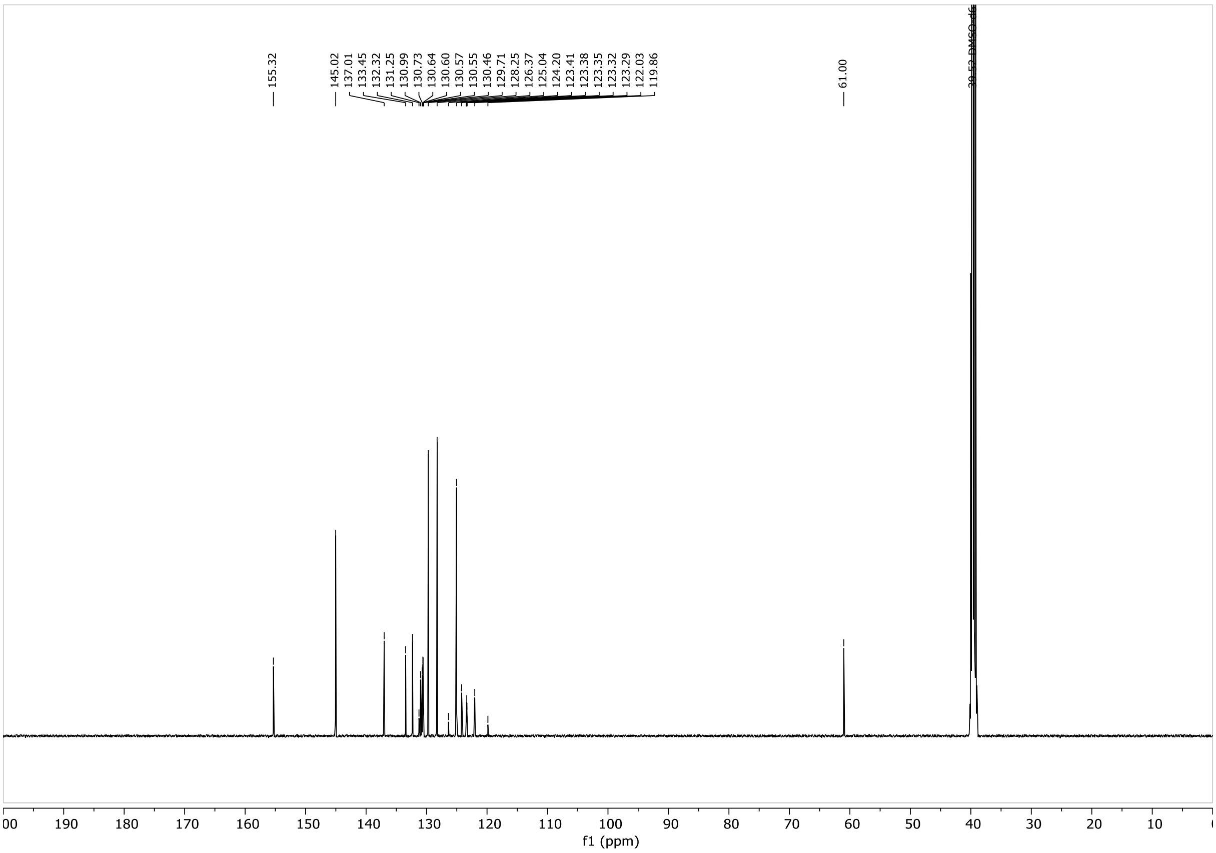
155.32
145.02
137.01
133.45
132.32
131.25
130.99
130.73
130.64
130.60
130.57
130.55
130.46
129.71
128.25
126.37
125.04
124.20
123.41
123.38
123.35
123.32
123.29
122.03
119.86
61.00
f1 (ppm)

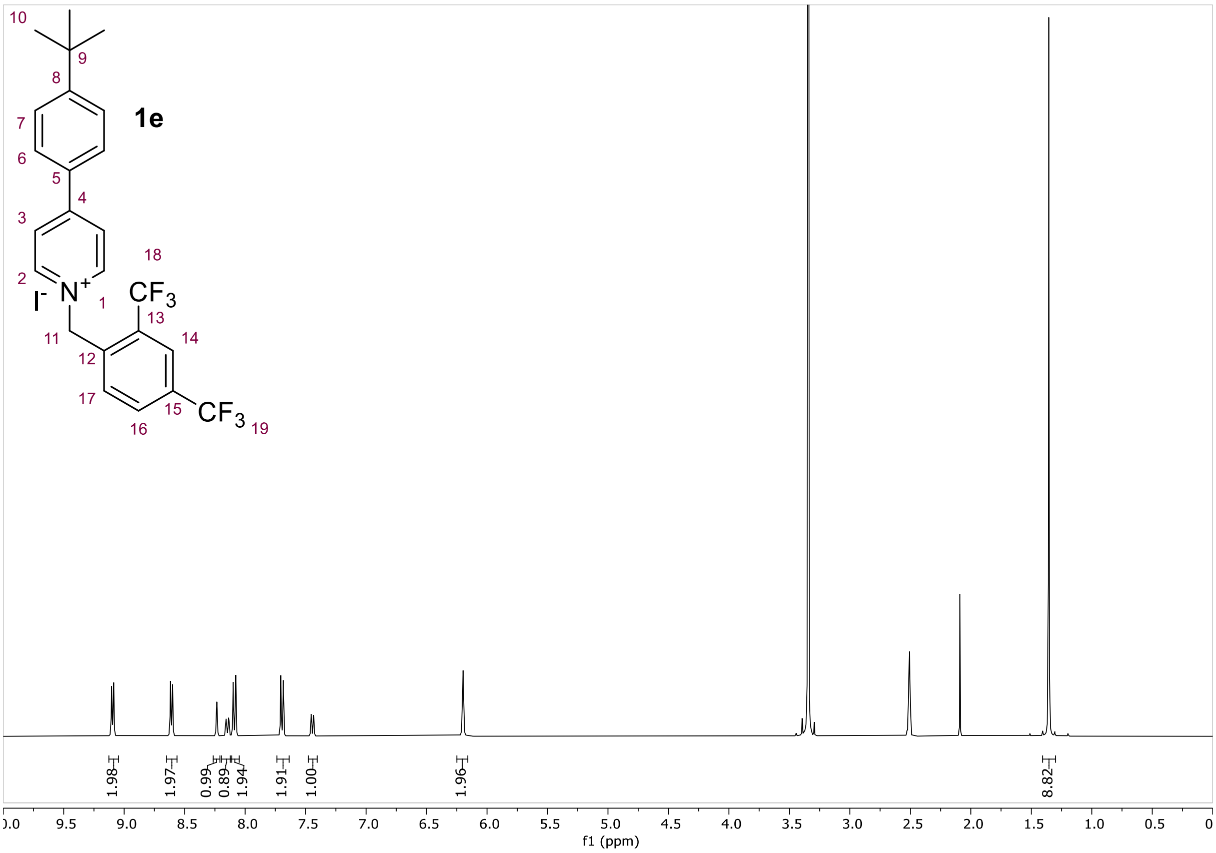

1e
10
9
8
7
6
5
4
3
2
1
18
CF3
13
14
I- N+
11
12
17
15
16
CF3 19
1.98
1.97
0.99
0.89
1.94
1.91
1.00
1.96
8.82
f1 (ppm)

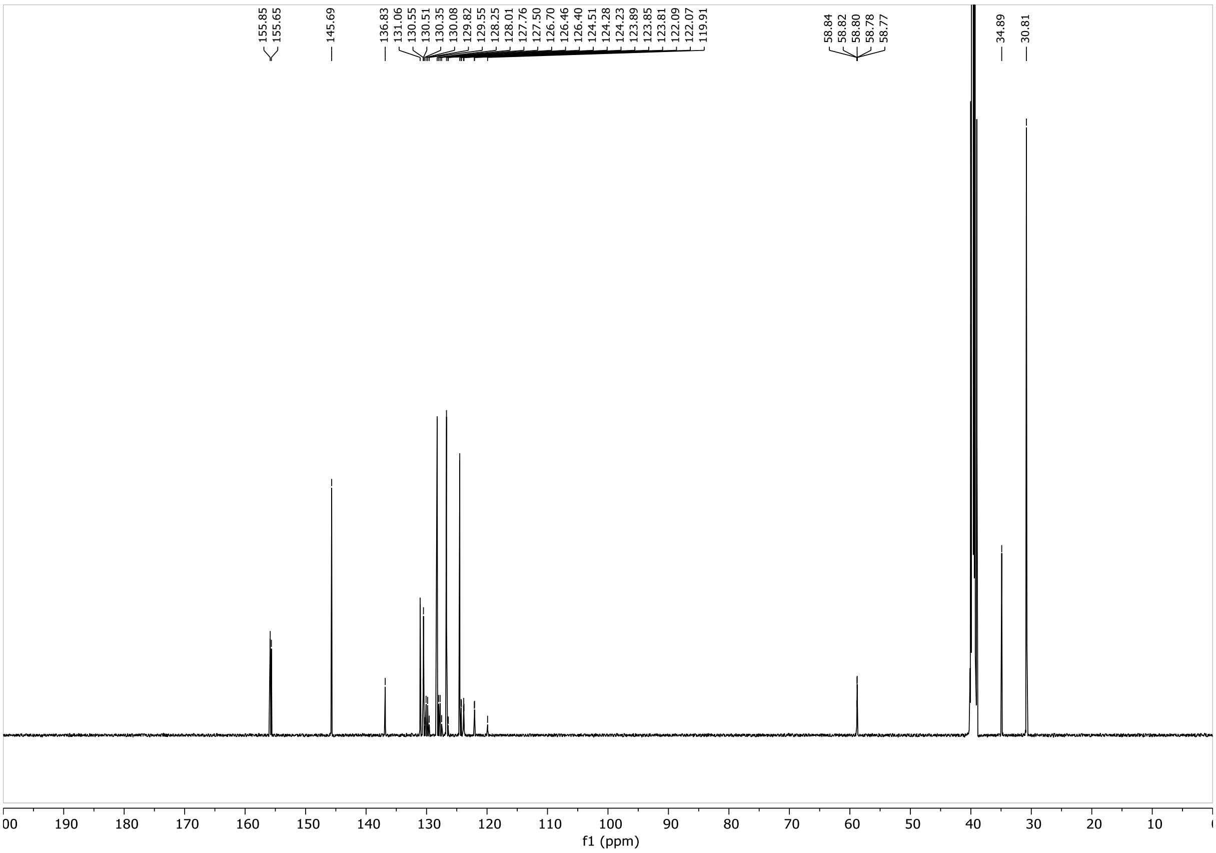

155.85
155.65
145.69
136.83
131.06
130.55
130.51
130.35
130.08
129.82
129.55
128.25
128.01
127.76
127.50
126.70
126.46
126.40
124.51
124.28
124.23
123.89
123.85
123.81
122.09
122.07
119.91
58.84
58.82
58.80
58.78
58.77
34.89
30.81
f1 (ppm)

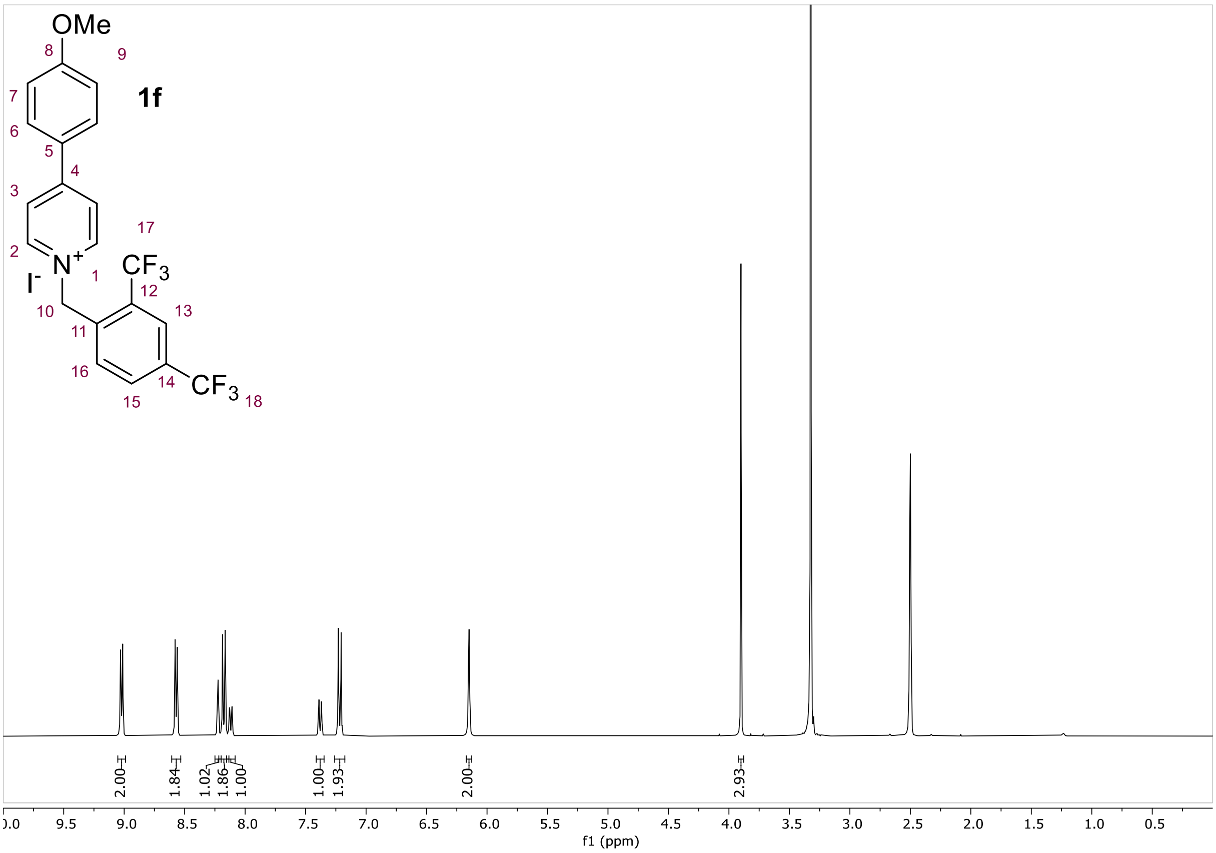
OMe
1f
CF3
CF3
I-
N+
2.00
1.84
1.02
1.86
1.00
1.00
1.93
2.00
2.93
f1 (ppm)

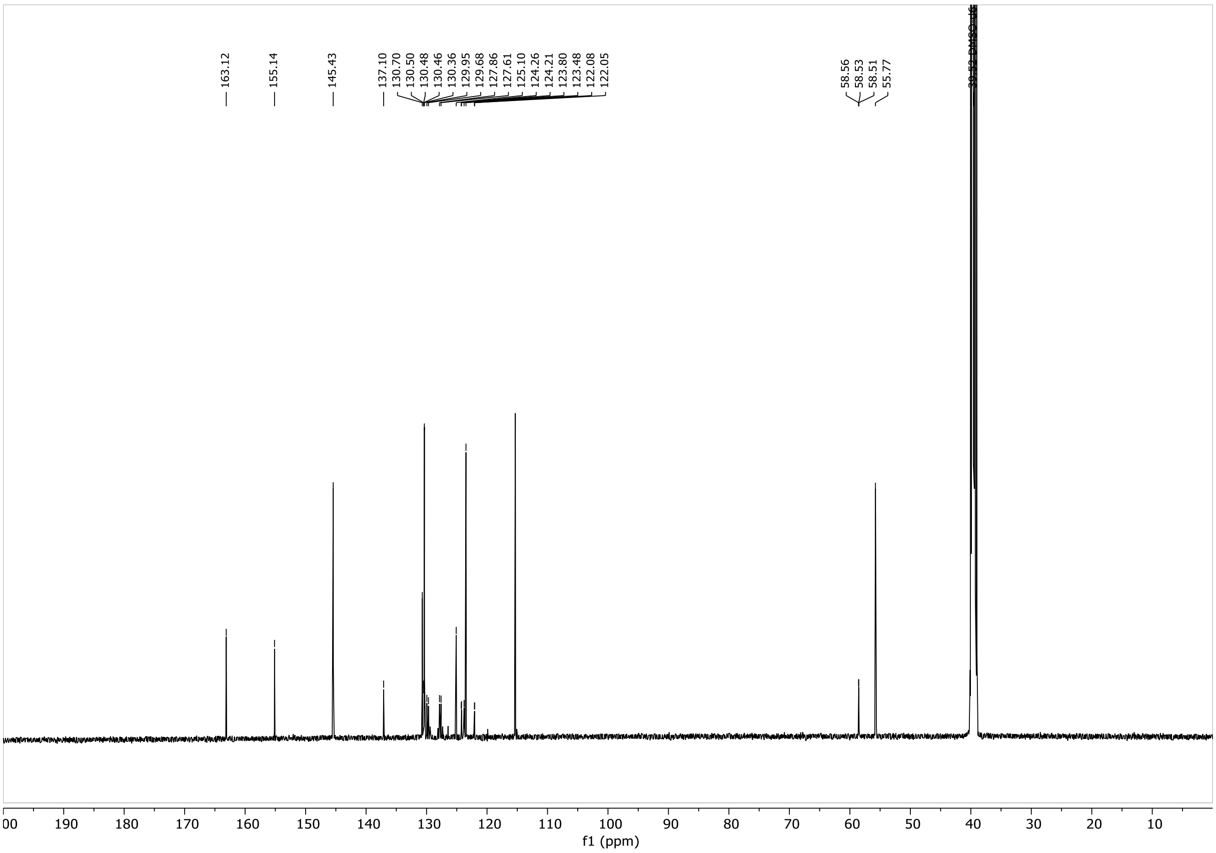
163.12
155.14
145.43
137.10
130.70
130.50
130.48
130.46
130.36
129.95
129.68
127.86
127.61
125.10
124.26
124.21
123.80
123.48
122.08
122.05
58.56
58.53
58.51
55.77
f1 (ppm)

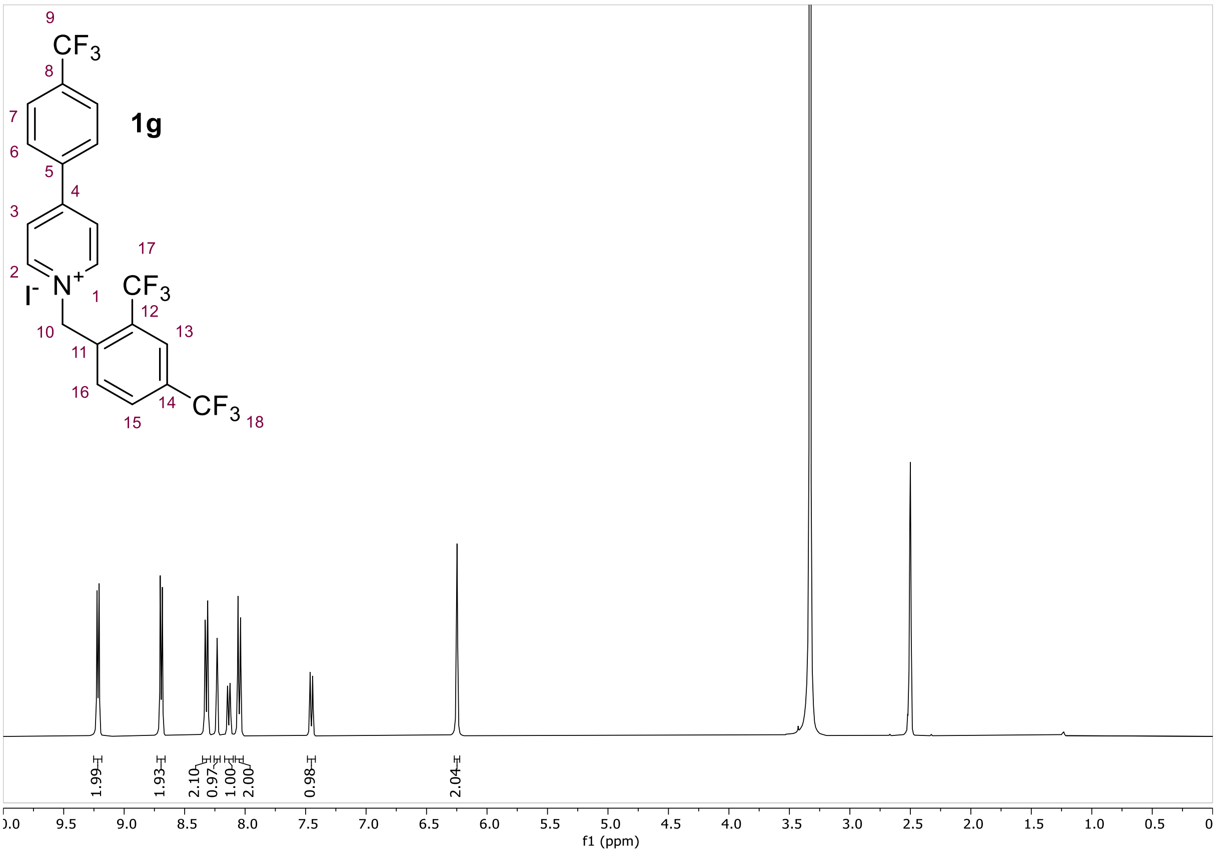
1g
9
CF3
8
7
6
5
4
3
17
2
N+
I-
1
CF3
12
13
10
11
16
14
CF3
15
18
1.99
1.93
2.10
0.97
1.00
2.00
0.98
2.04
f1 (ppm)

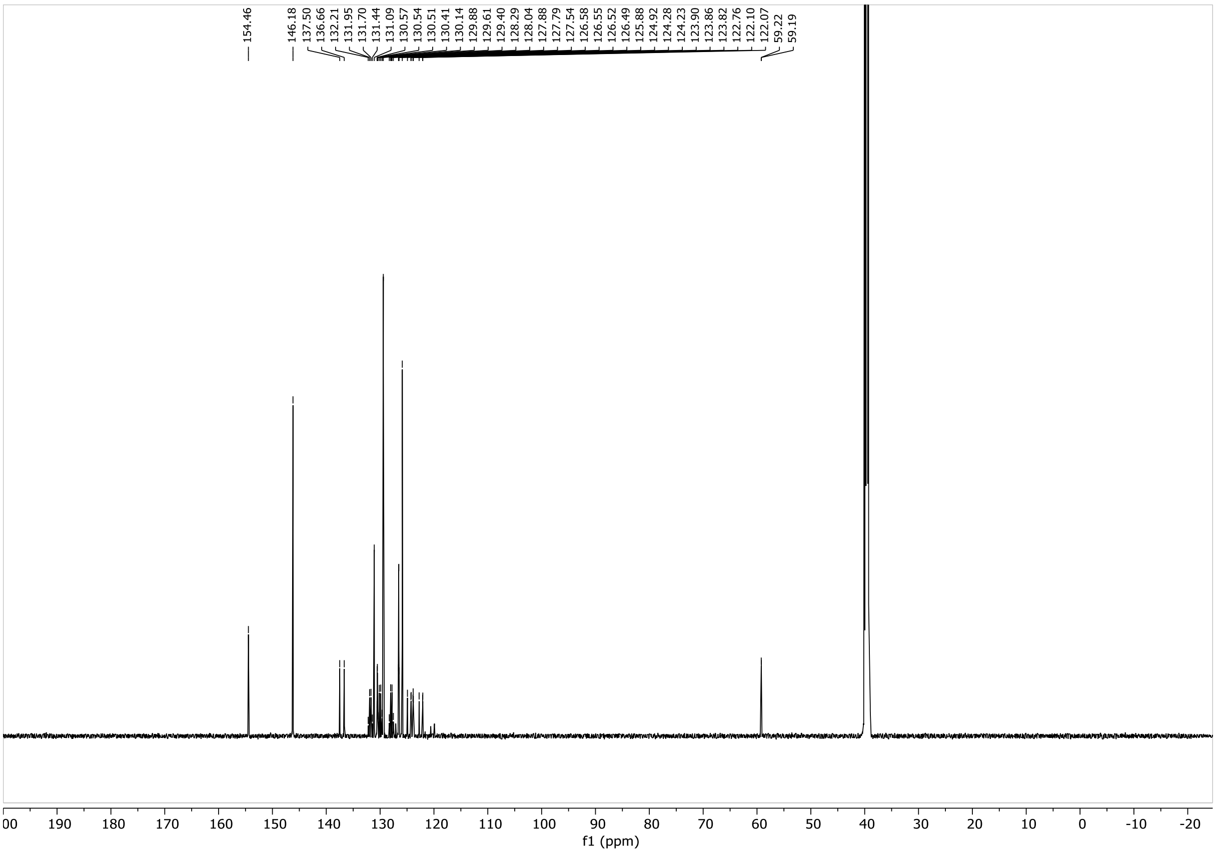
154.46
146.18
137.50
136.66
132.21
131.95
131.70
131.44
131.09
130.57
130.54
130.51
130.41
130.14
129.88
129.61
129.40
128.29
128.04
127.88
127.79
127.54
126.58
126.55
126.52
126.49
125.88
124.92
124.28
124.23
123.90
123.86
123.82
122.76
122.10
122.07
59.22
59.19
f1 (ppm)

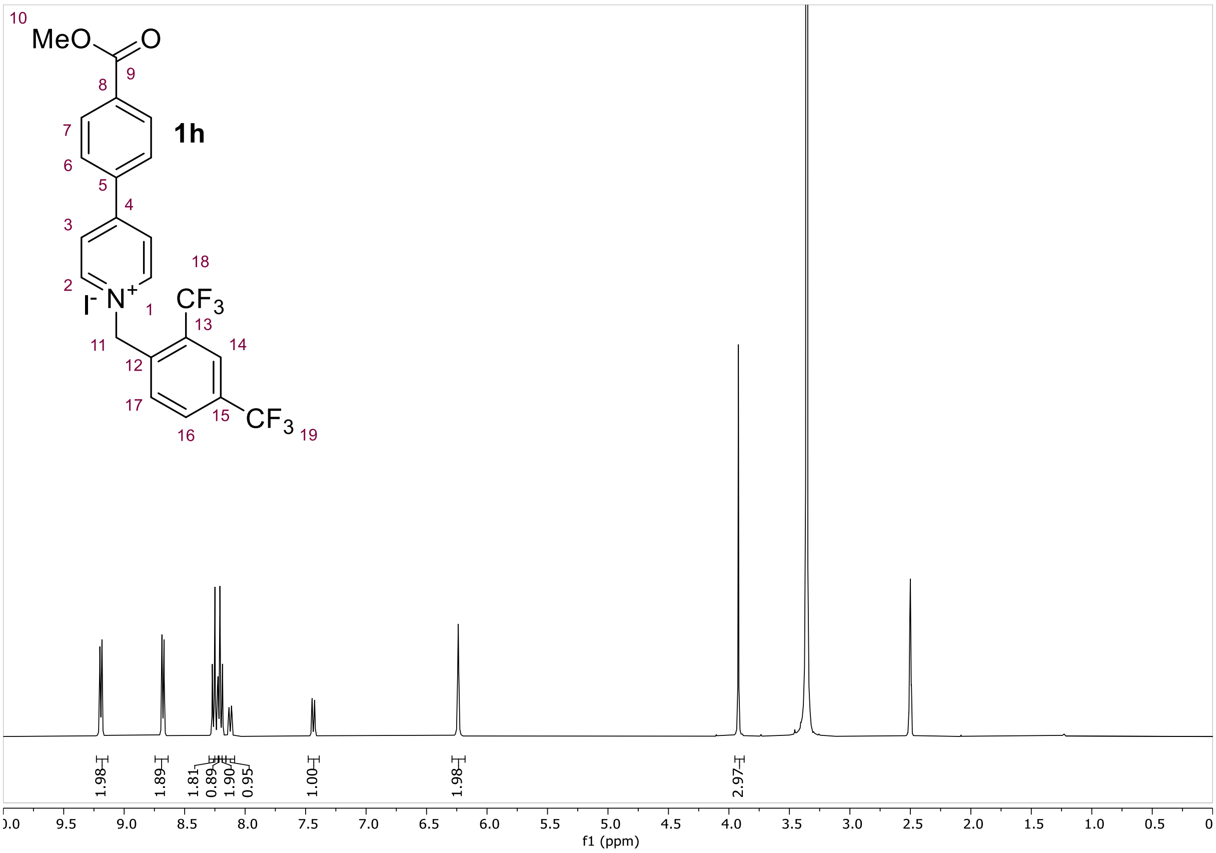
MeO
O
1h
I⁻
N⁺
CF3
CF3
1.98
1.89
1.81
0.89
1.90
0.95
1.00
1.98
2.97
f1 (ppm)

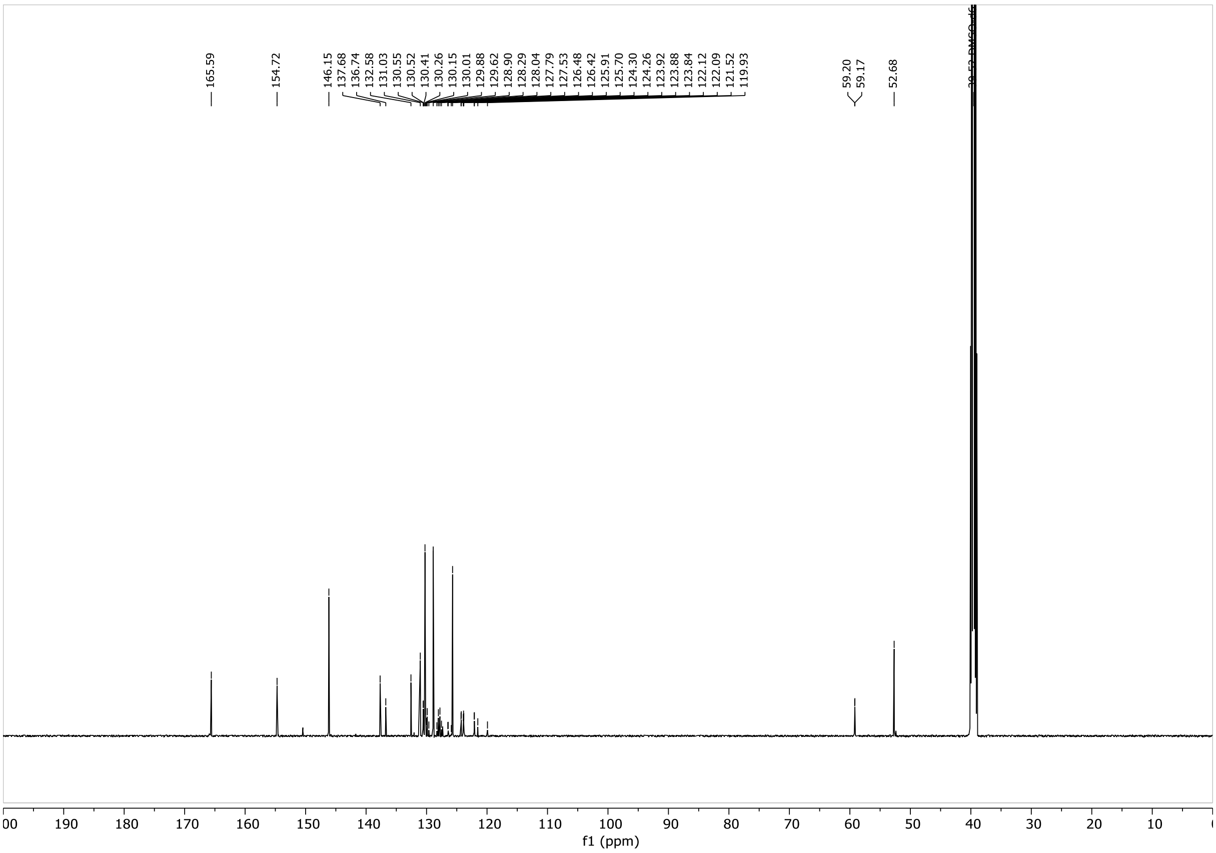
165.59
154.72
146.15
137.68
136.74
132.58
131.03
130.55
130.52
130.41
130.26
130.15
130.01
129.88
129.62
128.90
128.29
128.04
127.79
127.53
126.48
126.42
125.91
125.70
124.30
124.26
123.92
123.88
123.84
122.12
122.09
121.52
119.93
59.20
59.17
52.68
f1 (ppm)

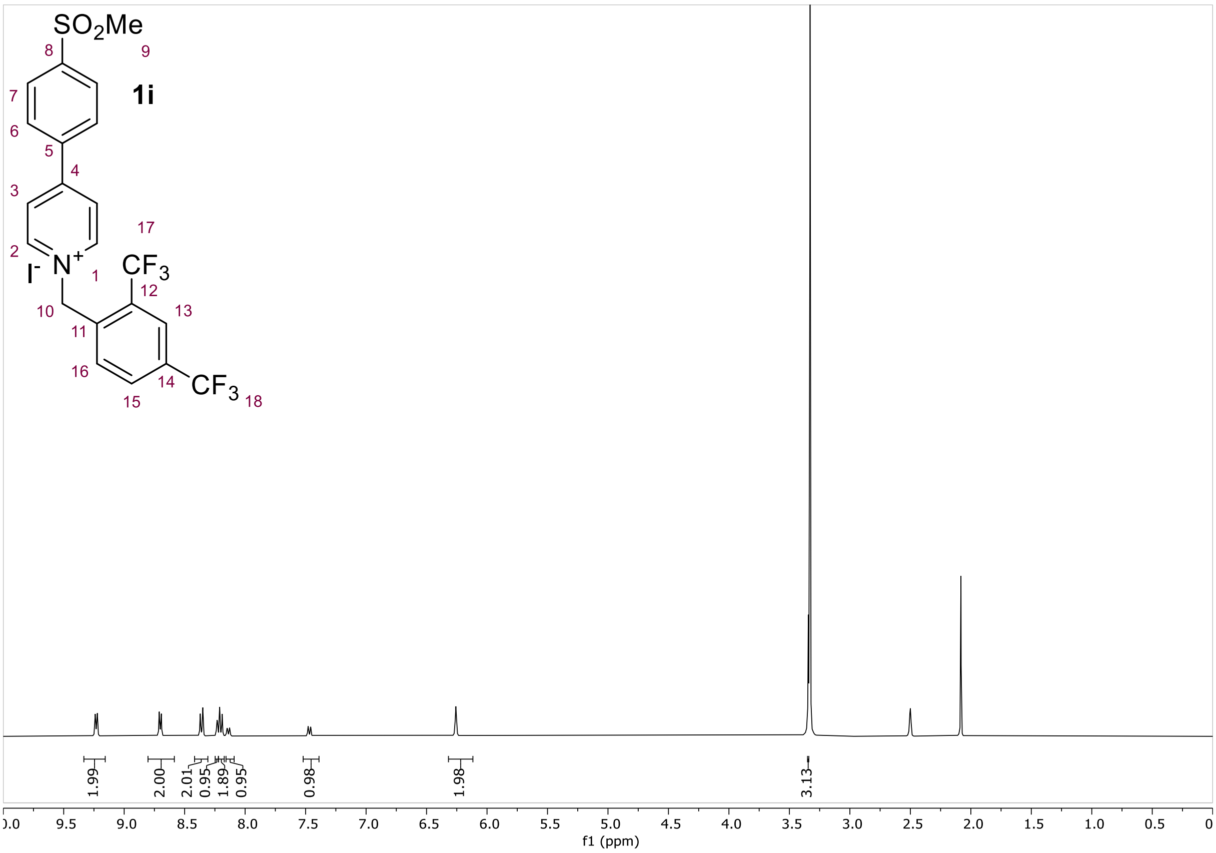

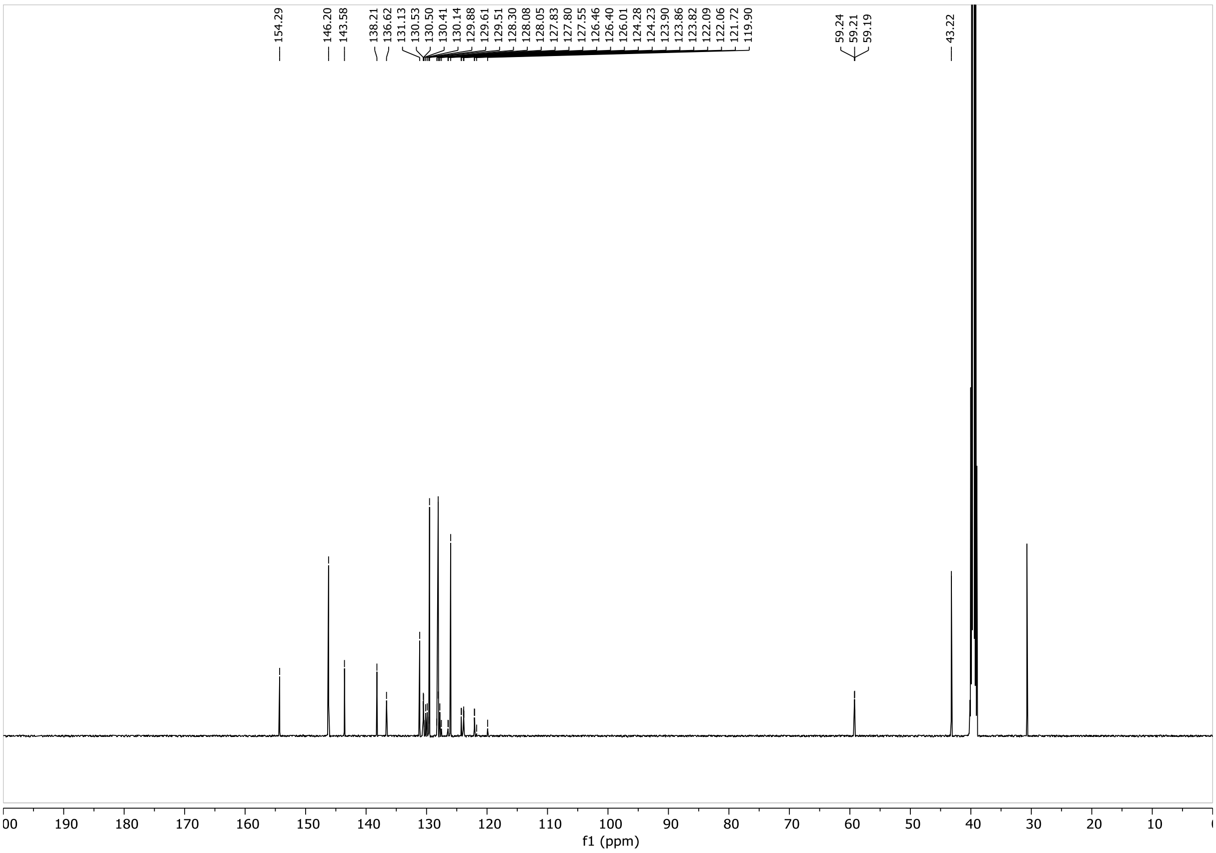

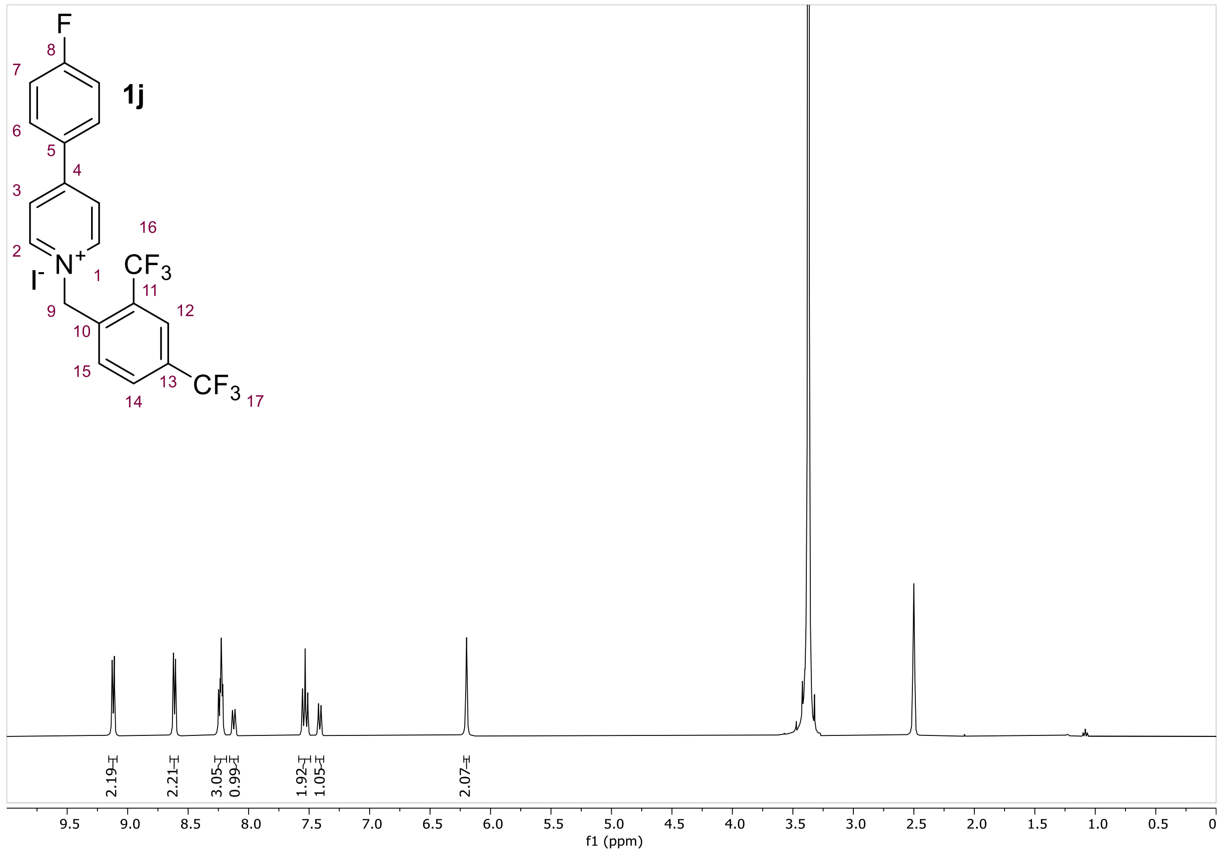

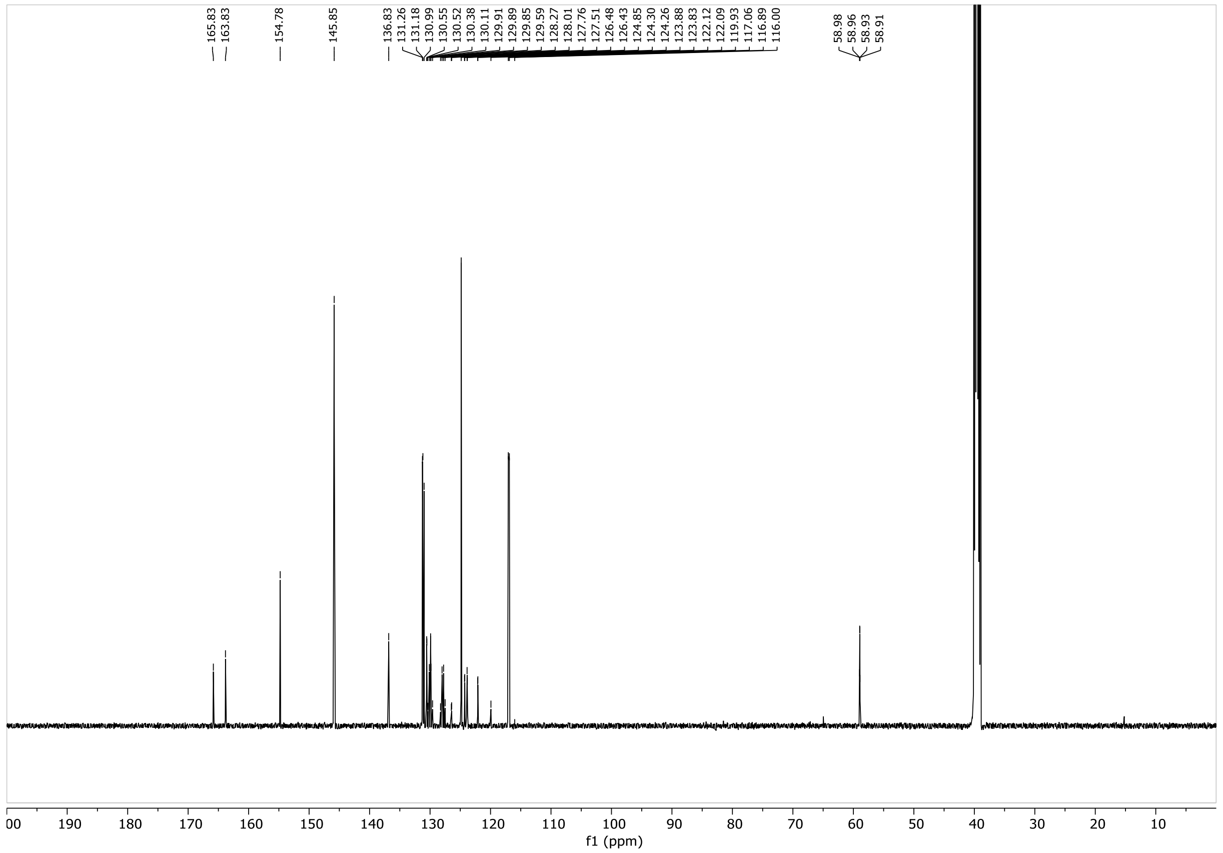

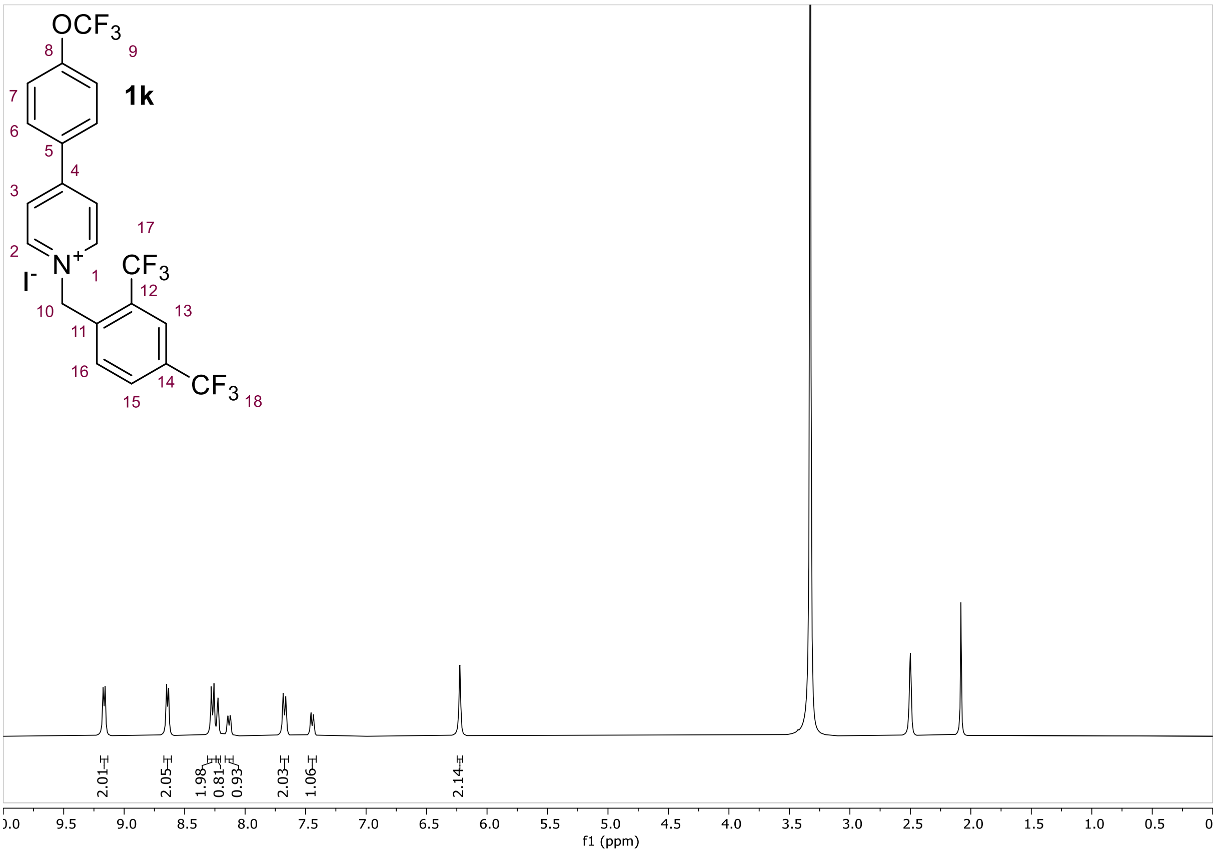
OCF3
1k
I-
N+
CF3
CF3
2.01
2.05
1.98
0.81
0.93
2.03
1.06
2.14
f1 (ppm)

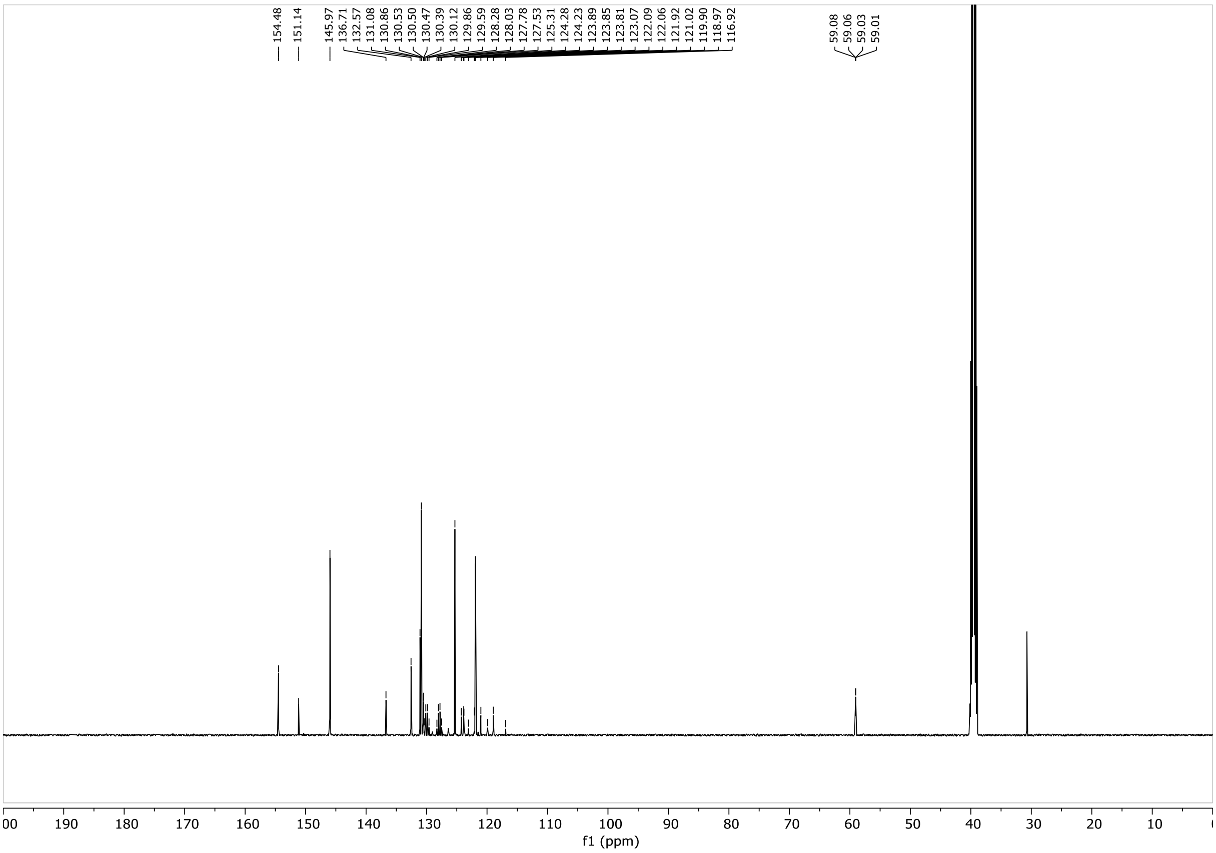
154.48
151.14
145.97
136.71
132.57
131.08
130.86
130.53
130.50
130.47
130.39
130.12
129.86
129.59
128.28
128.03
127.78
127.53
125.31
124.28
124.23
123.89
123.85
123.81
123.07
122.09
122.06
121.92
121.02
119.90
118.97
116.92
59.08
59.06
59.03
59.01
f1 (ppm)

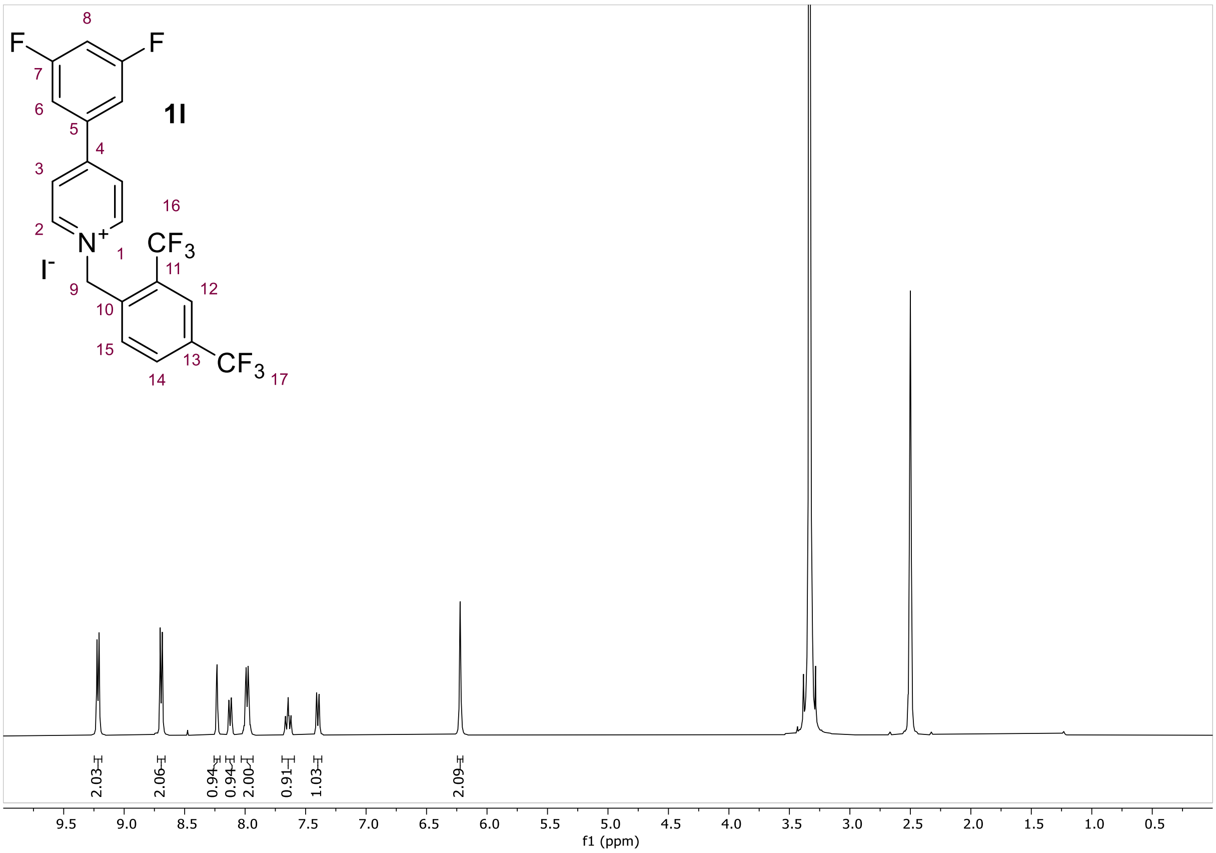

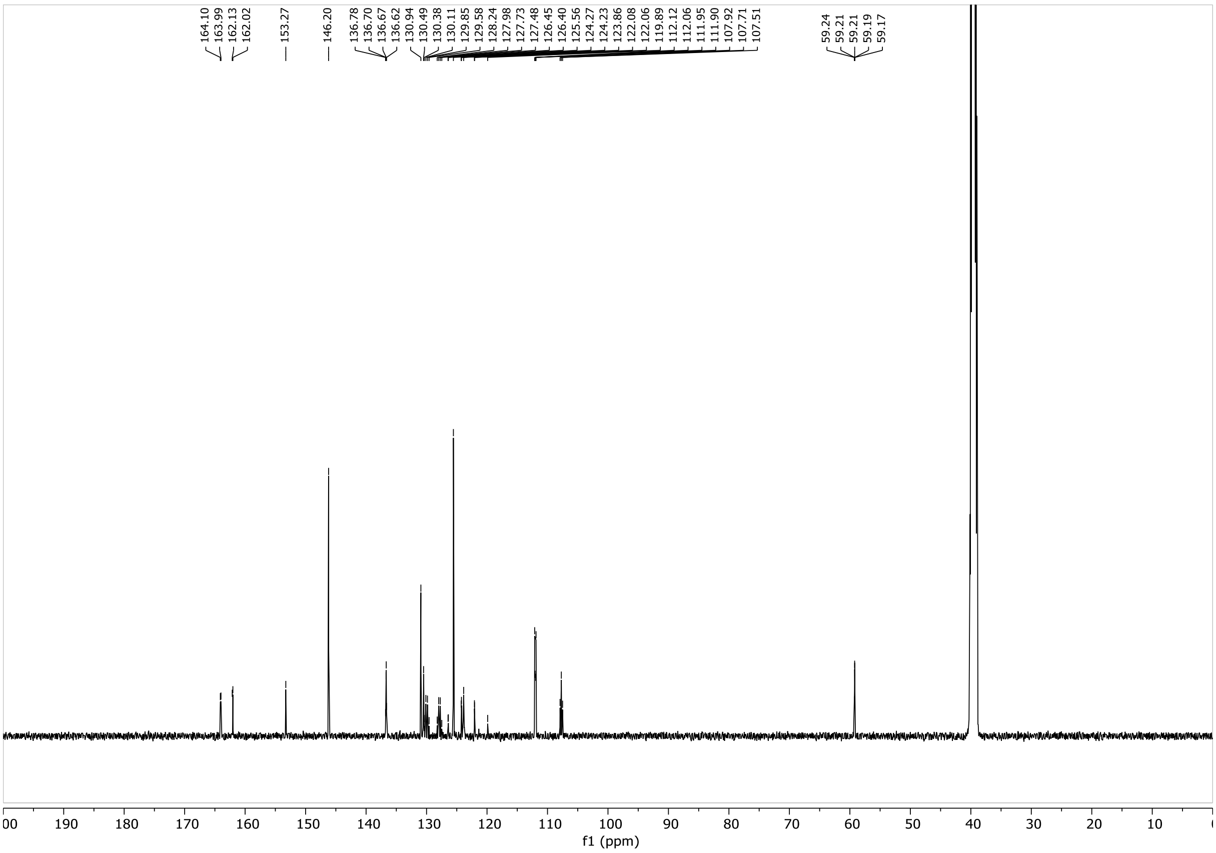

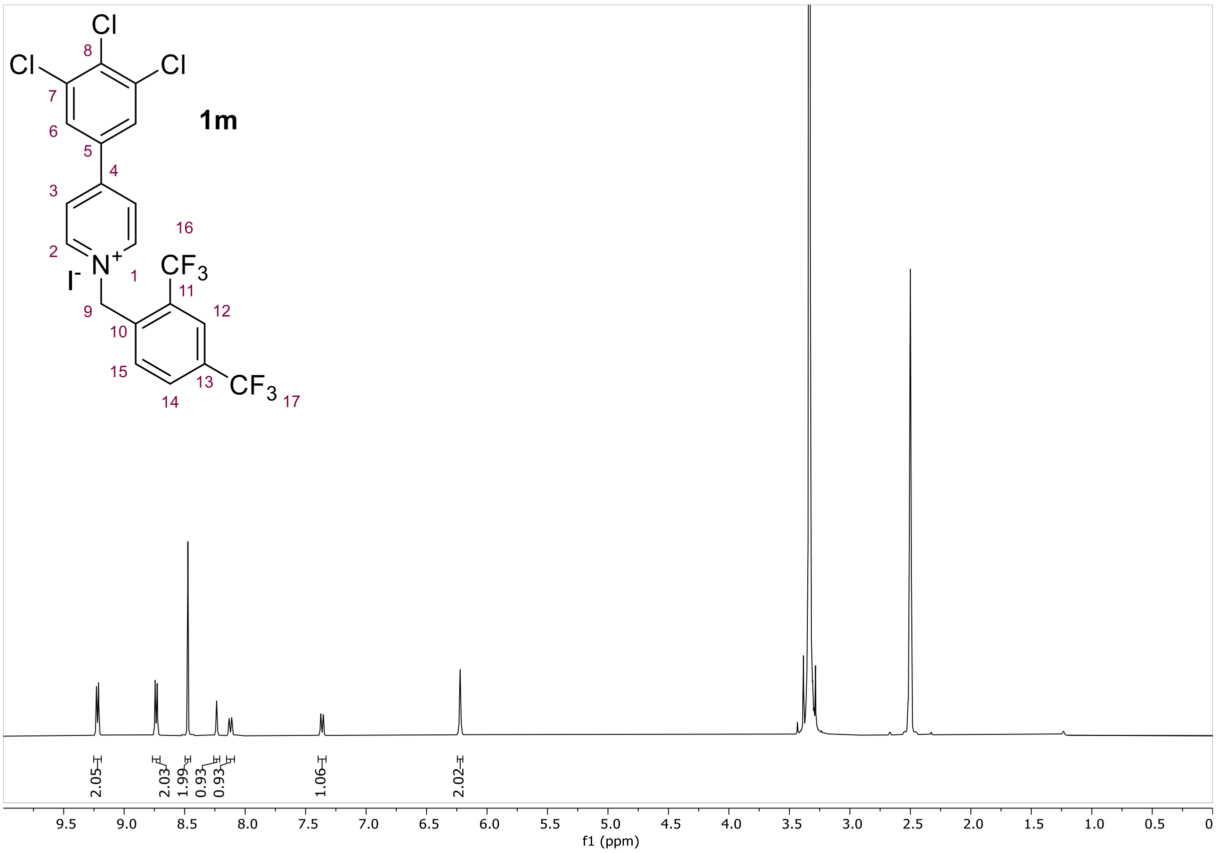

Cl
Cl
Cl
1m
I-
N+
CF3
CF3
2.05
2.03
1.99
0.93
0.93
1.06
2.02
9.5 9.0 8.5 8.0 7.5 7.0 6.5 6.0 5.5 5.0 4.5 4.0 3.5 3.0 2.5 2.0 1.5 1.0 0.5 0
f1 (ppm)

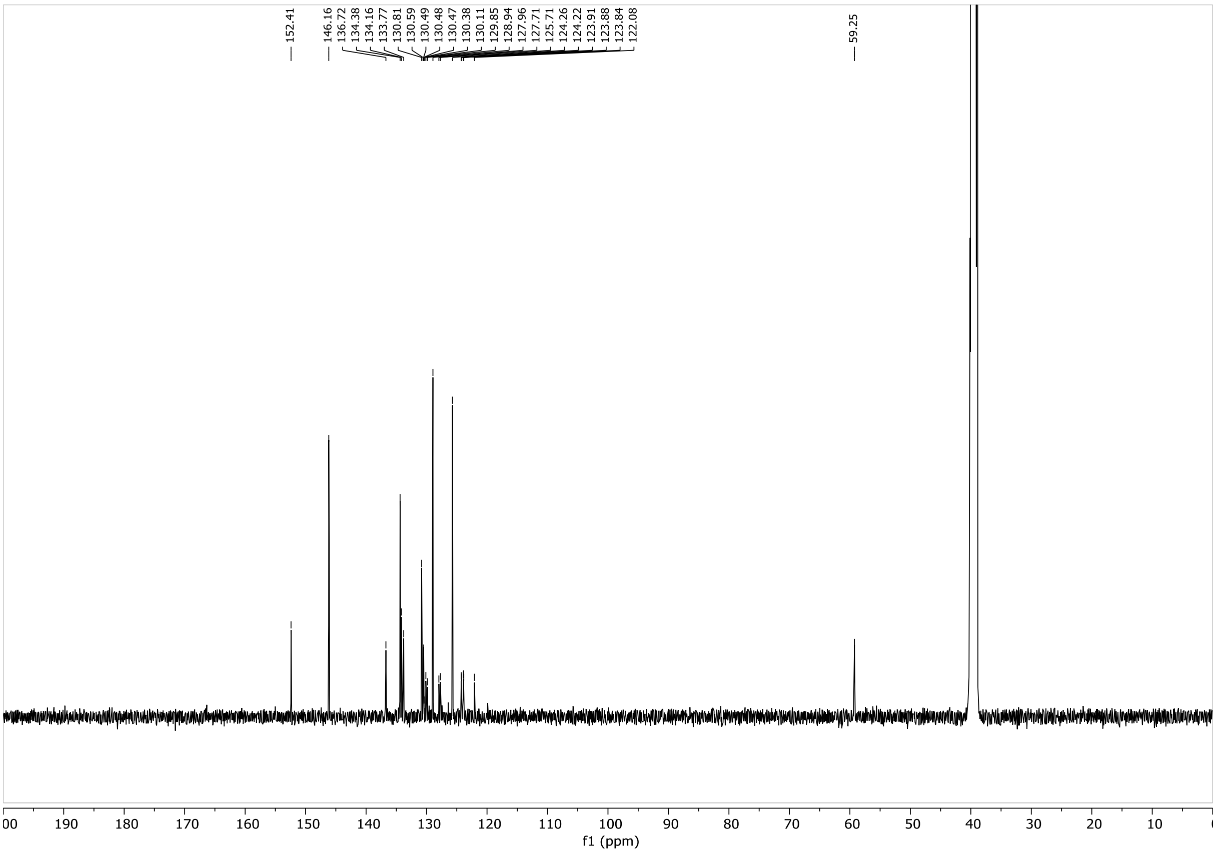

152.41
146.16
136.72
134.38
134.16
133.77
130.81
130.59
130.49
130.48
130.47
130.38
130.11
129.85
128.94
127.96
127.71
125.71
124.26
124.22
123.91
123.88
123.84
122.08
59.25
00 190 180 170 160 150 140 130 120 110 100 90 80 70 60 50 40 30 20 10
f1 (ppm)

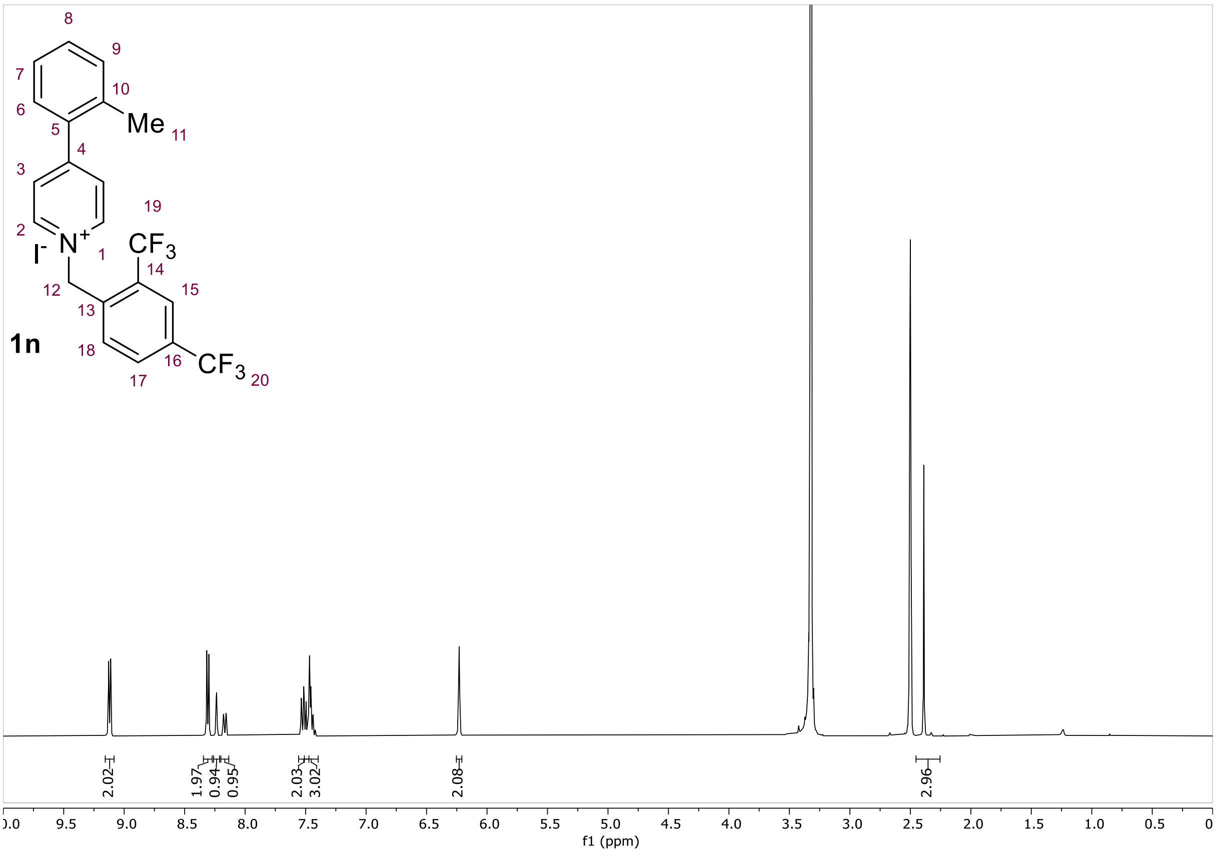
1n
Me
CF3
CF3
I-
N+
2.02
1.97
0.94
0.95
2.03
3.02
2.08
2.96
f1 (ppm)

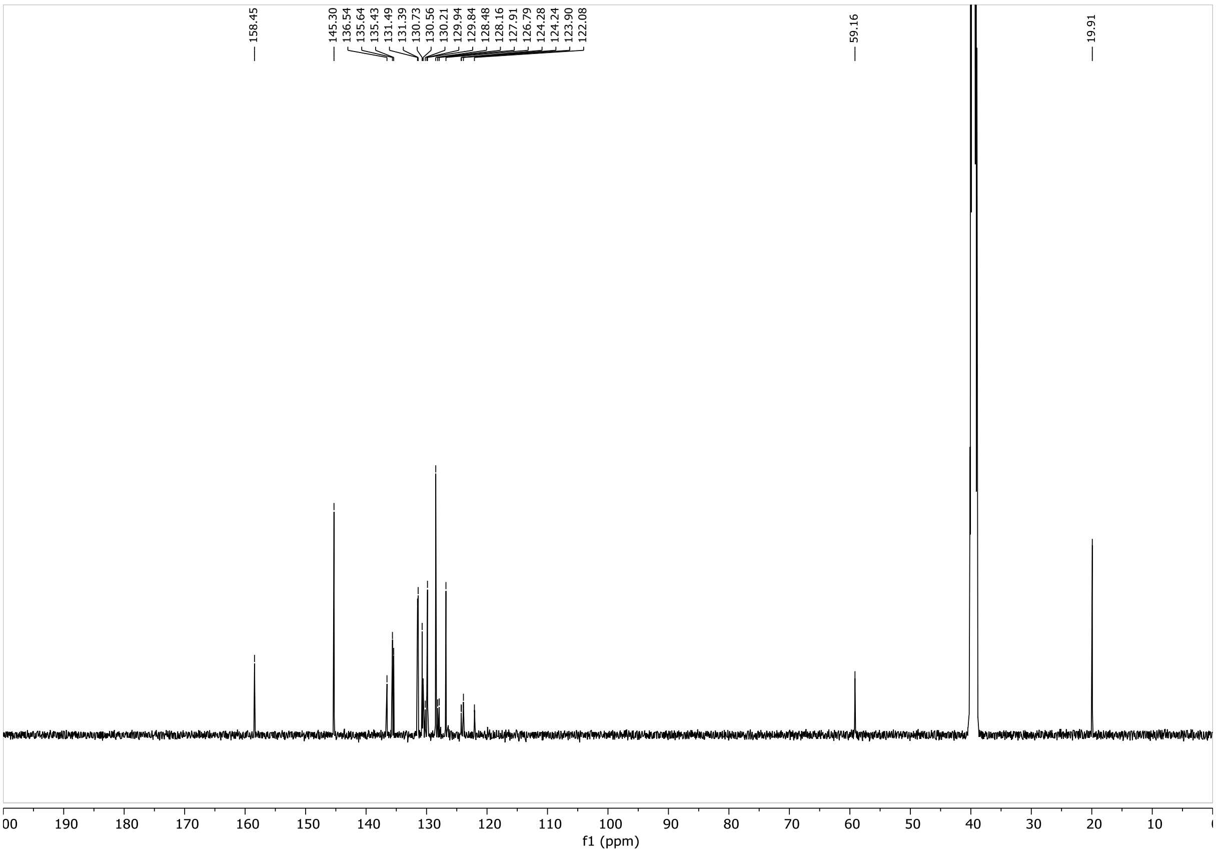
158.45
145.30
136.54
135.64
135.43
131.49
131.39
130.73
130.56
130.21
129.94
129.84
128.48
128.16
127.91
126.79
124.28
124.24
123.90
122.08
59.16
19.91
f1 (ppm)

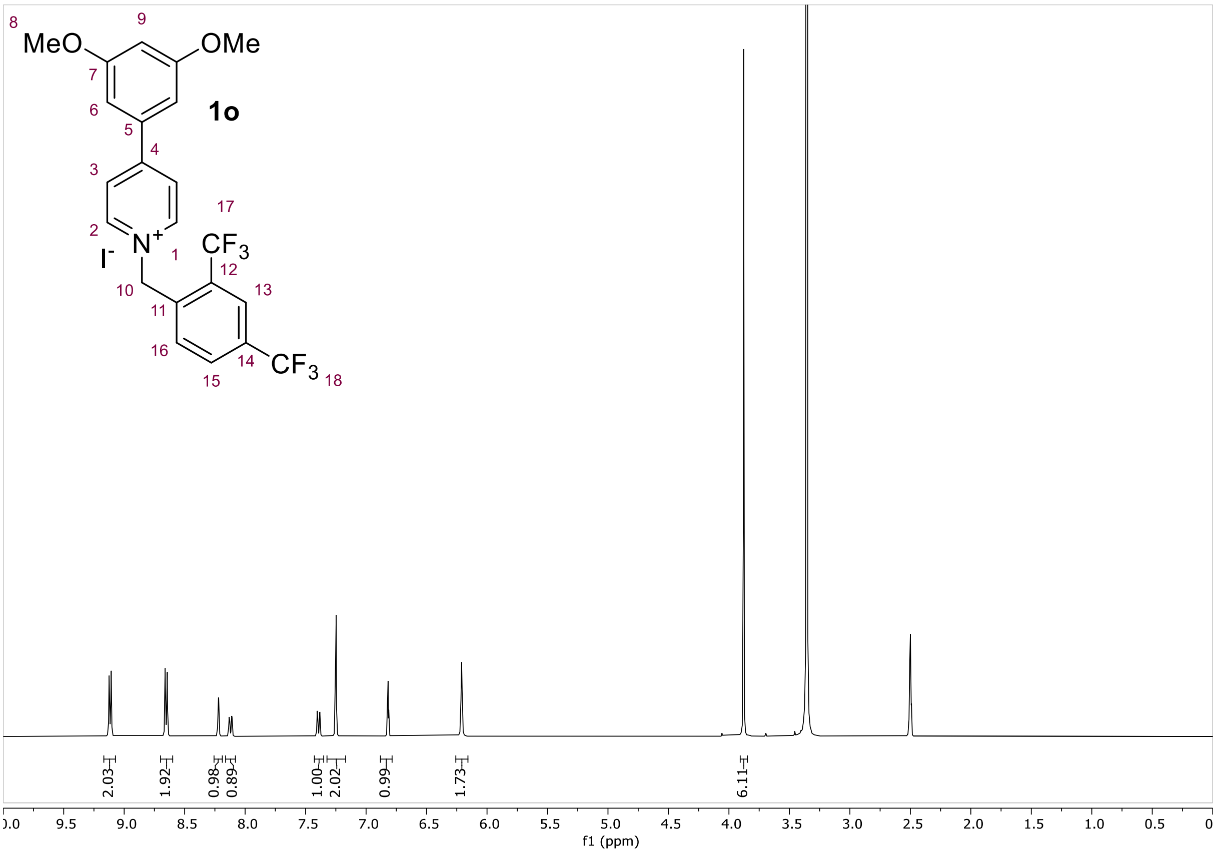

1o
MeO
OMe
CF3
CF3
I-
N+
2.03
1.92
0.98
0.89
1.00
2.02
0.99
1.73
6.11
f1 (ppm)

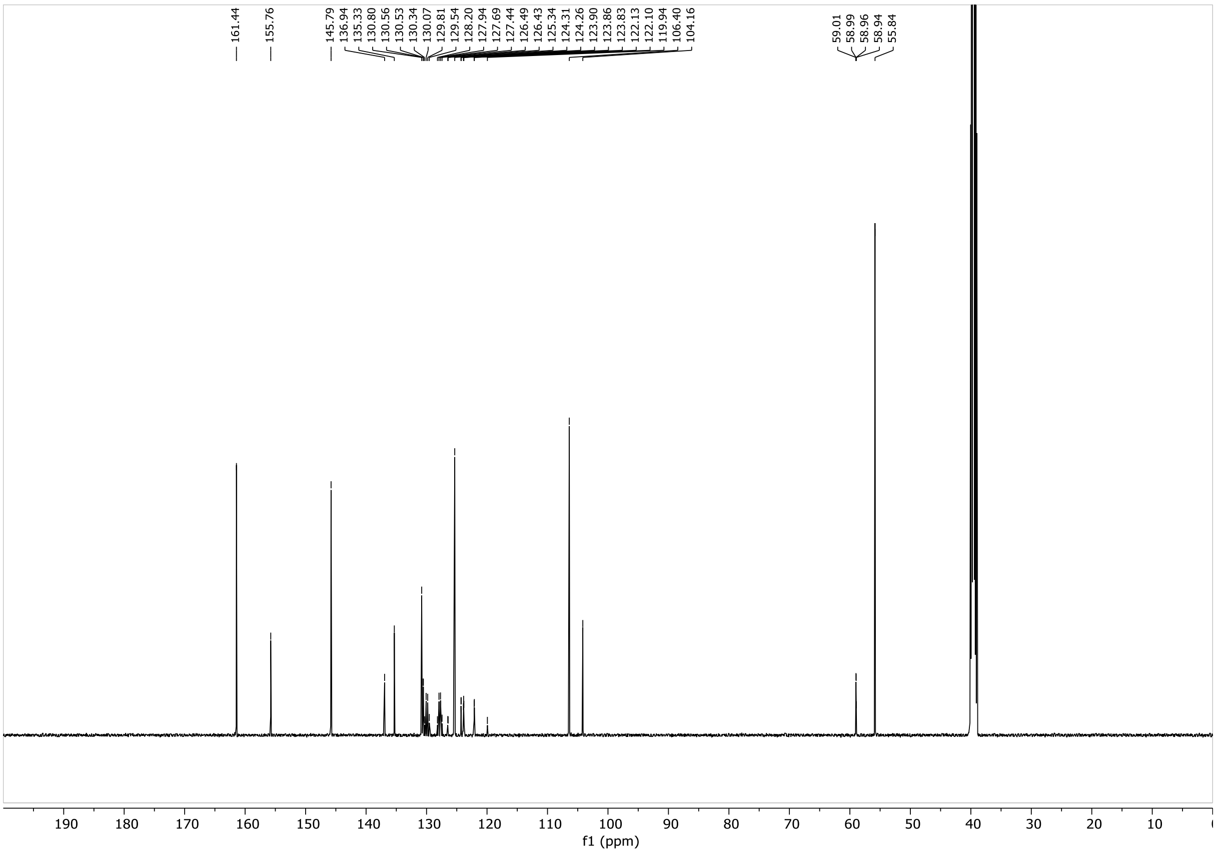

161.44
155.76
145.79
136.94
135.33
130.80
130.56
130.53
130.34
130.07
129.81
129.54
128.20
127.94
127.69
127.44
126.49
126.43
125.34
124.31
124.26
123.90
123.86
123.83
122.13
122.10
119.94
106.40
104.16
59.01
58.99
58.96
58.94
55.84
f1 (ppm)

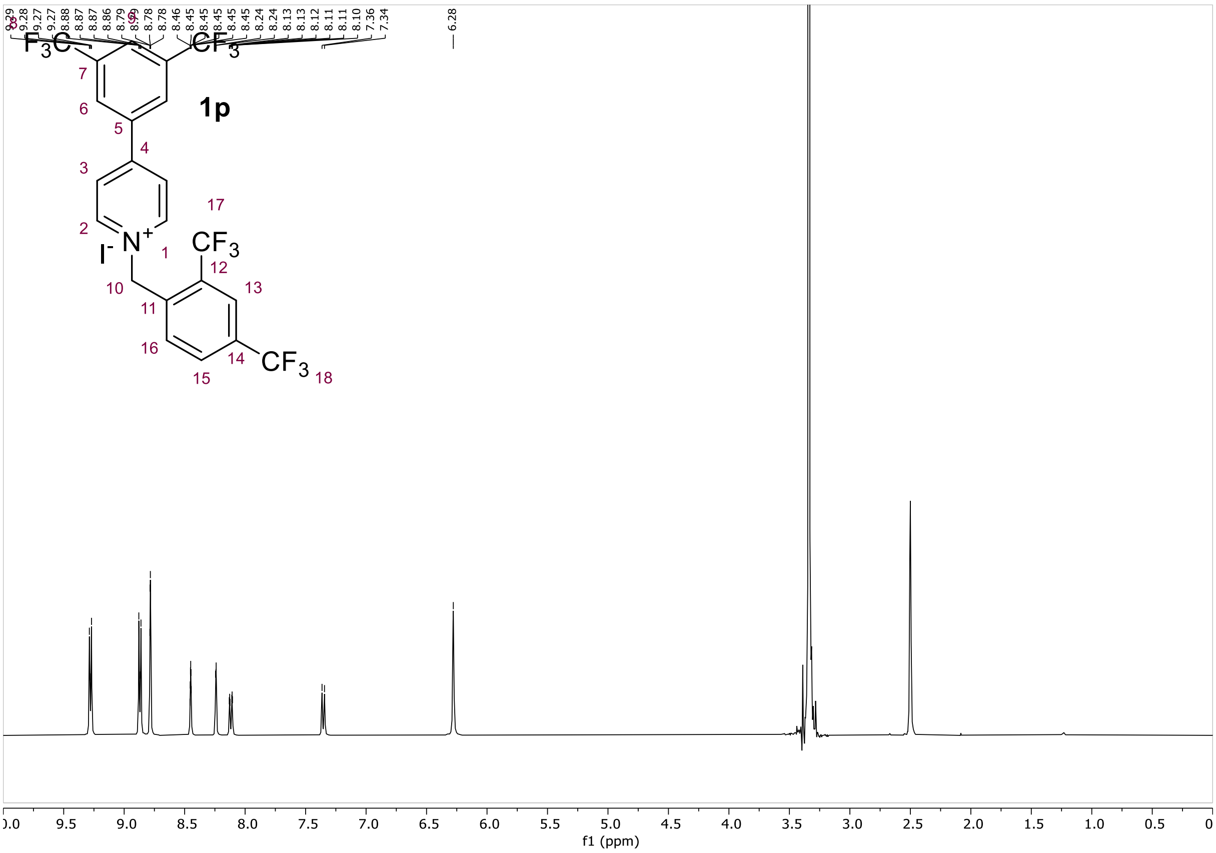

9.29
9.28
9.27
9.27
8.88
8.87
8.87
8.86
8.79
8.79
8.78
8.78
8.46
8.45
8.45
8.45
8.45
8.24
8.24
8.13
8.13
8.12
8.11
8.11
8.10
7.36
7.34
6.28
F3C
CF3
7
6
5
4
3
2
1
1p
17
CF3
12
13
I-
N+
10
11
16
15
14
CF3
18
f1 (ppm)

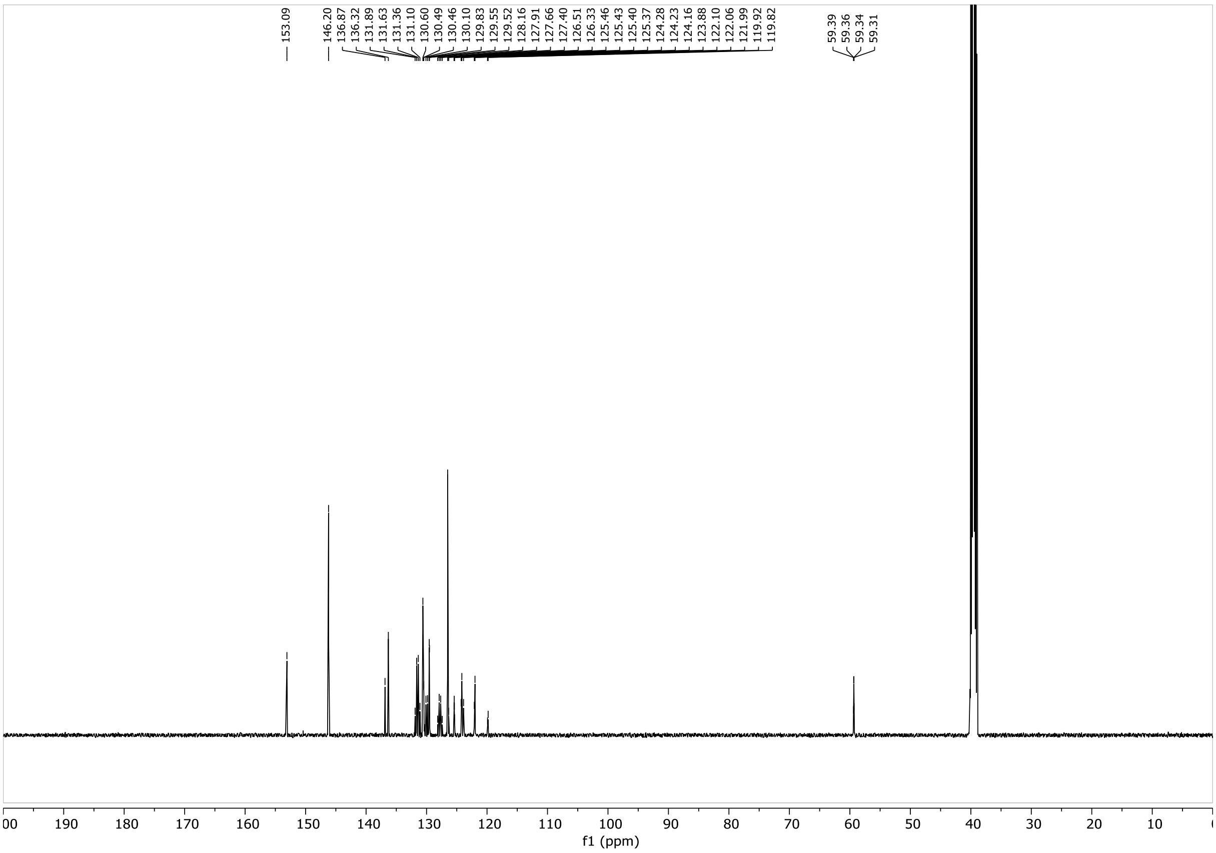

153.09
146.20
136.87
136.32
131.89
131.63
131.36
131.10
130.60
130.49
130.46
130.10
129.83
129.55
129.52
128.16
127.91
127.66
127.40
126.51
126.33
125.46
125.43
125.40
125.37
124.28
124.23
124.16
123.88
122.10
122.06
121.99
121.92
119.82
59.39
59.36
59.34
59.31
f1 (ppm)

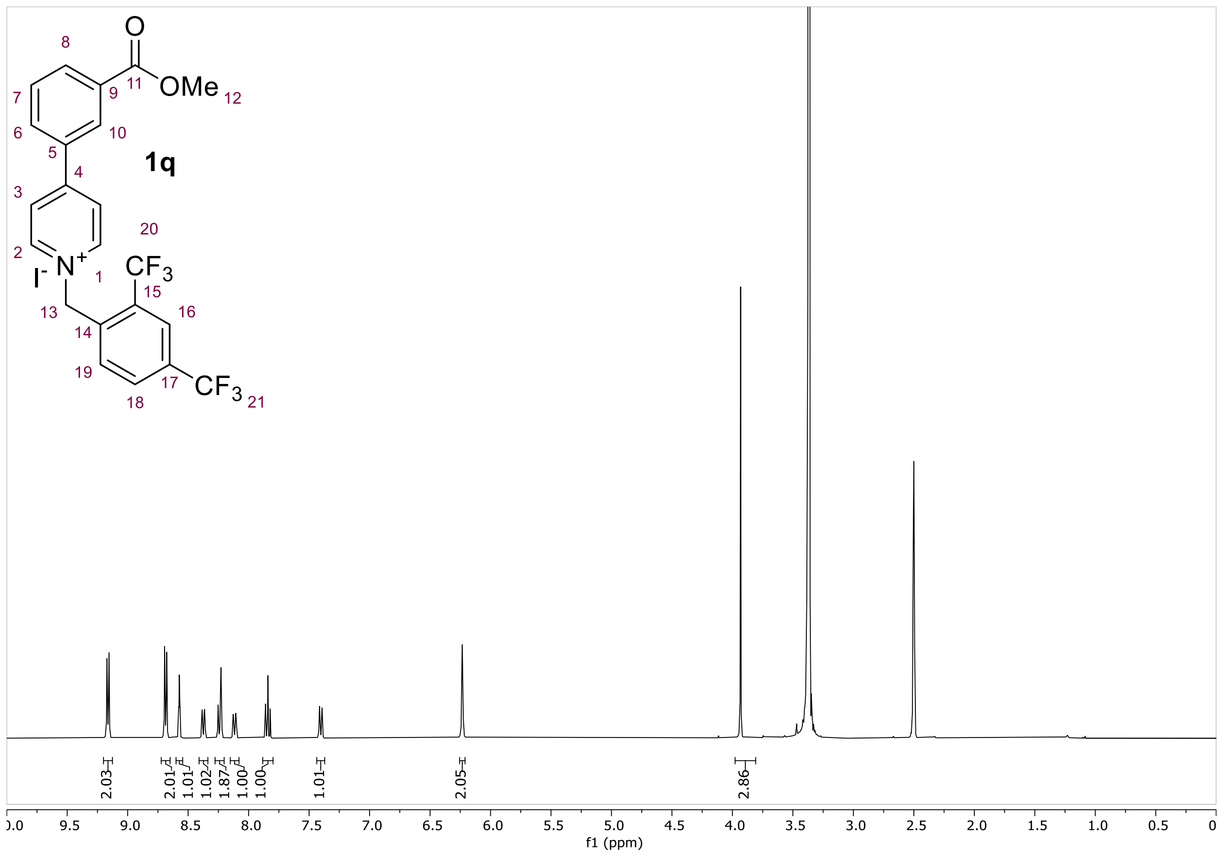

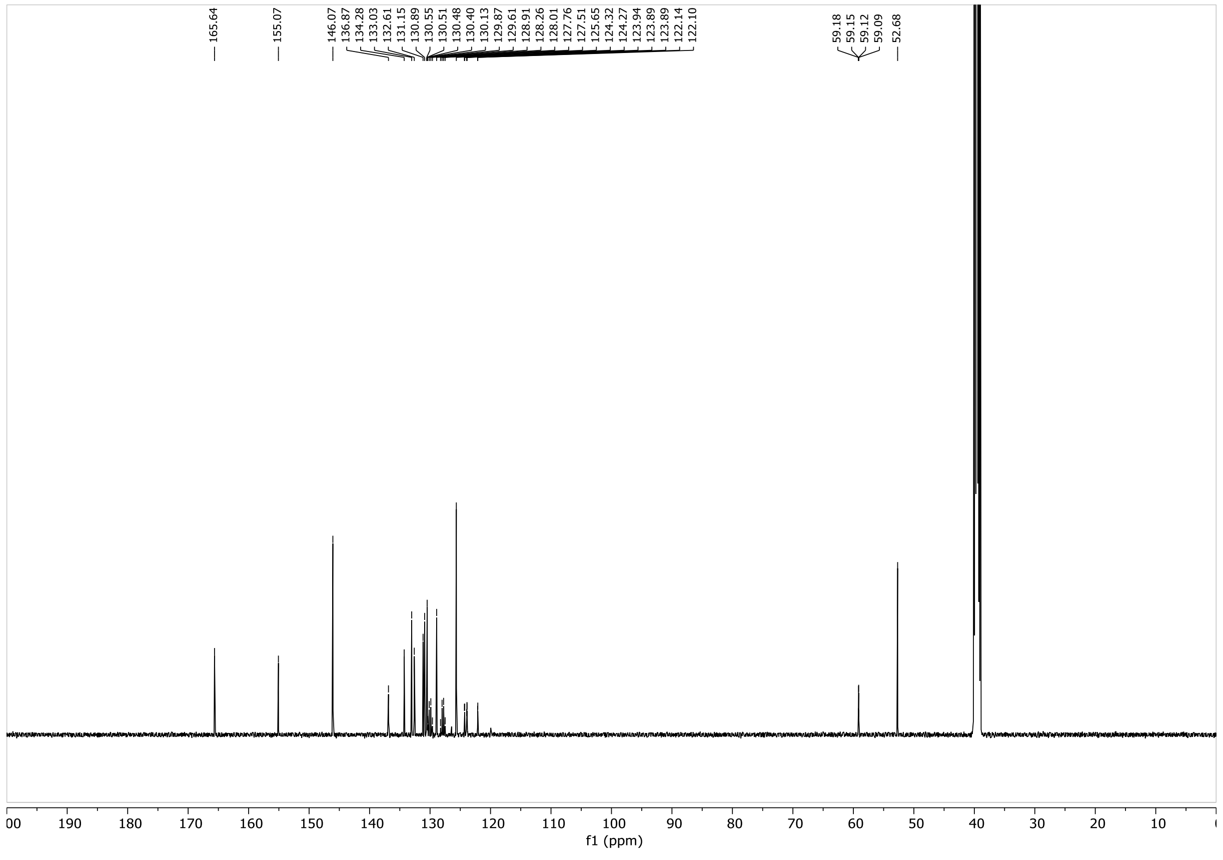

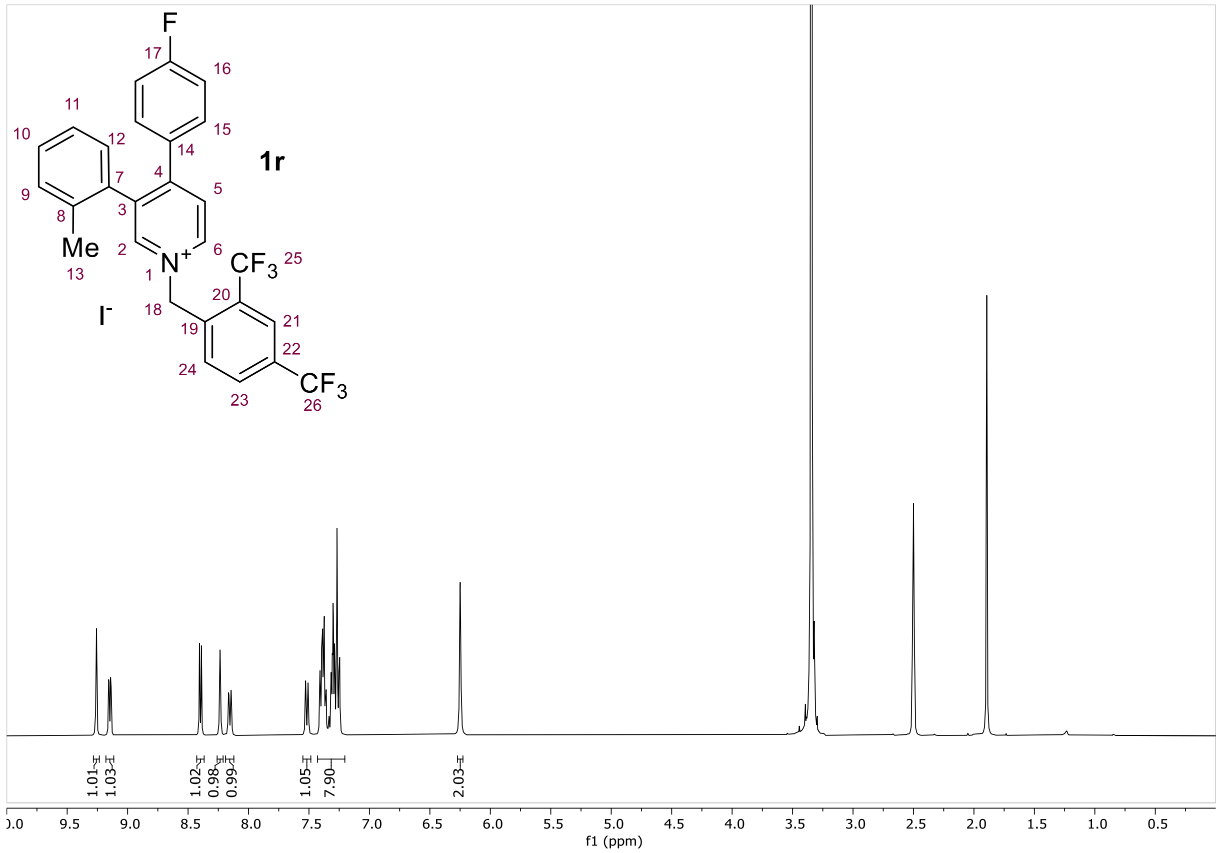

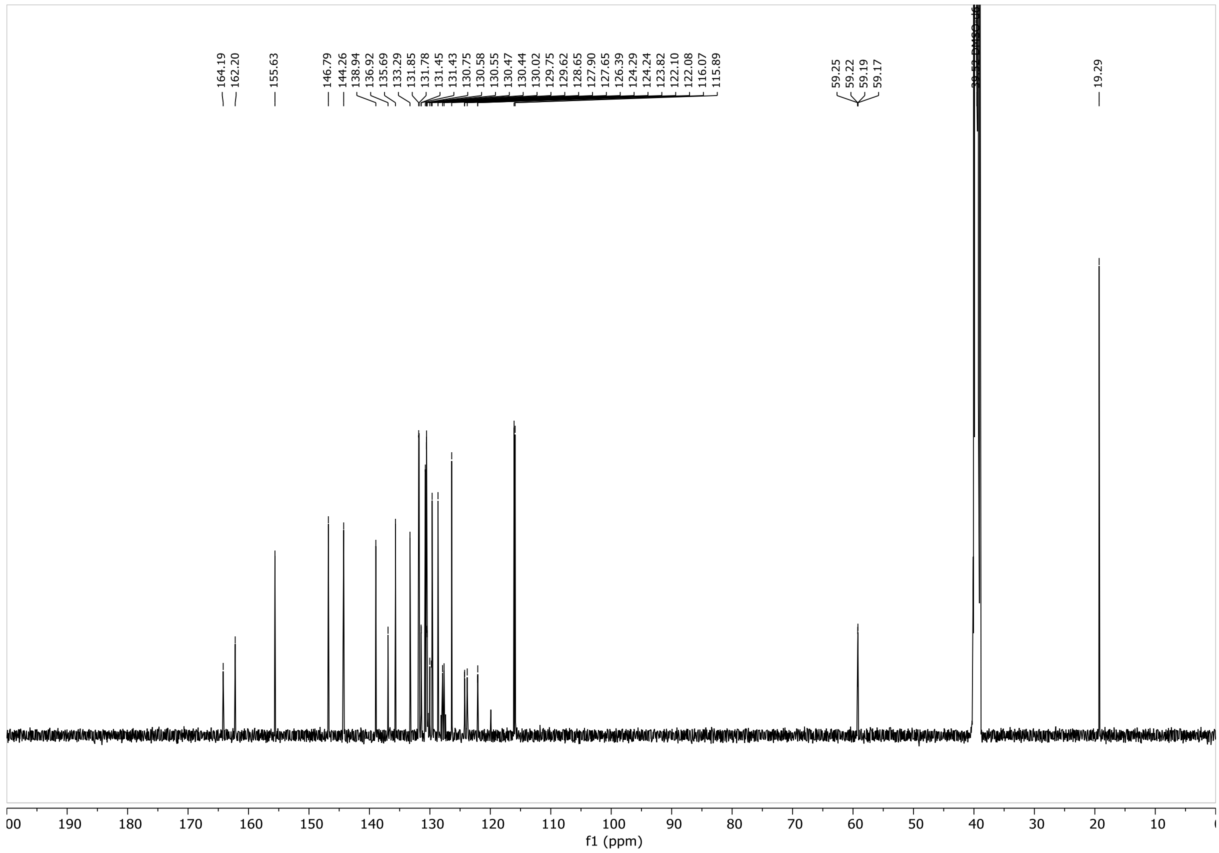

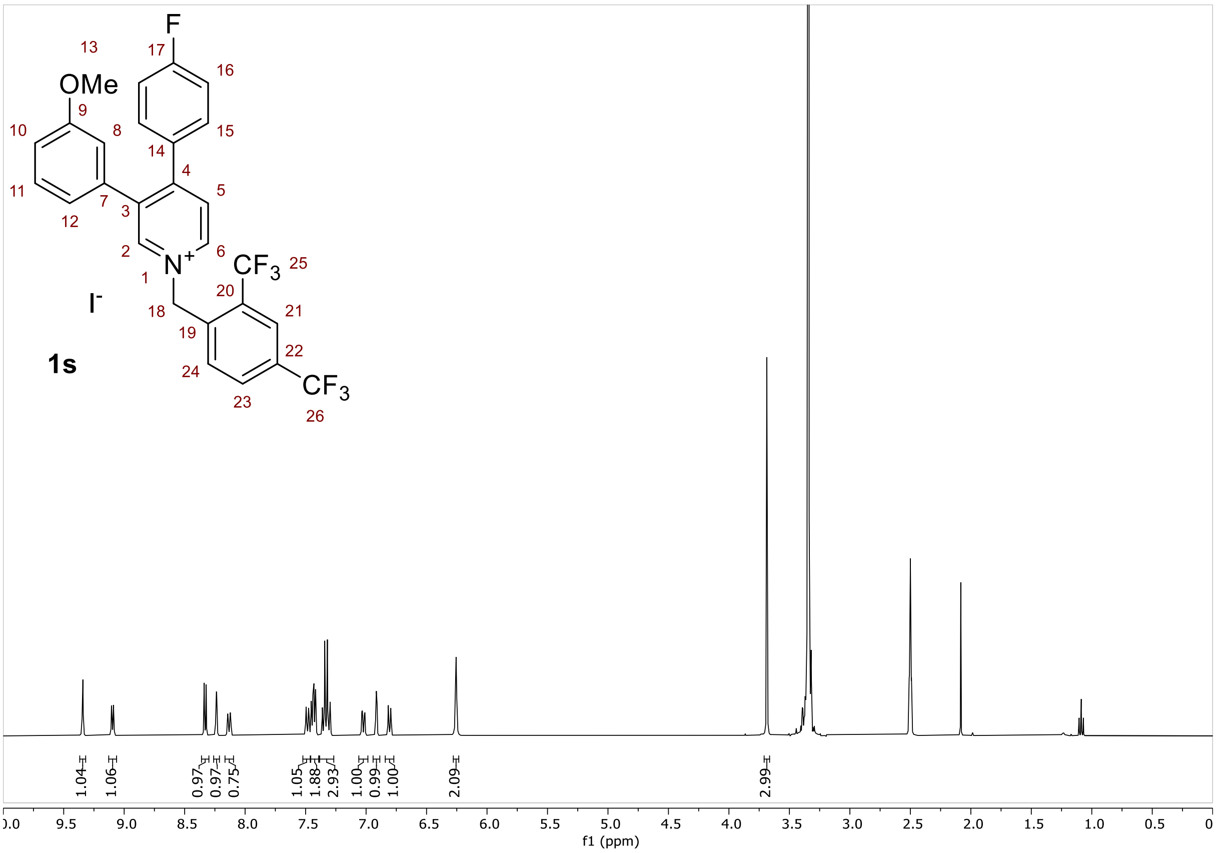

1s
1.04
1.06
0.97
0.97
0.75
1.05
1.88
2.93
1.00
0.99
1.00
2.09
2.99
f1 (ppm)

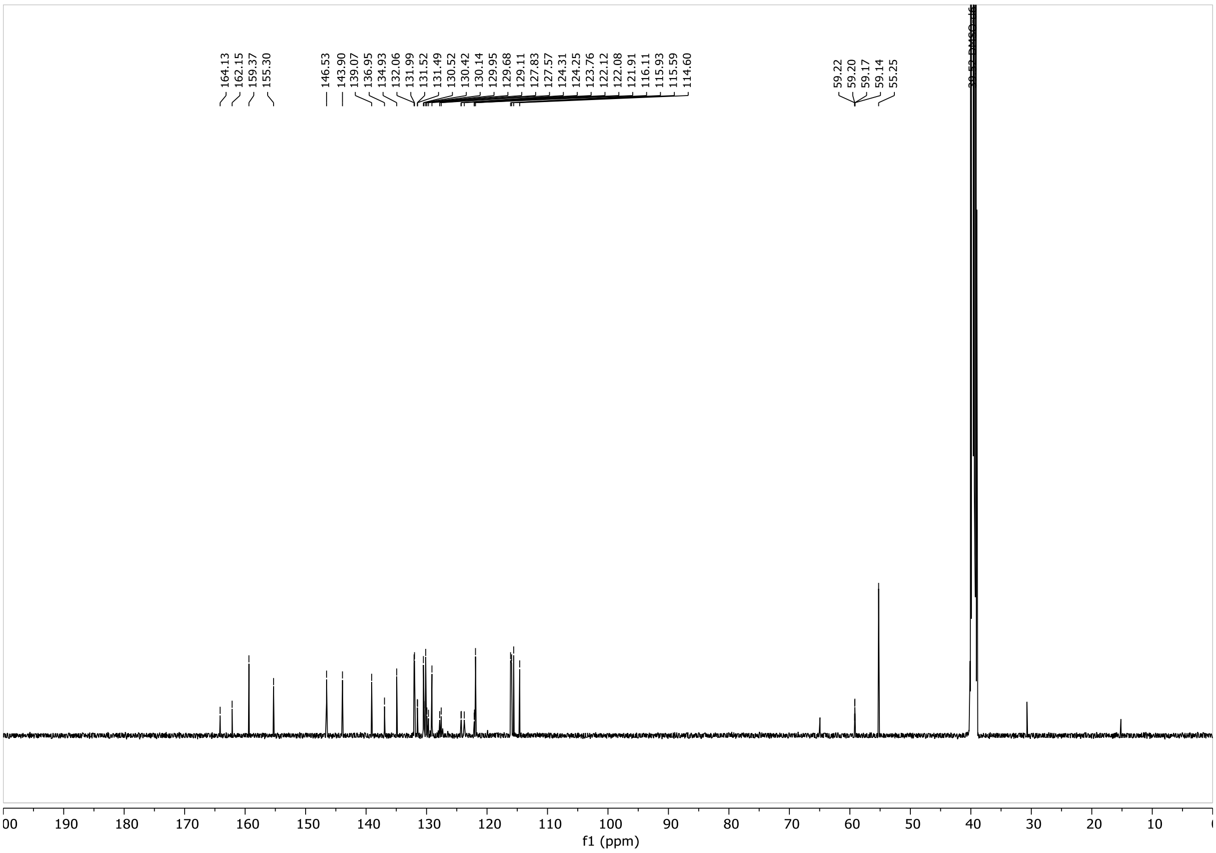

164.13
162.15
159.37
155.30
146.53
143.90
139.07
136.95
134.93
132.06
131.99
131.52
131.49
130.52
130.42
130.14
129.95
129.68
129.11
127.83
127.57
124.31
124.25
123.76
122.12
122.08
121.91
116.11
115.93
115.59
114.60
59.22
59.20
59.17
59.14
55.25
f1 (ppm)

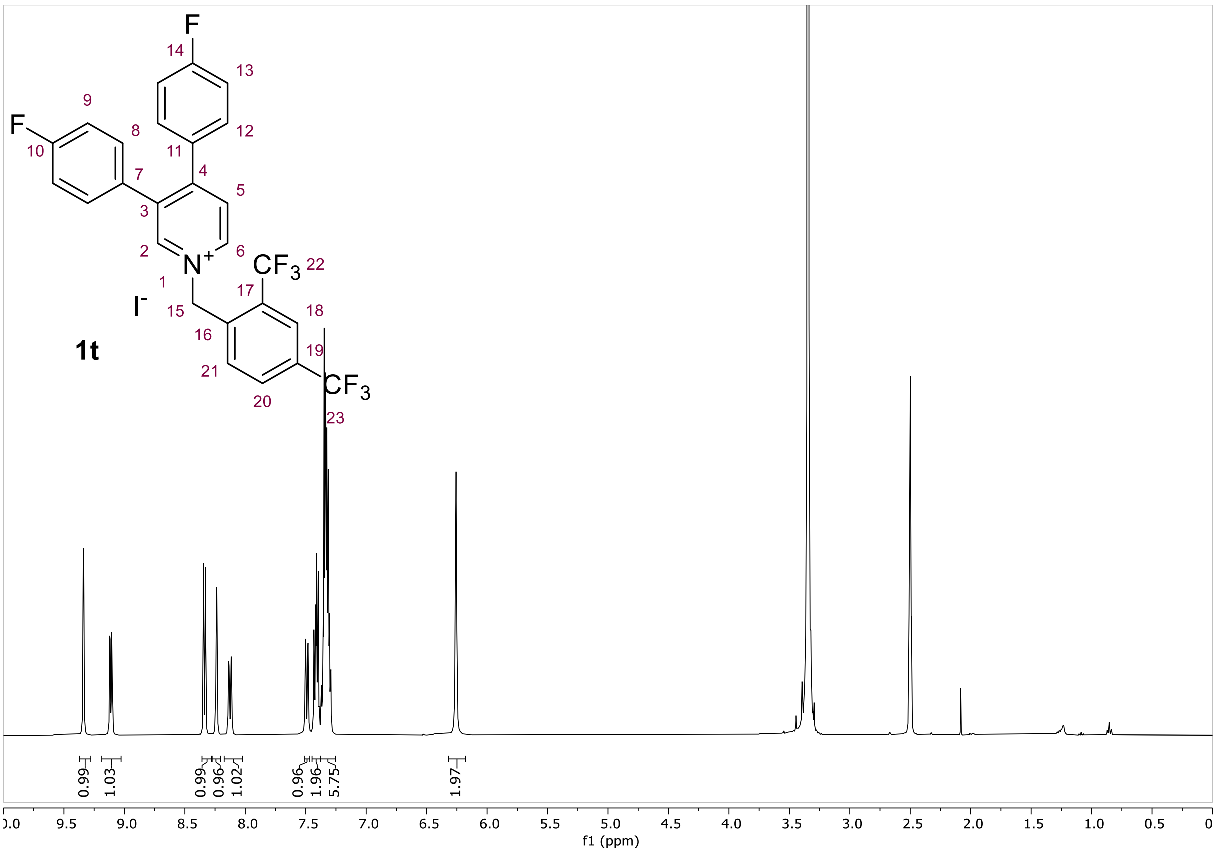
1t
F
I-
CF3
CF3
0.99
1.03
0.99
0.96
1.02
0.96
1.96
5.75
1.97
f1 (ppm)

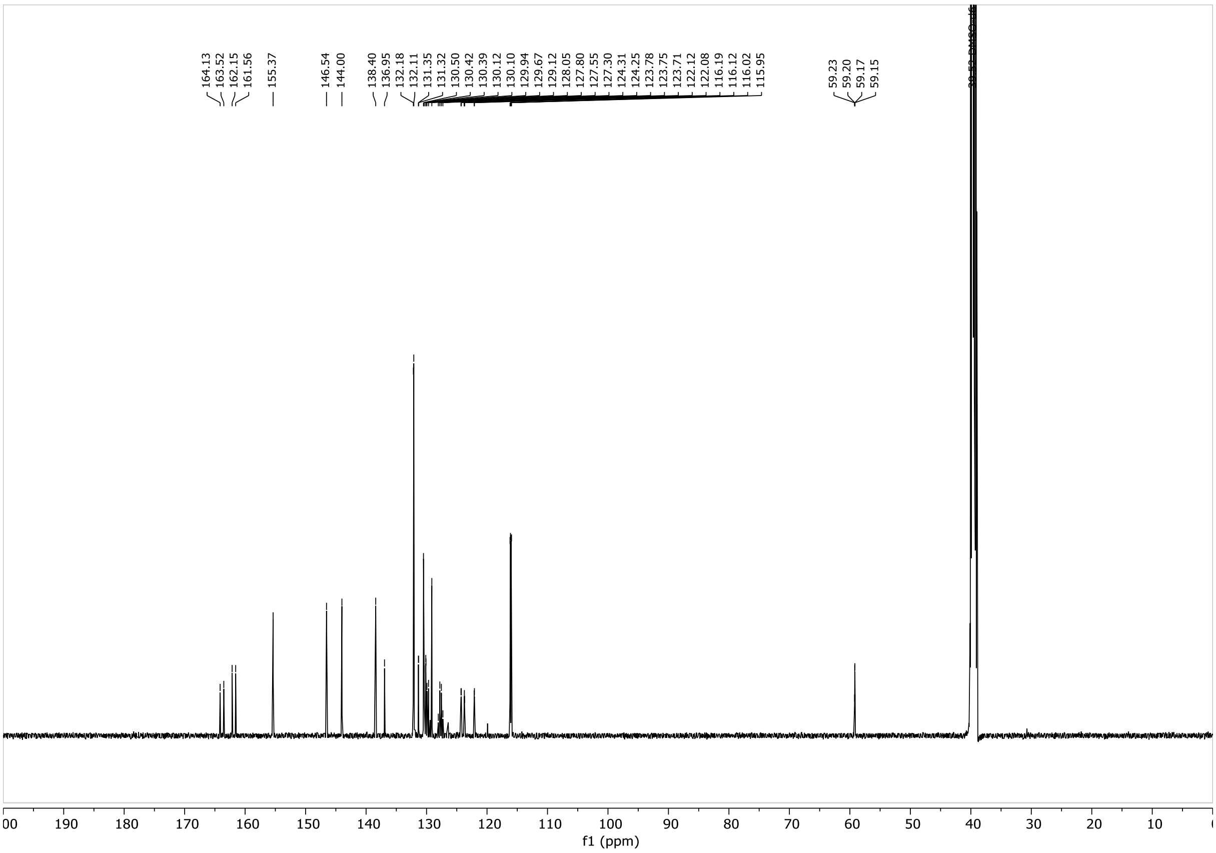
164.13
163.52
162.15
161.56
155.37
146.54
144.00
138.40
136.95
132.18
132.11
131.35
131.32
130.50
130.42
130.39
130.12
130.10
129.94
129.67
129.12
128.05
127.80
127.55
127.30
124.31
124.25
123.78
123.75
123.71
122.12
122.08
116.19
116.12
116.02
115.95
59.23
59.20
59.17
59.15
f1 (ppm)

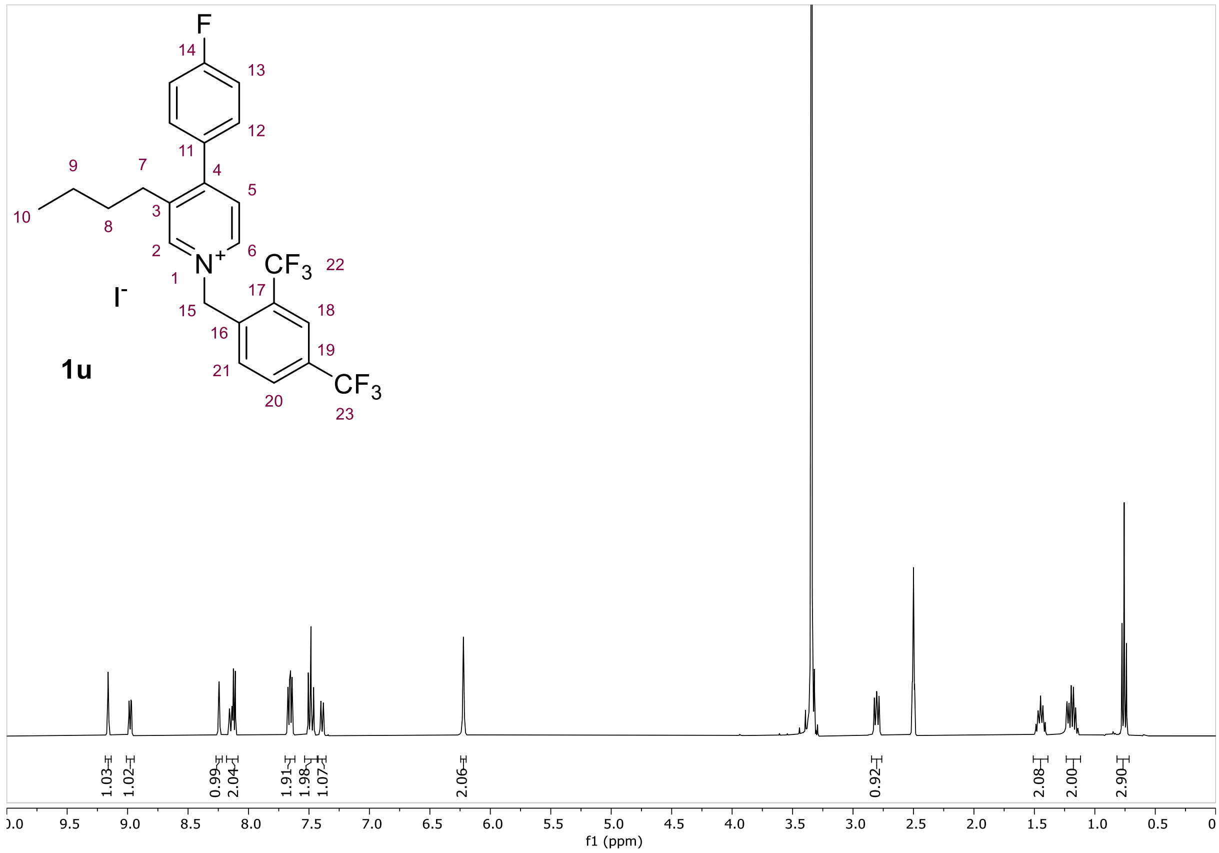

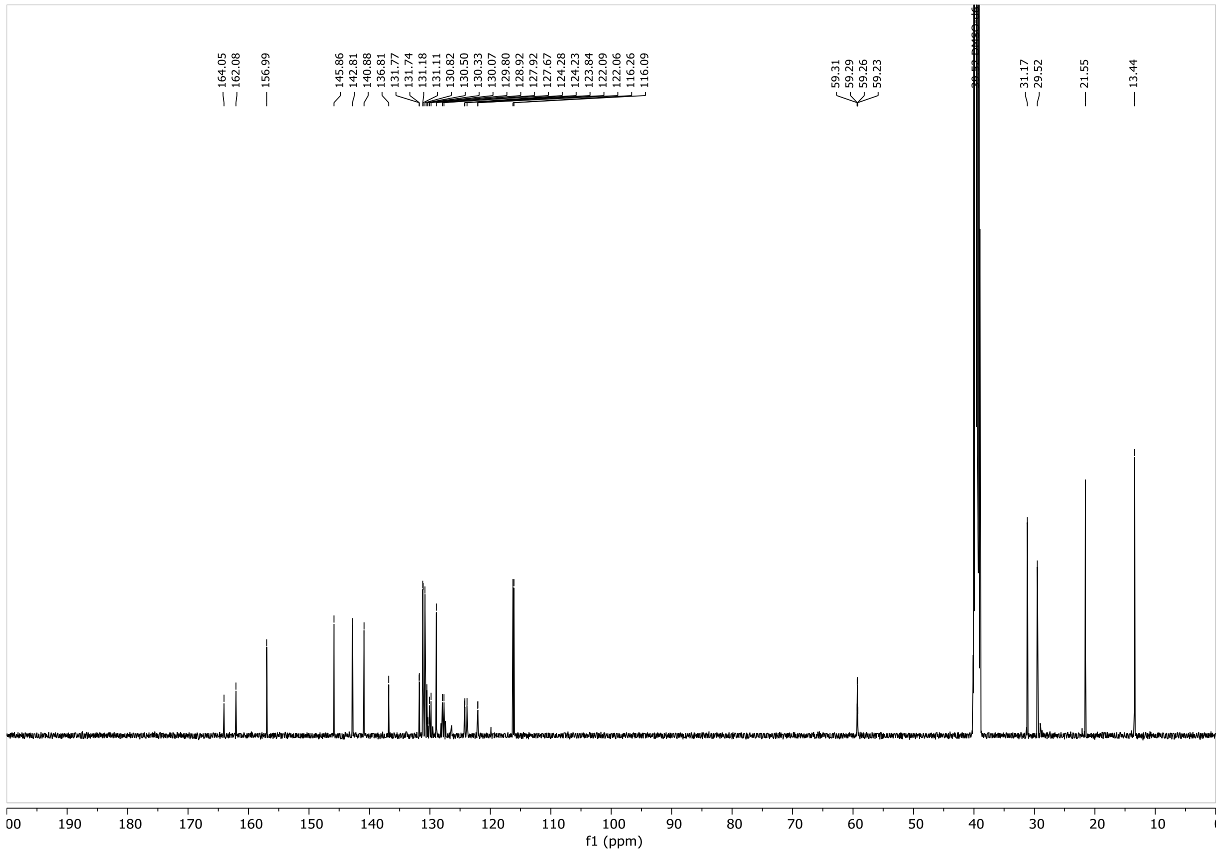

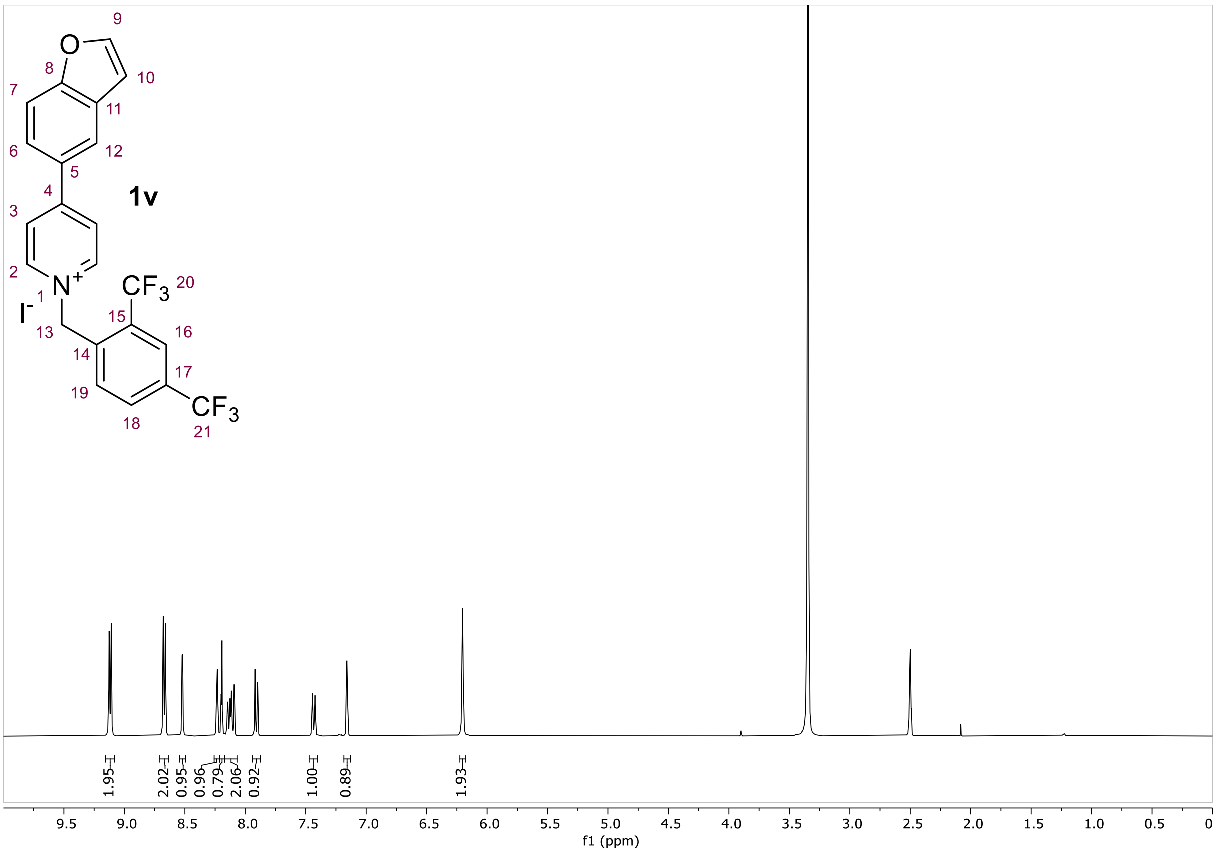

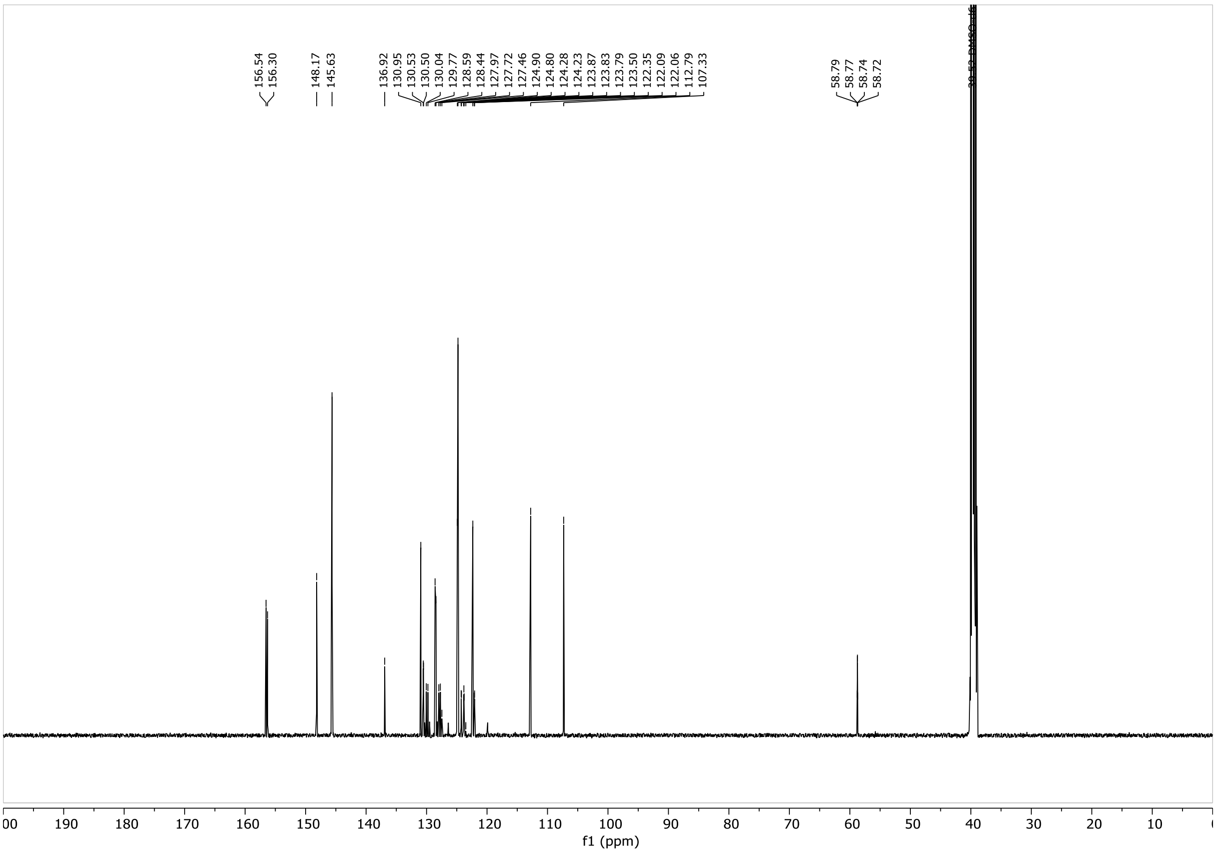

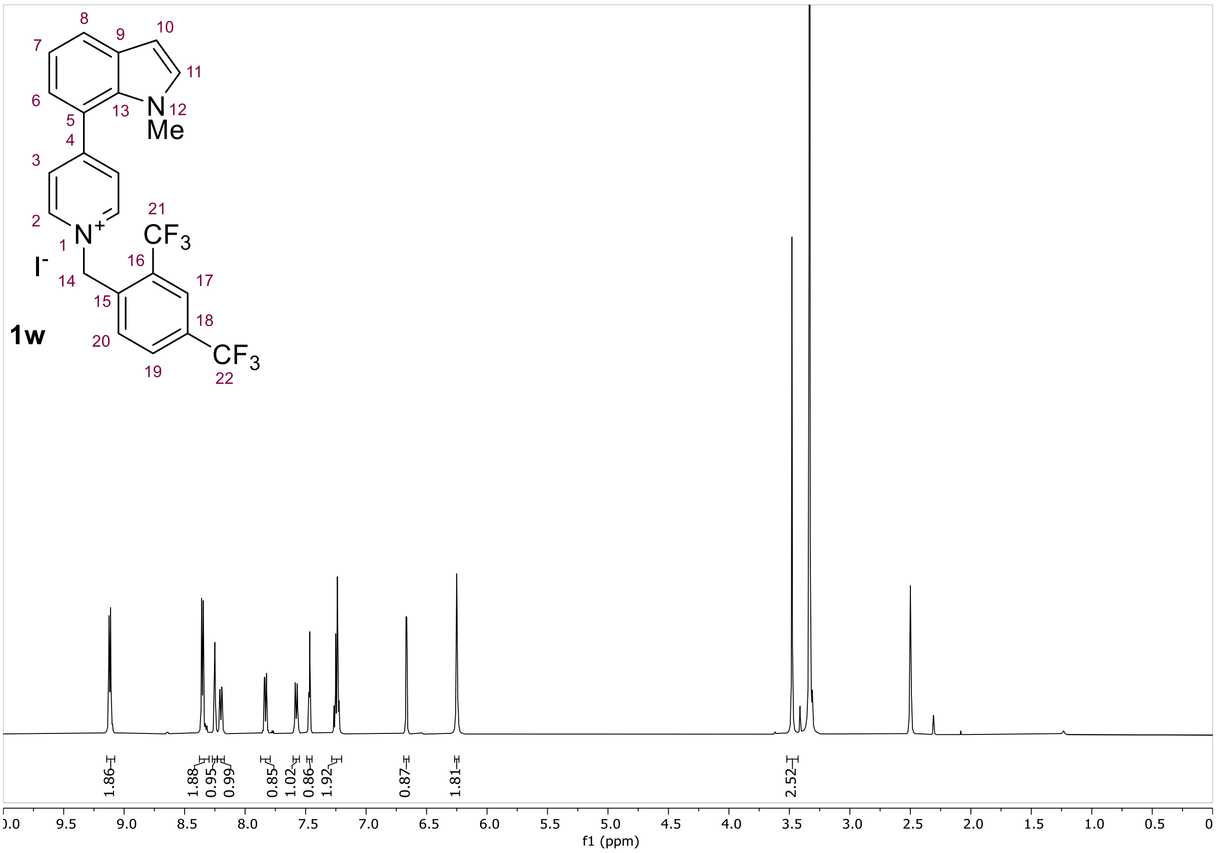

1w
Me
N
N^+
I^-
CF_3
CF_3
1.86
1.88
0.95
0.99
0.85
1.02
0.86
1.92
0.87
1.81
2.52
f1 (ppm)

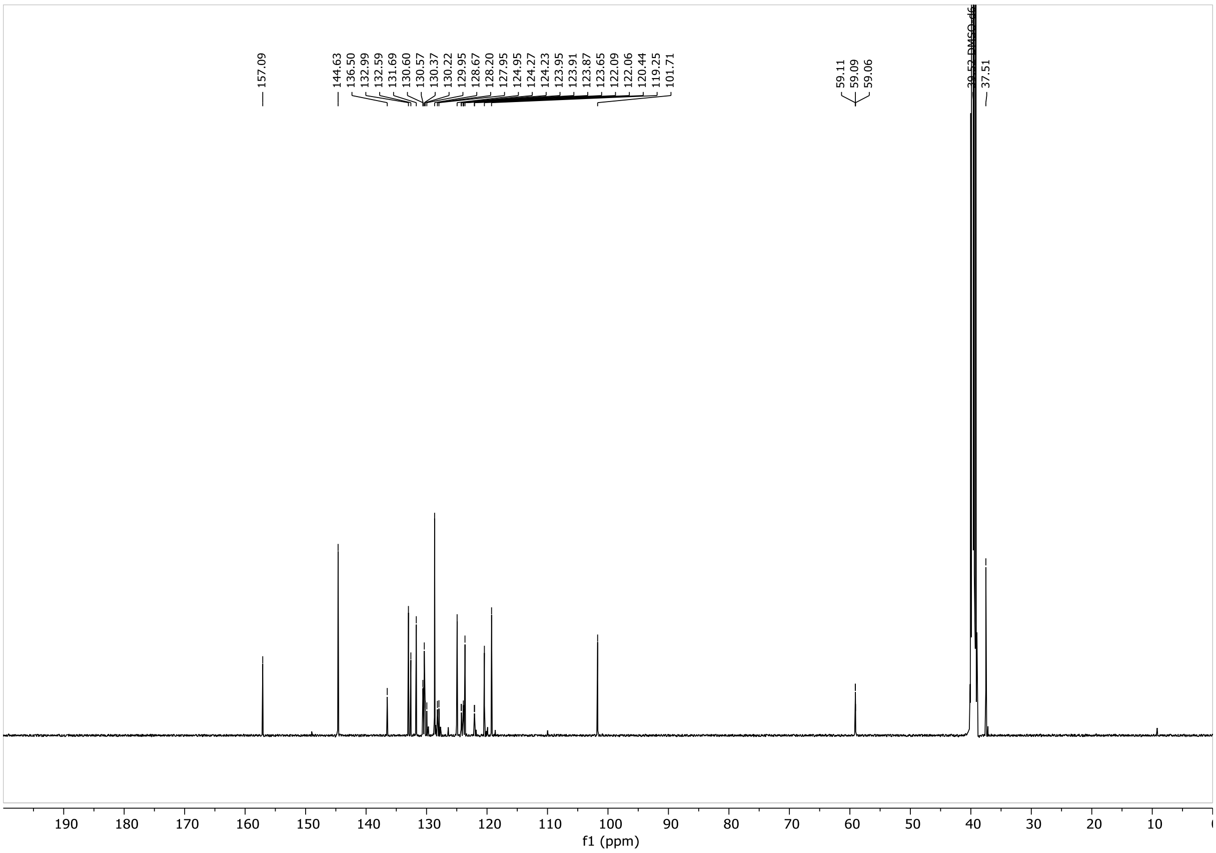

157.09
144.63
136.50
132.99
132.59
131.69
130.60
130.57
130.37
130.22
129.95
128.67
128.20
127.95
124.95
124.27
124.23
123.95
123.91
123.87
123.65
122.09
122.06
120.44
119.25
101.71
59.11
59.09
59.06
39.52 DMSO-d6
37.51
f1 (ppm)

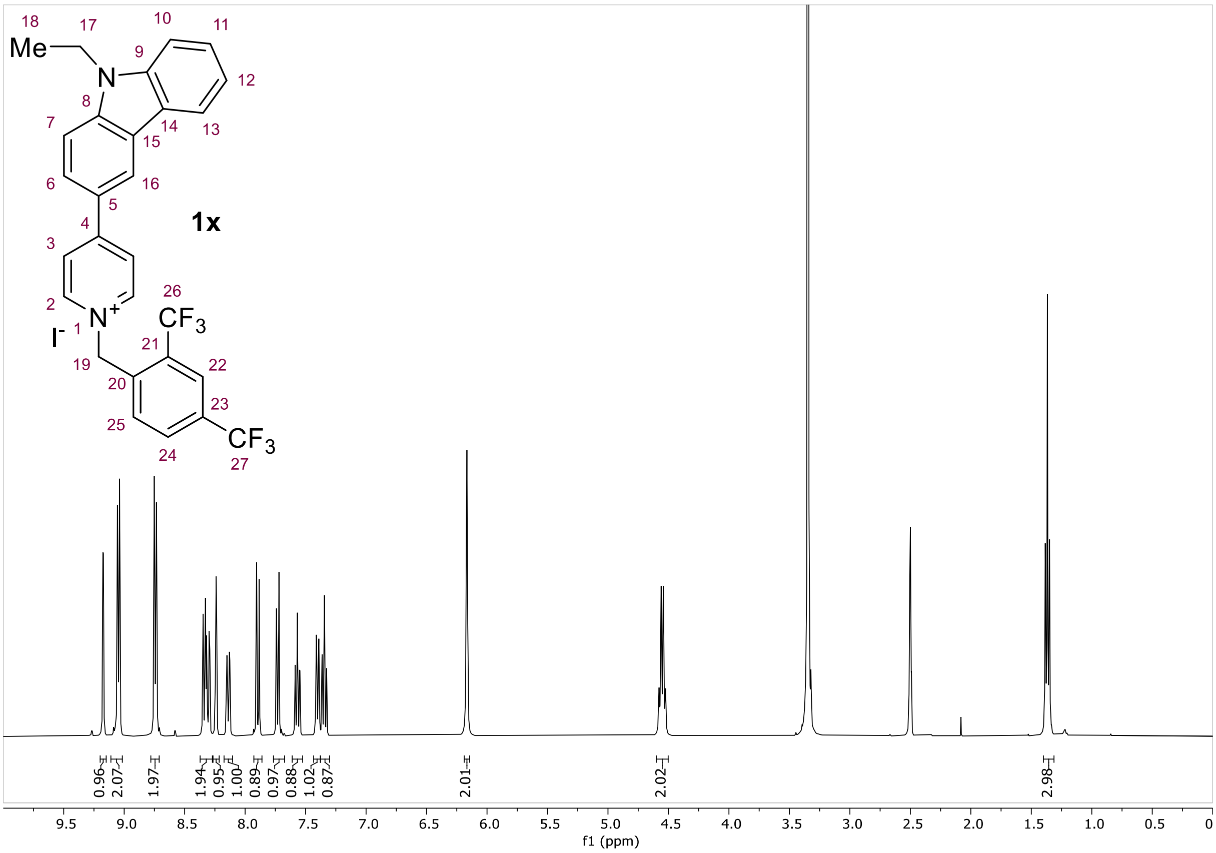
1x
Me
N
N+
I-
CF3
CF3
0.96
2.07
1.97
1.94
0.95
1.00
0.89
0.97
0.88
1.02
0.87
2.01
2.02
2.98
f1 (ppm)

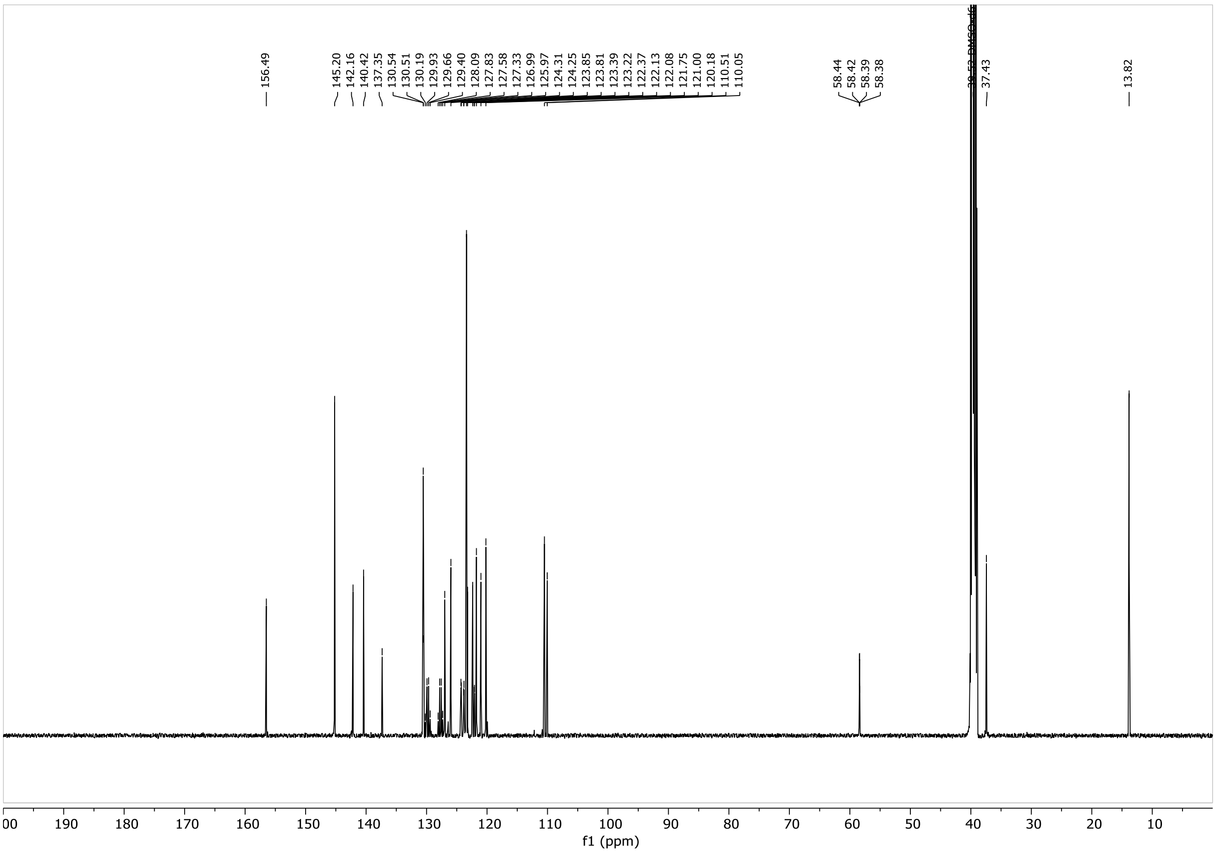
156.49
145.20
142.16
140.42
137.35
130.54
130.51
130.19
129.93
129.66
129.40
128.09
127.83
127.58
127.33
126.99
125.97
124.31
124.25
123.85
123.81
123.39
123.22
122.37
122.13
122.08
121.75
121.00
120.18
110.51
110.05
58.44
58.42
58.39
58.38
37.43
13.82
f1 (ppm)

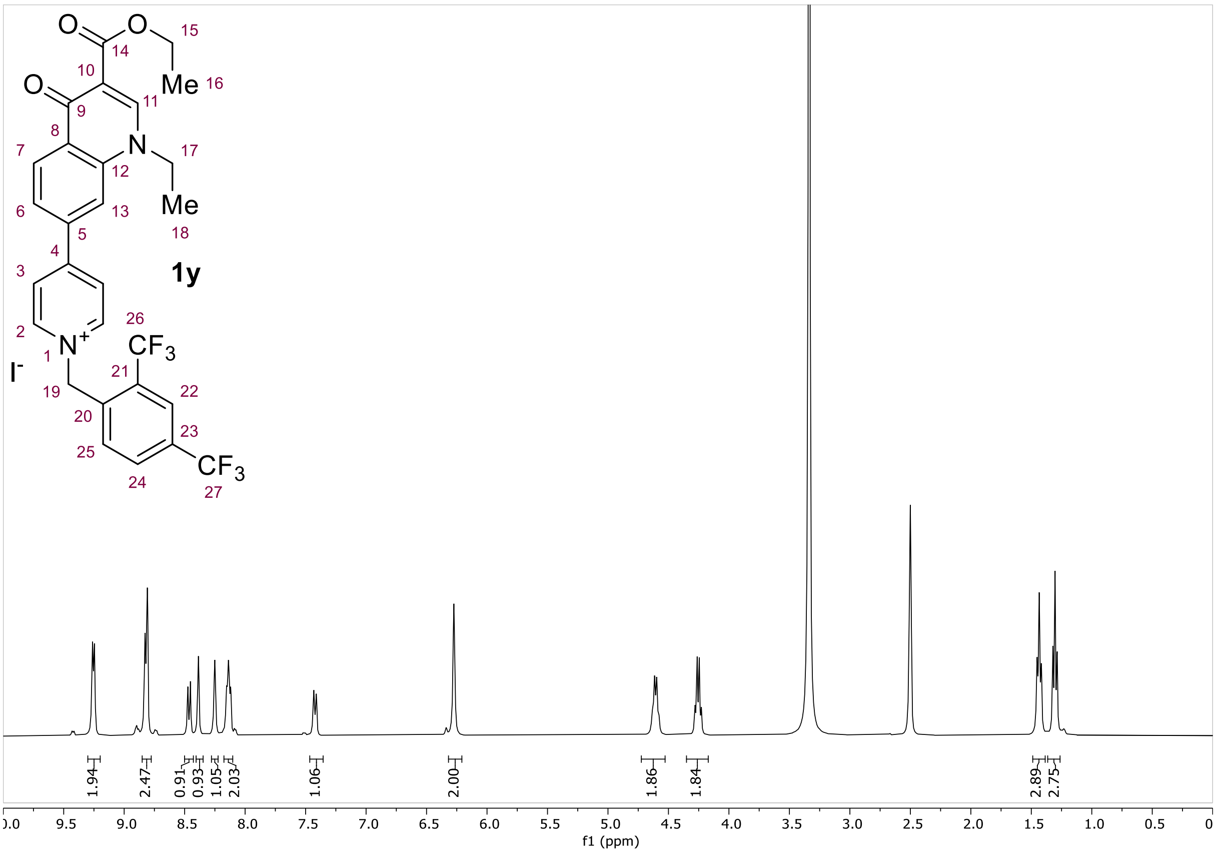
1y
1.94
2.47
0.91
0.93
1.05
2.03
1.06
2.00
1.86
1.84
2.89
2.75
f1 (ppm)

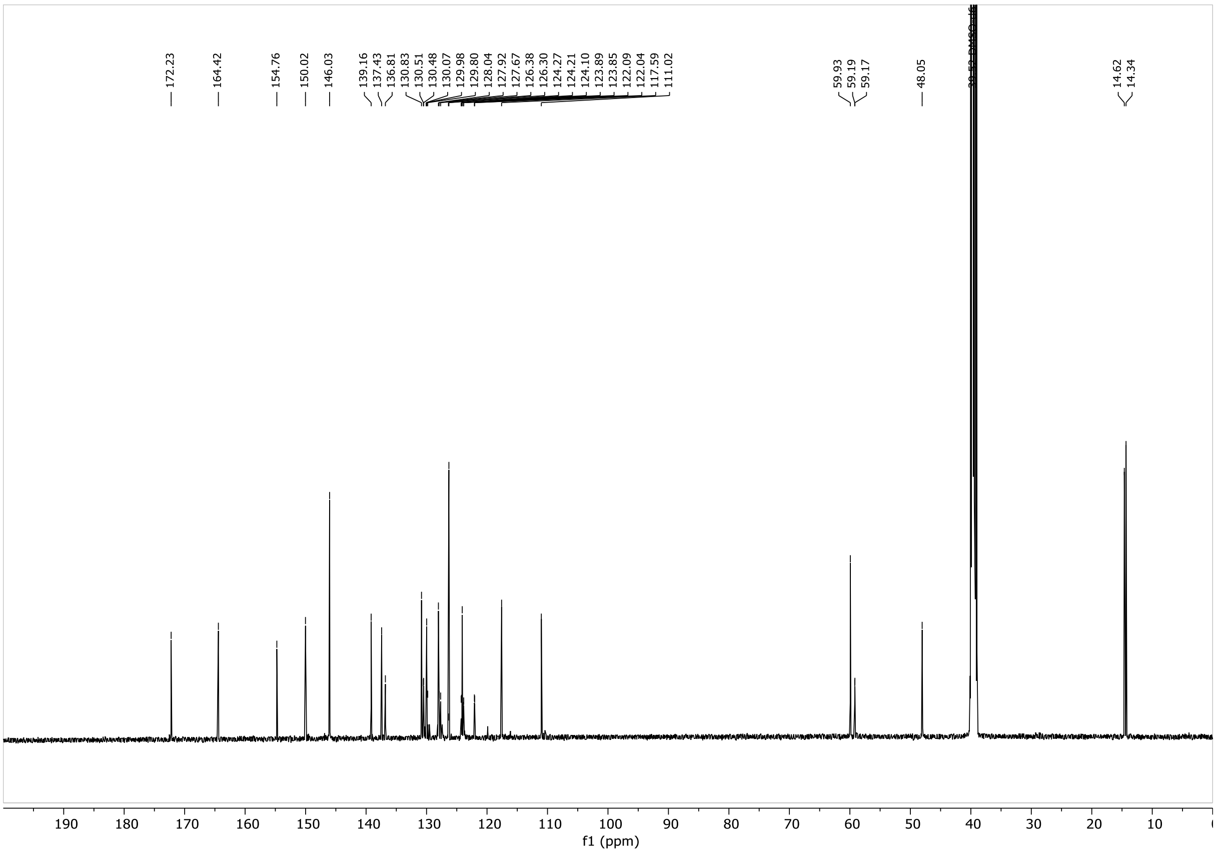
172.23
164.42
154.76
150.02
146.03
139.16
137.43
136.81
130.83
130.51
130.48
130.07
129.98
129.80
128.04
127.92
127.67
126.38
126.30
124.27
124.21
124.10
123.89
123.85
122.09
122.04
117.59
111.02
59.93
59.19
59.17
48.05
14.62
14.34
f1 (ppm)

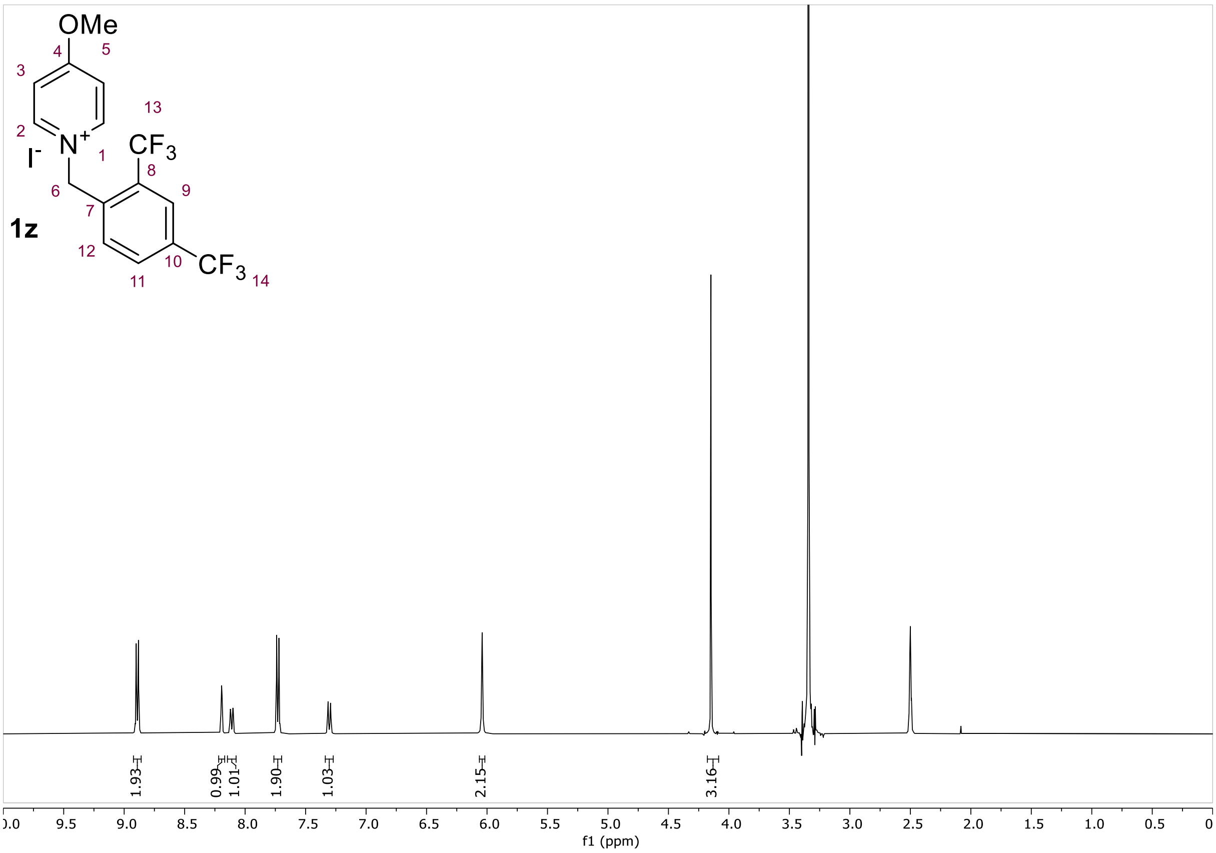
OMe
I-
N+
CF3
CF3
1z
1.93
0.99
1.01
1.90
1.03
2.15
3.16
f1 (ppm)

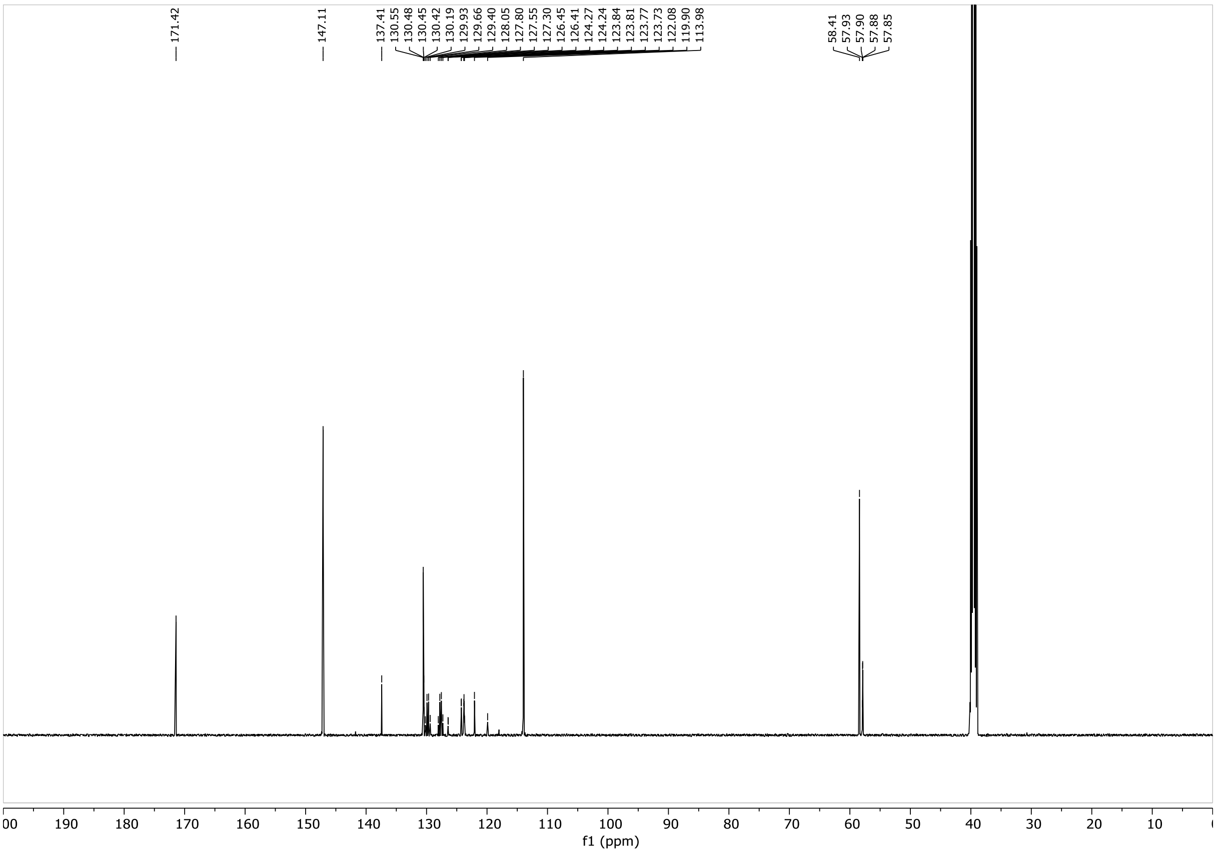
171.42
147.11
137.41
130.55
130.48
130.45
130.42
130.19
129.93
129.66
129.40
128.05
127.80
127.55
127.30
126.45
126.41
124.27
124.24
123.84
123.81
123.77
123.73
122.08
119.90
113.98
58.41
57.93
57.90
57.88
57.85
f1 (ppm)

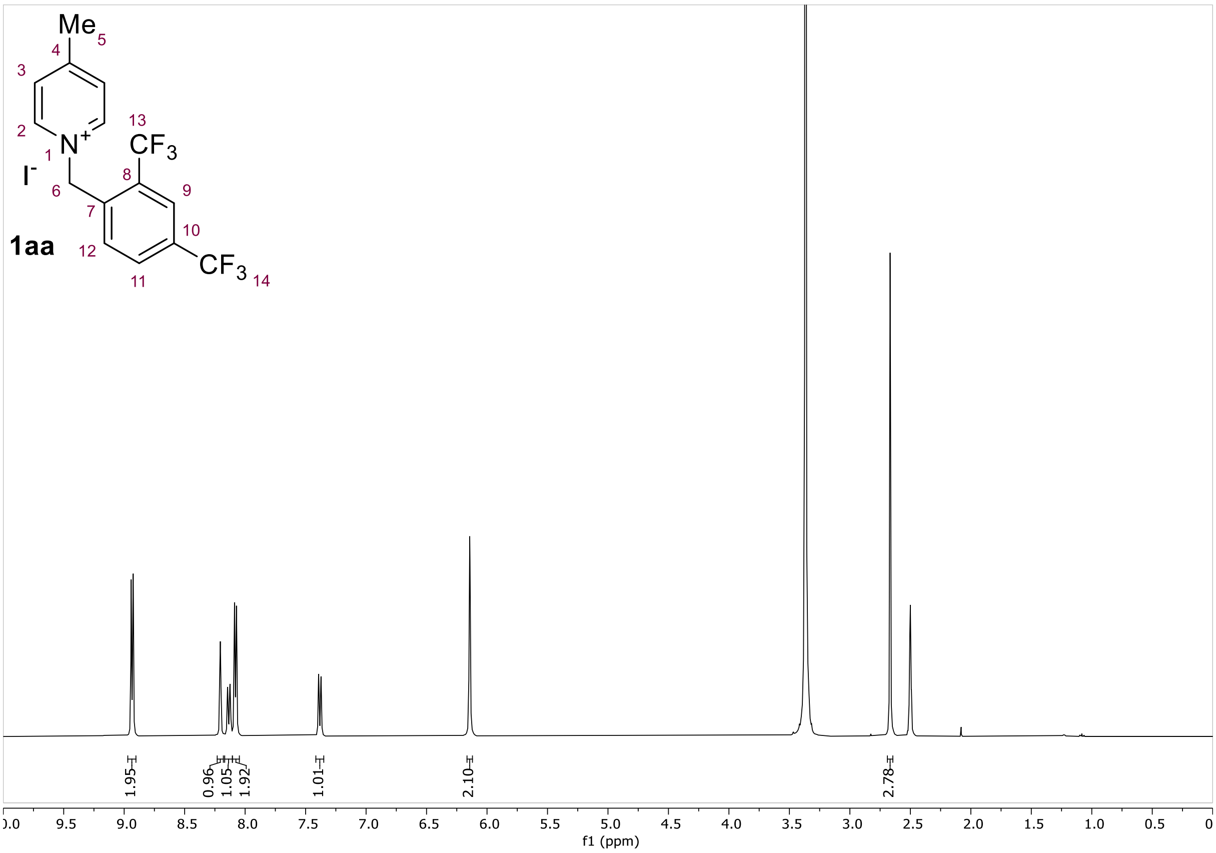
Me
1aa
f1 (ppm)

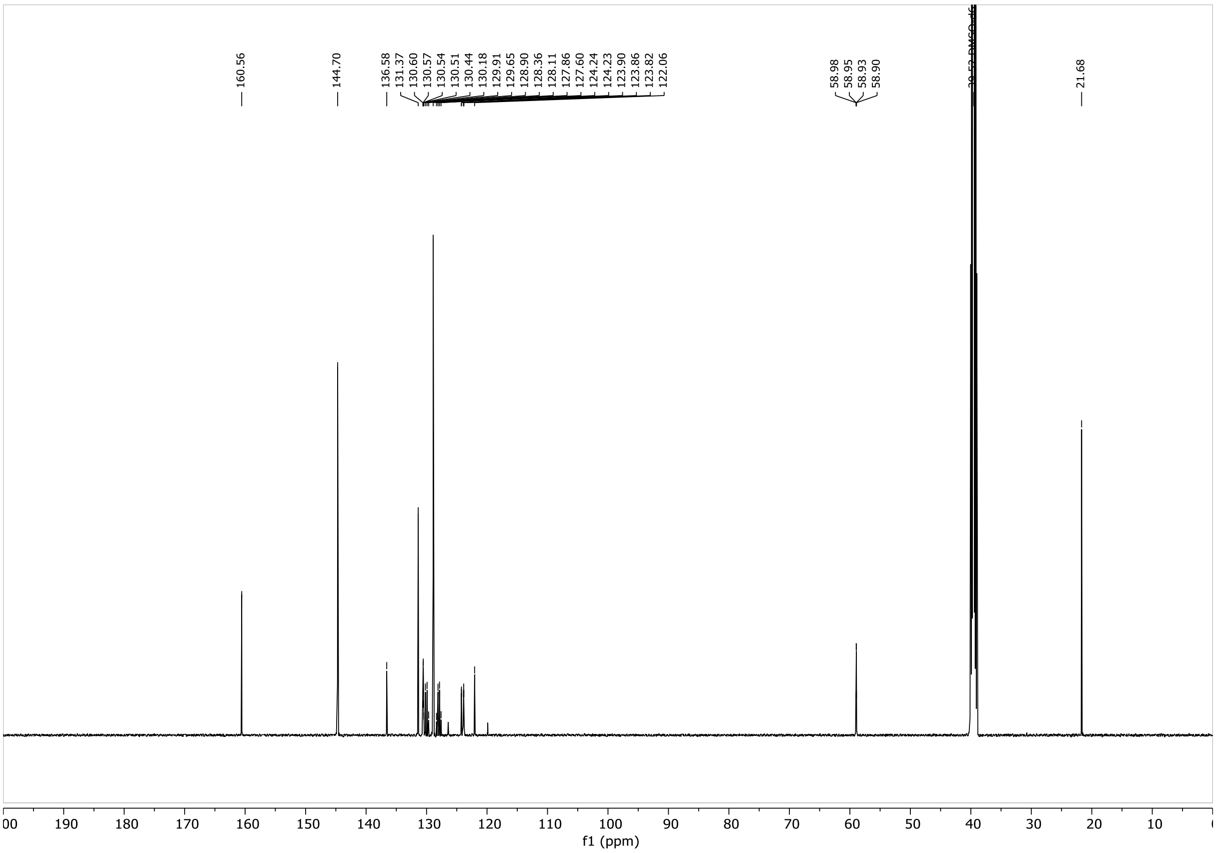
160.56
144.70
136.58
131.37
130.60
130.57
130.54
130.51
130.44
130.18
129.91
129.65
128.90
128.36
128.11
127.86
127.60
124.24
124.23
123.90
123.86
123.82
122.06
58.98
58.95
58.93
58.90
21.68
f1 (ppm)

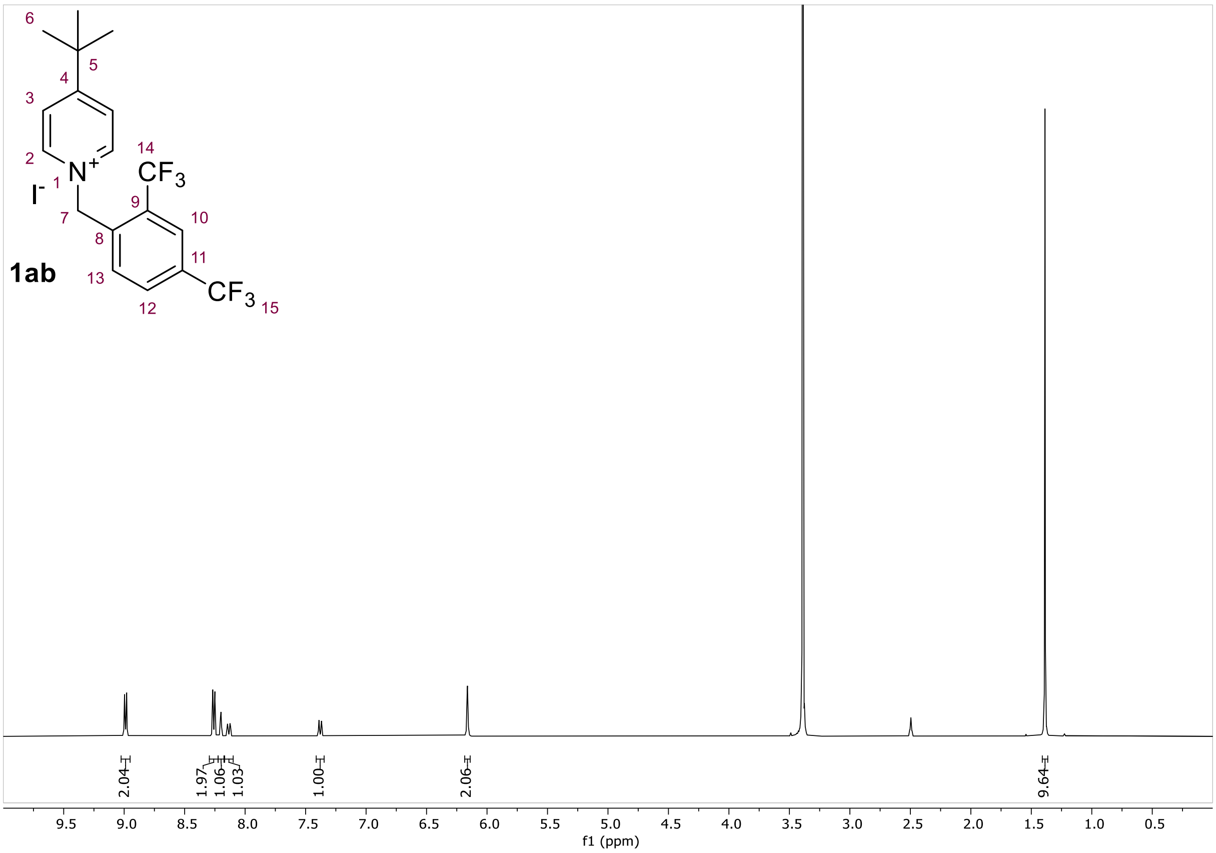

6
5
4
3
2
14
N+
CF3
I-
1
7
9
10
8
11
1ab
13
CF3
15
12
2.04
1.97
1.06
1.03
1.00
2.06
9.64
9.5
9.0
8.5
8.0
7.5
7.0
6.5
6.0
5.5
5.0
4.5
4.0
3.5
3.0
2.5
2.0
1.5
1.0
0.5
f1 (ppm)

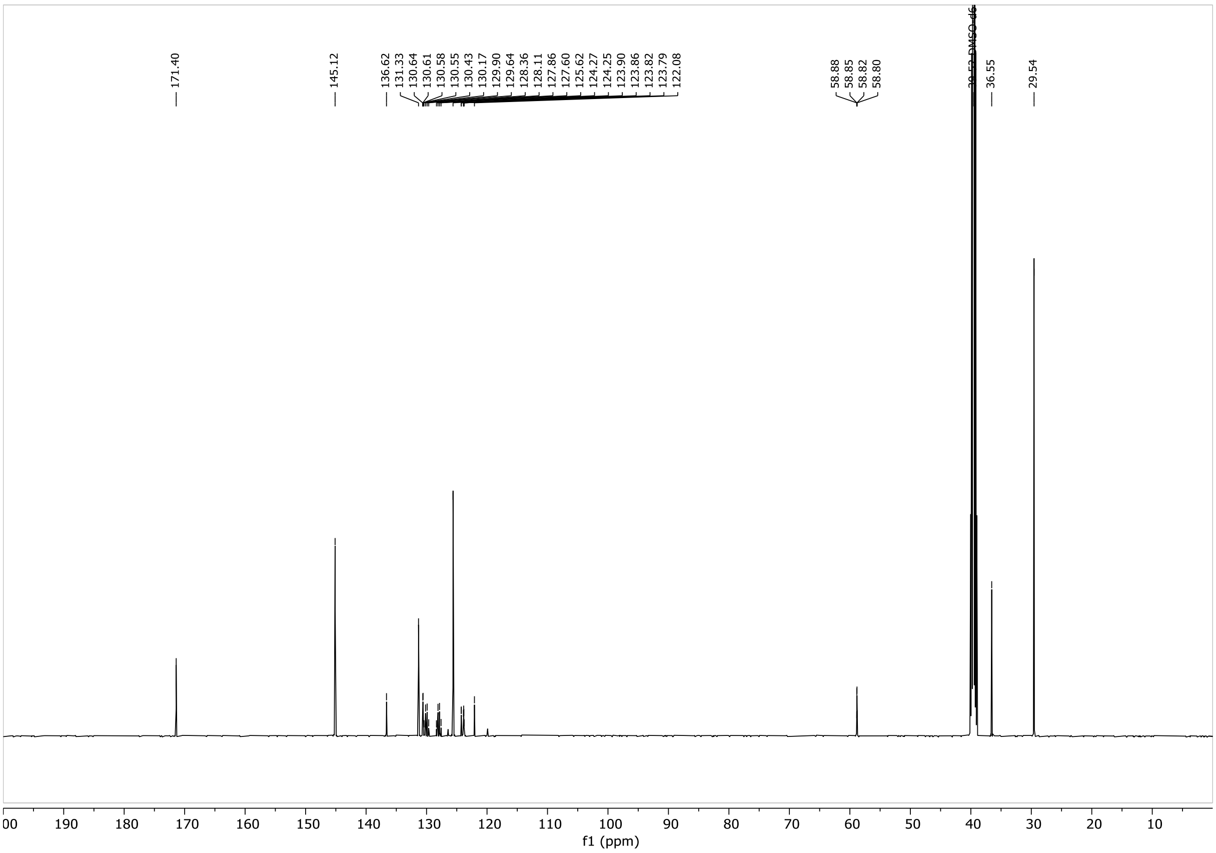

171.40
145.12
136.62
131.33
130.64
130.61
130.58
130.55
130.43
130.17
129.90
129.64
128.36
128.11
127.86
127.60
125.62
124.27
124.25
123.90
123.86
123.82
123.79
122.08
58.88
58.85
58.82
58.80
36.55
29.54
00
190
180
170
160
150
140
130
120
110
100
90
80
70
60
50
40
30
20
10
f1 (ppm)

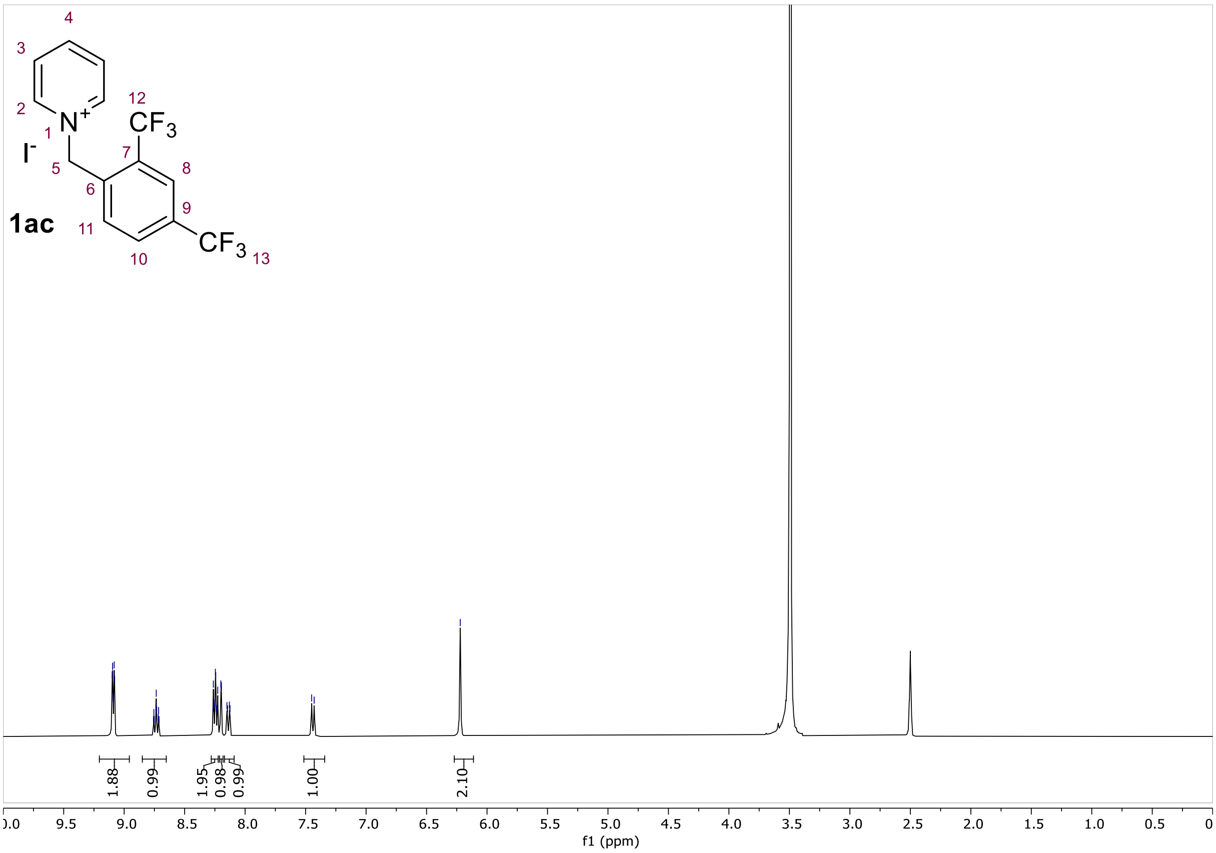
1ac
I^-
N^+
CF_3
CF_3
1
2
3
4
5
6
7
8
9
10
11
12
13
1.88
0.99
1.95
0.98
0.99
1.00
2.10
f1 (ppm)

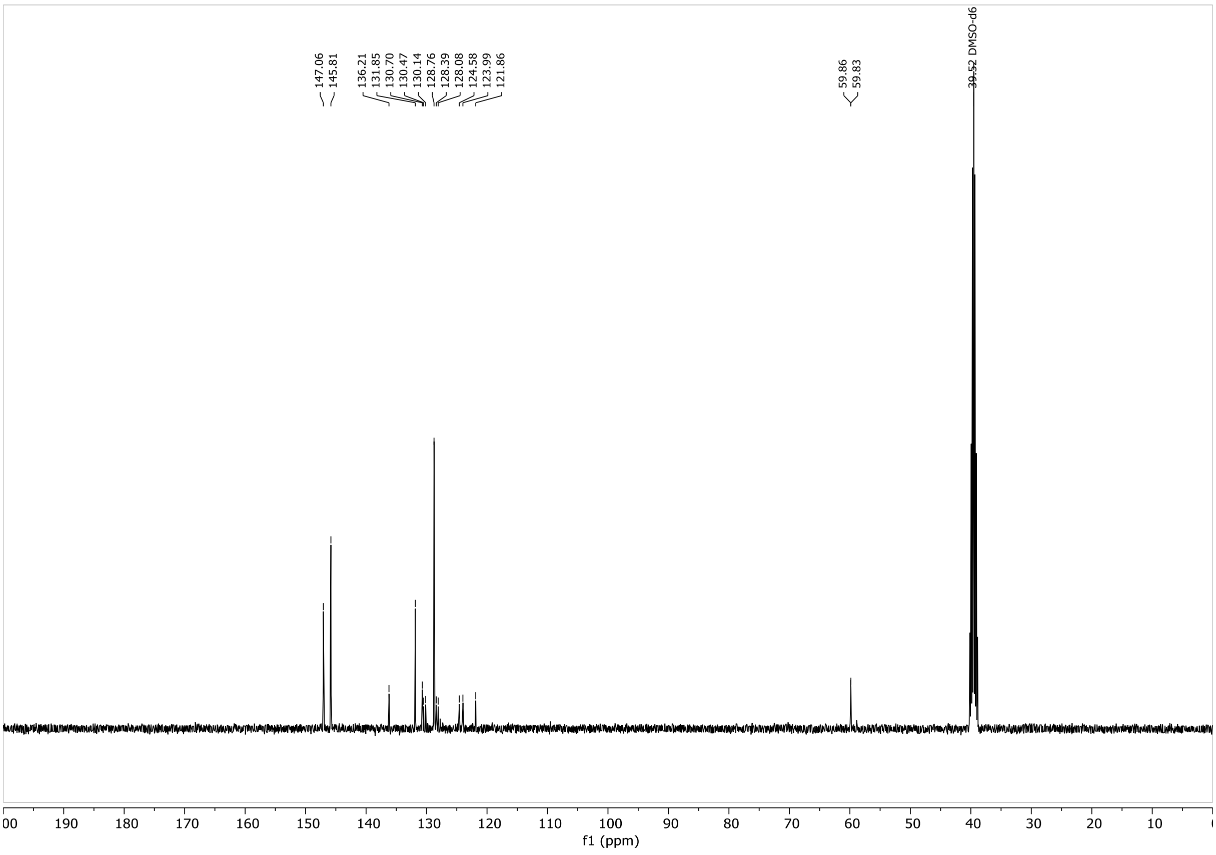
147.06
145.81
136.21
131.85
130.70
130.47
130.14
128.76
128.39
128.08
124.58
123.99
121.86
59.86
59.83
39.52 DMSO-d6
f1 (ppm)

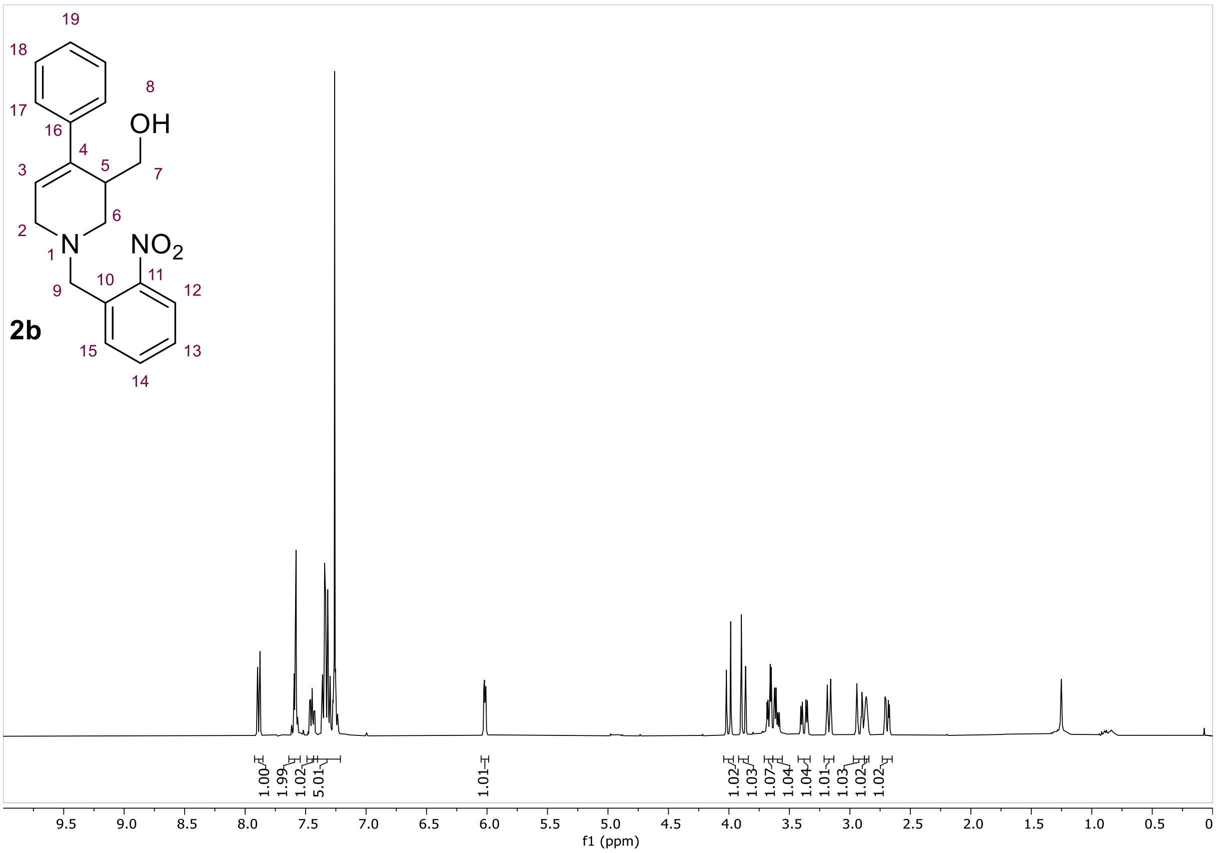
2b
NO2
OH
1.00
1.99
1.02
5.01
1.01
1.02
1.03
1.07
1.04
1.04
1.01
1.03
1.02
1.02
f1 (ppm)

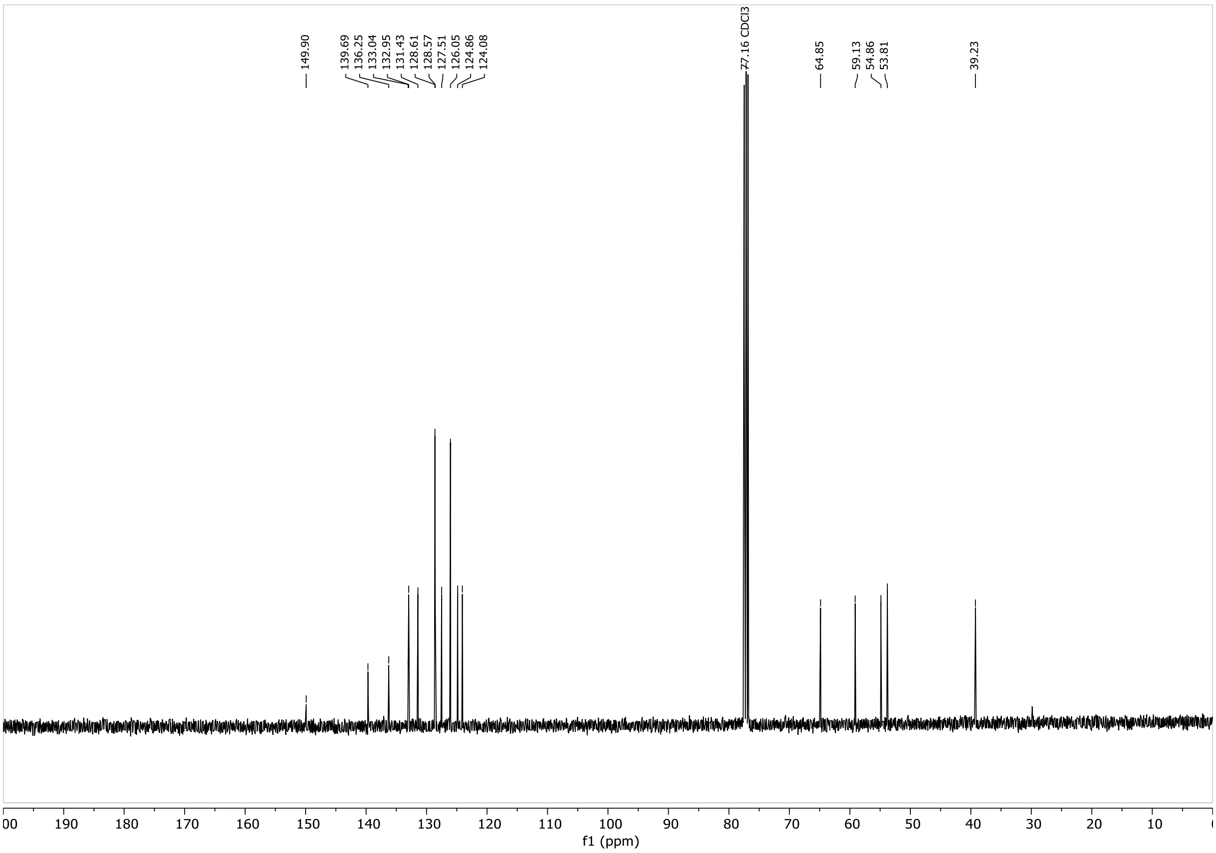
149.90
139.69
136.25
133.04
132.95
131.43
128.61
128.57
127.51
126.05
124.86
124.08
77.16 CDCl3
64.85
59.13
54.86
53.81
39.23
f1 (ppm)

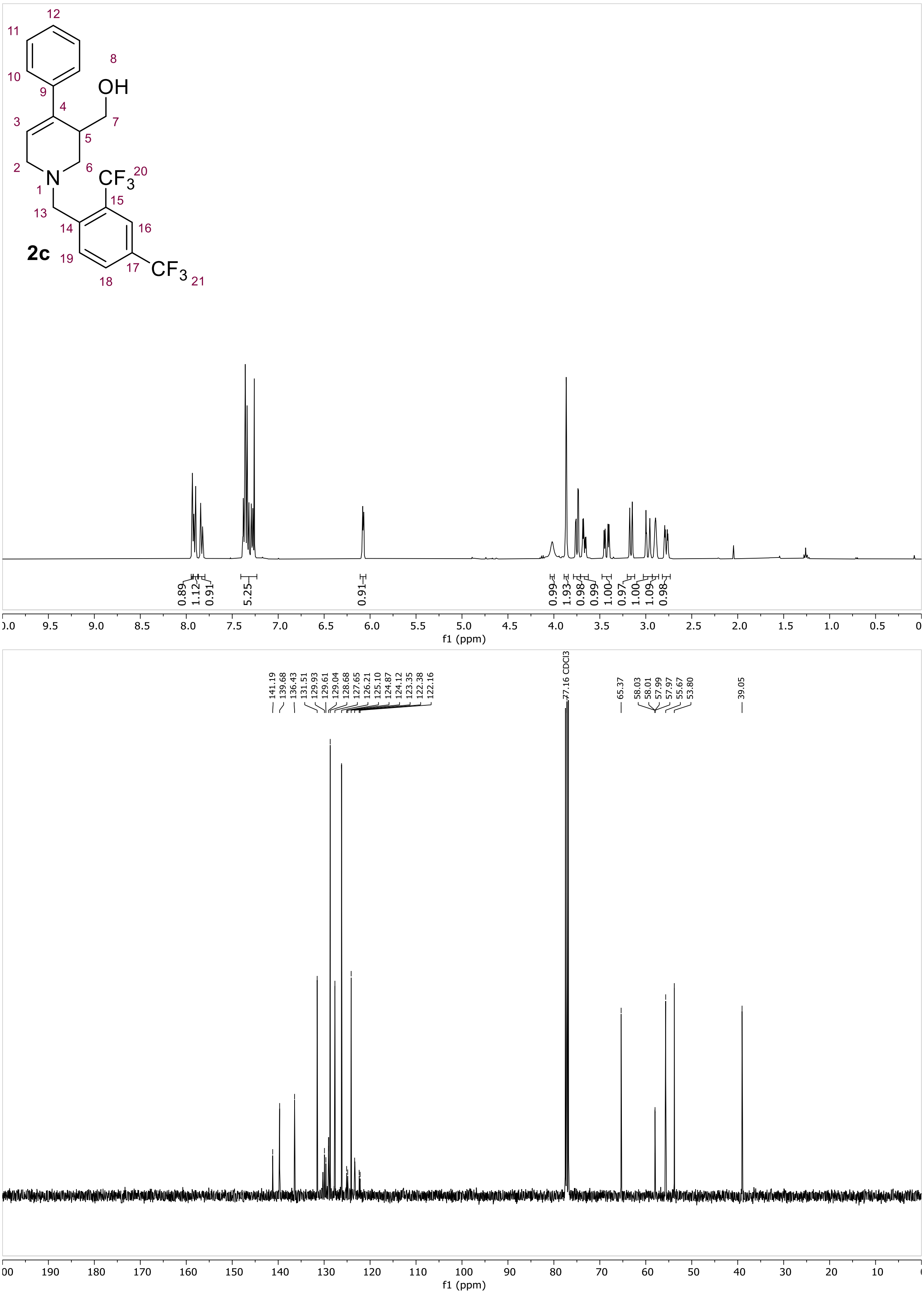

2c
OH
CF3
CF3
N
0.89
1.12
0.91
5.25
0.91
0.99
1.93
0.98
0.99
1.00
0.97
1.00
1.09
0.98
f1 (ppm)
141.19
139.68
136.43
131.51
129.93
129.61
129.04
128.68
127.65
126.21
125.10
124.87
124.12
123.35
122.38
122.16
77.16 CDCl3
65.37
58.03
58.01
57.99
57.97
55.67
53.80
39.05
f1 (ppm)

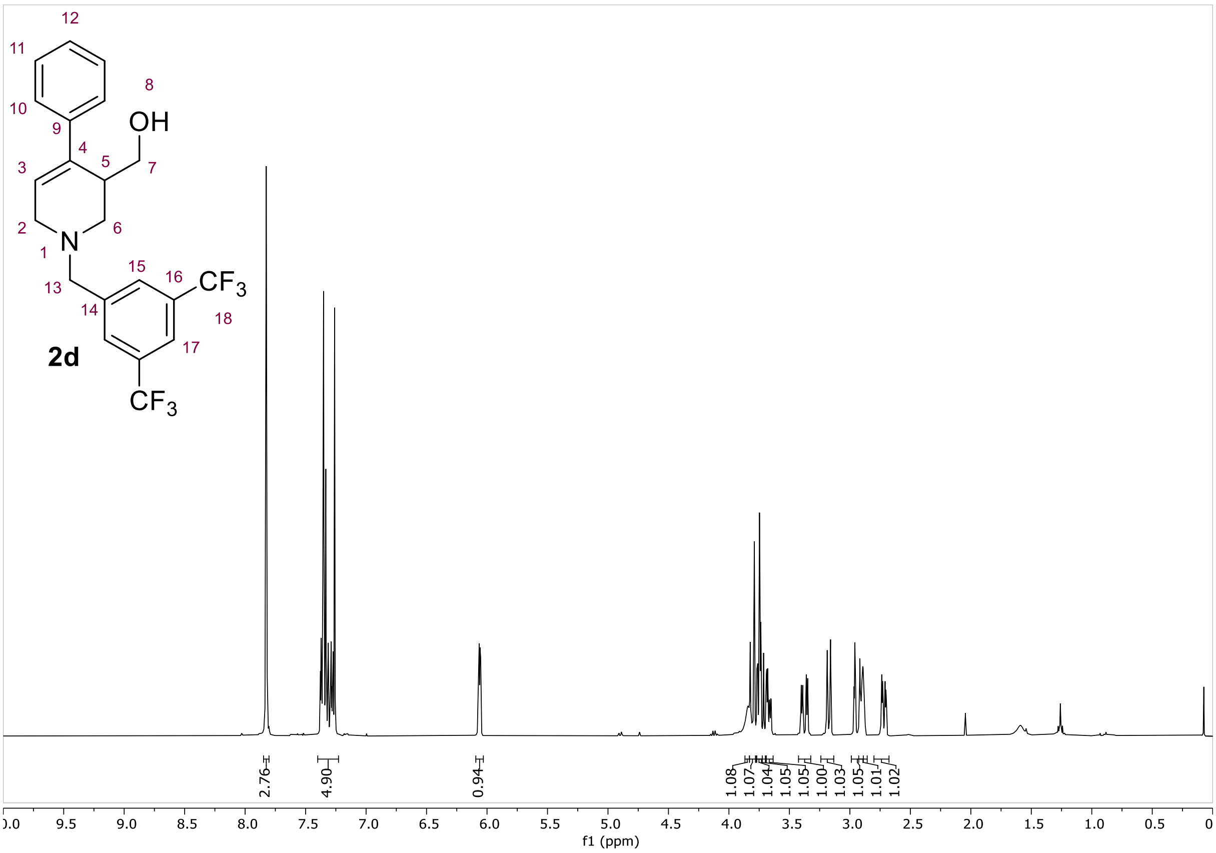
2d
OH
N
CF3
CF3
1
2
3
4
5
6
7
8
9
10
11
12
13
14
15
16
17
18
2.76
4.90
0.94
1.08
1.07
1.04
1.05
1.05
1.00
1.03
1.05
1.01
1.02
f1 (ppm)

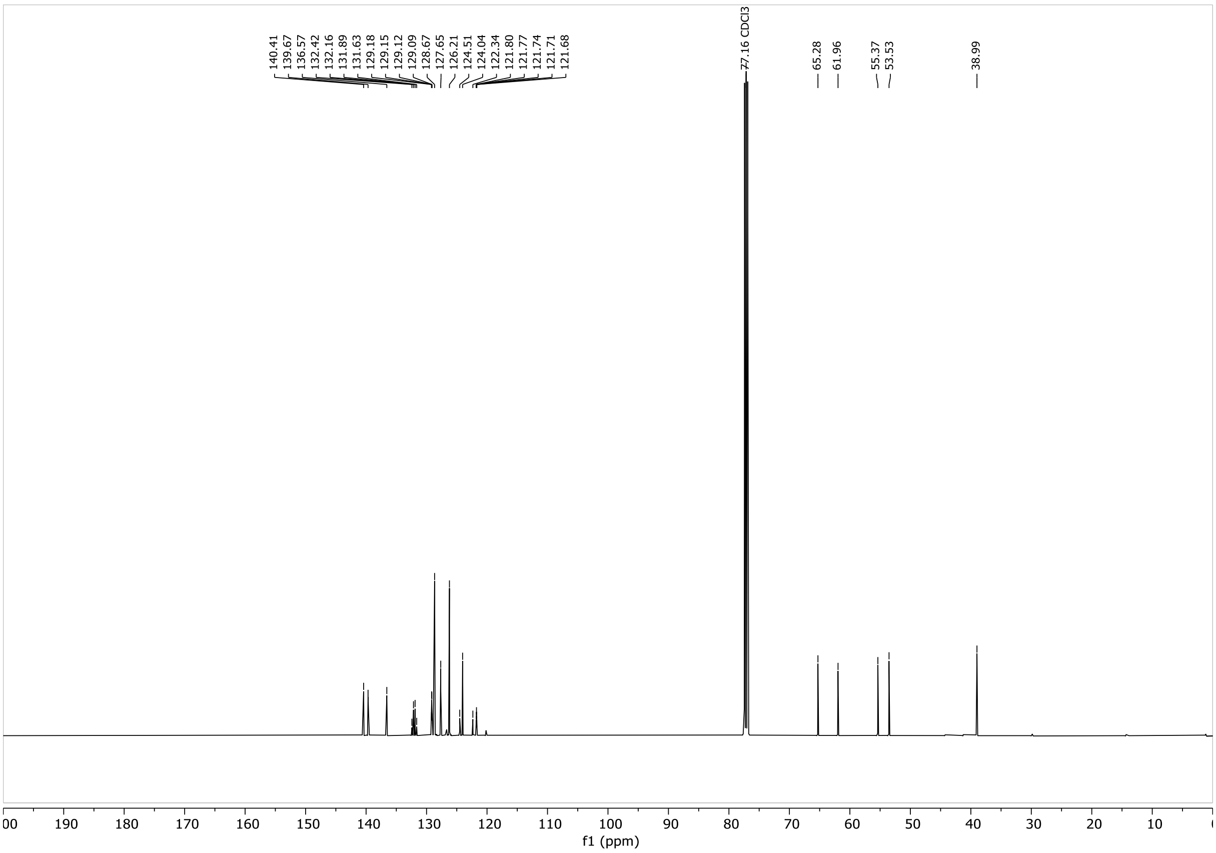
140.41
139.67
136.57
132.42
132.16
131.89
131.63
129.18
129.15
129.12
129.09
128.67
127.65
126.21
124.51
124.04
122.34
121.80
121.77
121.74
121.71
121.68
77.16 CDCl3
65.28
61.96
55.37
53.53
38.99
f1 (ppm)

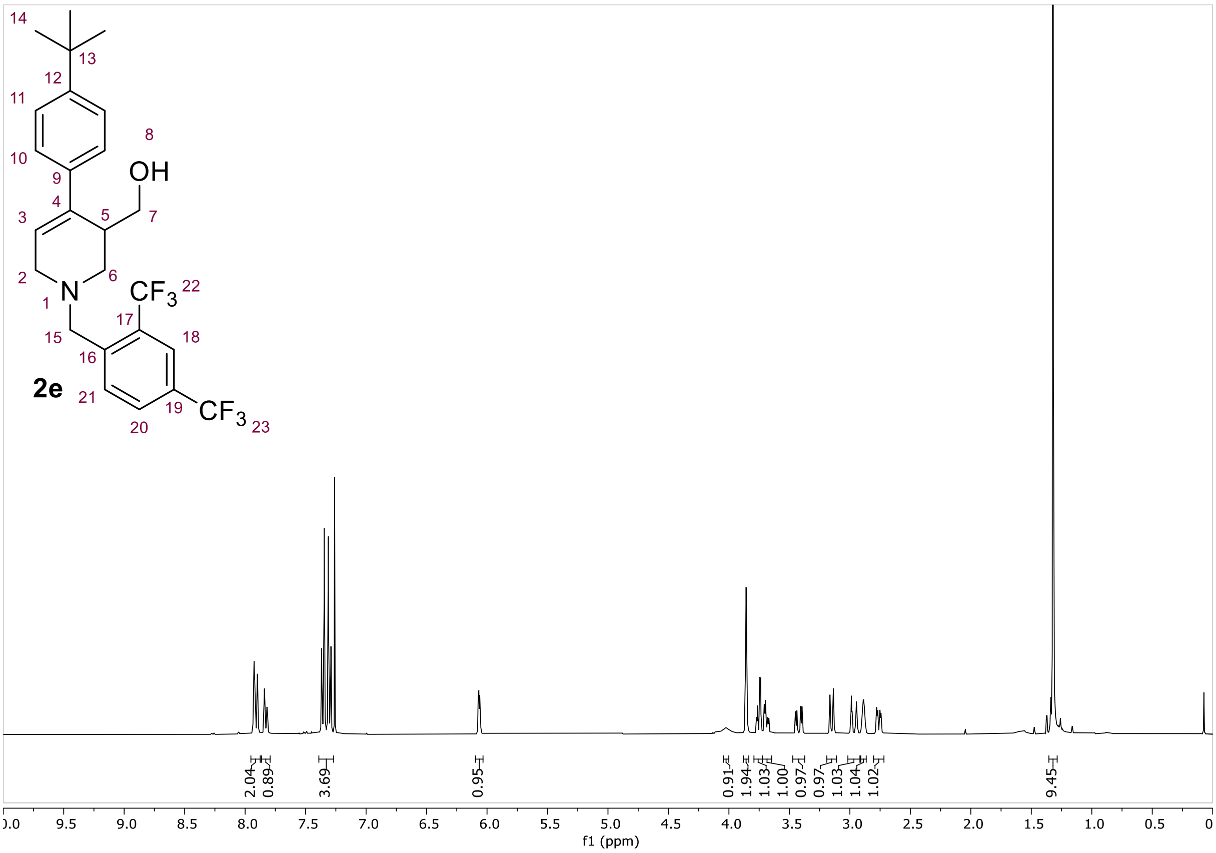
2e
OH
N
CF3
CF3
2.04
0.89
3.69
0.95
0.91
1.94
1.03
1.00
0.97
1.03
1.04
1.02
9.45
f1 (ppm)

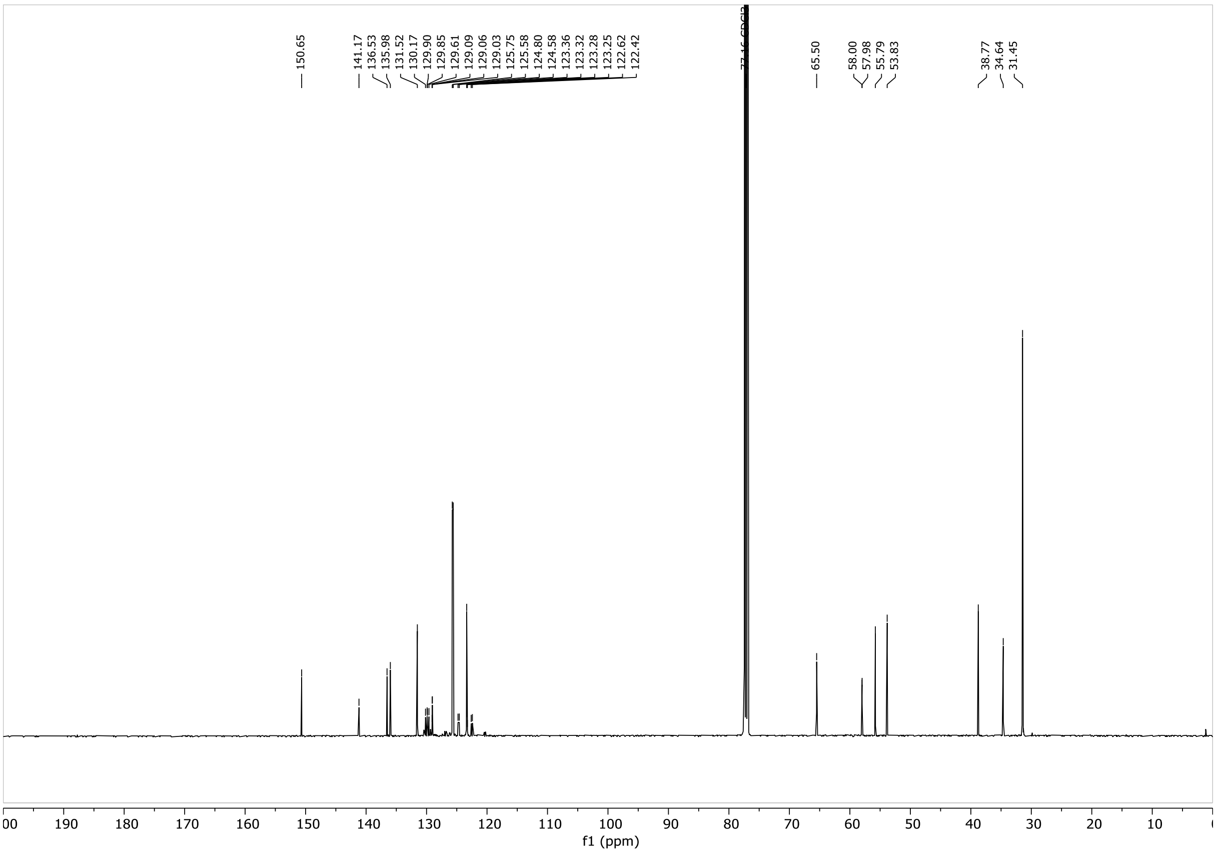
150.65
141.17
136.53
135.98
131.52
130.17
129.90
129.85
129.61
129.09
129.06
129.03
125.75
125.58
124.80
124.58
123.36
123.32
123.28
123.25
122.62
122.42
[illegible]
65.50
58.00
57.98
55.79
53.83
38.77
34.64
31.45
f1 (ppm)

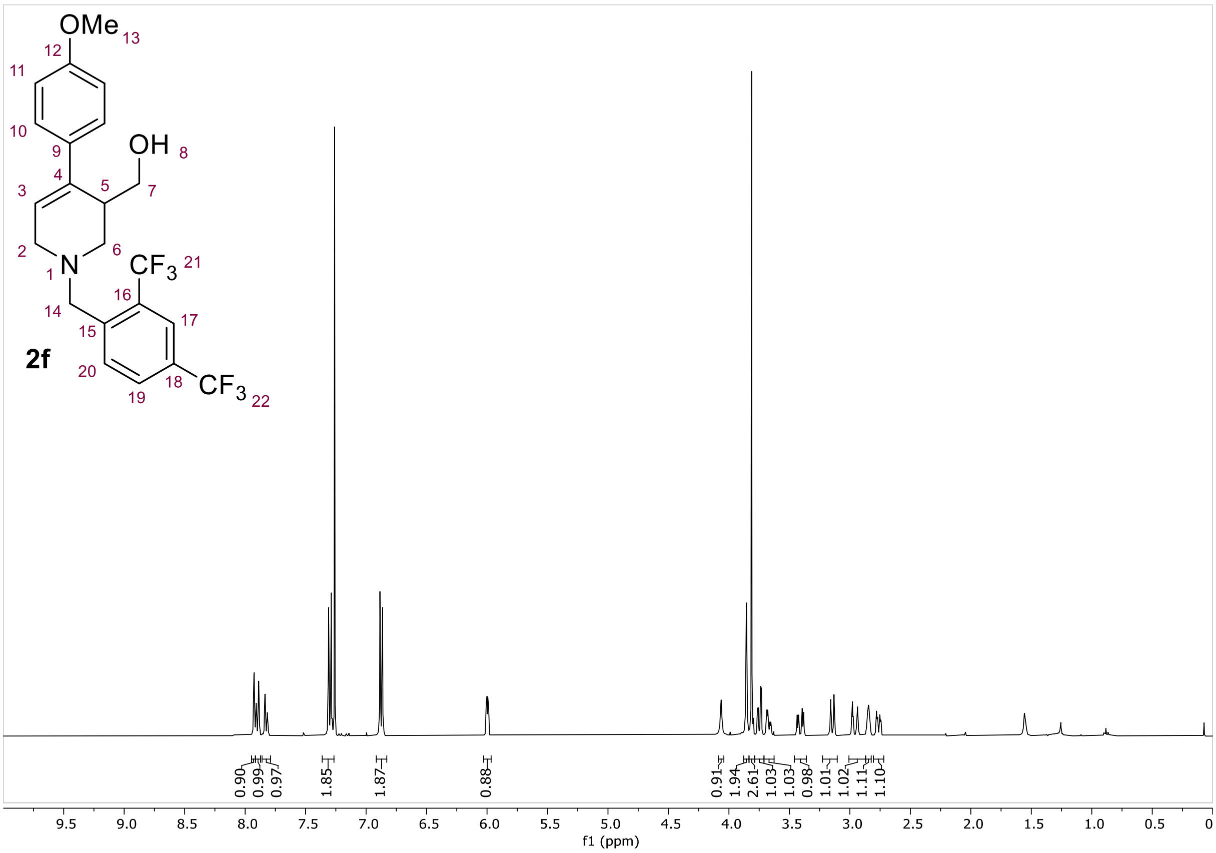

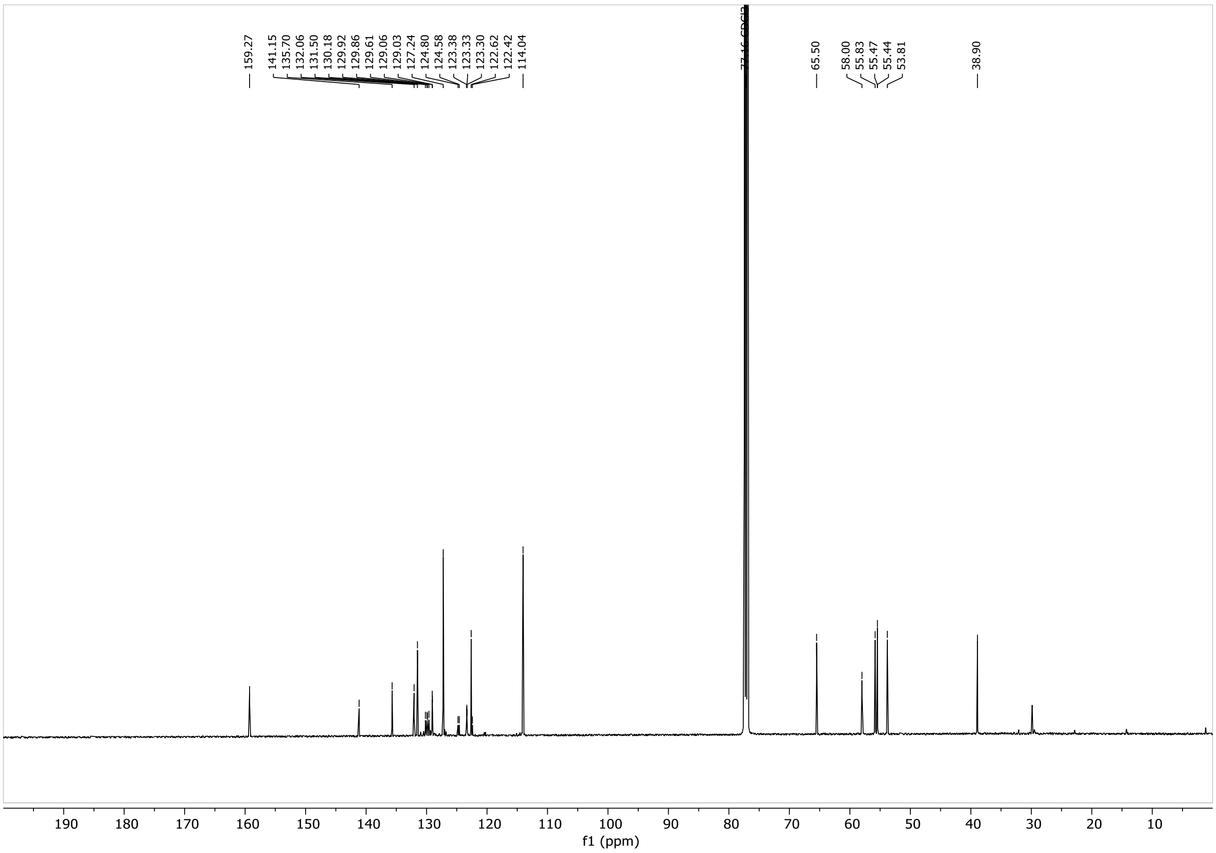

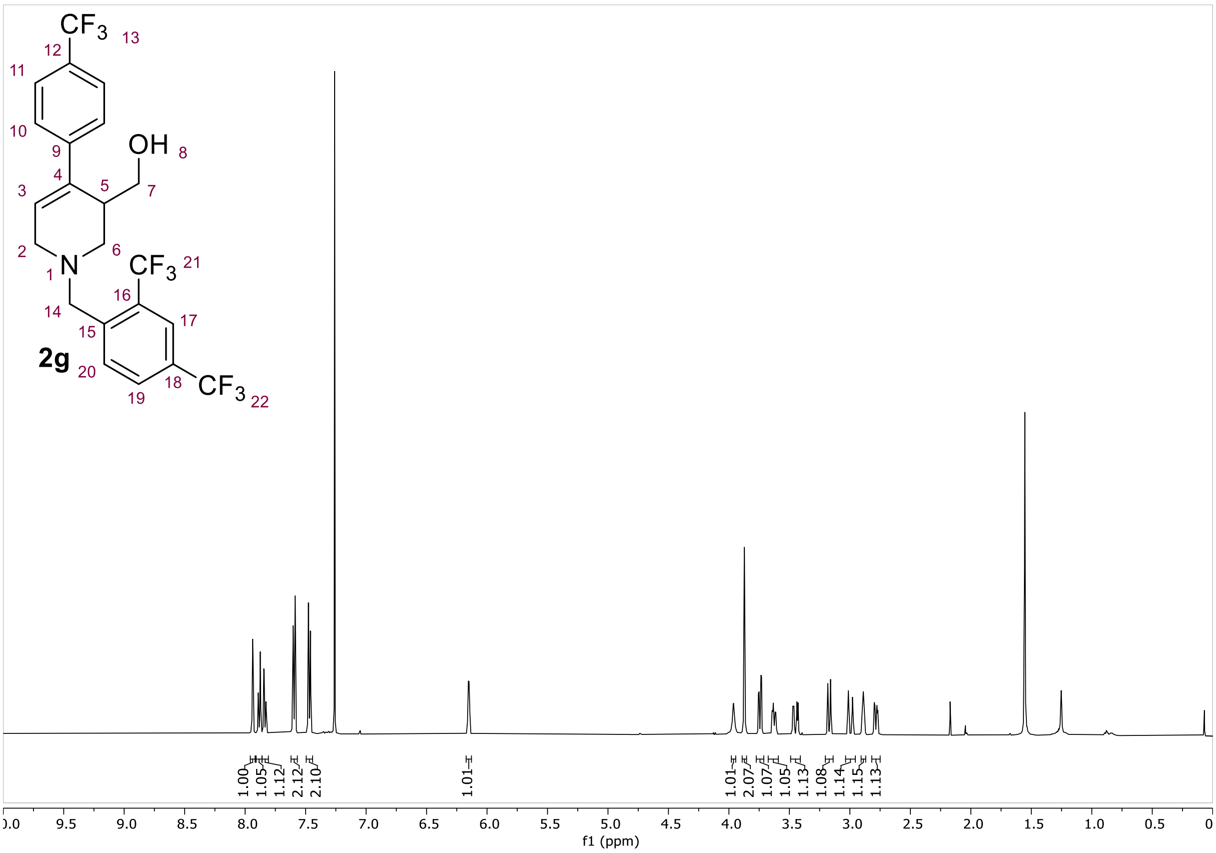
2g

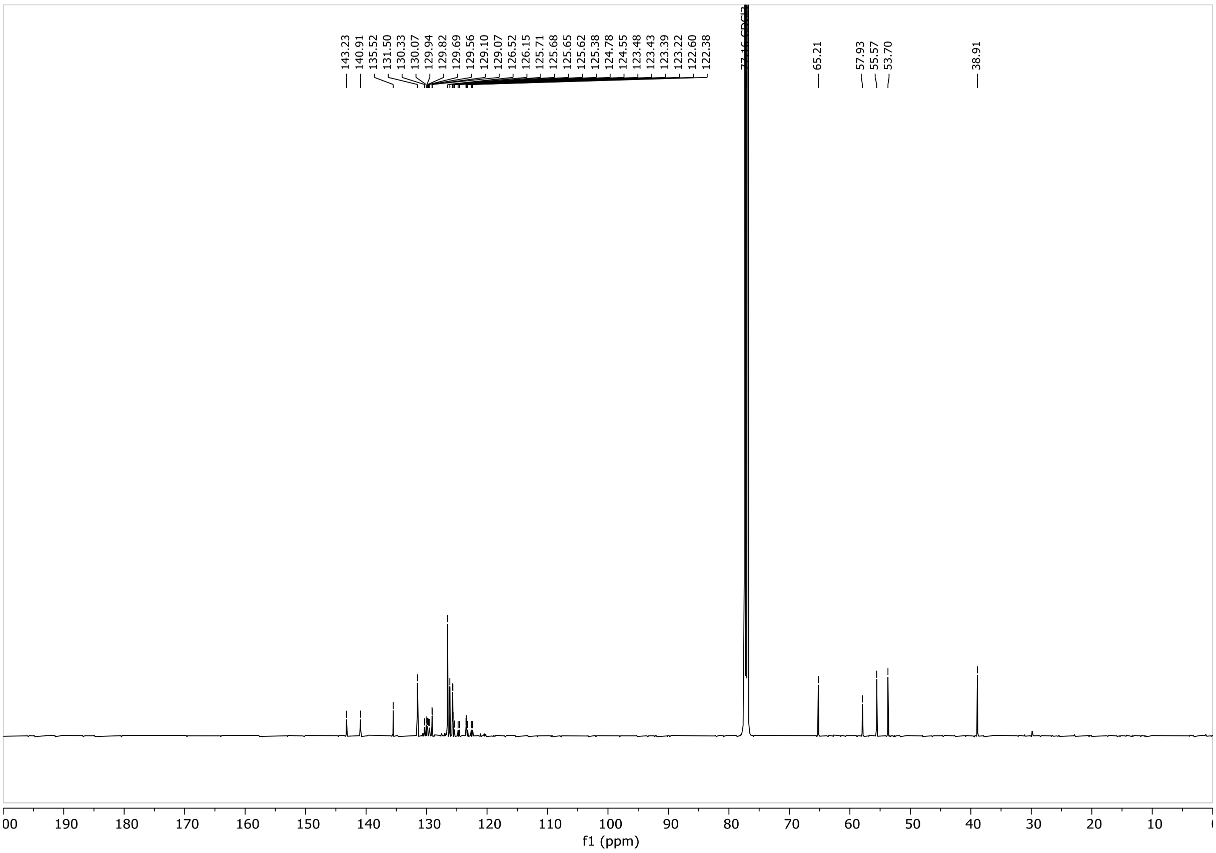

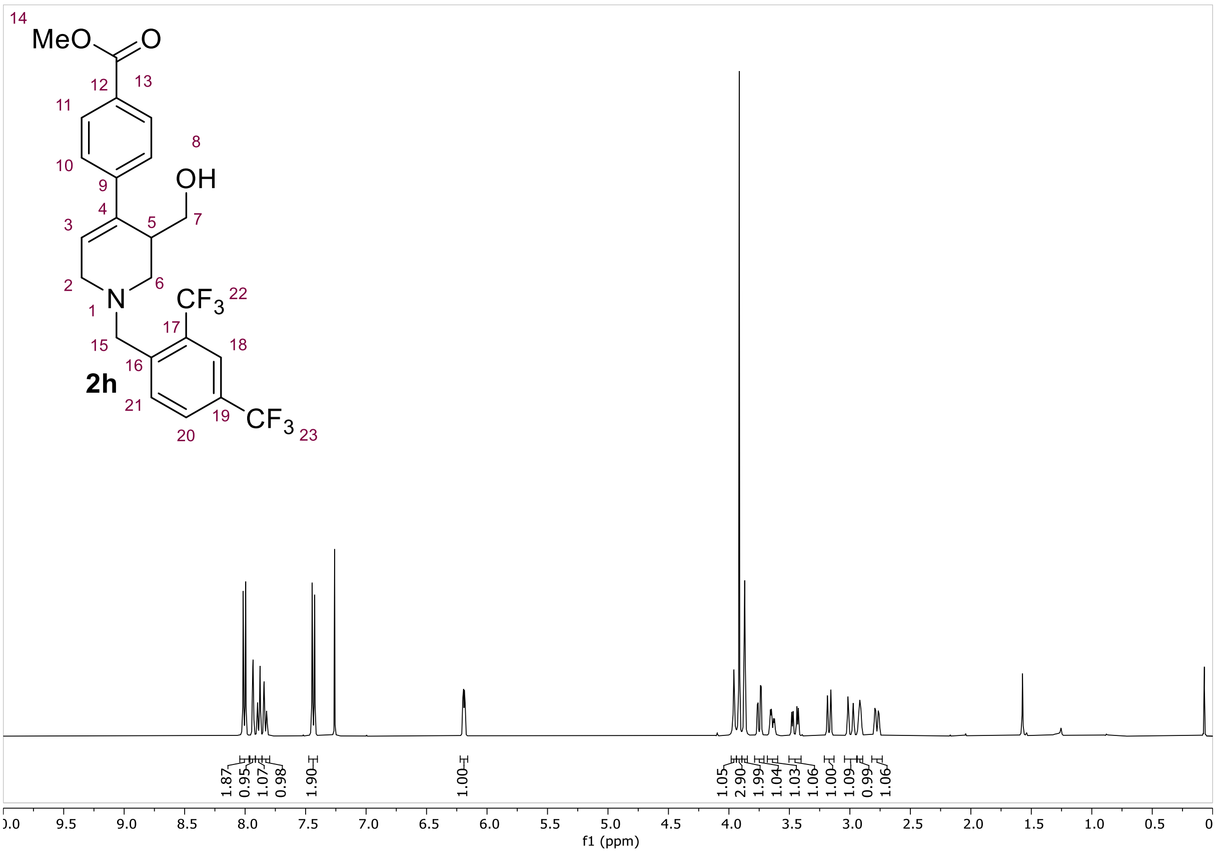
2h
MeO
O
OH
N
CF3
CF3
1.87
0.95
1.07
0.98
1.90
1.00
1.05
2.90
1.99
1.04
1.03
1.06
1.00
1.09
0.99
1.06
f1 (ppm)

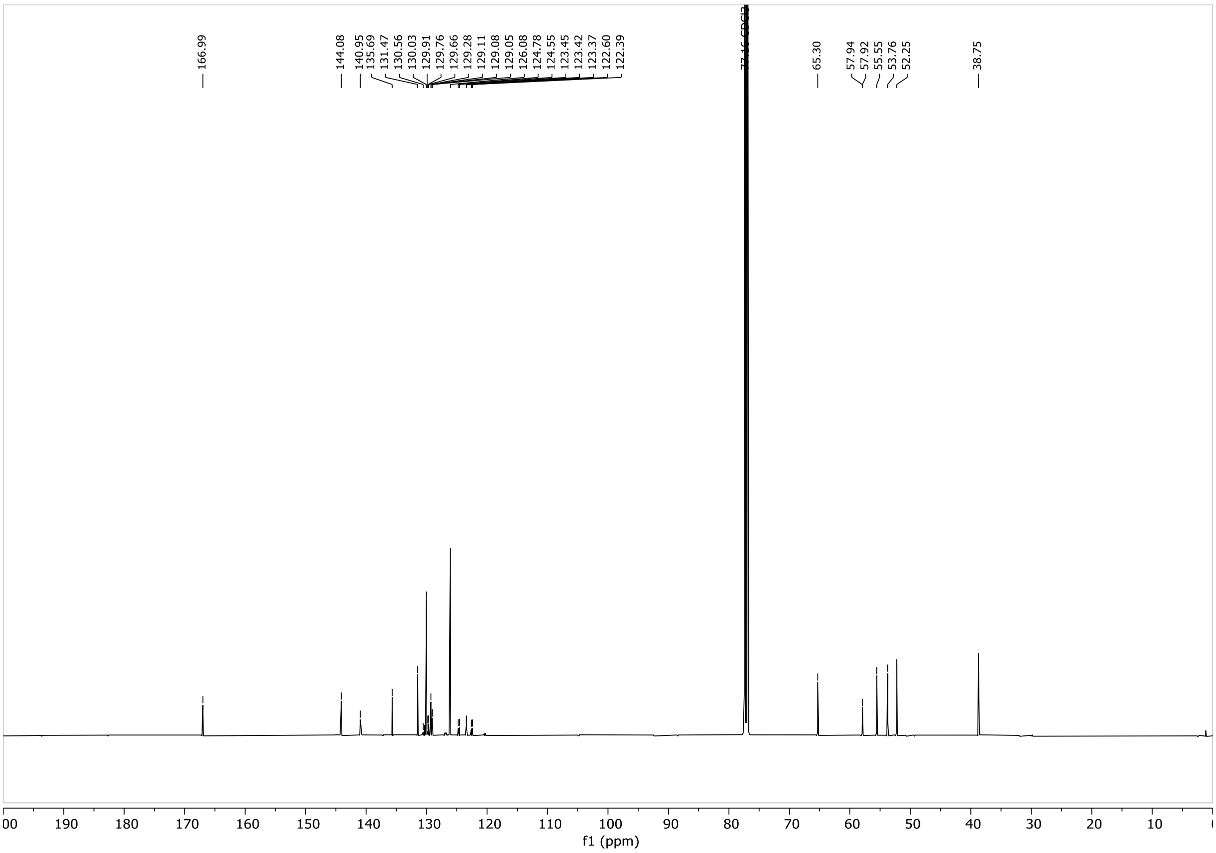
166.99
144.08
140.95
135.69
131.47
130.56
130.03
129.91
129.76
129.66
129.28
129.11
129.08
129.05
126.08
124.78
124.55
123.45
123.42
123.37
122.60
122.39
65.30
57.94
57.92
55.55
53.76
52.25
38.75
f1 (ppm)

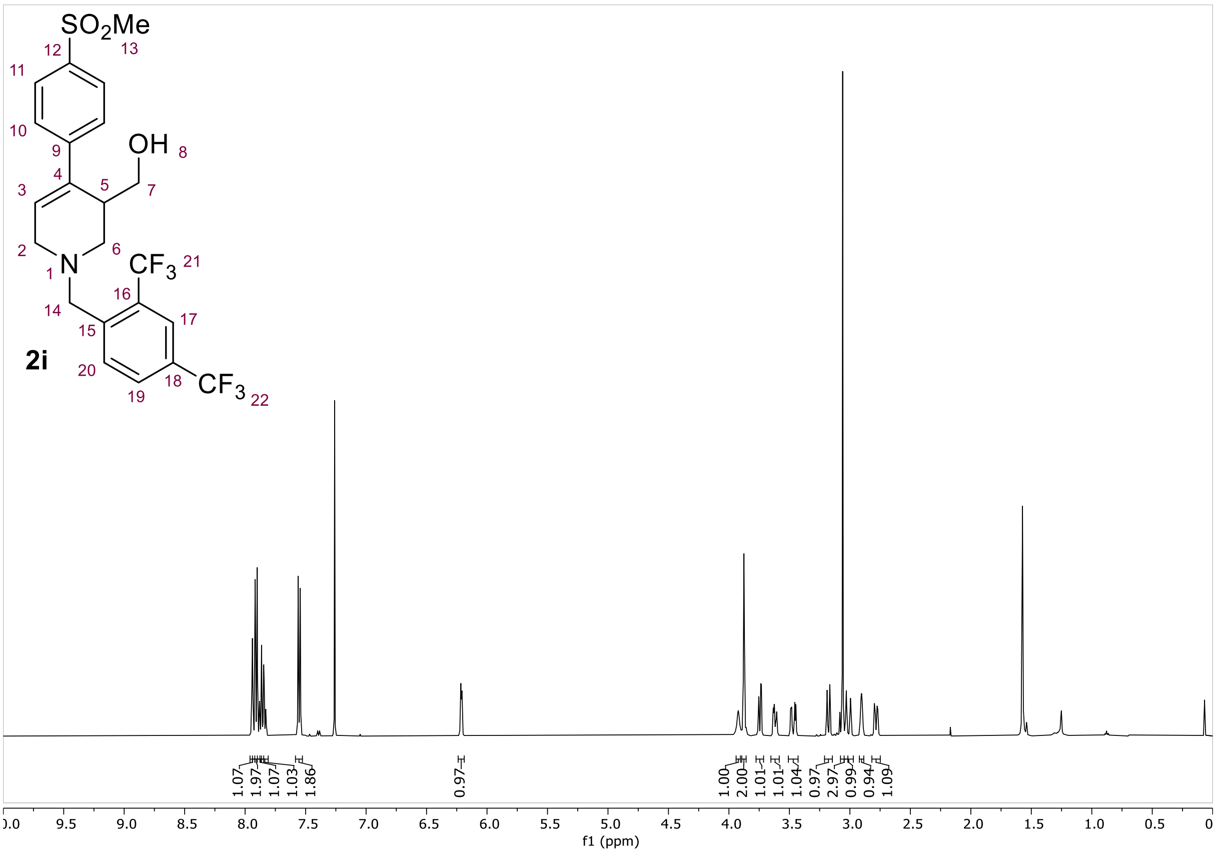
SO2Me
OH
N
CF3
2i
1.07
1.97
1.07
1.03
1.86
0.97
1.00
2.00
1.01
1.01
1.04
0.97
2.97
0.99
0.94
1.09
f1 (ppm)

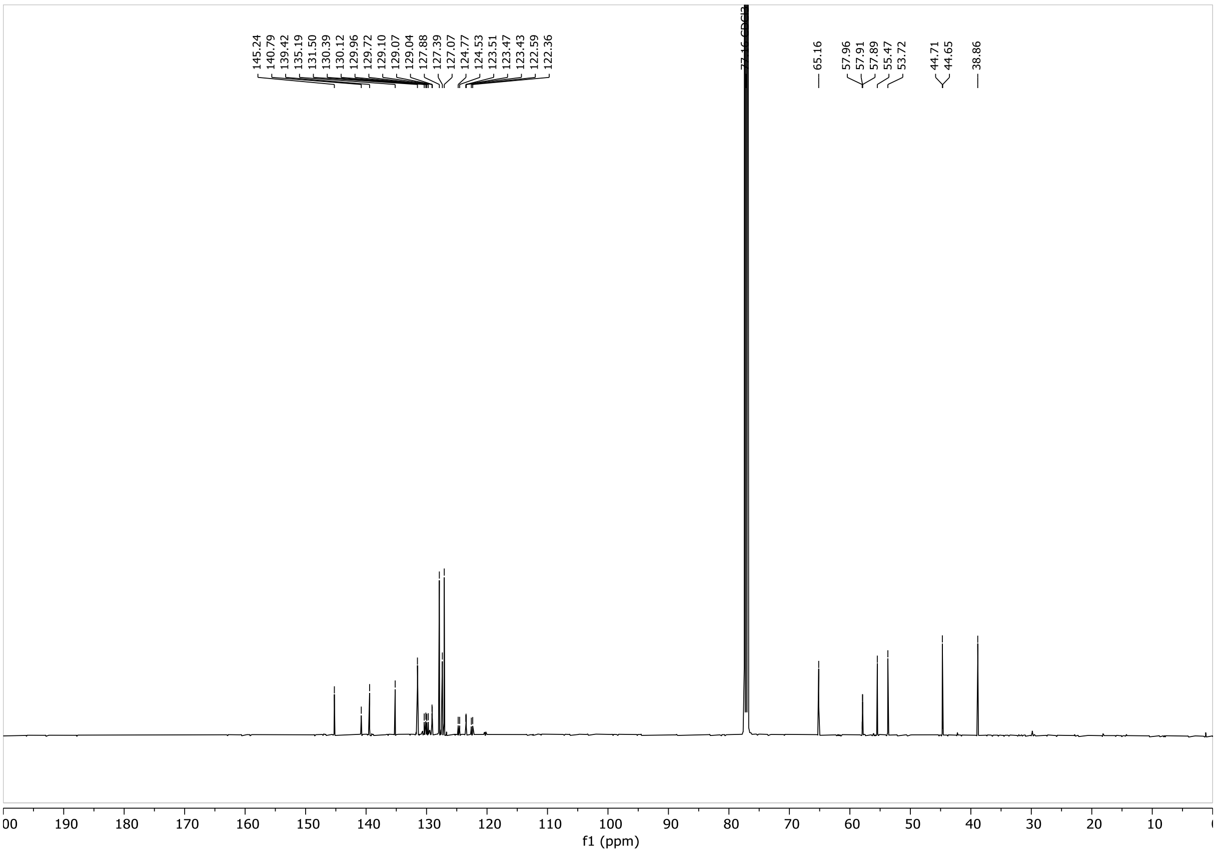
145.24
140.79
139.42
135.19
131.50
130.39
130.12
129.96
129.72
129.10
129.07
129.04
127.88
127.39
127.07
124.77
124.53
123.51
123.47
123.43
122.59
122.36
65.16
57.96
57.91
57.89
55.47
53.72
44.71
44.65
38.86
f1 (ppm)

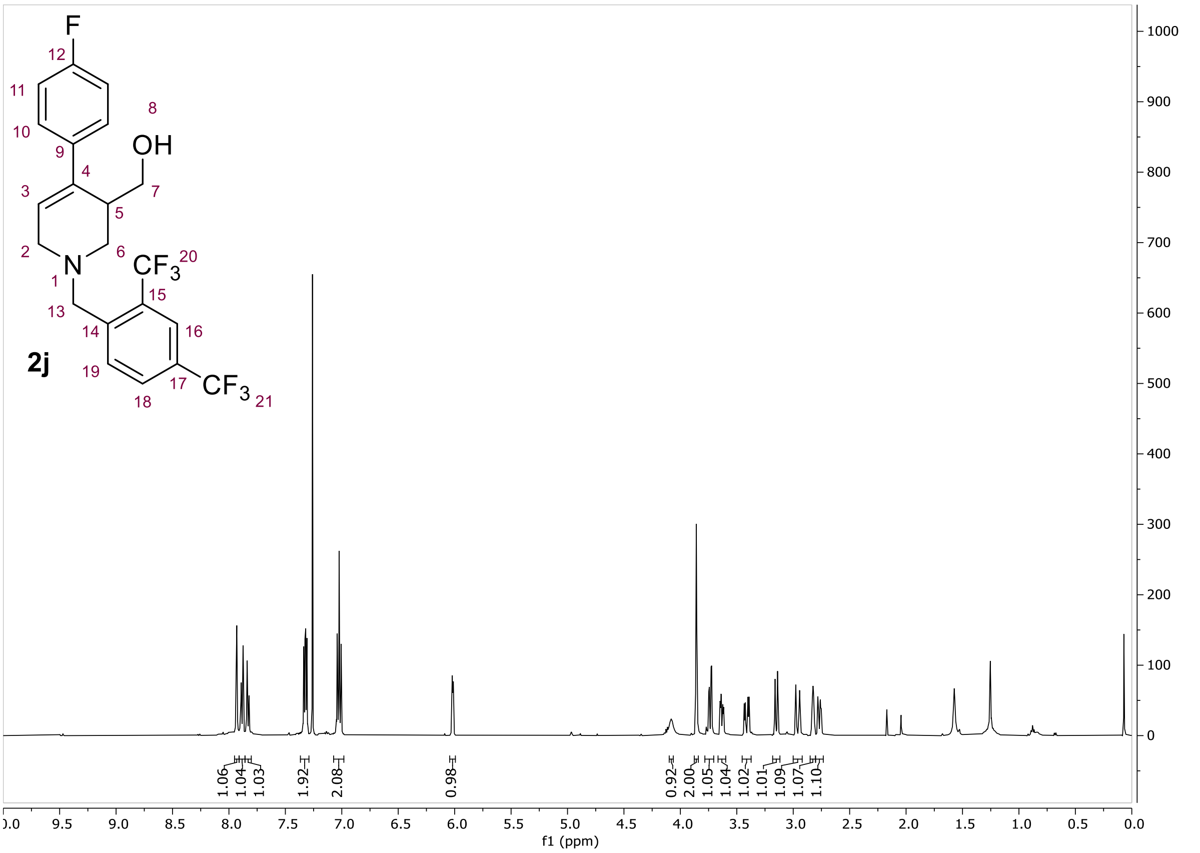

2j
F
OH
N
CF3
CF3
1.06
1.04
1.03
1.92
2.08
0.98
0.92
2.00
1.05
1.04
1.02
1.01
1.09
1.07
1.10
f1 (ppm)

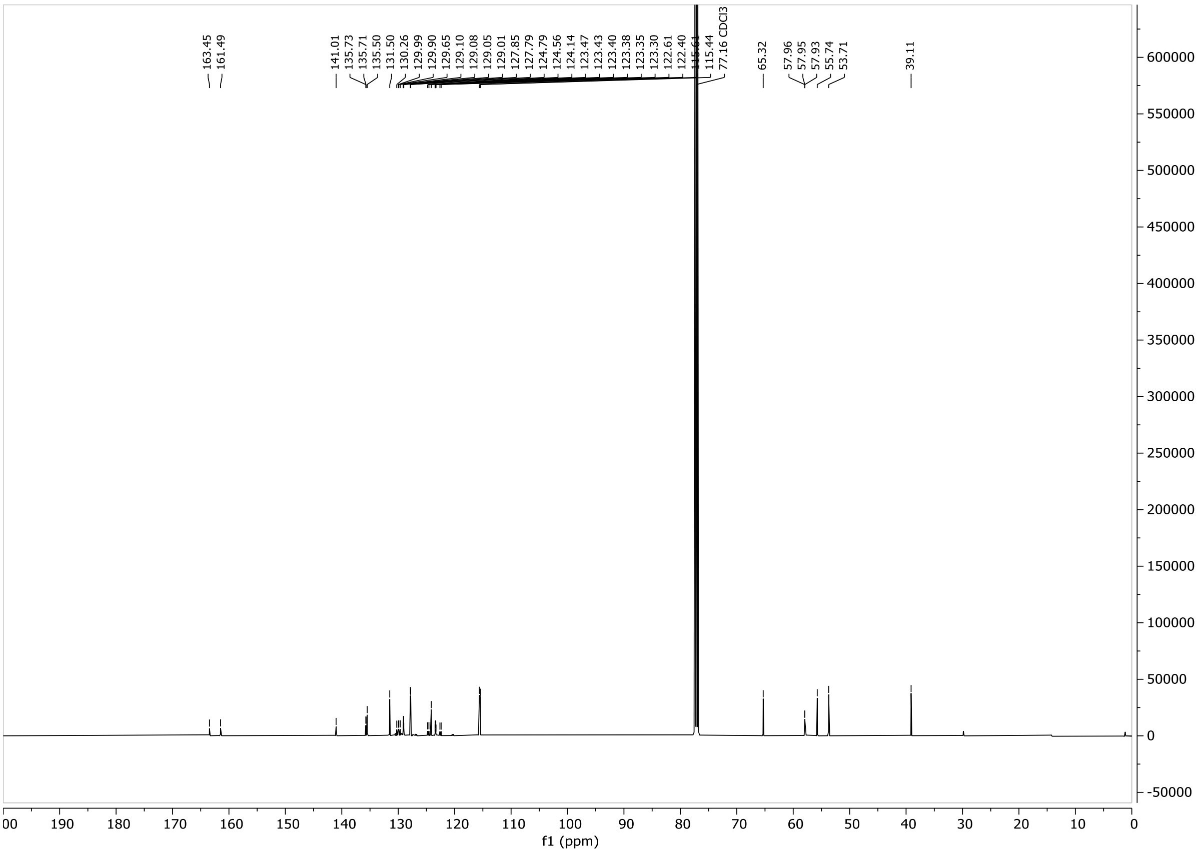

163.45
161.49
141.01
135.73
135.71
135.50
131.50
130.26
129.99
129.90
129.65
129.10
129.08
129.05
129.01
127.85
127.79
124.79
124.56
124.14
123.47
123.43
123.40
123.38
123.35
123.30
122.61
122.40
115.44
77.16 CDCl3
65.32
57.96
57.95
57.93
55.74
53.71
39.11
f1 (ppm)

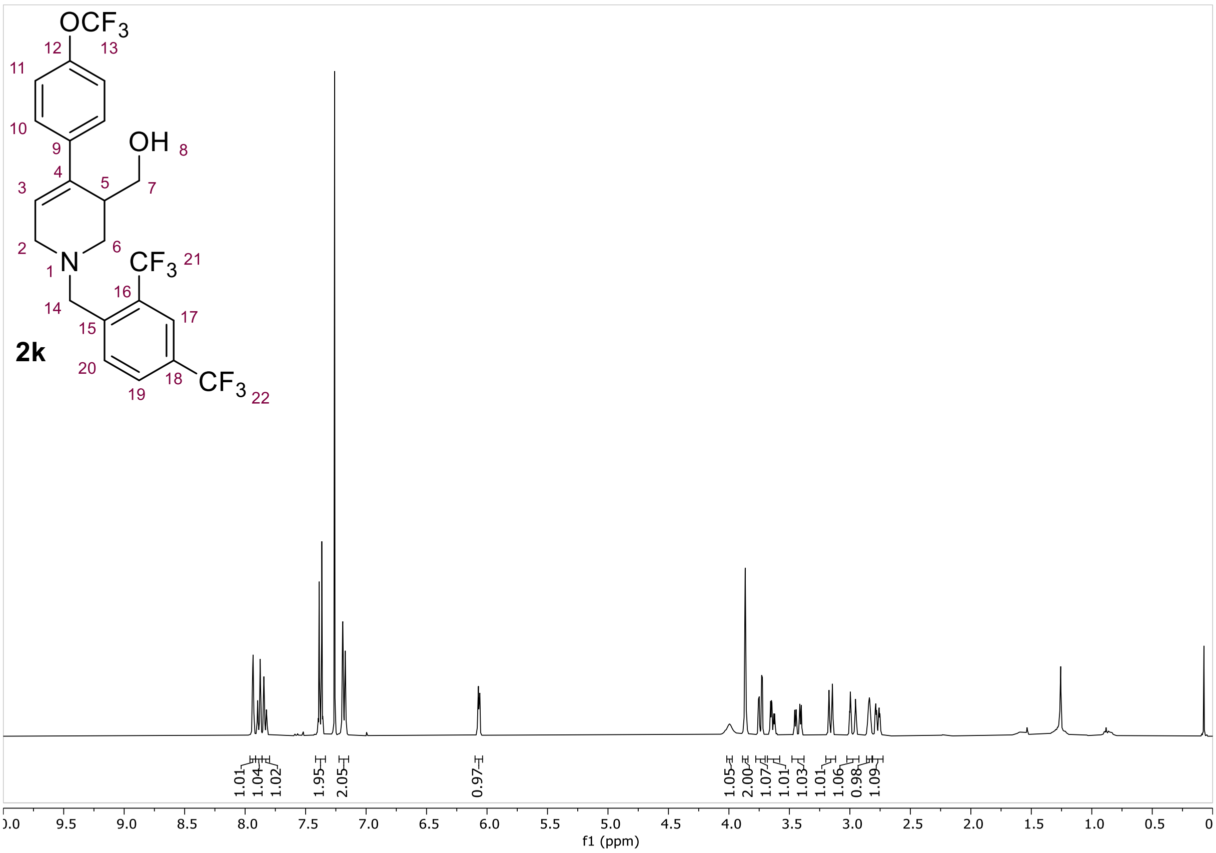
OCF3
2k
OH
N
CF3
CF3
1.01
1.04
1.02
1.95
2.05
0.97
1.05
2.00
1.07
1.01
1.03
1.01
1.06
0.98
1.09
f1 (ppm)

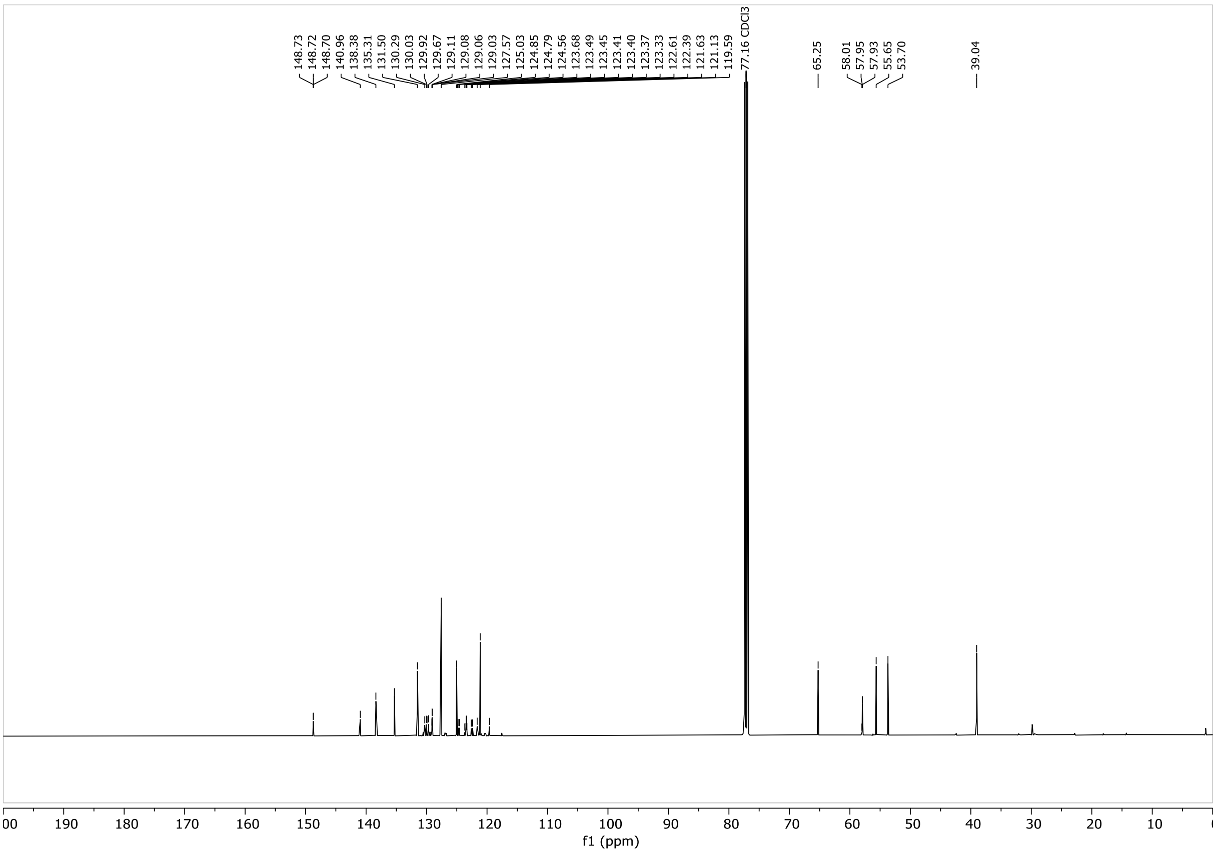
148.73
148.72
148.70
140.96
138.38
135.31
131.50
130.29
130.03
129.92
129.67
129.11
129.08
129.06
129.03
127.57
125.03
124.85
124.79
124.56
123.68
123.49
123.45
123.41
123.40
123.37
123.33
122.61
122.39
121.63
121.13
119.59
77.16 CDCl3
65.25
58.01
57.95
57.93
55.65
53.70
39.04
f1 (ppm)

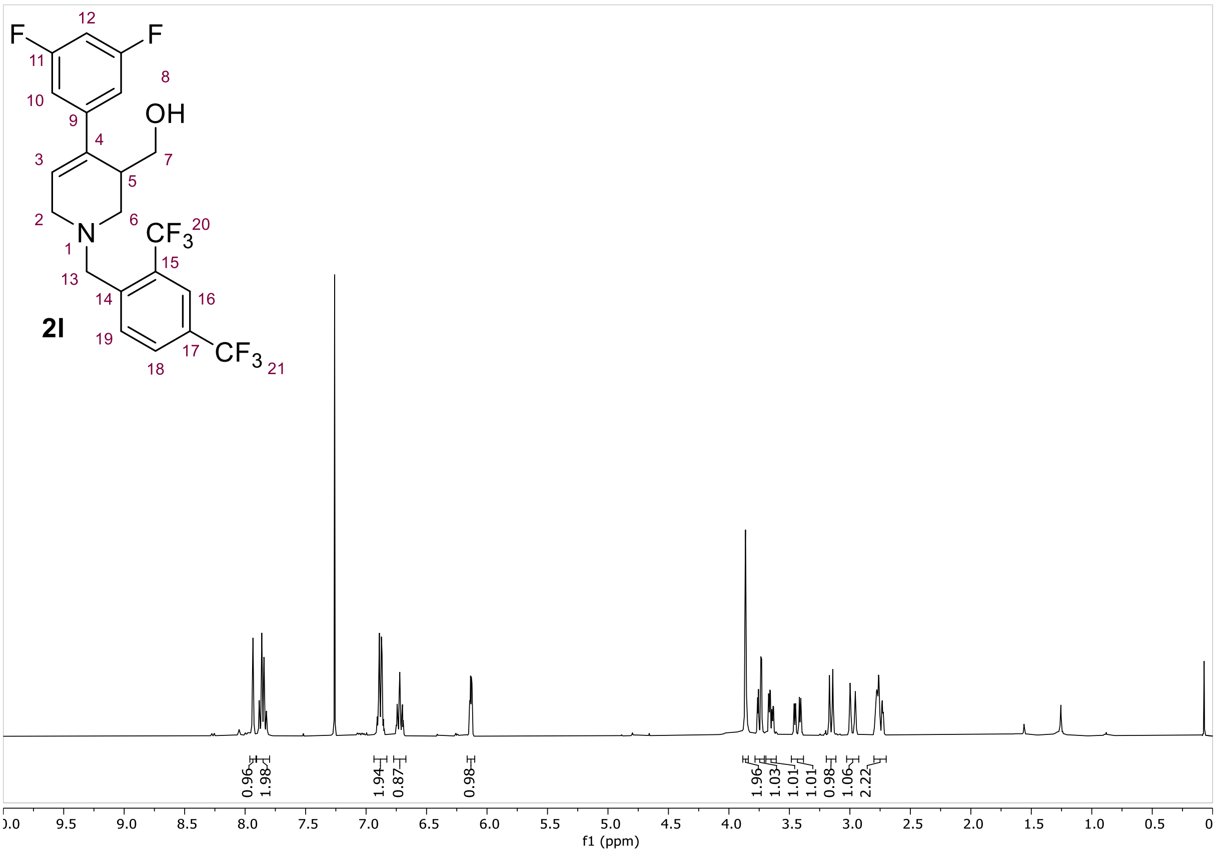

F
F
OH
N
CF3
CF3
2l
12
11
8
10
9
4
7
3
5
2
6
20
1
15
13
14
16
19
17
21
18
0.96
1.98
1.94
0.87
0.98
1.96
1.03
1.01
1.01
0.98
1.06
2.22
f1 (ppm)

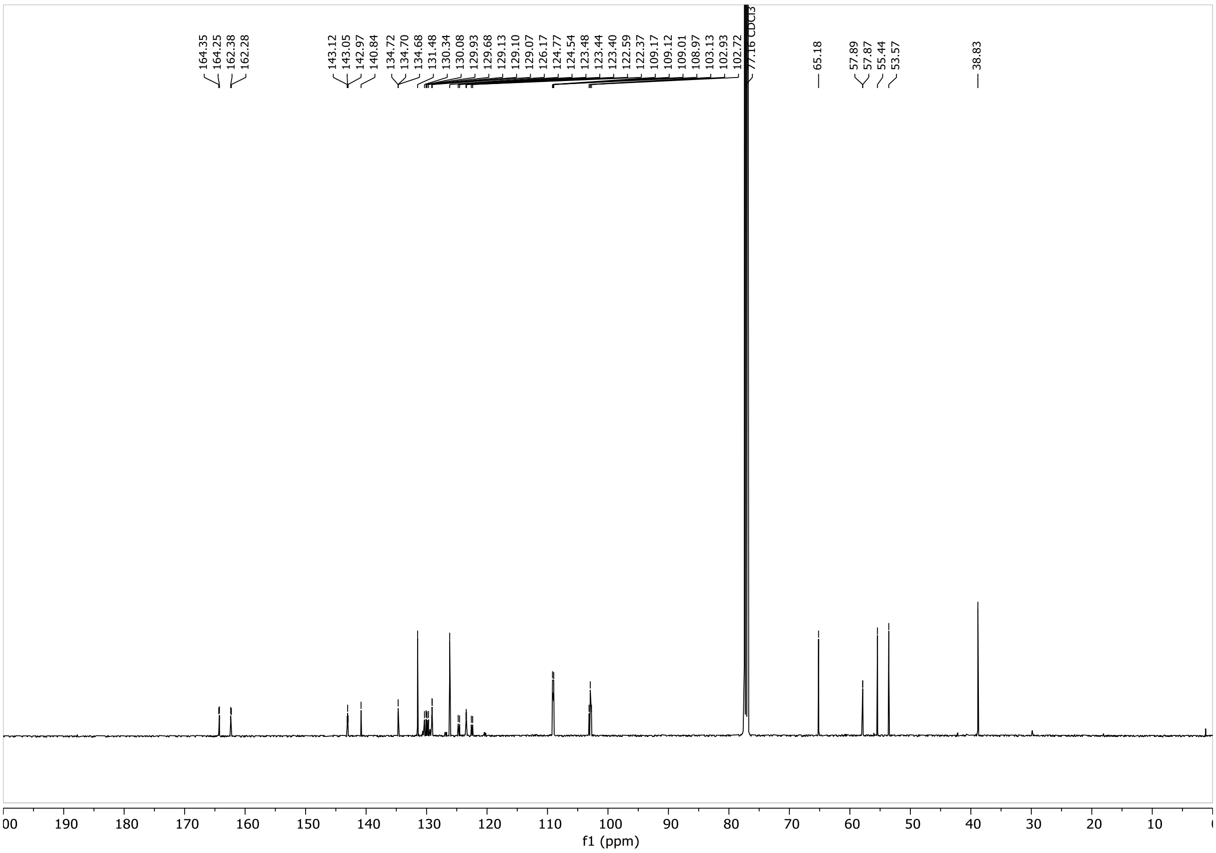

164.35
164.25
162.38
162.28
143.12
143.05
142.97
140.84
134.72
134.70
134.68
131.48
130.34
130.08
129.93
129.68
129.13
129.10
129.07
126.17
124.77
124.54
123.48
123.44
123.40
122.59
122.37
109.17
109.12
109.01
108.97
103.13
102.93
102.72
77.16 CDCl3
65.18
57.89
57.87
55.44
53.57
38.83
f1 (ppm)

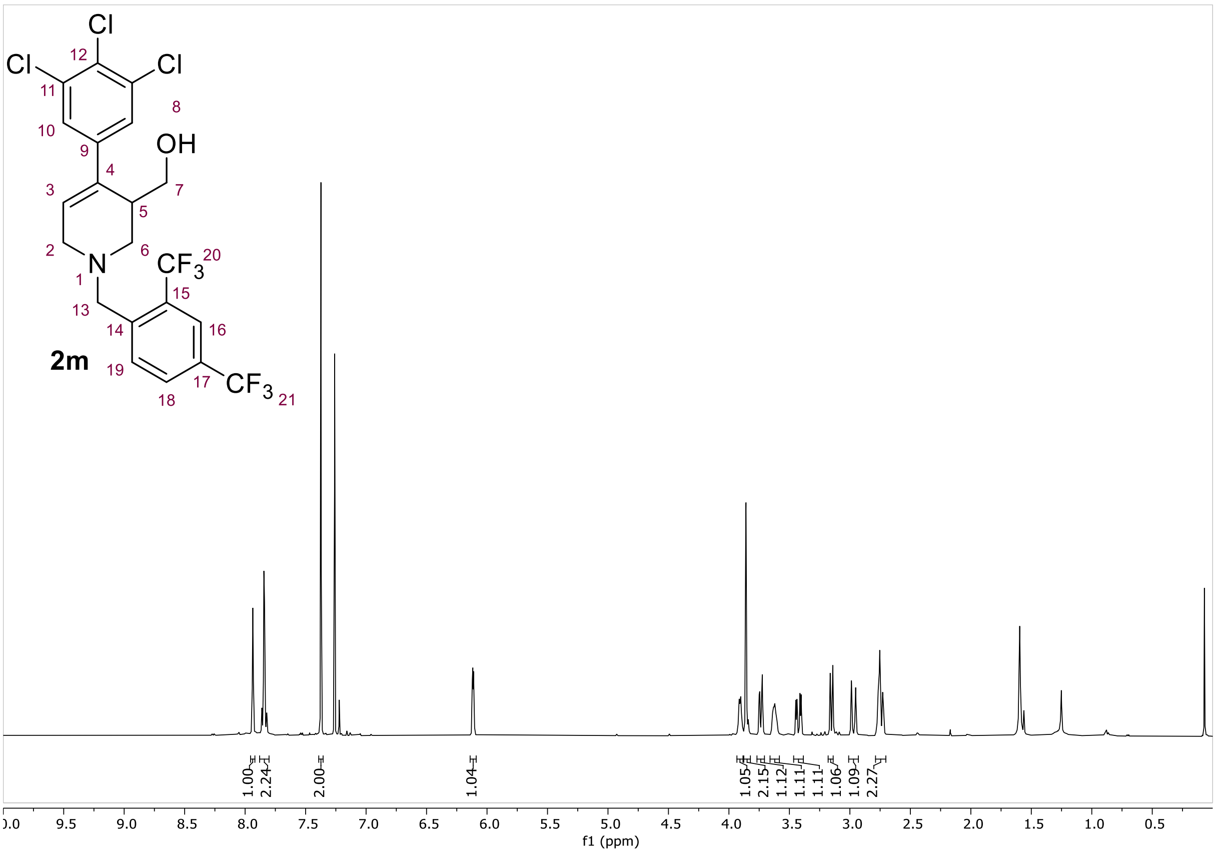

2m
f1 (ppm)

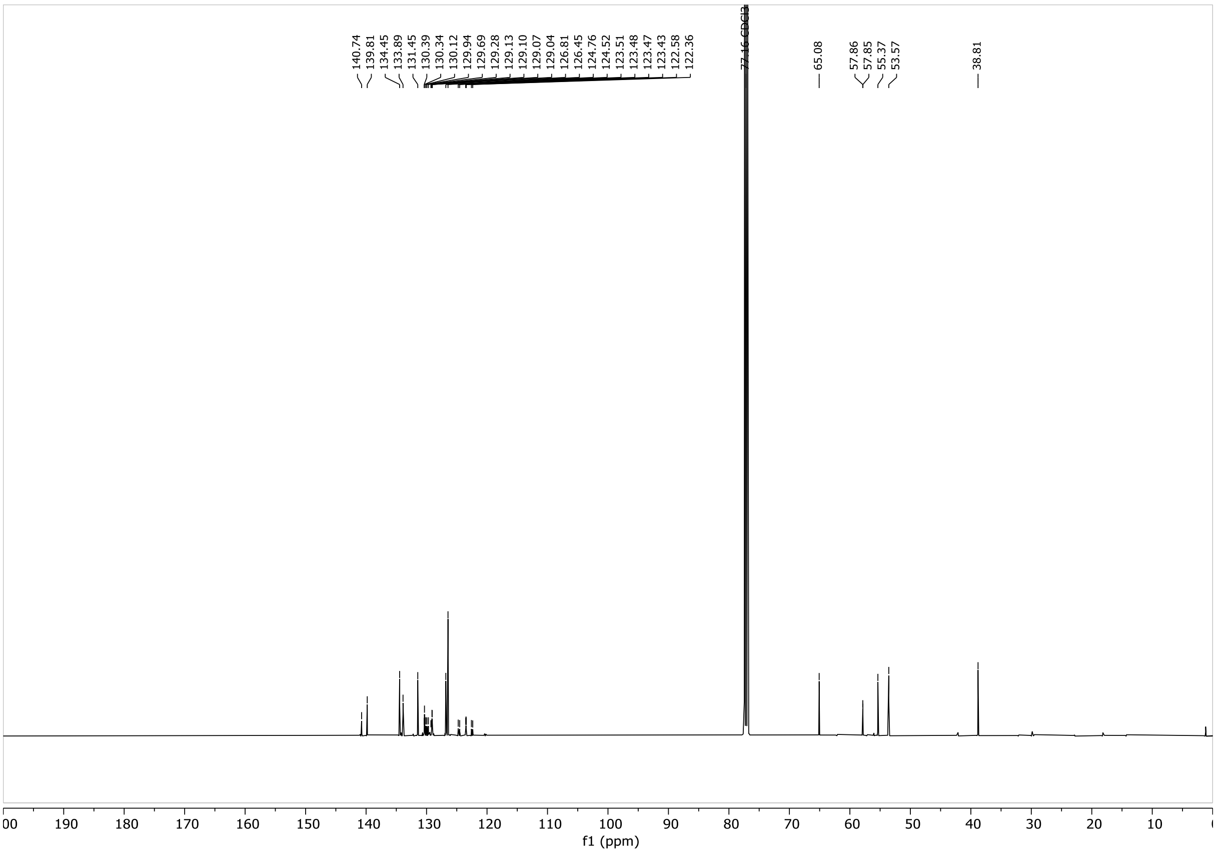

77.16 CDCl3
65.08
57.86
57.85
55.37
53.57
38.81
f1 (ppm)

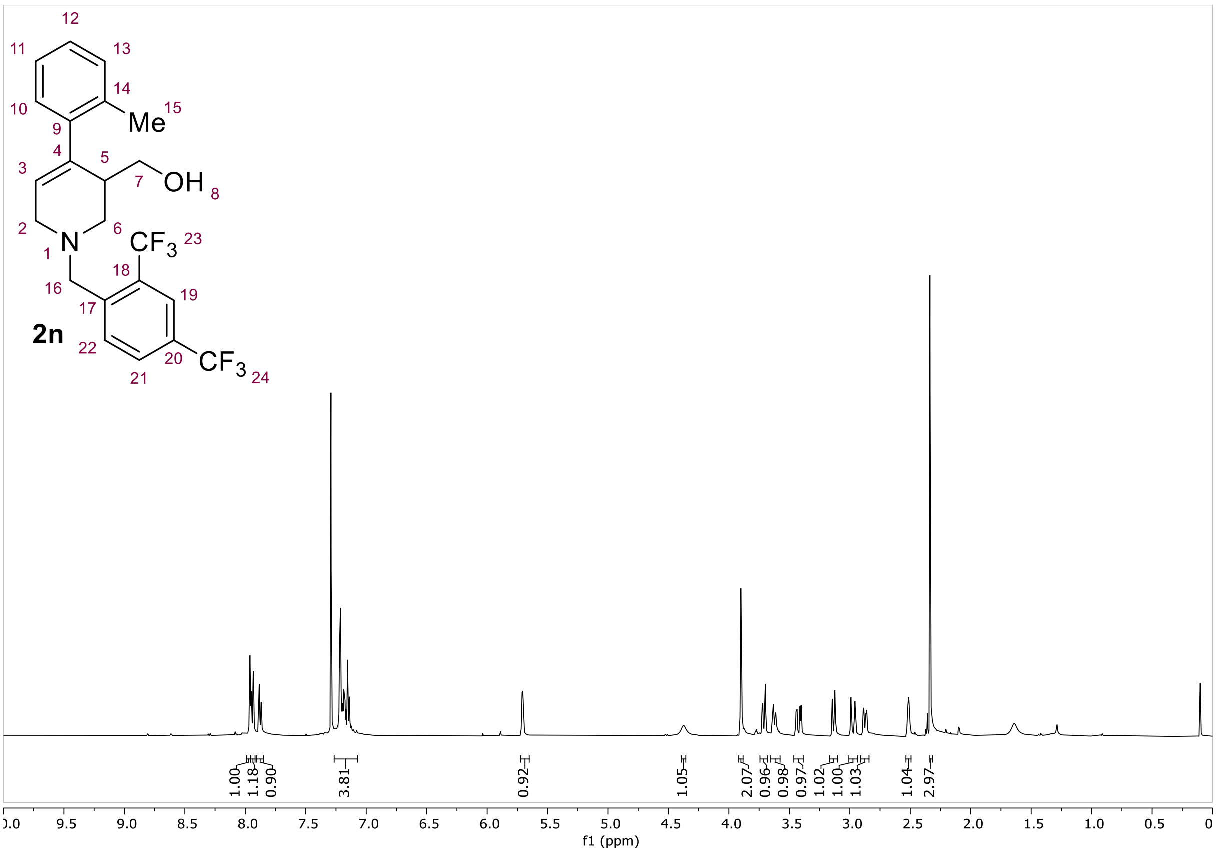
2n
1.00
1.18
0.90
3.81
0.92
1.05
2.07
0.96
0.98
0.97
1.02
1.00
1.03
1.04
2.97
f1 (ppm)

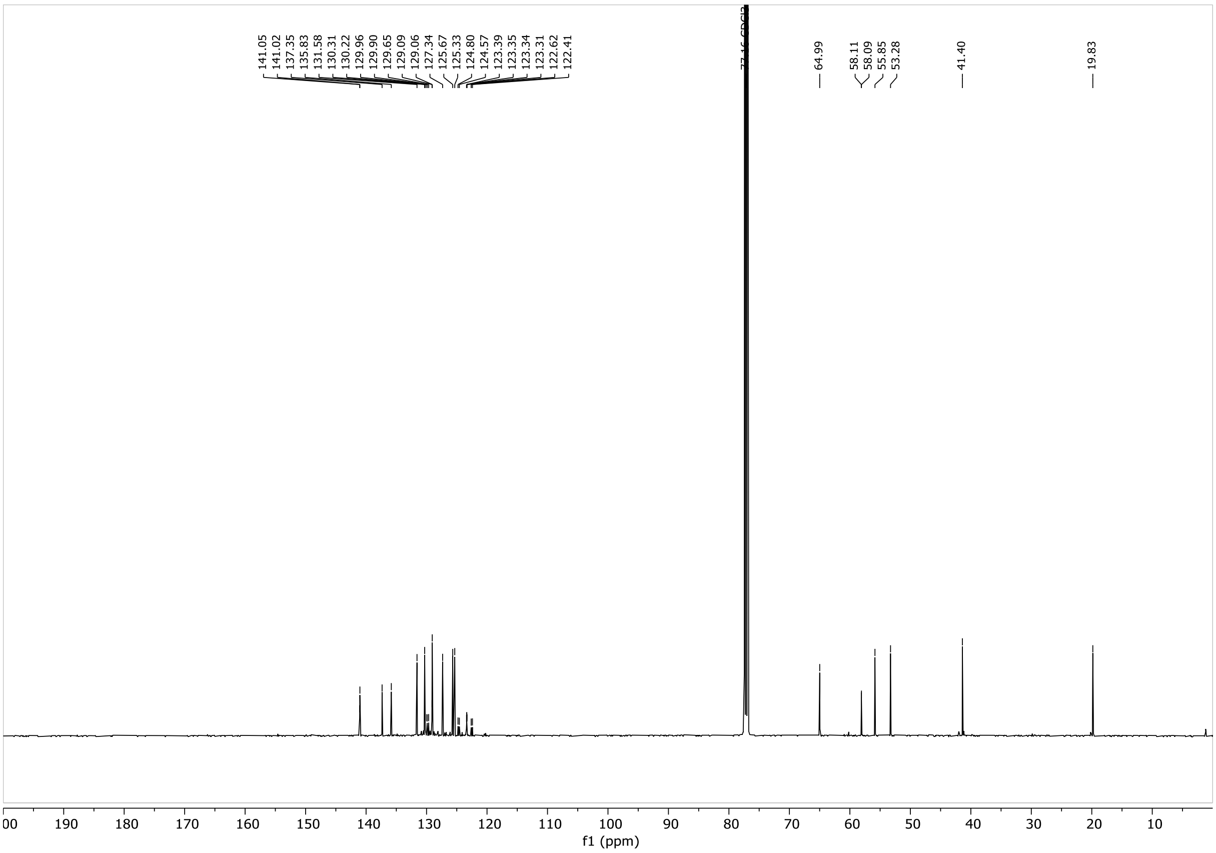
141.05
141.02
137.35
135.83
131.58
130.31
130.22
129.96
129.90
129.65
129.09
129.06
127.34
125.67
125.33
124.80
124.57
123.39
123.35
123.34
123.31
122.62
122.41
64.99
58.11
58.09
55.85
53.28
41.40
19.83
f1 (ppm)

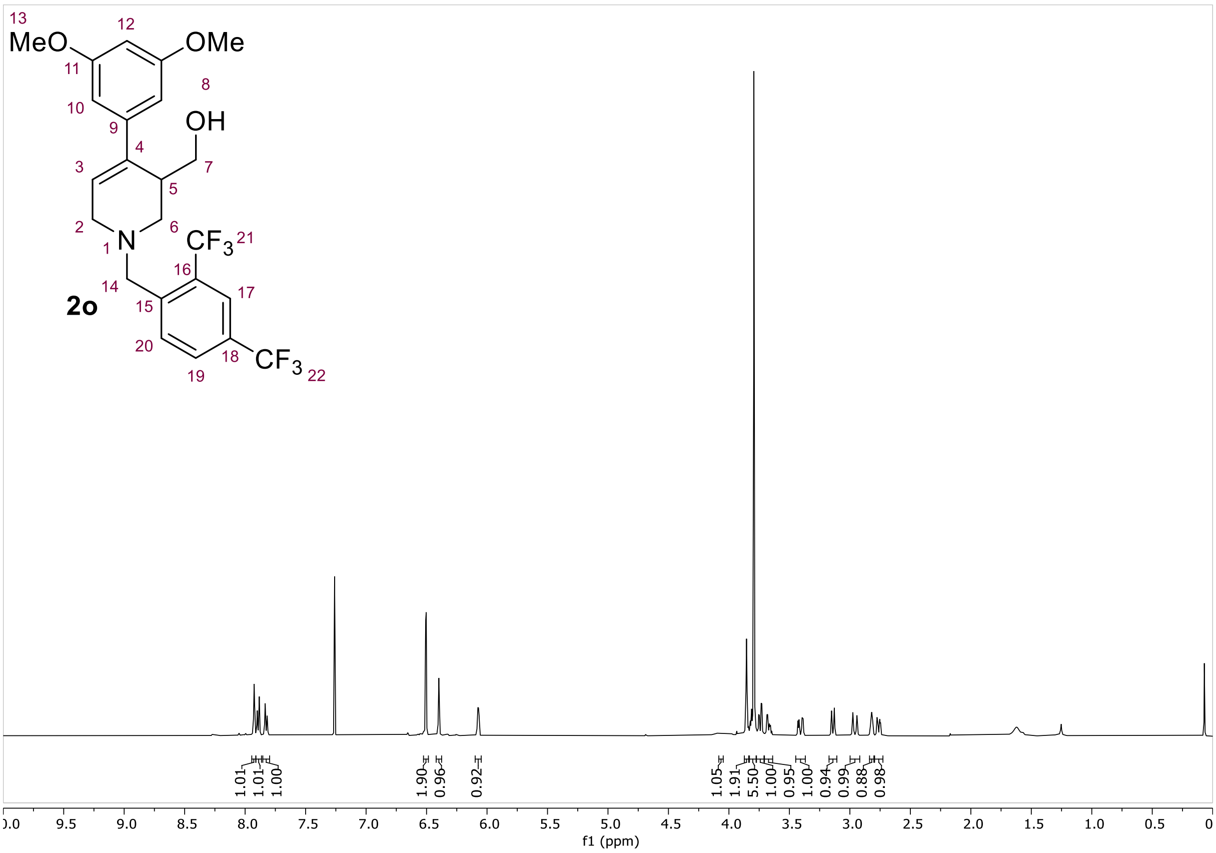
MeO
OMe
OH
N
CF3
CF3
2o
1.01
1.01
1.00
1.90
0.96
0.92
1.05
1.91
5.50
1.00
0.95
1.00
0.94
0.99
0.88
0.98
f1 (ppm)

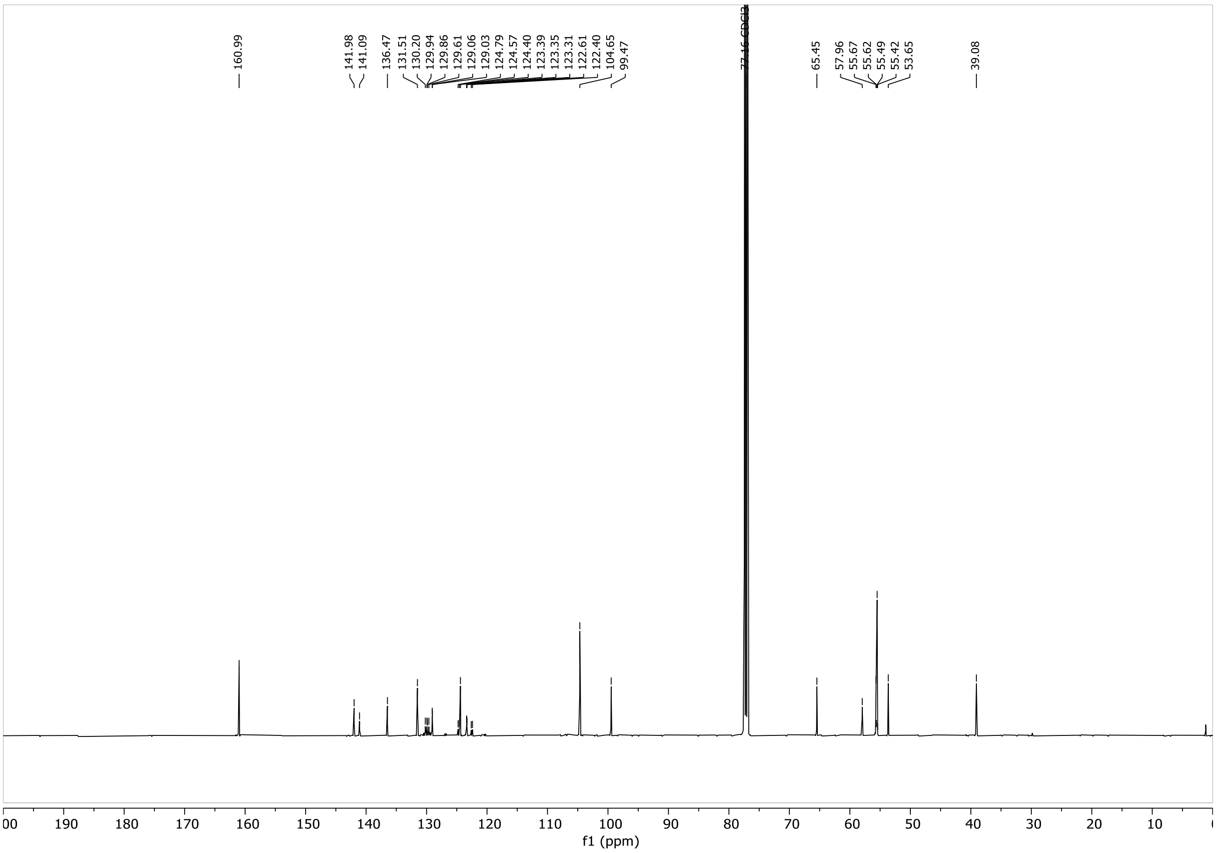
160.99
141.98
141.09
136.47
131.51
130.20
129.94
129.86
129.61
129.06
129.03
124.79
124.57
124.40
123.39
123.35
123.31
122.61
122.40
104.65
99.47
65.45
57.96
55.67
55.62
55.49
55.42
53.65
39.08
f1 (ppm)

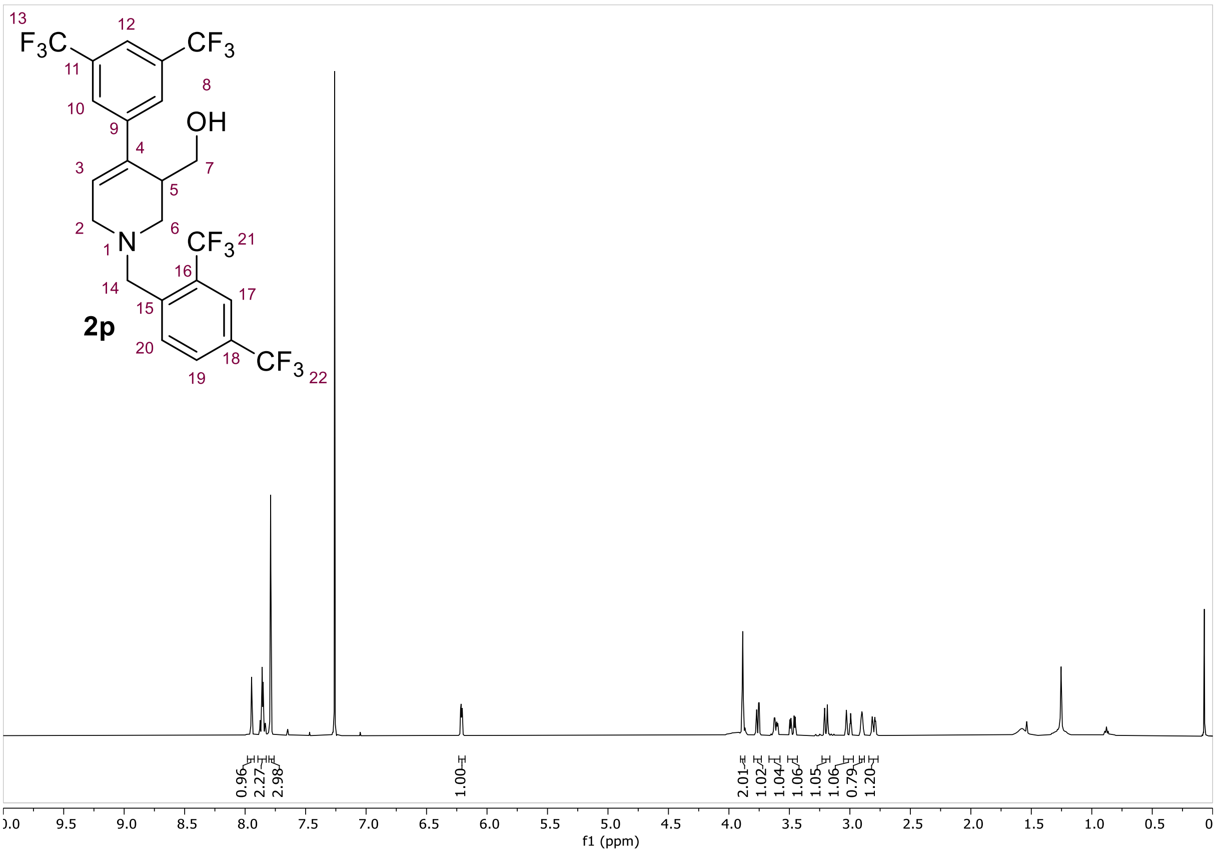

2p
OH
N
F_3C
CF_3
0.96
2.27
2.98
1.00
2.01
1.02
1.04
1.06
1.05
1.06
0.79
1.20
f1 (ppm)

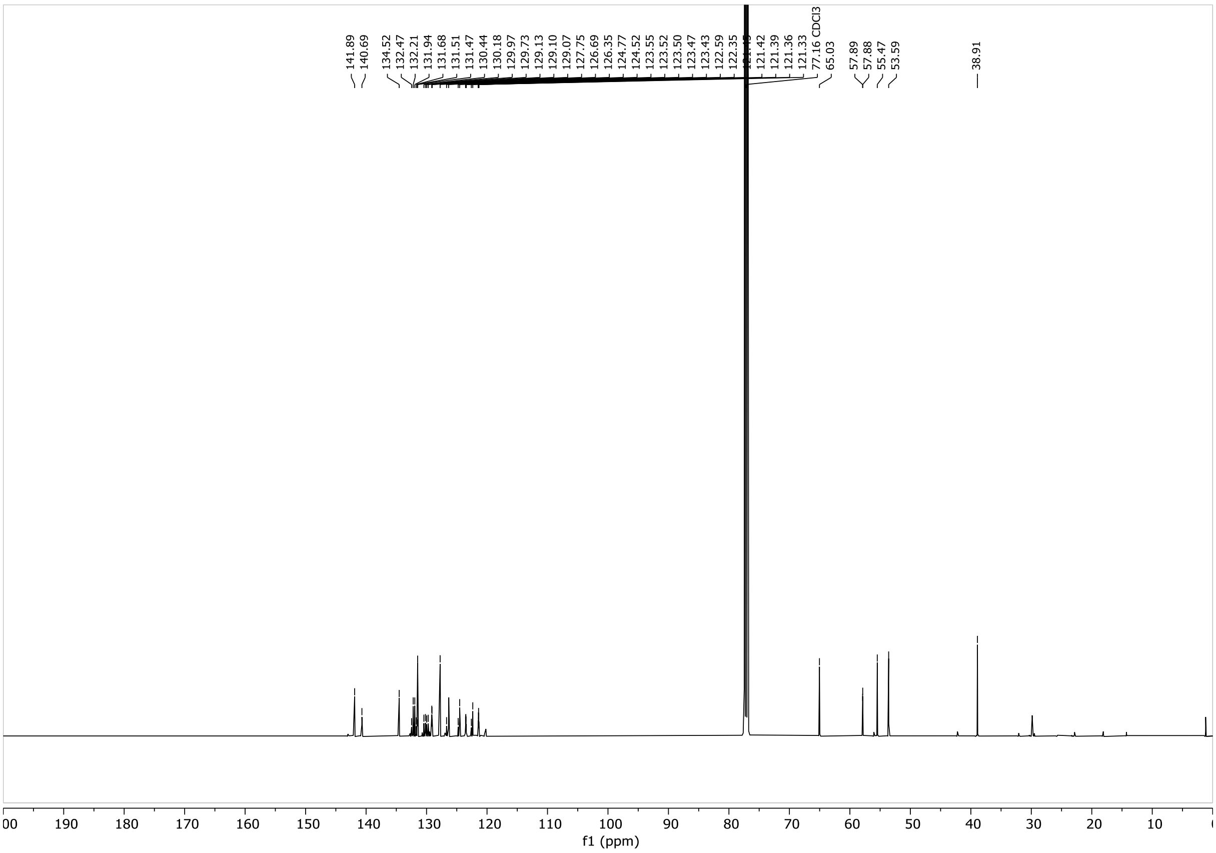

141.89
140.69
134.52
132.47
132.21
131.94
131.68
131.51
131.47
130.44
130.18
129.97
129.73
129.13
129.10
129.07
127.75
126.69
126.35
124.77
124.52
123.55
123.52
123.50
123.47
123.43
122.59
122.35
121.42
121.39
121.36
121.33
77.16 CDCl3
65.03
57.89
57.88
55.47
53.59
38.91
f1 (ppm)

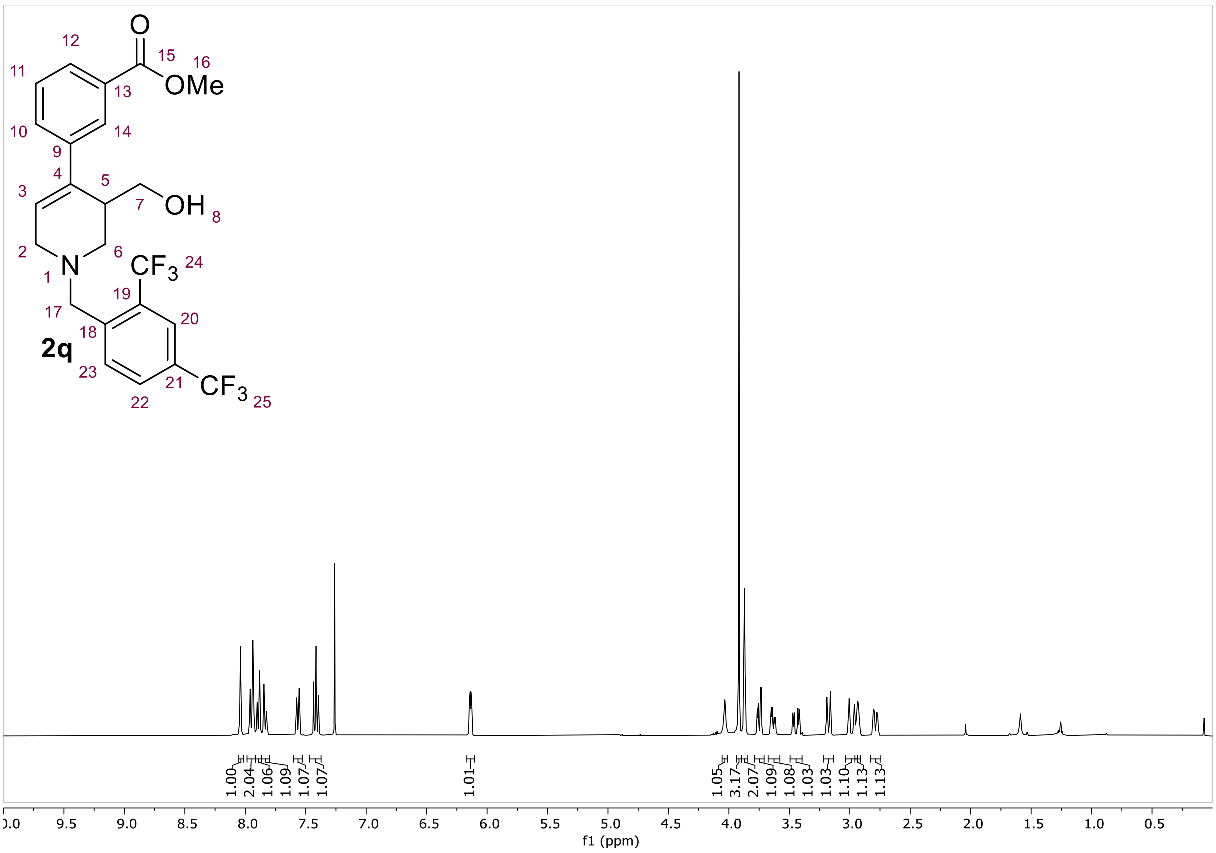
2q
O
OMe
OH
N
CF3
CF3
1.00
2.04
1.06
1.09
1.07
1.07
1.01
1.05
3.17
2.07
1.09
1.08
1.03
1.03
1.10
1.13
1.13
f1 (ppm)

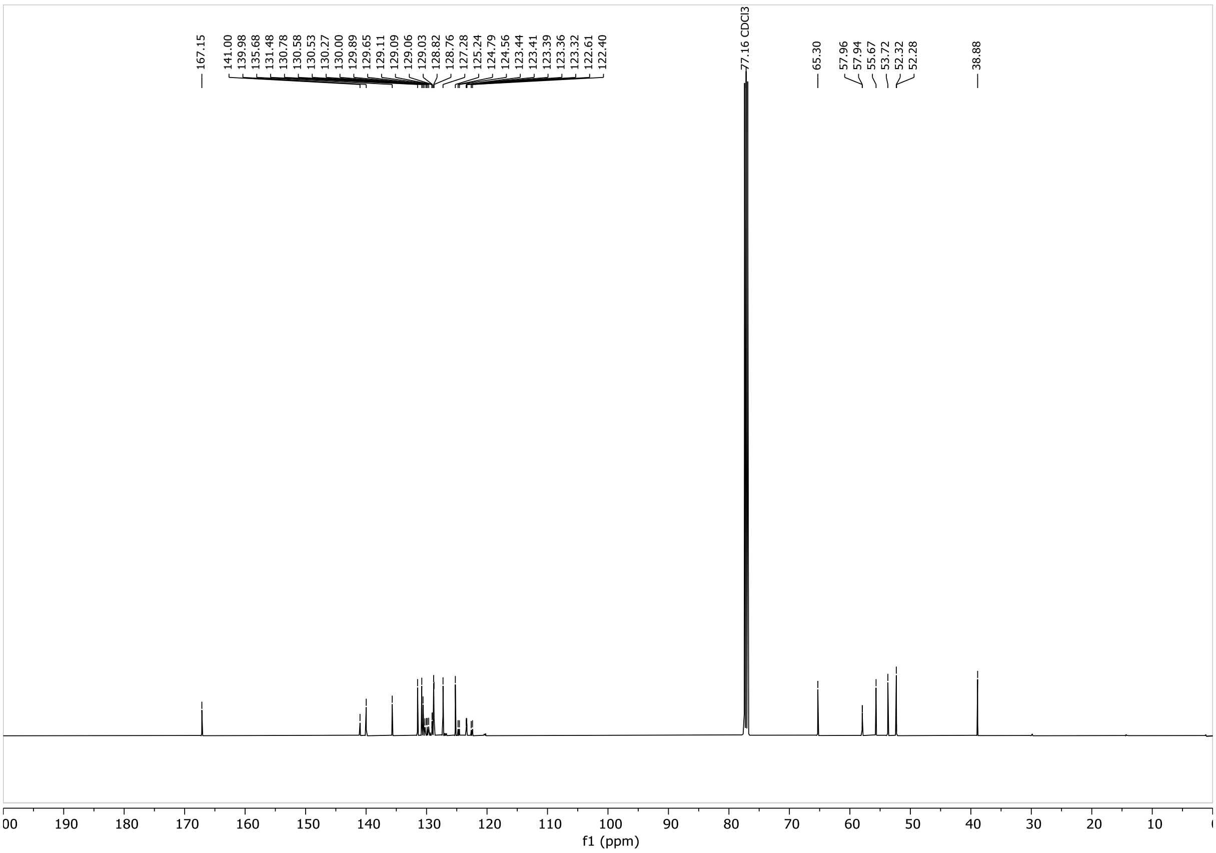
167.15
141.00
139.98
135.68
131.48
130.78
130.58
130.53
130.27
130.00
129.89
129.65
129.11
129.09
129.06
129.03
128.82
128.76
127.28
125.24
124.79
124.56
123.44
123.41
123.39
123.36
123.32
122.61
122.40
77.16 CDCl3
65.30
57.96
57.94
55.67
53.72
52.32
52.28
38.88
f1 (ppm)

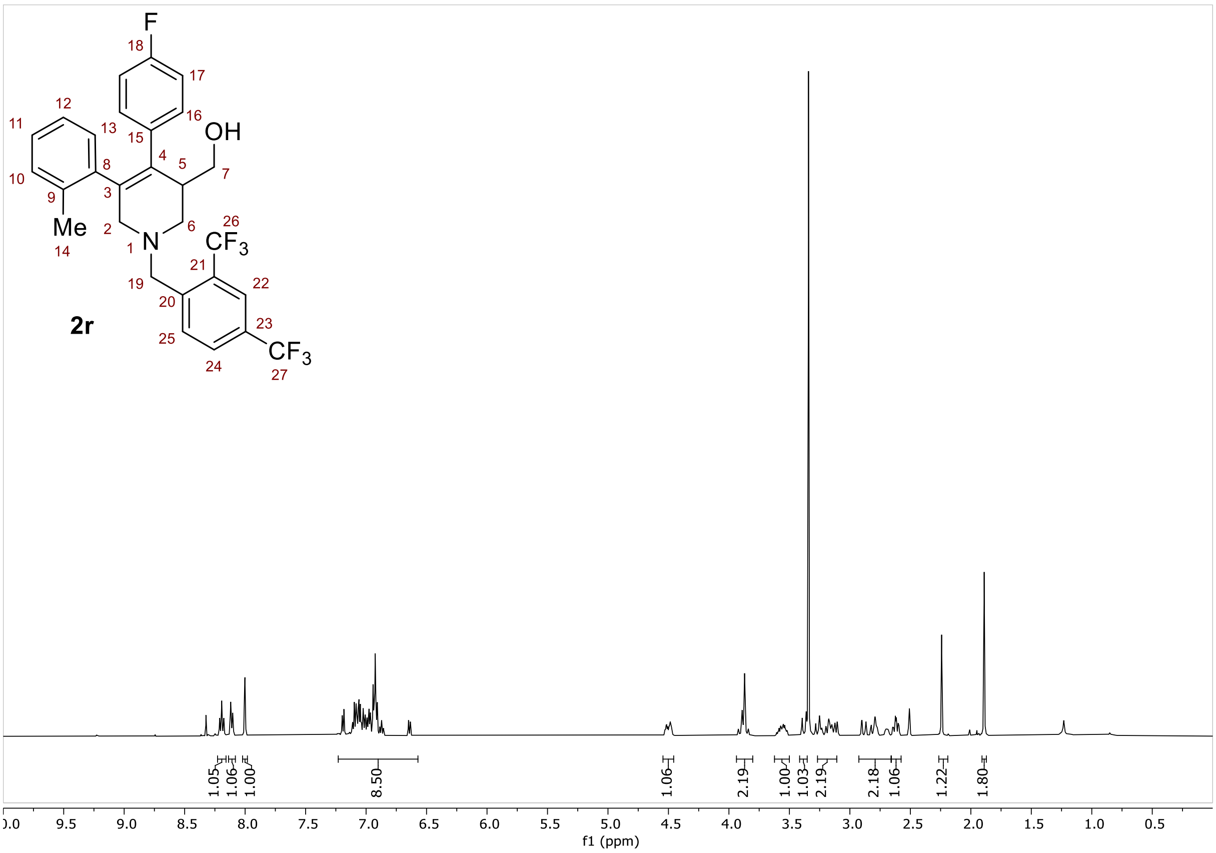

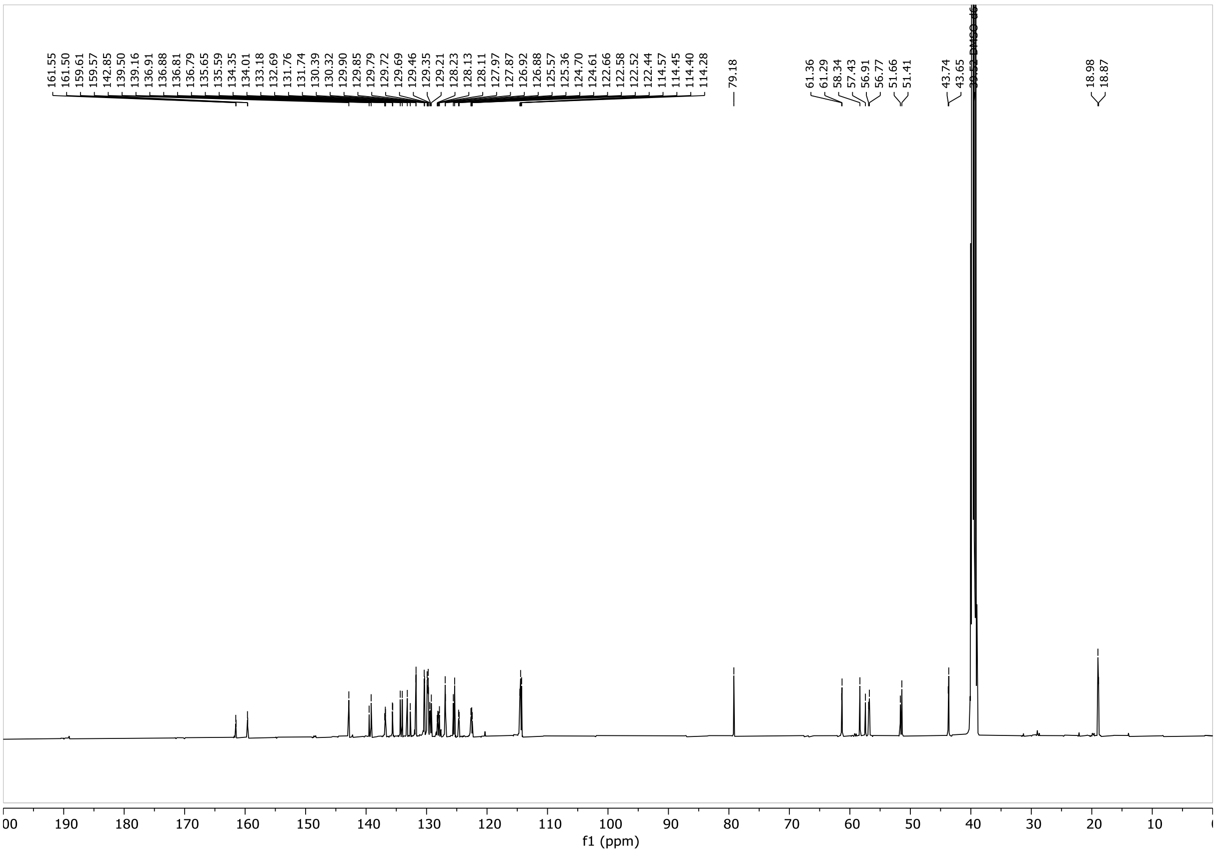

This compound showed rotameric behaviour at RT that could not be resolved using VT NMR.

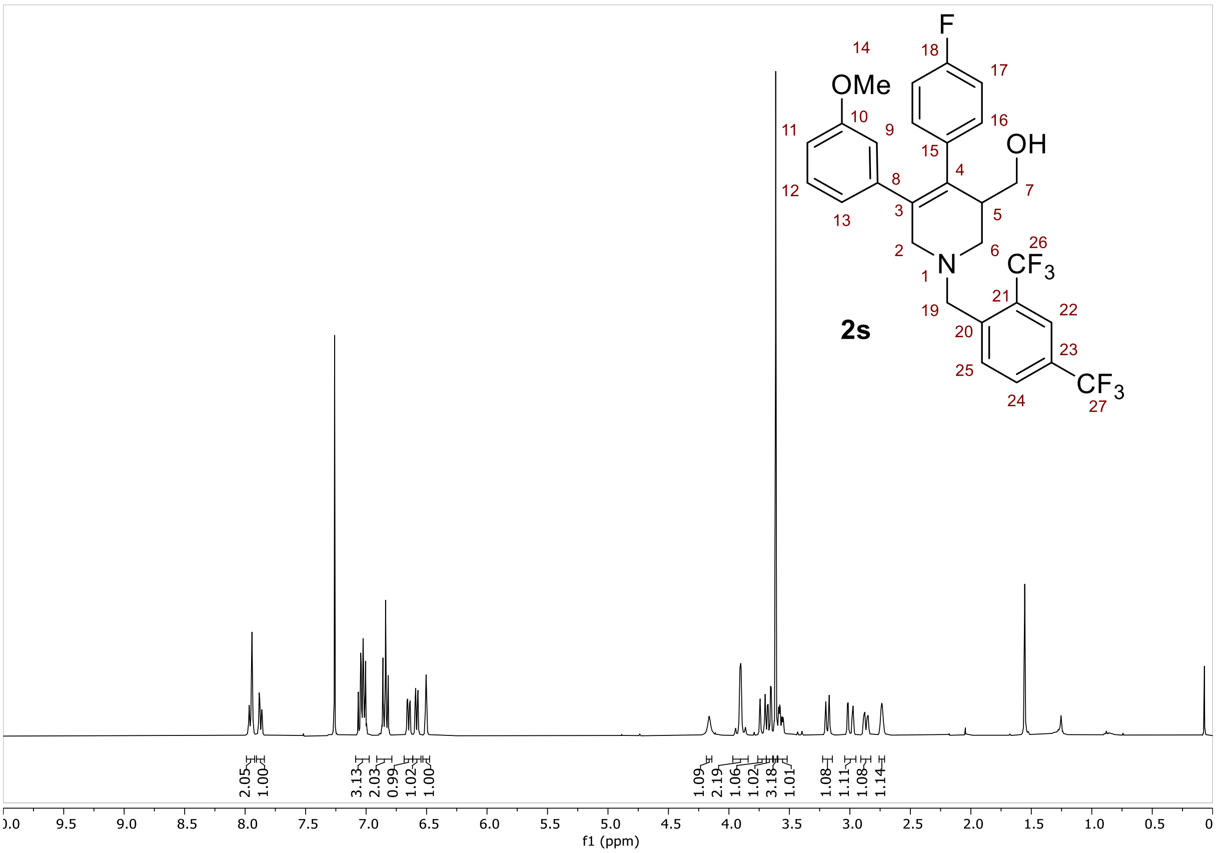
F
OMe
OH
N
CF3
CF3
2s
1 2 3 4 5 6 7 8 9 10 11 12 13 14 15 16 17 18 19 20 21 22 23 24 25 26 27
2.05 1.00 3.13 2.03 0.99 1.02 1.00 1.09 2.19 1.06 1.02 3.18 1.01 1.08 1.11 1.08 1.14
f1 (ppm)

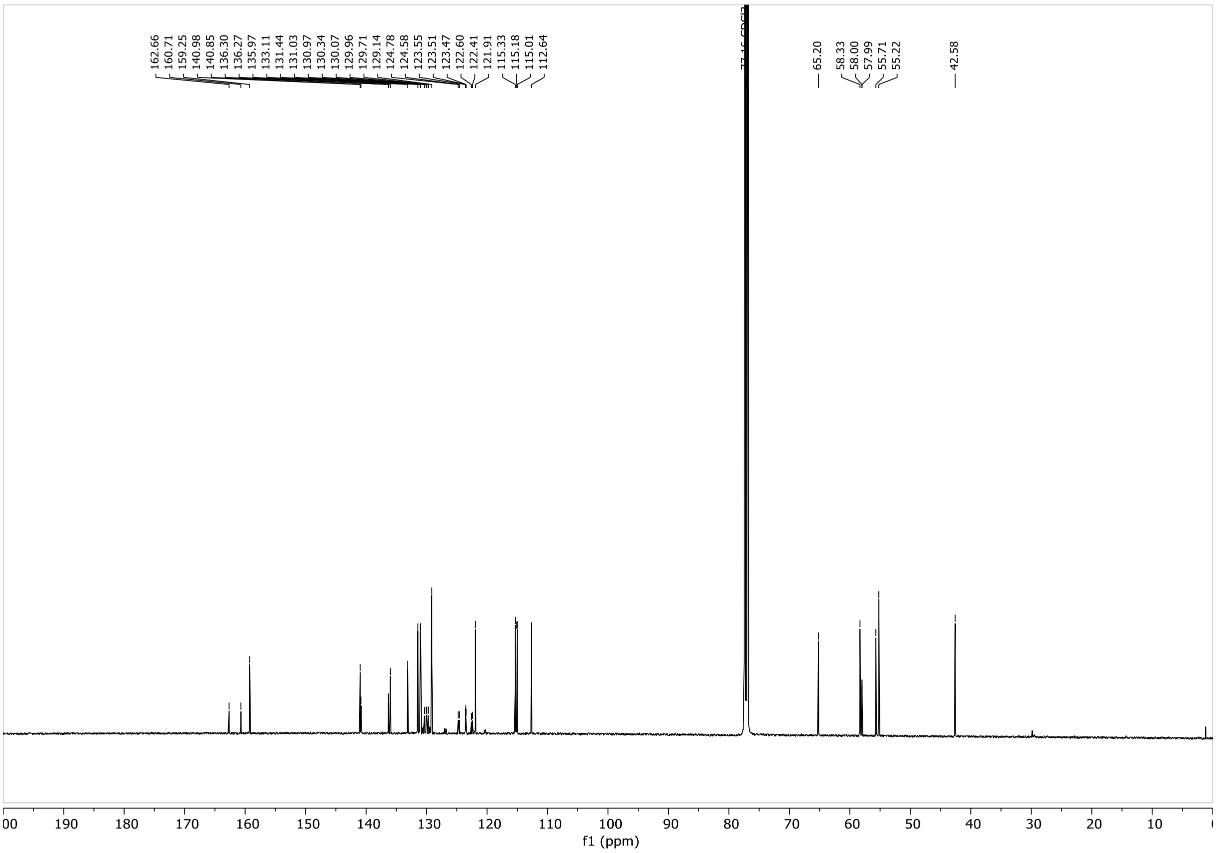
162.66 160.71 159.25 140.98 140.85 136.30 136.27 135.97 133.11 131.44 131.03 130.97 130.34 130.07 129.96 129.71 129.14 124.78 124.58 123.55 123.51 123.47 122.60 122.41 121.91 115.33 115.18 115.01 112.64
65.20 58.33 58.00 57.99 55.71 55.22 42.58
f1 (ppm)

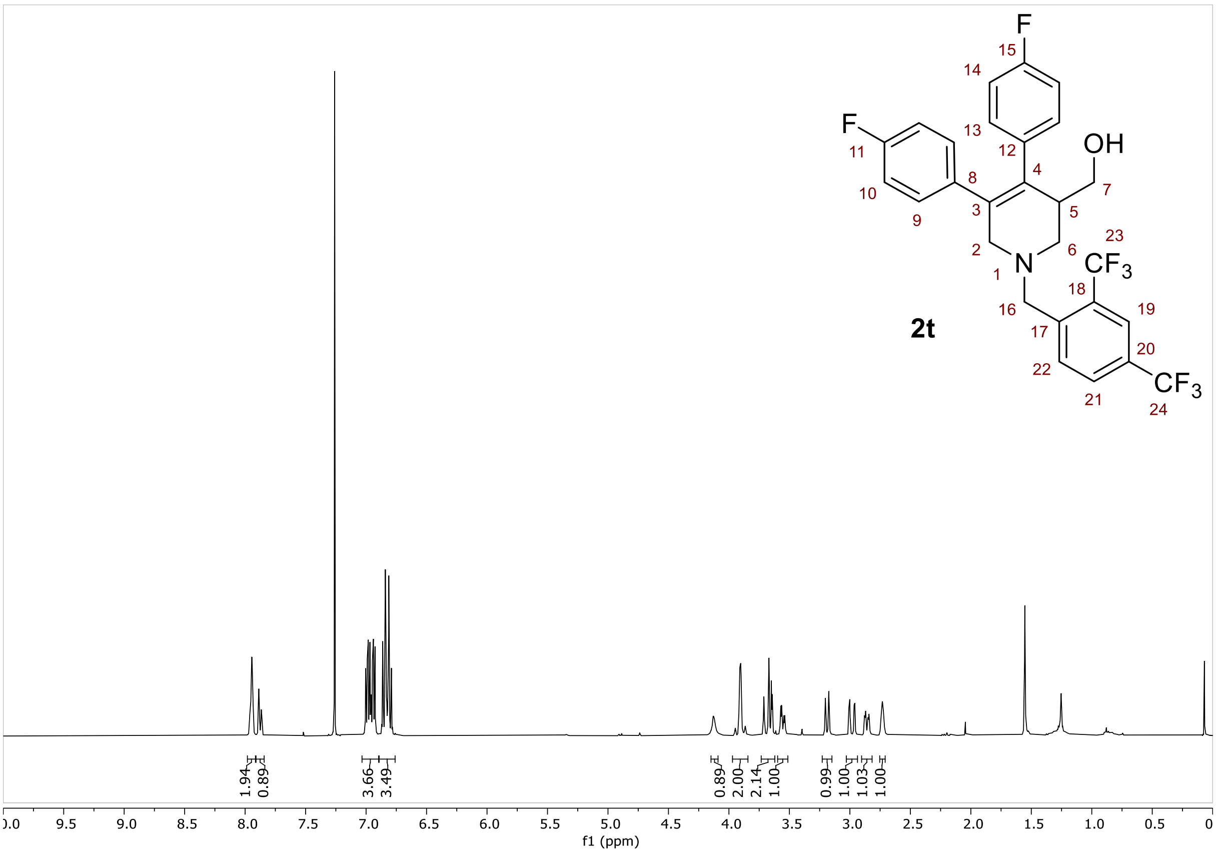

F
OH
F
CF3
CF3
N
2t
1.94
0.89
3.66
3.49
0.89
2.00
2.14
1.00
0.99
1.00
1.03
1.00
f1 (ppm)

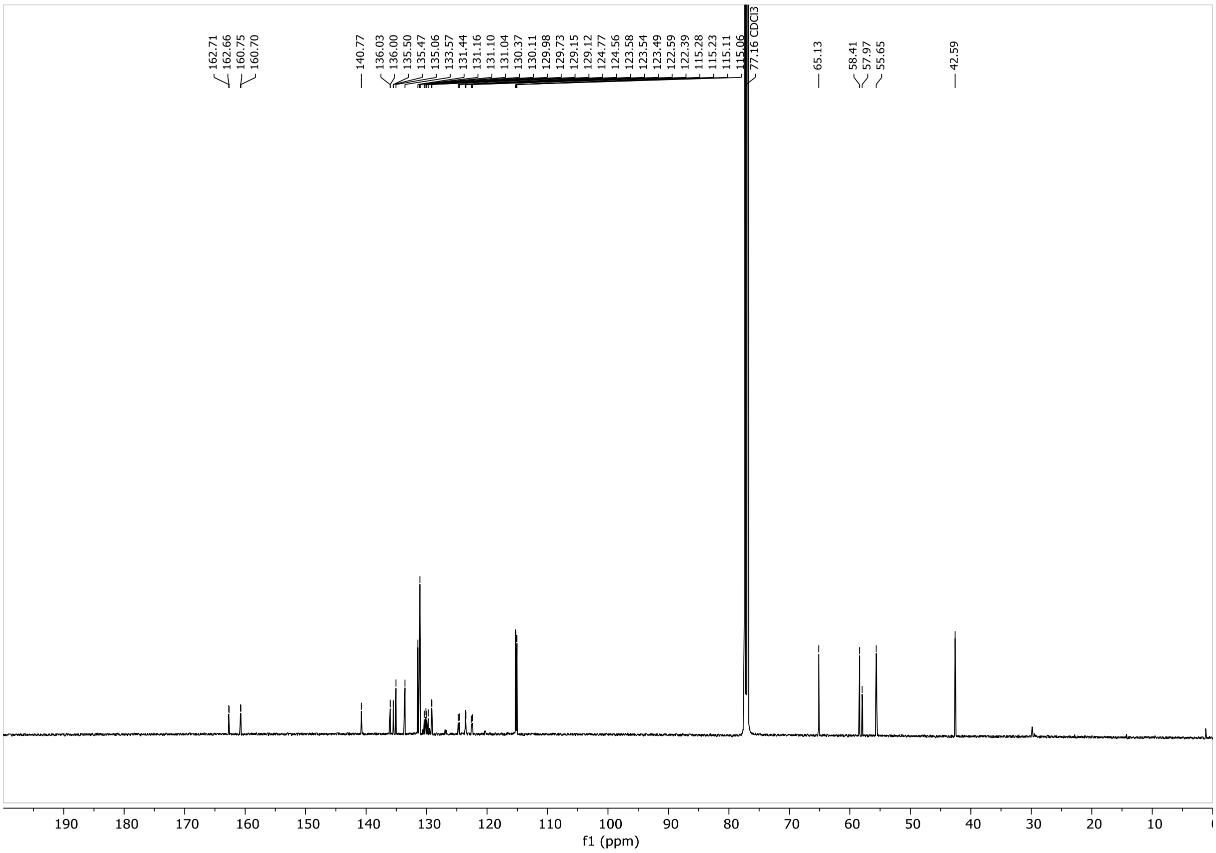

162.71
162.66
160.75
160.70
140.77
136.03
136.00
135.50
135.47
135.06
133.57
131.44
131.16
131.10
131.04
130.37
130.11
129.98
129.73
129.15
129.12
124.77
124.56
123.58
123.54
123.49
122.59
122.39
115.28
115.23
115.11
115.06
77.16 CDCl3
65.13
58.41
57.97
55.65
42.59
f1 (ppm)

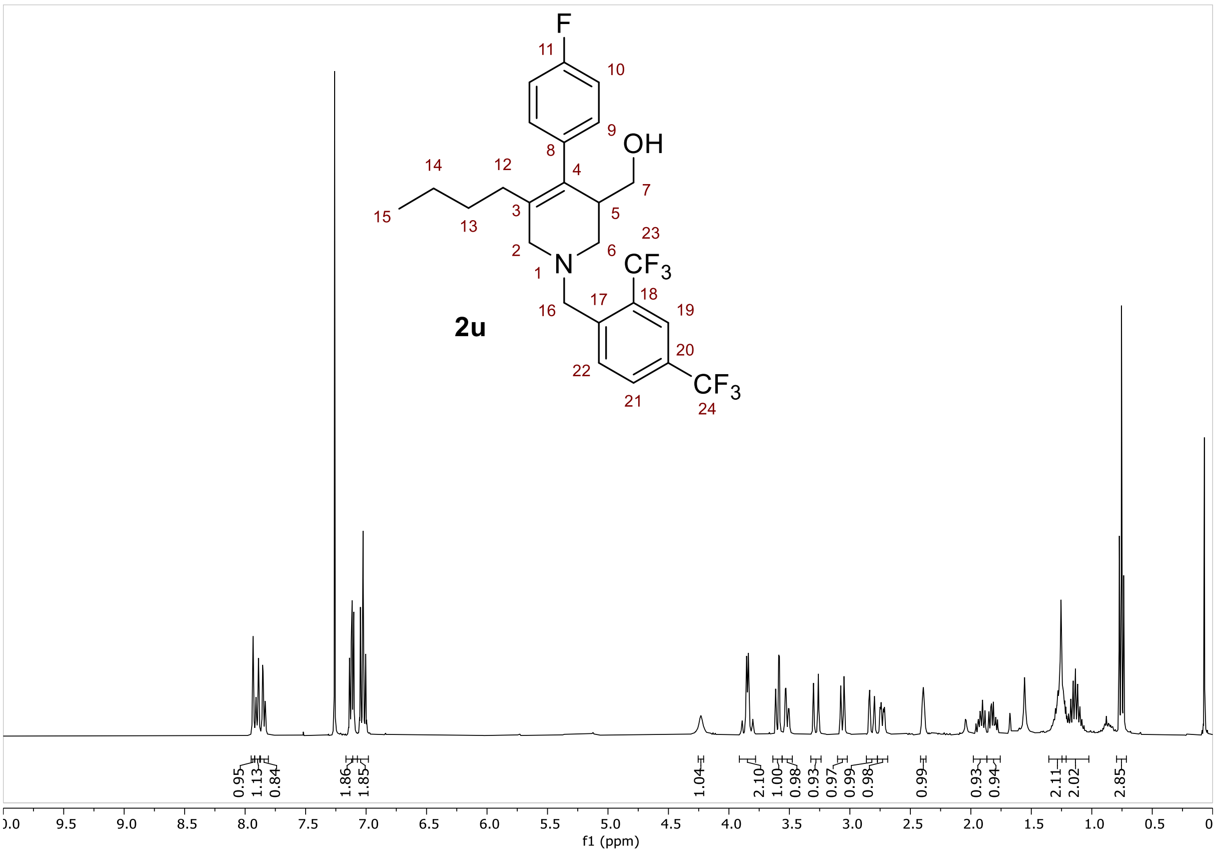

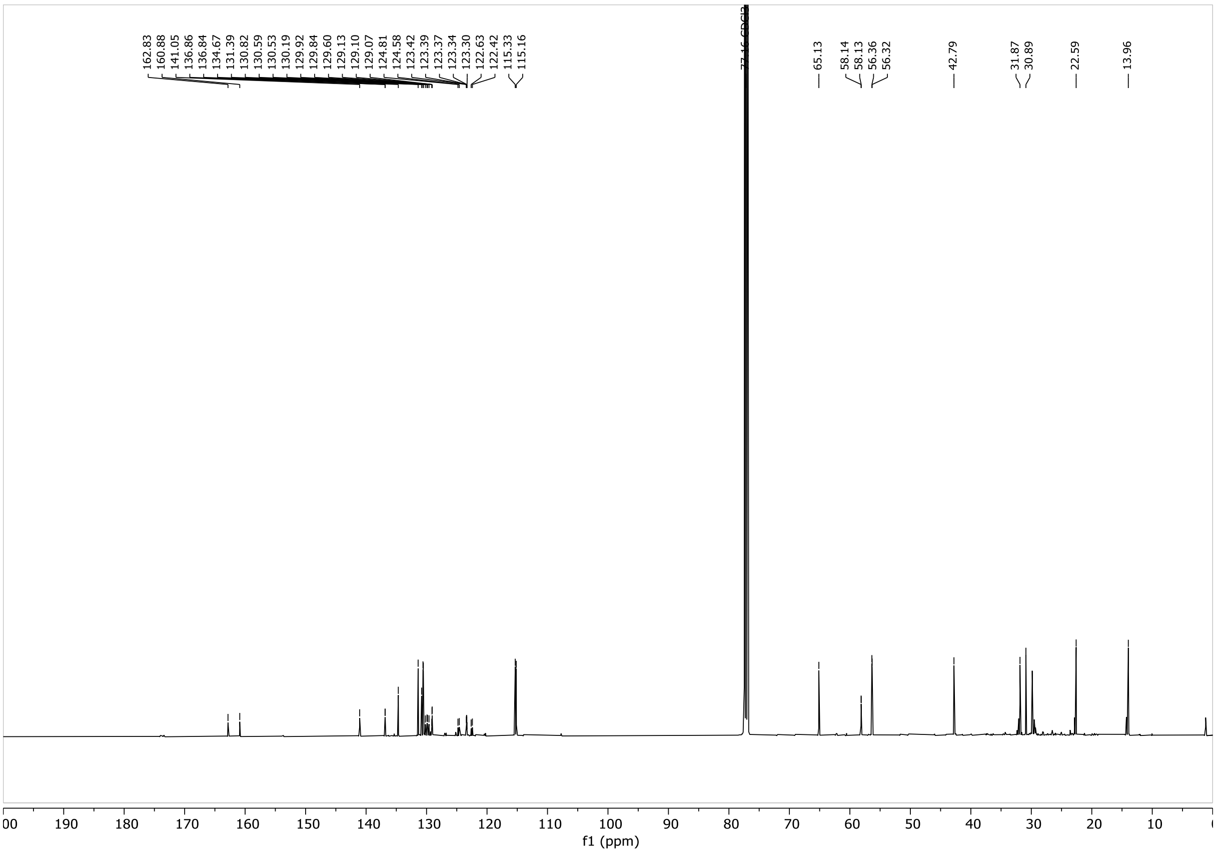

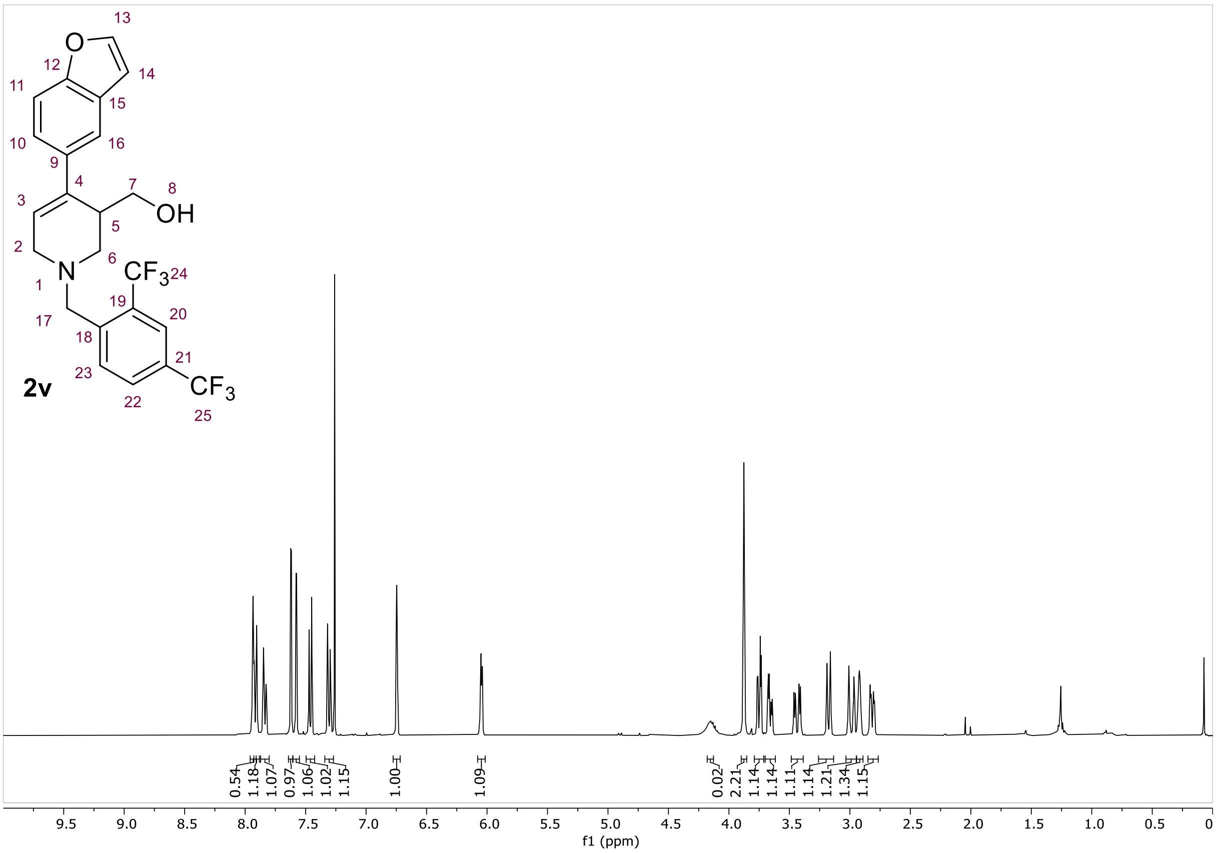

2v
OH
N
O
CF3
CF3
0.54
1.18
1.07
0.97
1.06
1.02
1.15
1.00
1.09
0.02
2.21
1.14
1.14
1.11
1.14
1.21
1.34
1.15
f1 (ppm)

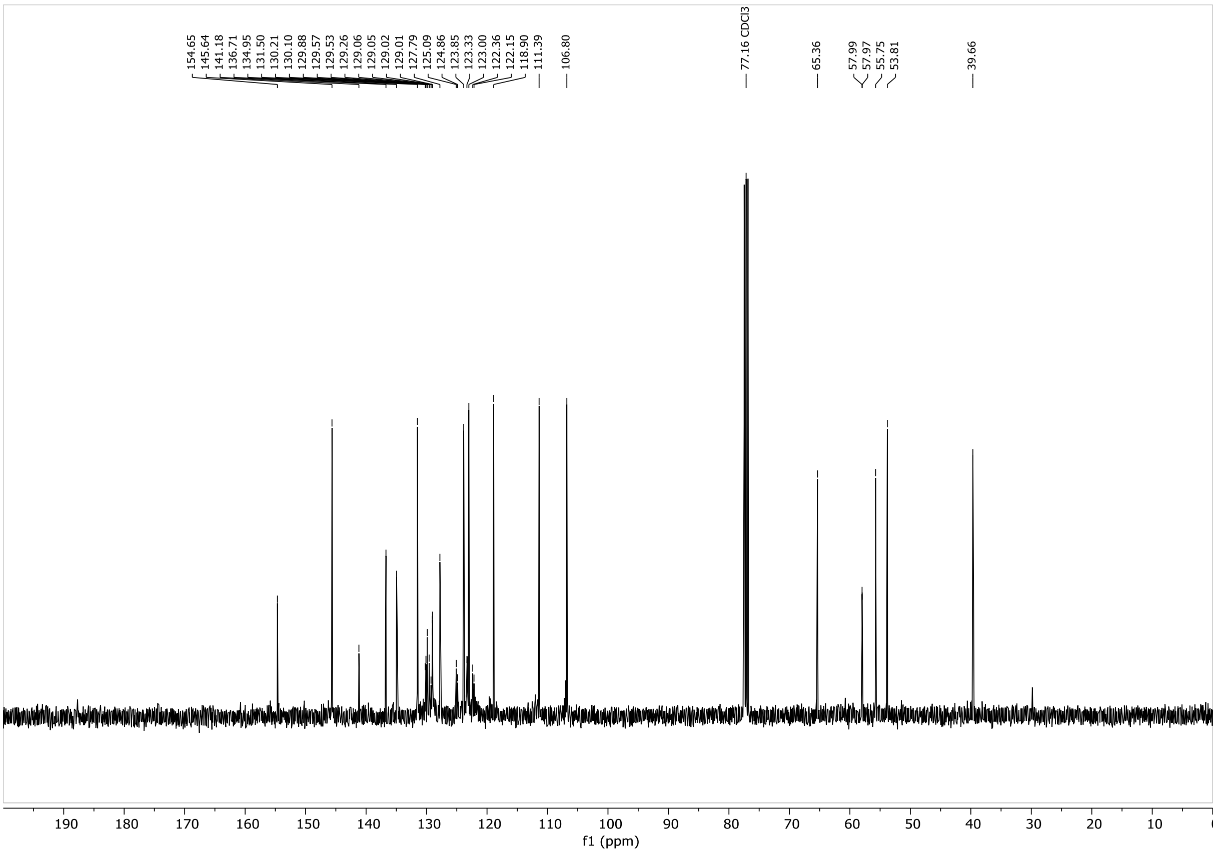

154.65
145.64
141.18
136.71
134.95
131.50
130.21
130.10
129.88
129.57
129.53
129.26
129.06
129.05
129.02
129.01
127.79
125.09
124.86
123.85
123.33
123.00
122.36
122.15
118.90
111.39
106.80
77.16 CDCl3
65.36
57.99
57.97
55.75
53.81
39.66
f1 (ppm)

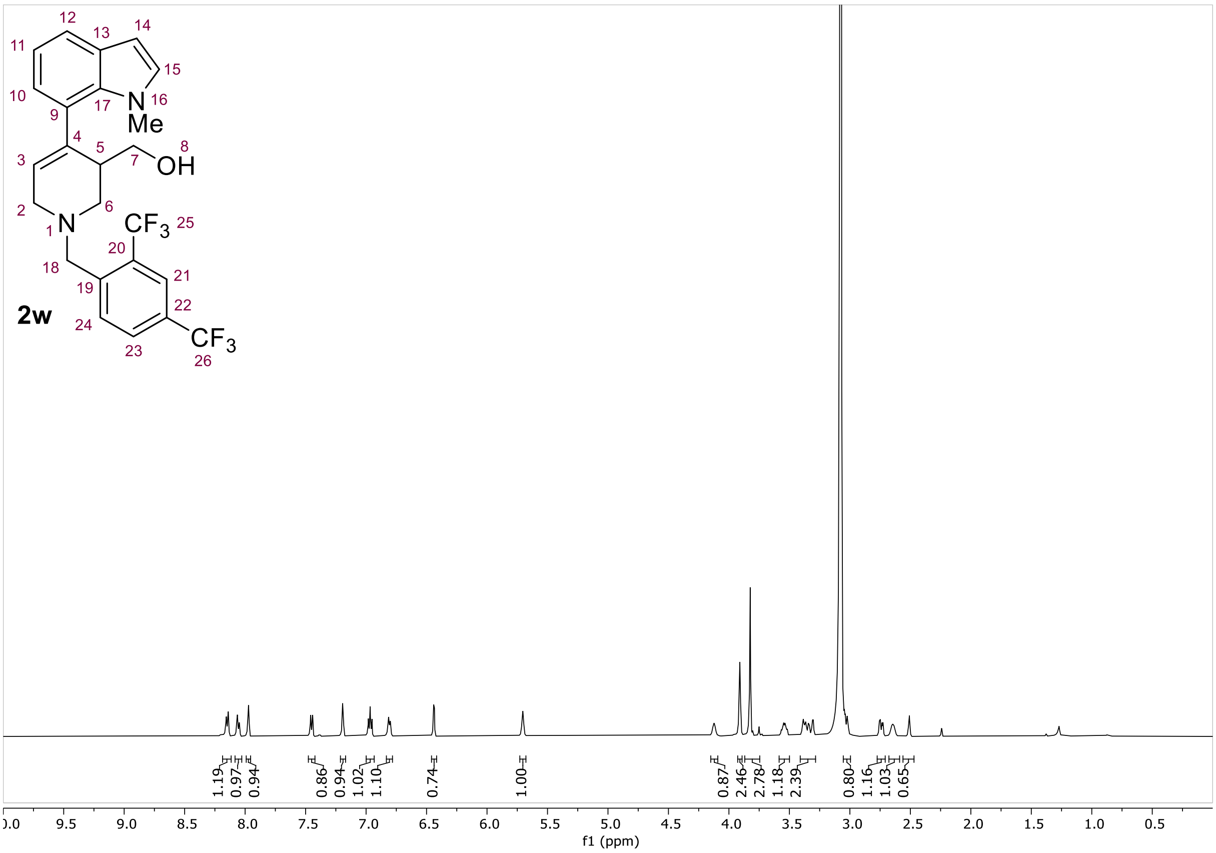
2w
OH
Me
CF_3
CF_3
1.19
0.97
0.94
0.86
0.94
1.02
1.10
0.74
1.00
0.87
2.46
2.78
1.18
2.39
0.80
1.16
1.03
0.65
f1 (ppm)

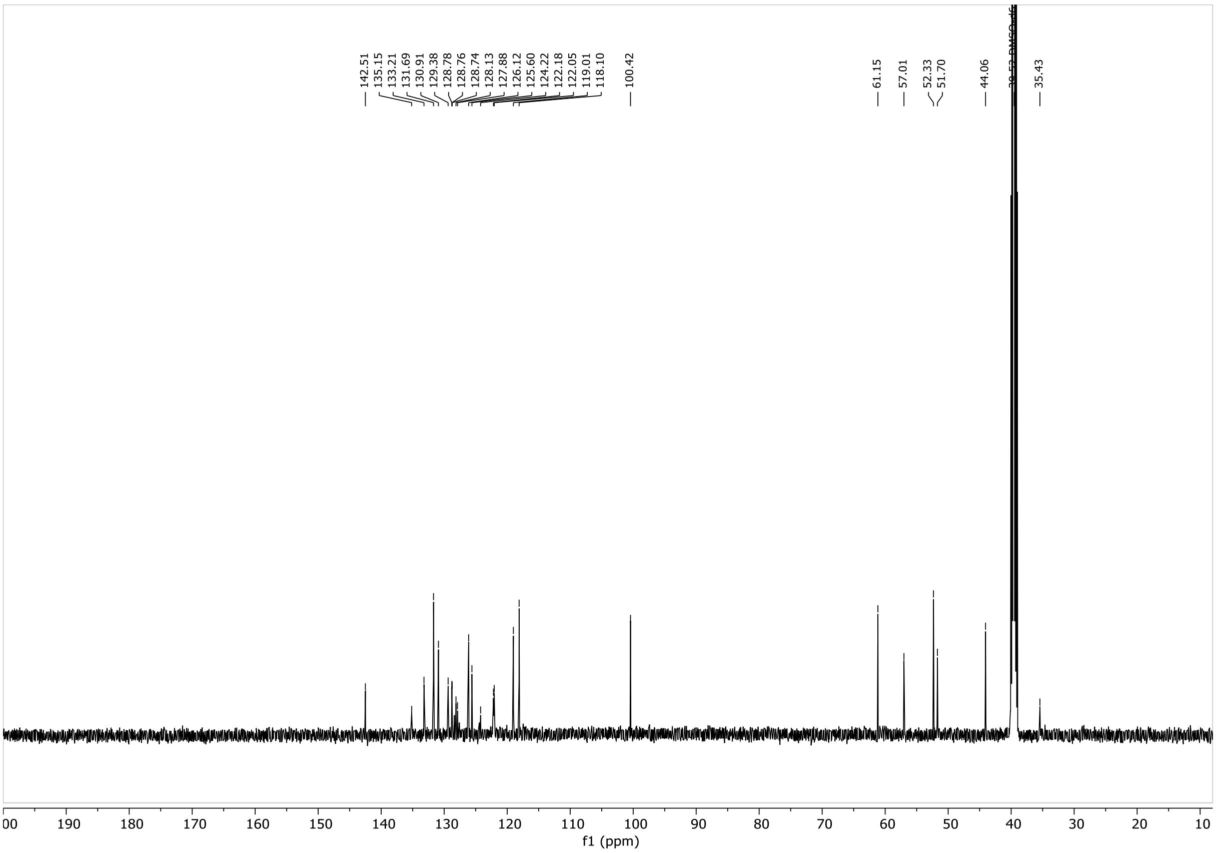
142.51
135.15
133.21
131.69
130.91
129.38
128.78
128.76
128.74
128.13
127.88
126.12
125.60
124.22
122.18
122.05
119.01
118.10
100.42
61.15
57.01
52.33
51.70
44.06
35.43
f1 (ppm)

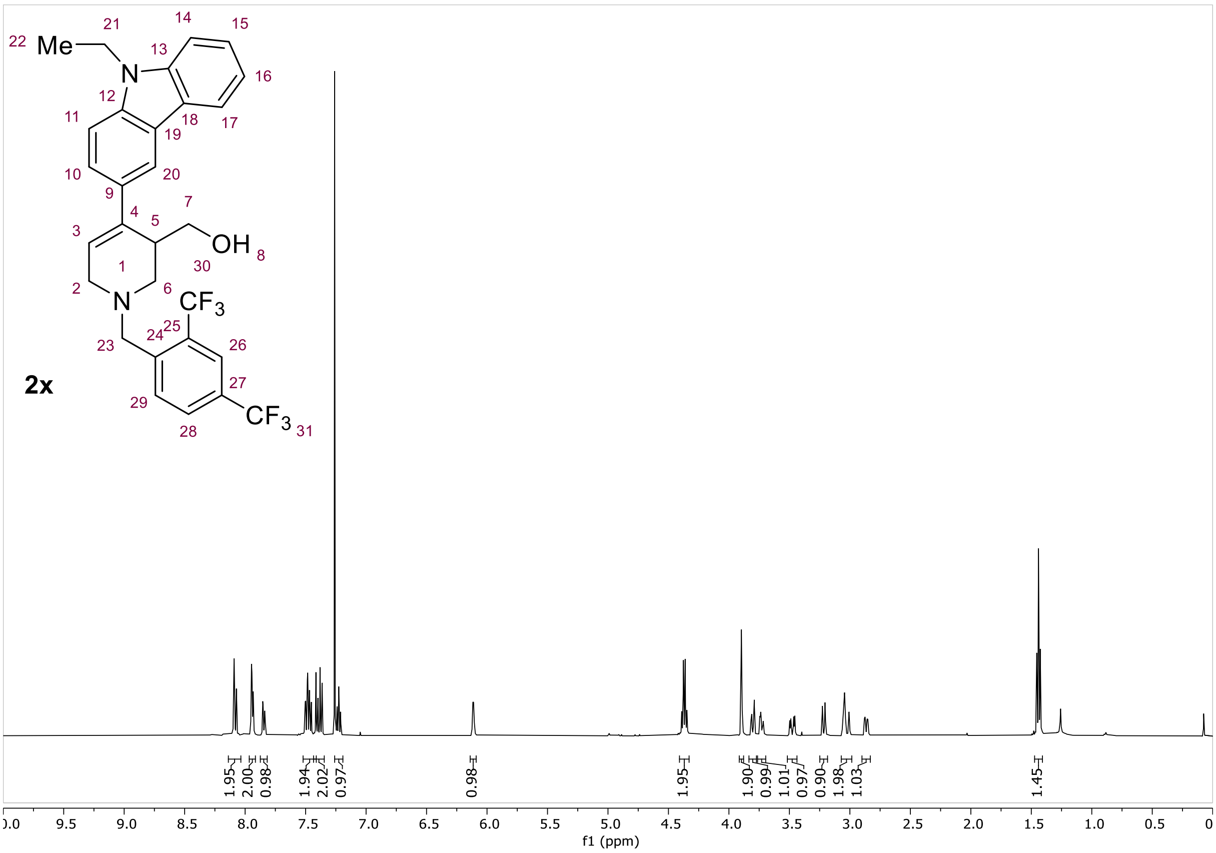

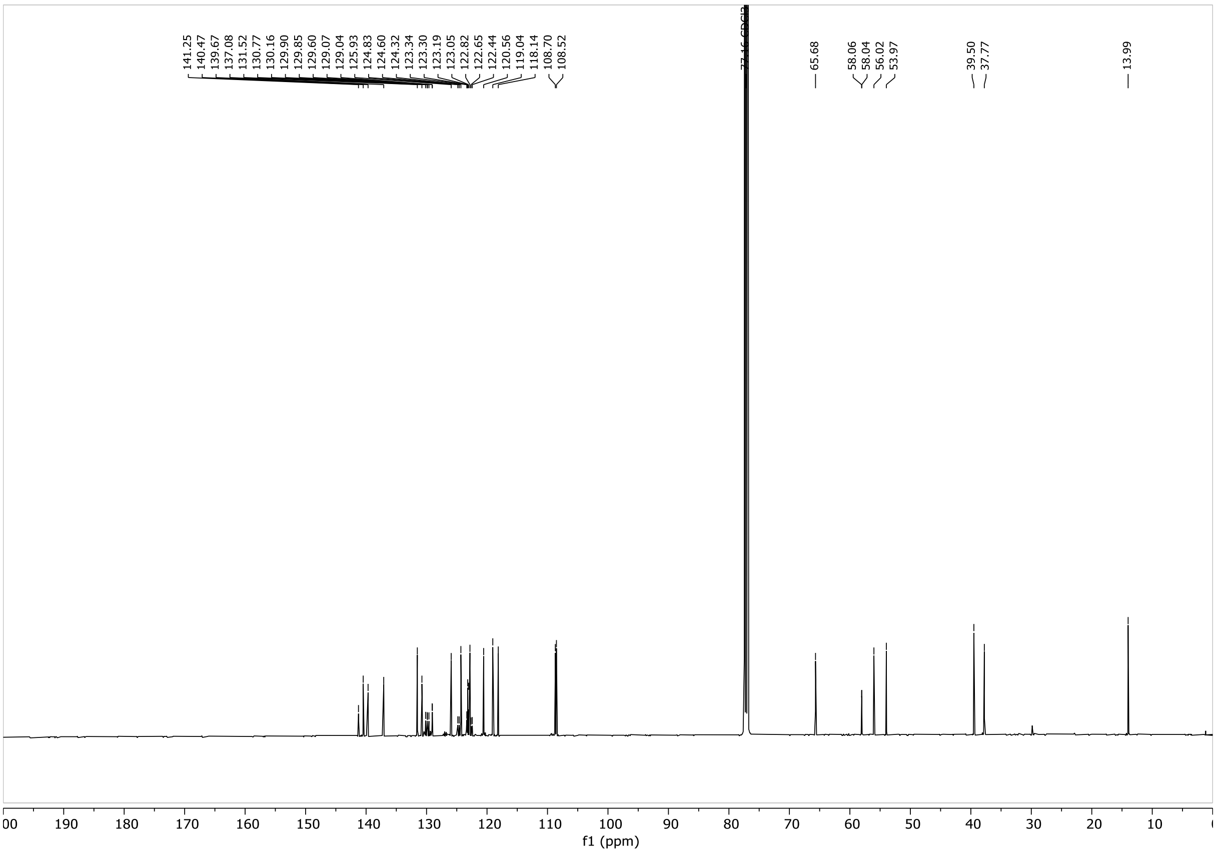

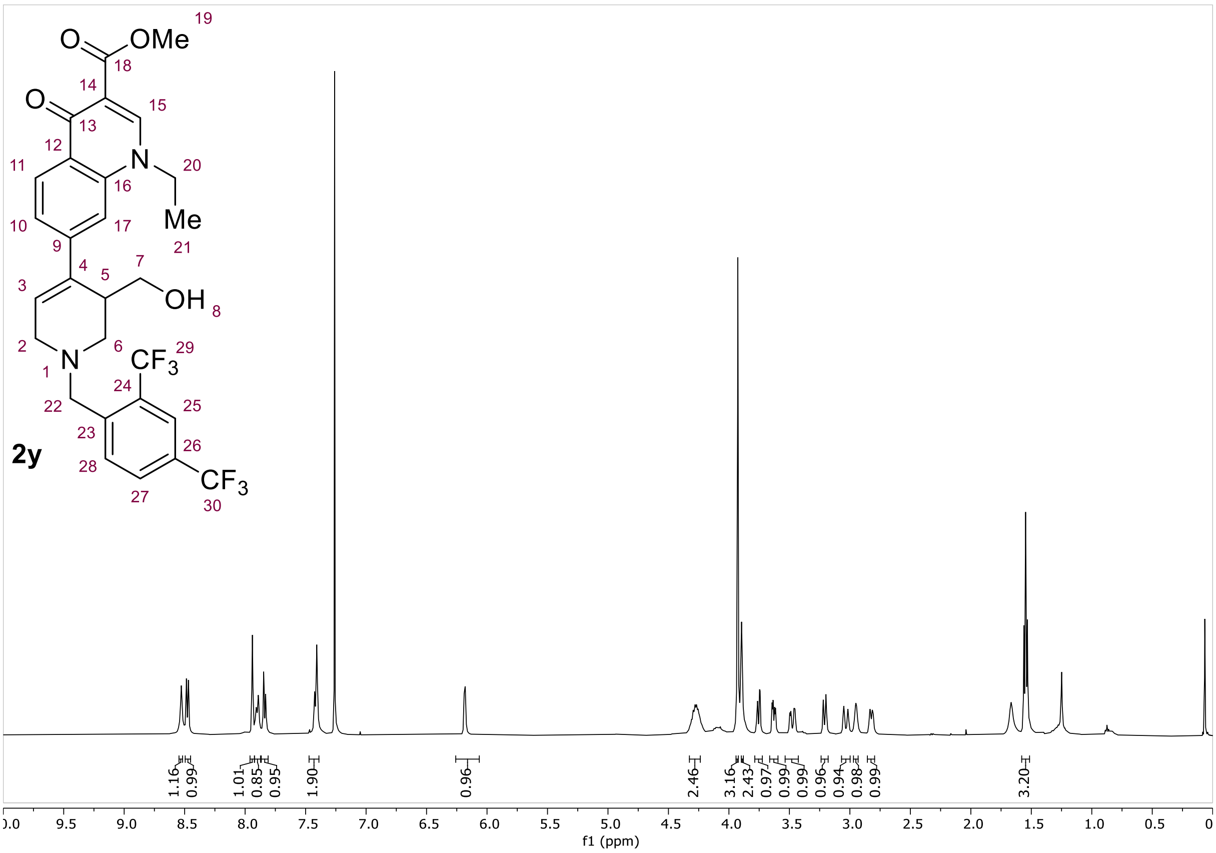

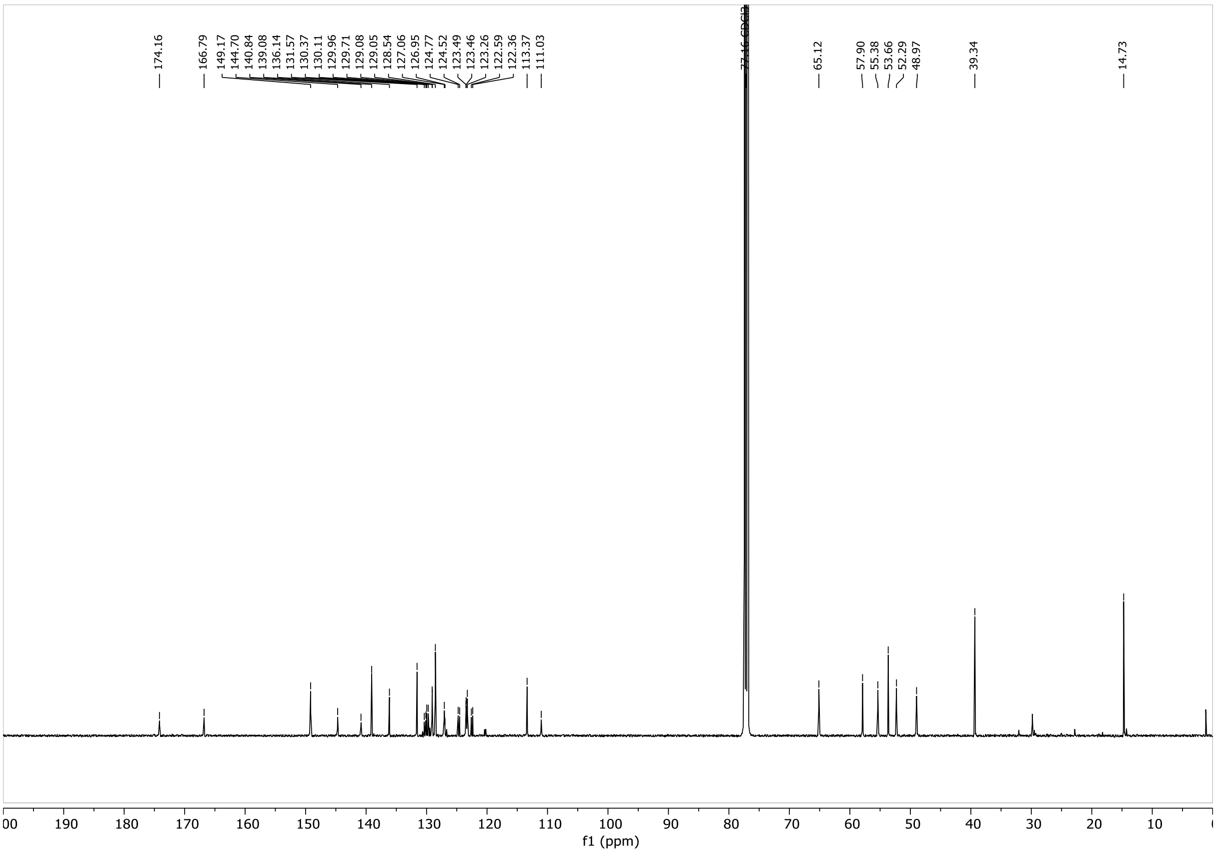

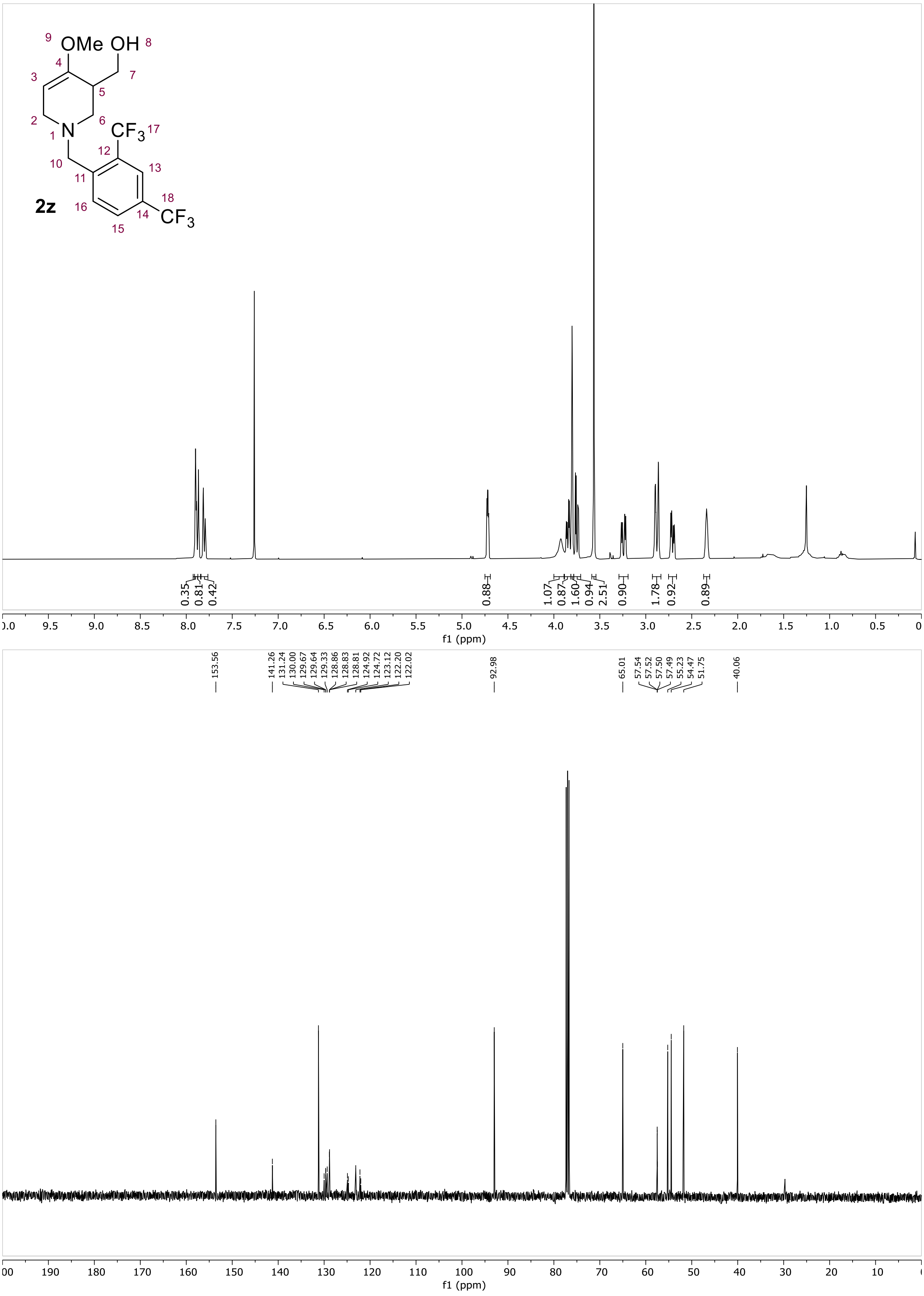

9
OMe
OH
8
4
3
7
5
2
6
1
N
CF3
17
12
10
13
11
18
2z
16
14
CF3
15
0.35
0.81
0.42
0.88
1.07
0.87
1.60
0.94
2.51
0.90
1.78
0.92
0.89
f1 (ppm)
153.56
141.26
131.24
130.00
129.67
129.64
129.33
128.86
128.83
128.81
124.92
124.72
123.12
122.20
122.02
92.98
65.01
57.54
57.52
57.50
57.49
55.23
54.47
51.75
40.06
f1 (ppm)

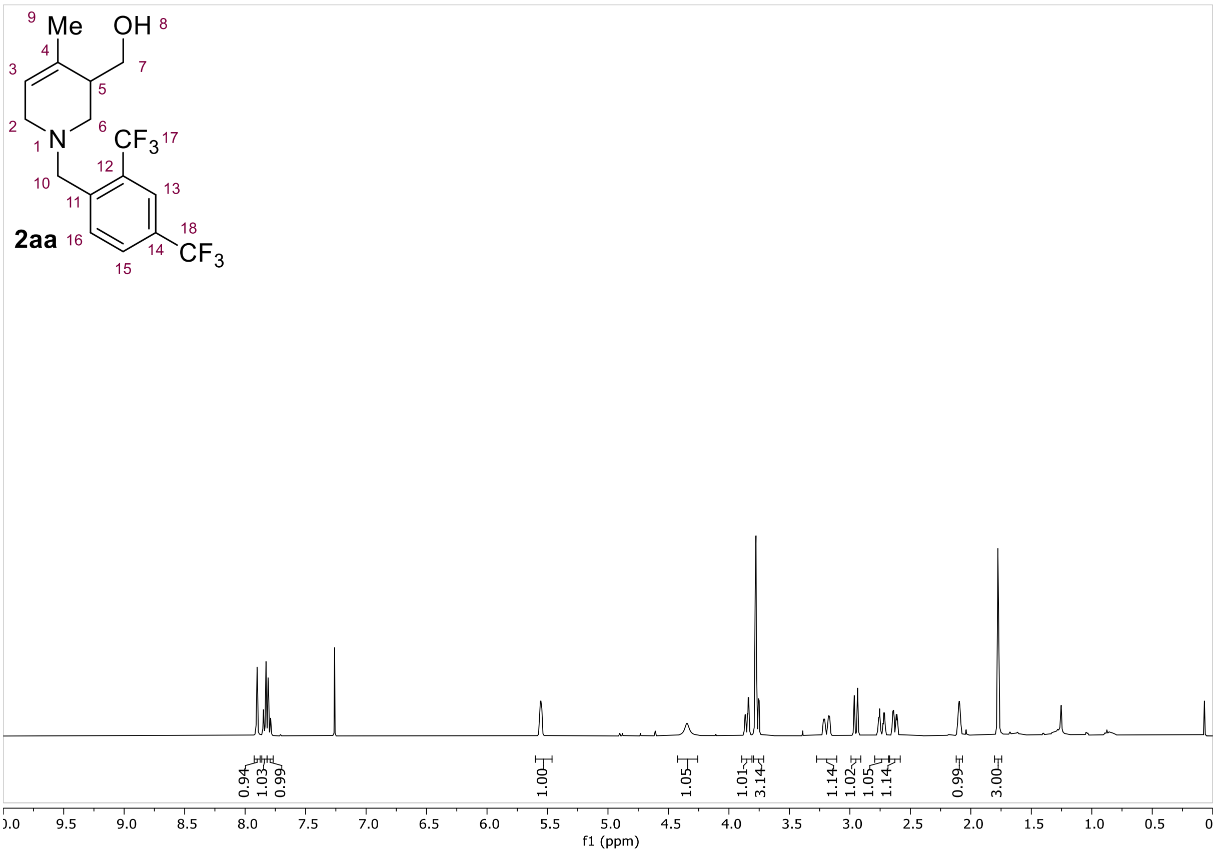

9 Me
OH 8
7
3
4
5
2
6
1 N
CF3 17
12
10
13
11
18
2aa 16
14 CF3
15
0.94
1.03
0.99
1.00
1.05
1.01
3.14
1.14
1.02
1.05
1.14
0.99
3.00
9.5 9.0 8.5 8.0 7.5 7.0 6.5 6.0 5.5 5.0 4.5 4.0 3.5 3.0 2.5 2.0 1.5 1.0 0.5
f1 (ppm)

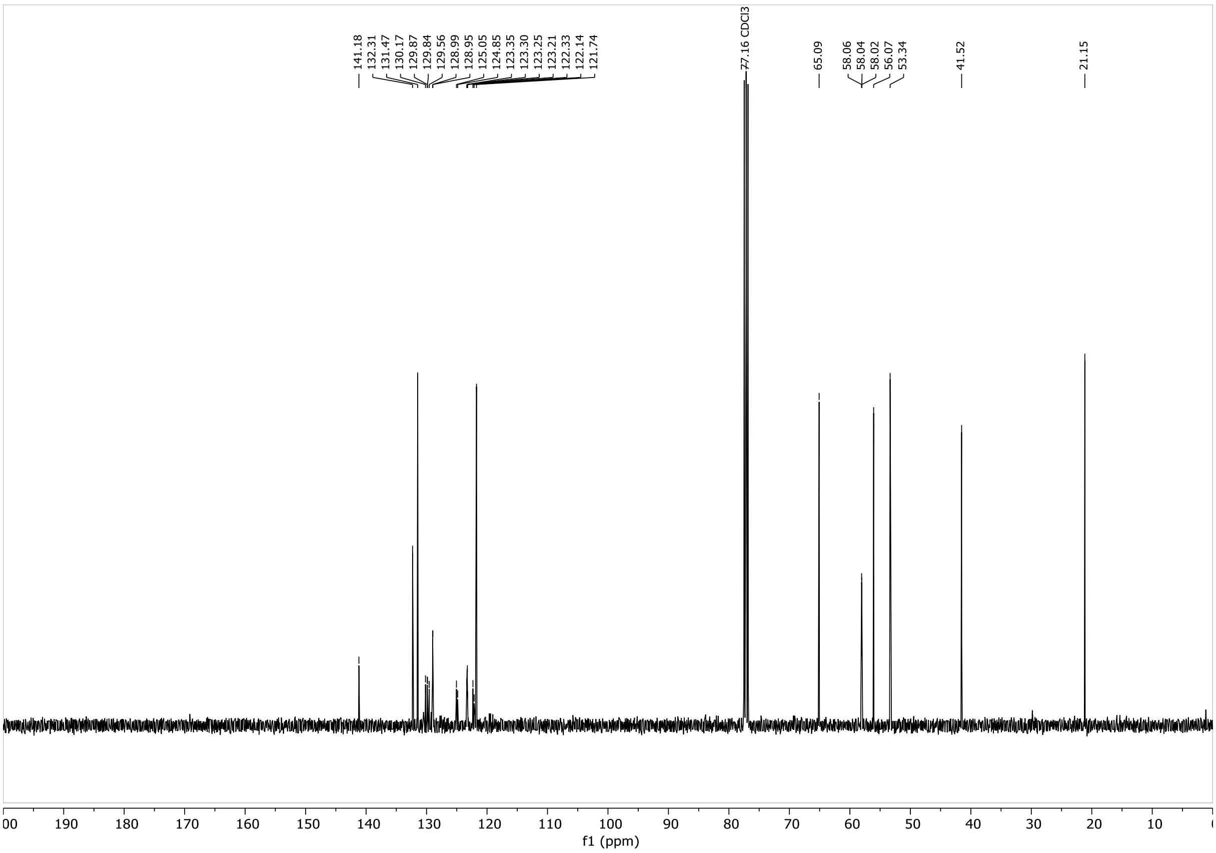

141.18
132.31
131.47
130.17
129.87
129.84
129.56
128.99
128.95
125.05
124.85
123.35
123.30
123.25
123.21
122.33
122.14
121.74
77.16 CDCl3
65.09
58.06
58.04
58.02
56.07
53.34
41.52
21.15
190 180 170 160 150 140 130 120 110 100 90 80 70 60 50 40 30 20 10
f1 (ppm)

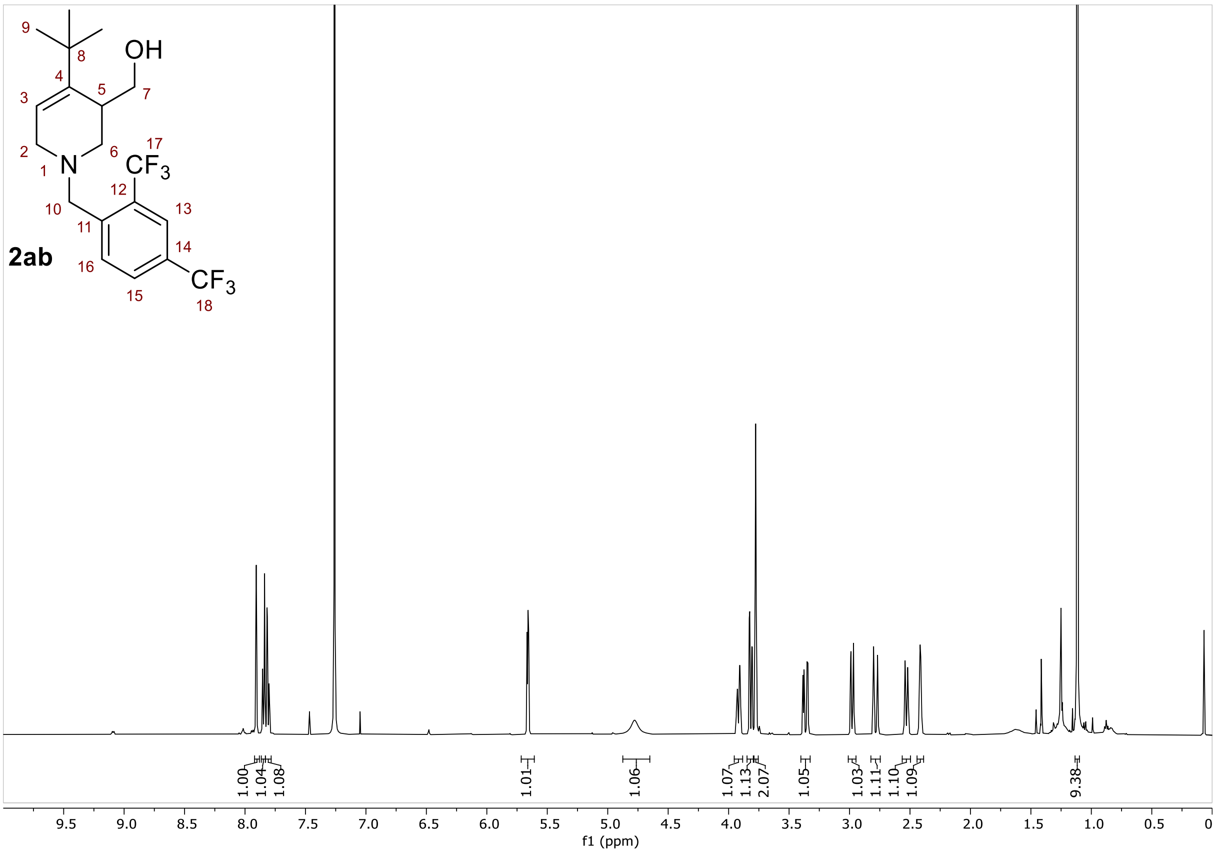
OH
CF3
CF3
2ab
1.00
1.04
1.08
1.01
1.06
1.07
1.13
2.07
1.05
1.03
1.11
1.10
1.09
9.38
f1 (ppm)

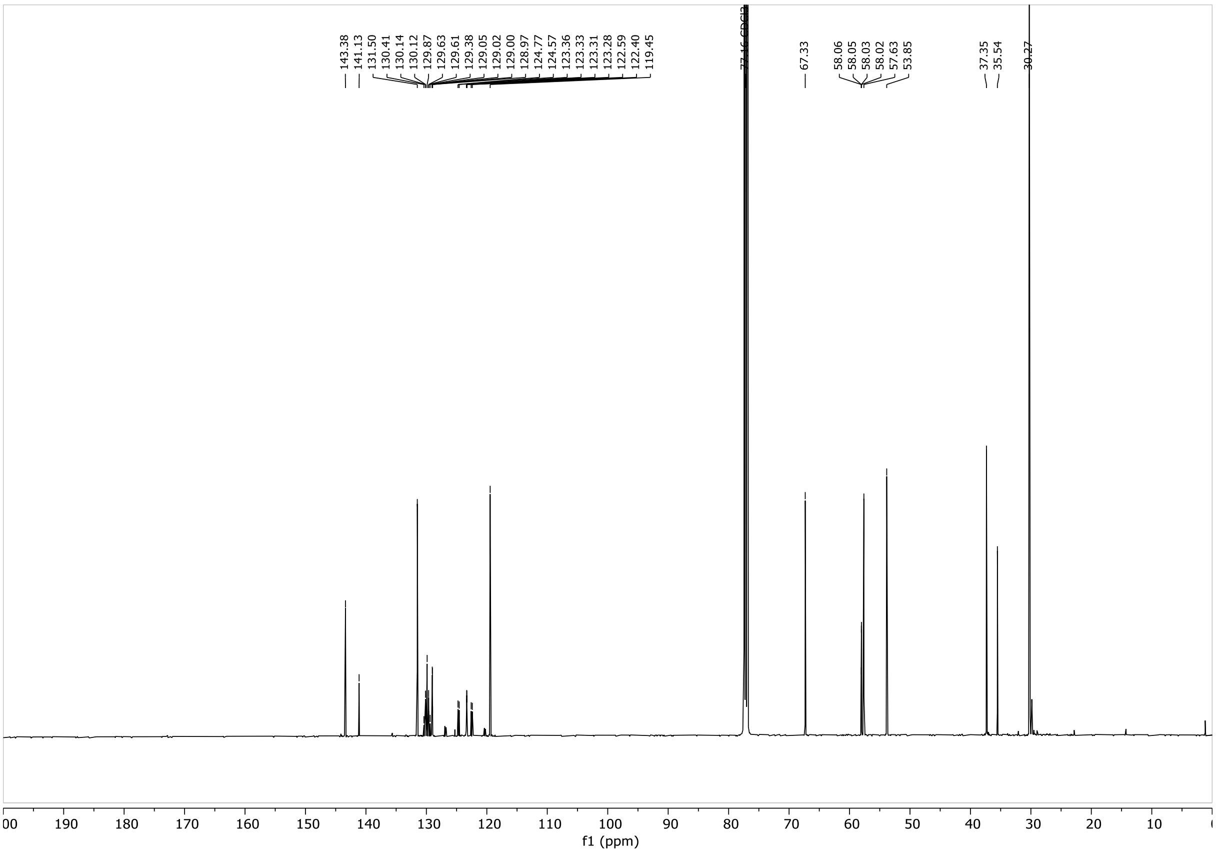
143.38
141.13
131.50
130.41
130.14
130.12
129.87
129.63
129.61
129.38
129.05
129.02
129.00
128.97
124.77
124.57
123.36
123.33
123.31
123.28
122.59
122.40
119.45
67.33
58.06
58.05
58.03
58.02
57.63
53.85
37.35
35.54
30.27
f1 (ppm)

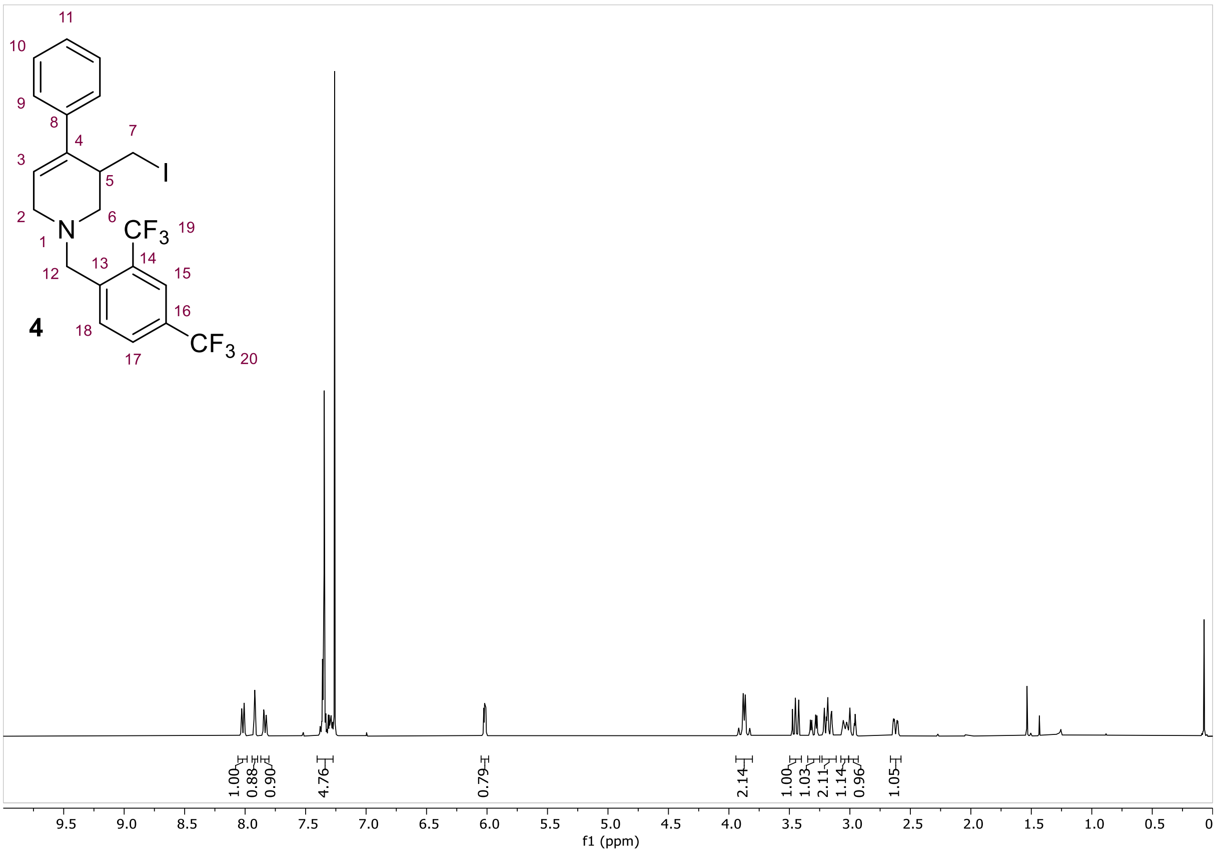

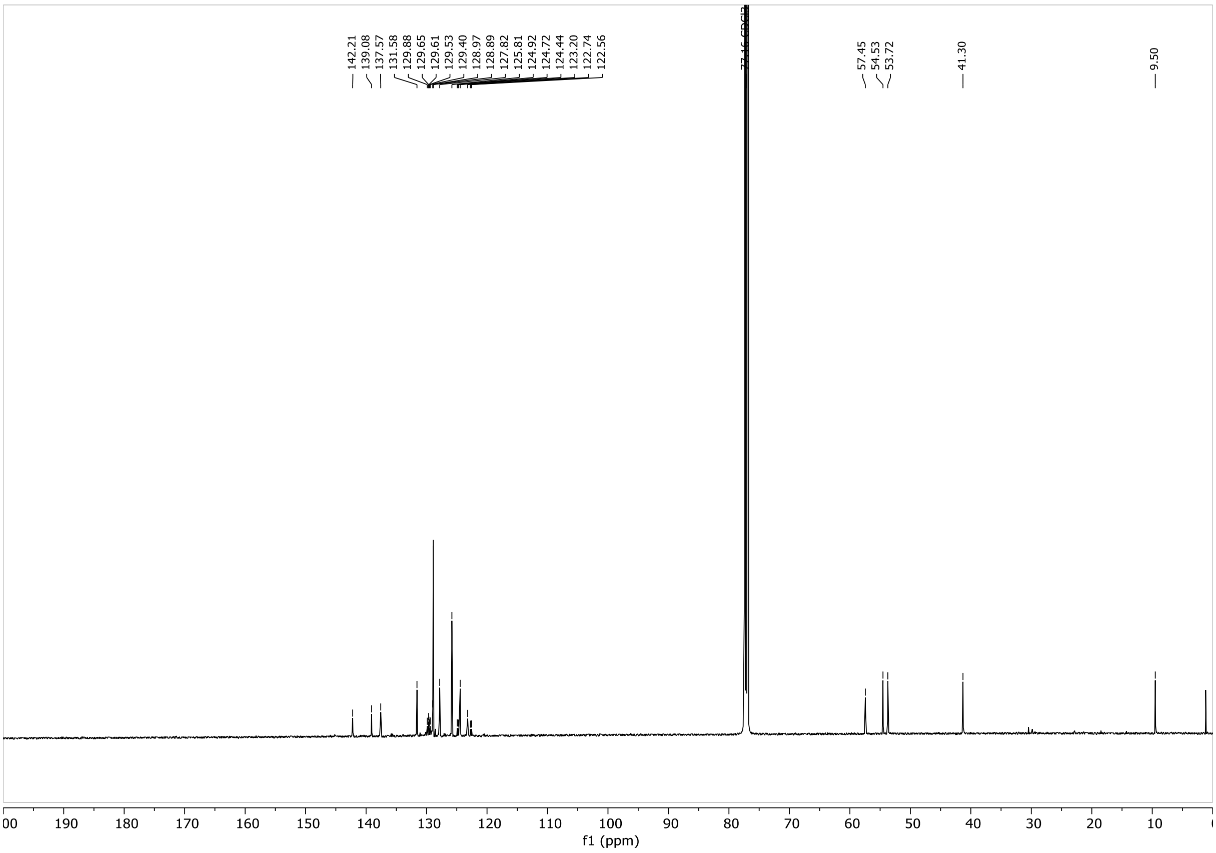

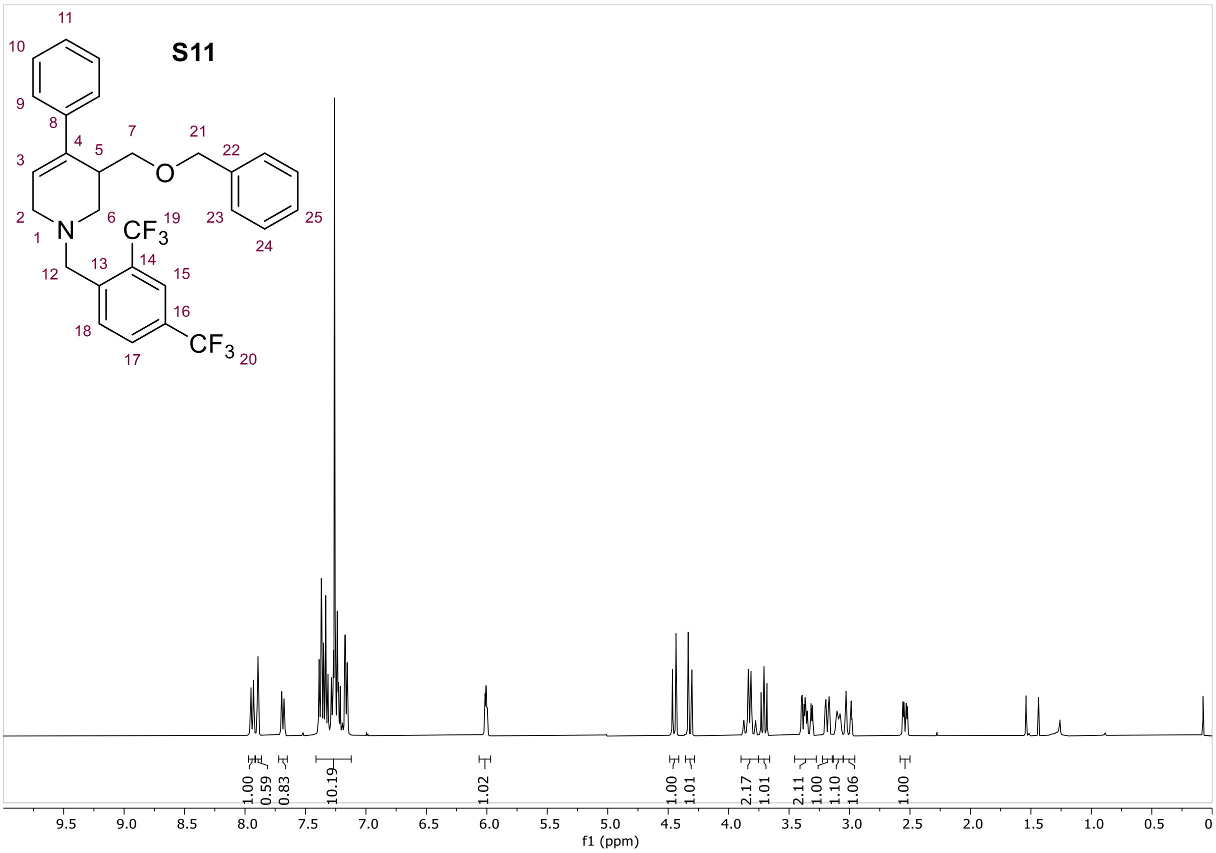
S11
CF3
CF3
N
O
1.00
0.59
0.83
10.19
1.02
1.00
1.01
2.17
1.01
2.11
1.00
1.10
1.06
1.00
9.5 9.0 8.5 8.0 7.5 7.0 6.5 6.0 5.5 5.0 4.5 4.0 3.5 3.0 2.5 2.0 1.5 1.0 0.5 0
f1 (ppm)

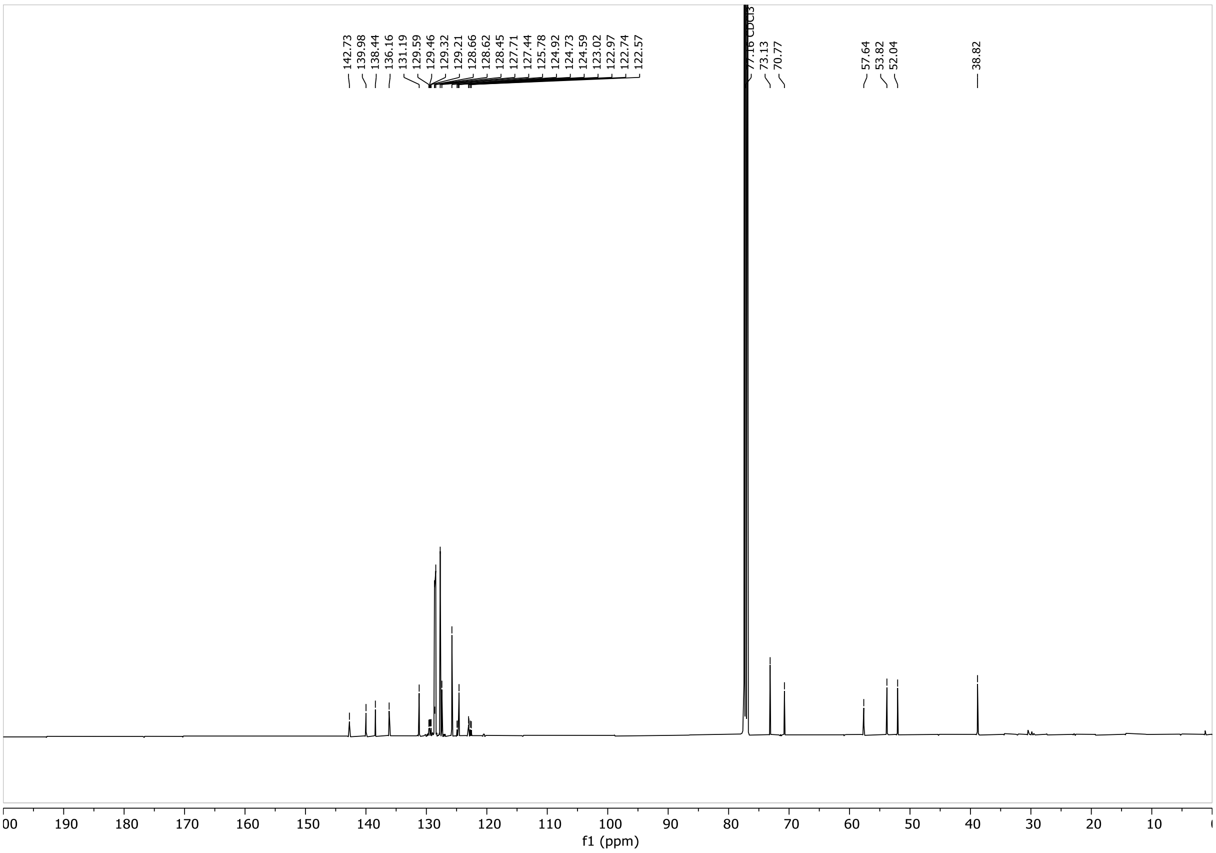
142.73
139.98
138.44
136.16
131.19
129.59
129.46
129.32
129.21
128.62
128.66
128.45
127.71
127.44
125.78
124.92
124.73
124.59
123.02
122.97
122.74
122.57
77.16 CDCl3
73.13
70.77
57.64
53.82
52.04
38.82
190 180 170 160 150 140 130 120 110 100 90 80 70 60 50 40 30 20 10
f1 (ppm)

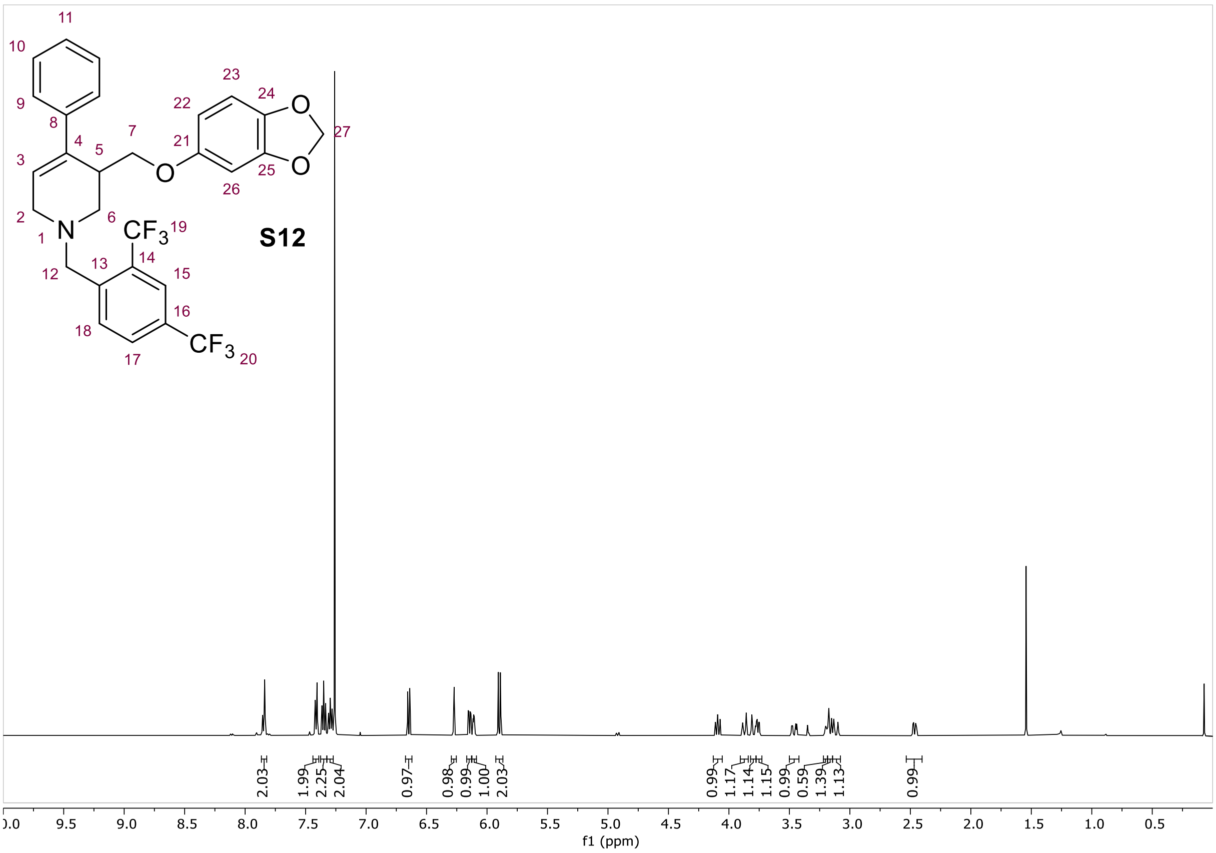
S12
2.03
1.99
2.25
2.04
0.97
0.98
0.99
1.00
2.03
0.99
1.17
1.14
1.15
0.99
0.59
1.39
1.13
0.99
f1 (ppm)

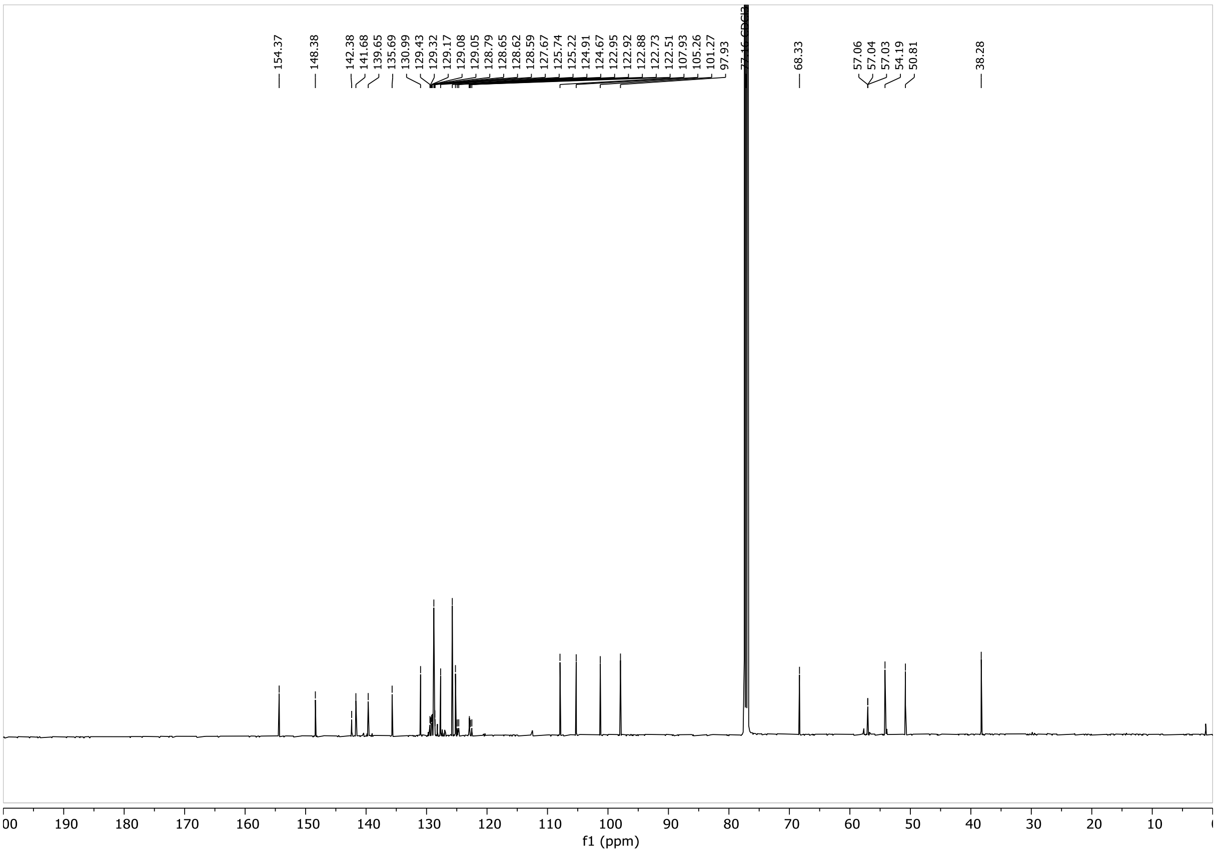
154.37
148.38
142.38
141.68
139.65
135.69
130.99
129.43
129.32
129.17
129.08
129.05
128.79
128.65
128.62
128.59
127.67
125.74
125.22
124.91
124.67
122.95
122.92
122.88
122.73
122.51
107.93
105.26
101.27
97.93
68.33
57.06
57.04
57.03
54.19
50.81
38.28
f1 (ppm)

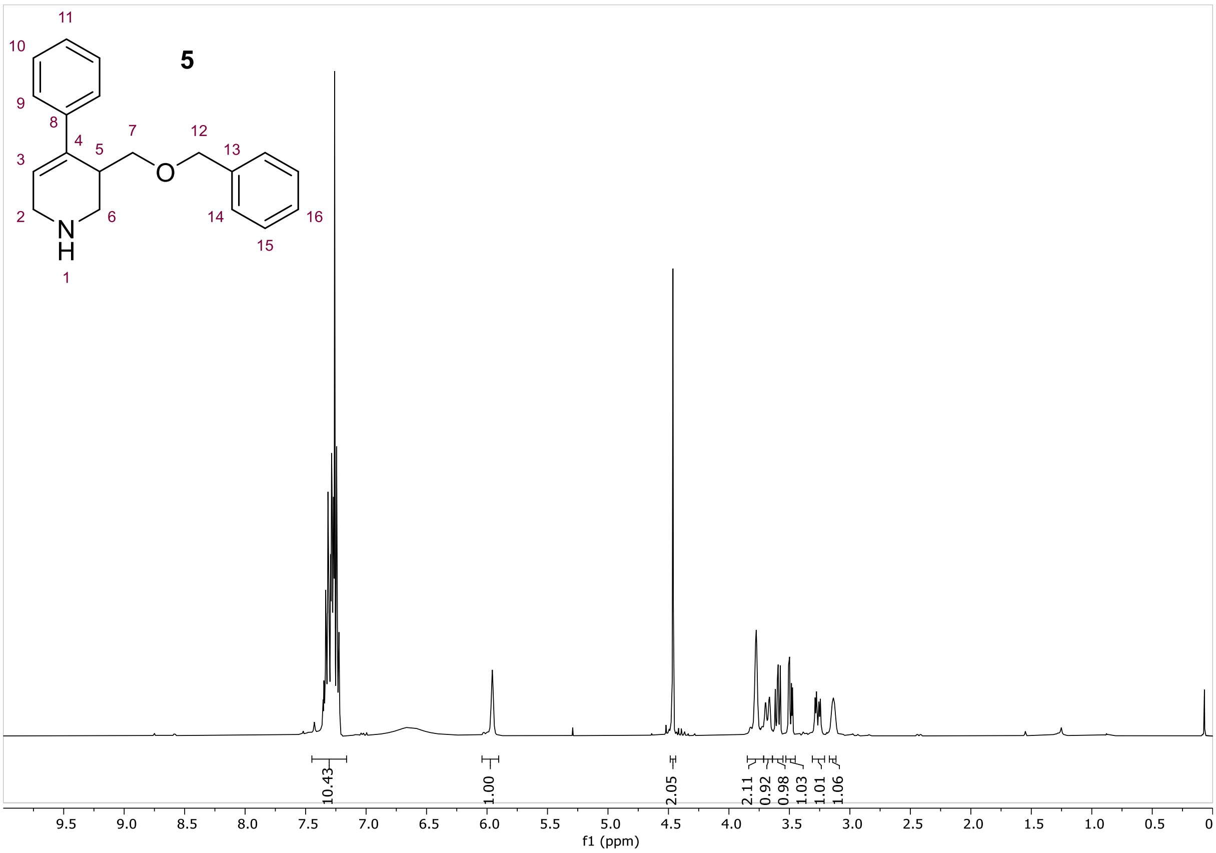
5
10.43
1.00
2.05
2.11
0.92
0.98
1.03
1.01
1.06
f1 (ppm)

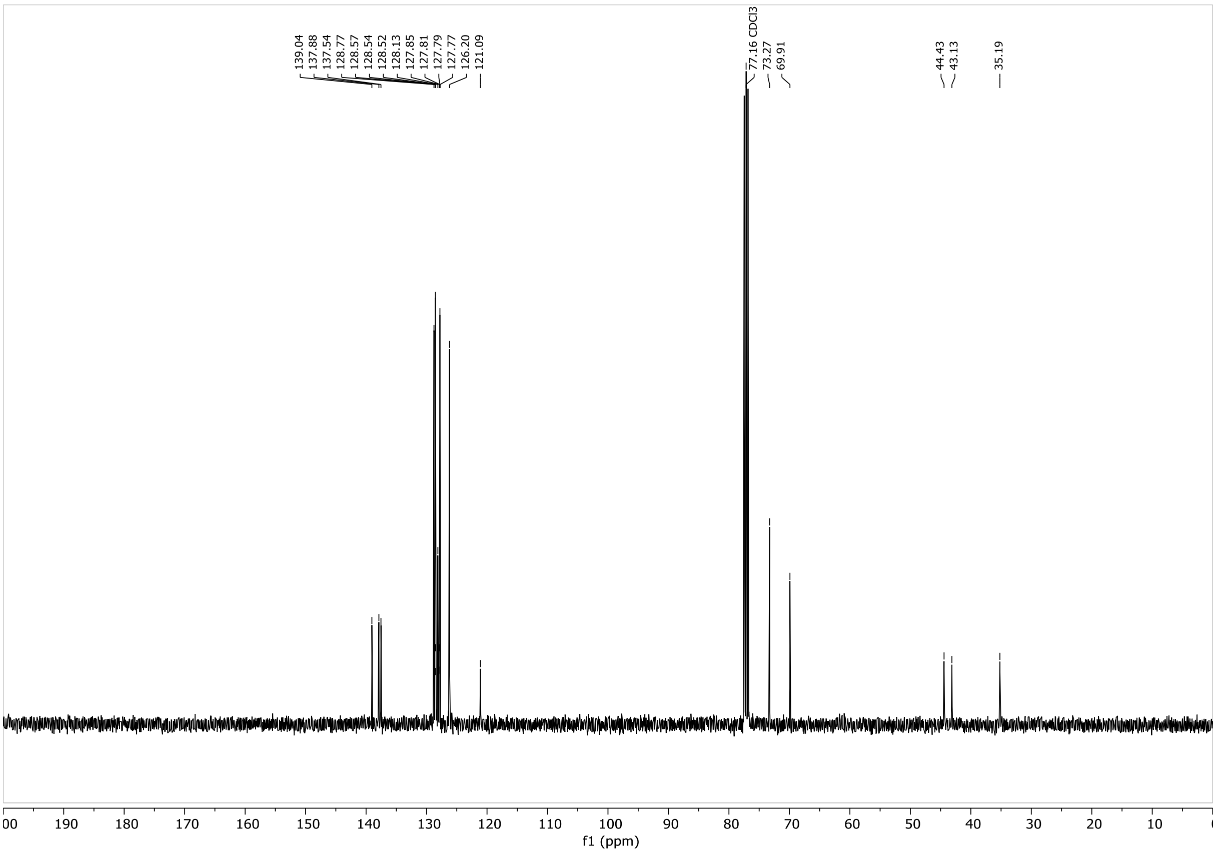
139.04
137.88
137.54
128.77
128.57
128.54
128.52
128.13
127.85
127.81
127.79
127.77
126.20
121.09
77.16 CDCl3
73.27
69.91
44.43
43.13
35.19
f1 (ppm)

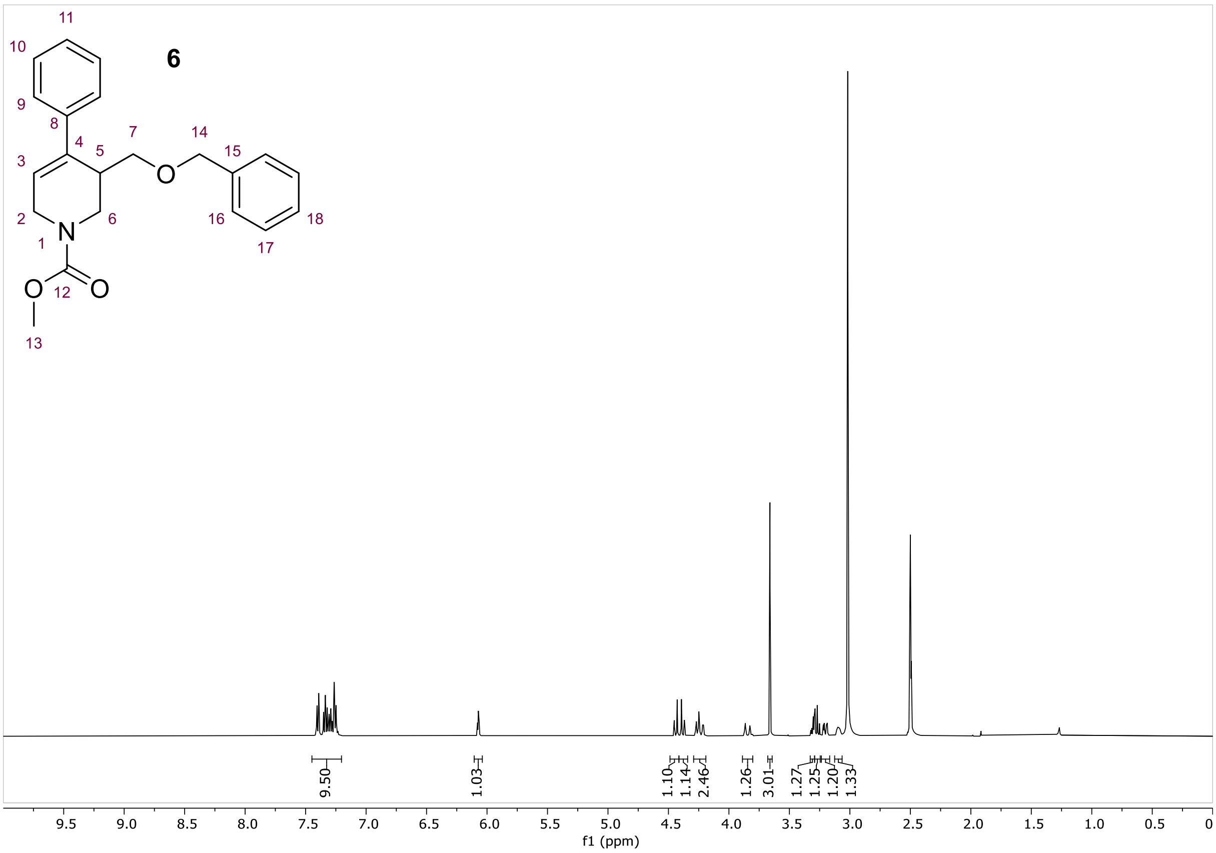
6
9.50
1.03
1.10
1.14
2.46
1.26
3.01
1.27
1.25
1.20
1.33
f1 (ppm)

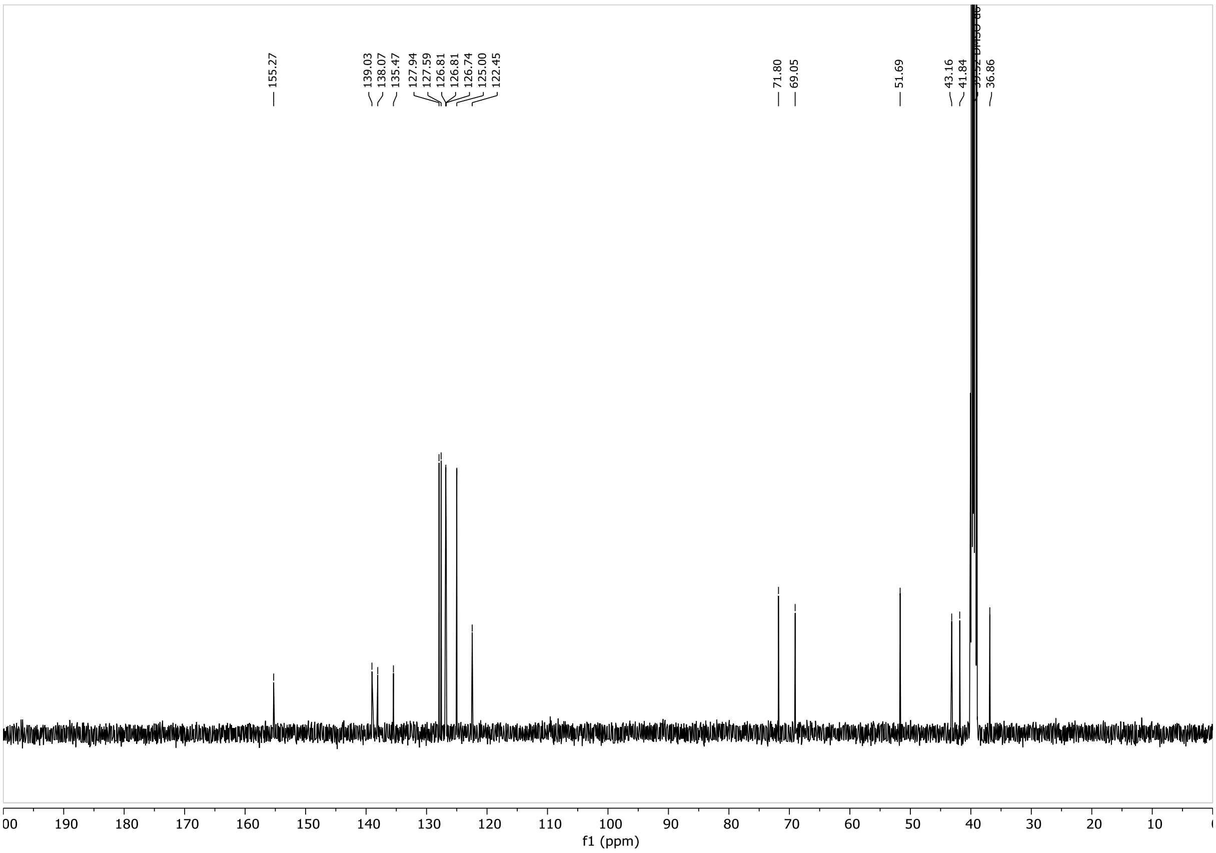
155.27
139.03
138.07
135.47
127.94
127.59
126.81
126.81
126.74
125.00
122.45
71.80
69.05
51.69
43.16
41.84
36.86
f1 (ppm)

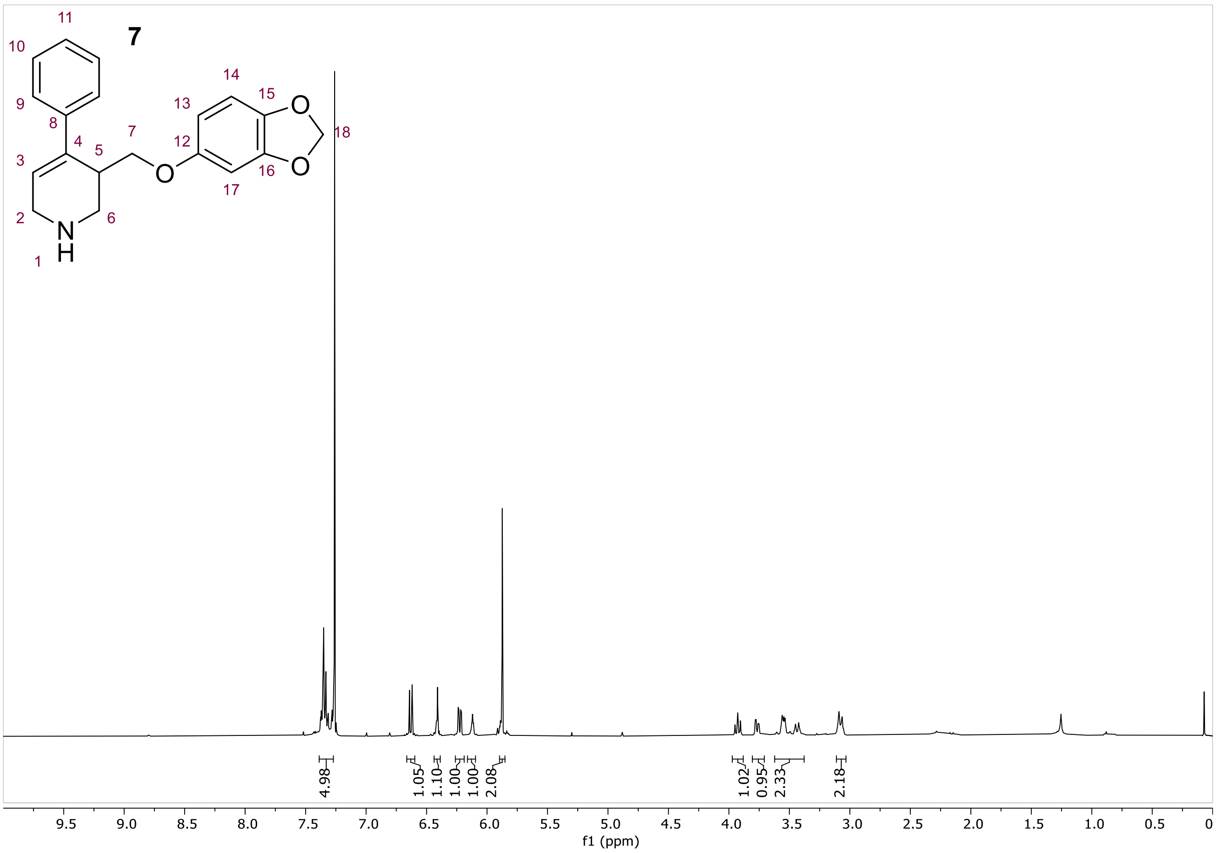
7
11
10
9
8
4
3
5
7
2
6
1
N
H
O
12
13
14
15
O
18
O
16
17
4.98
1.05
1.10
1.00
1.00
2.08
1.02
0.95
2.33
2.18
9.5
9.0
8.5
8.0
7.5
7.0
6.5
6.0
5.5
5.0
4.5
4.0
3.5
3.0
2.5
2.0
1.5
1.0
0.5
0
f1 (ppm)

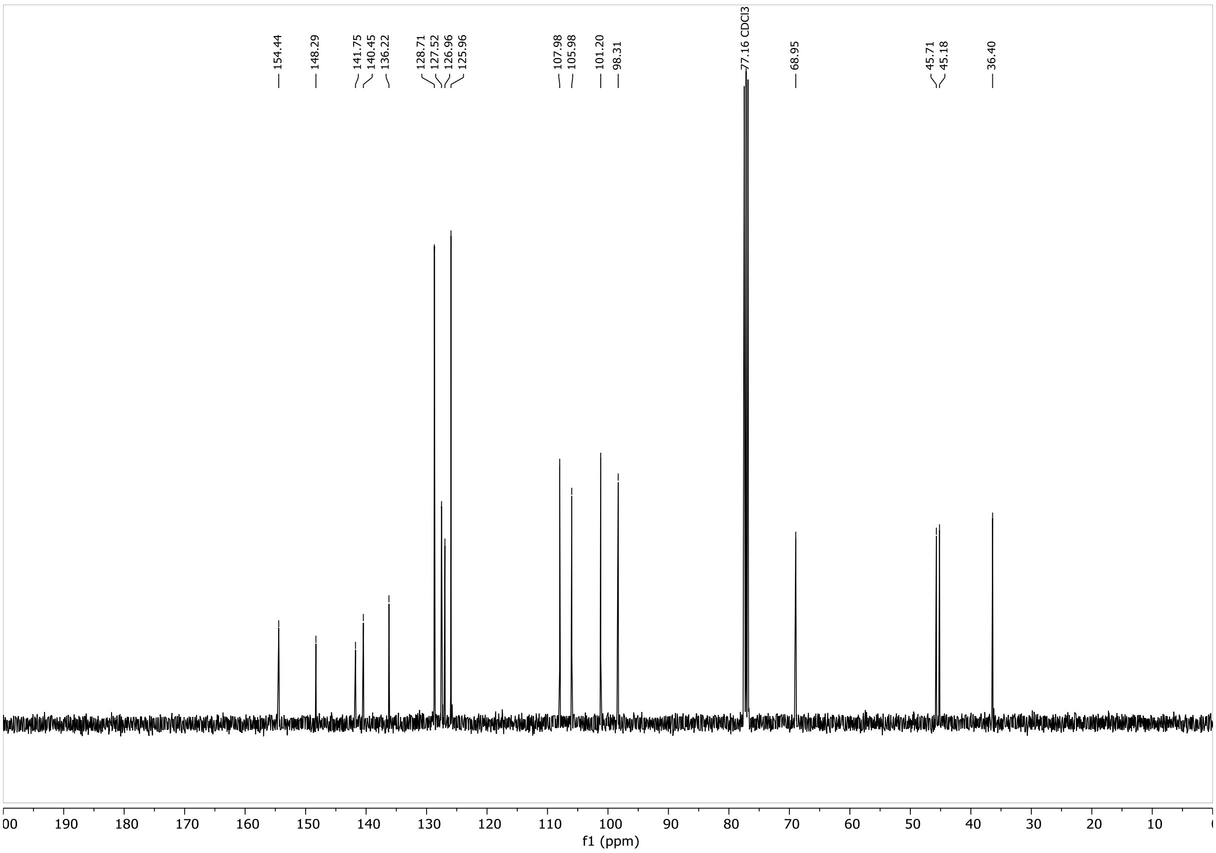
154.44
148.29
141.75
140.45
136.22
128.71
127.52
126.96
125.96
107.98
105.98
101.20
98.31
77.16 CDCl3
68.95
45.71
45.18
36.40
190
180
170
160
150
140
130
120
110
100
90
80
70
60
50
40
30
20
10
f1 (ppm)

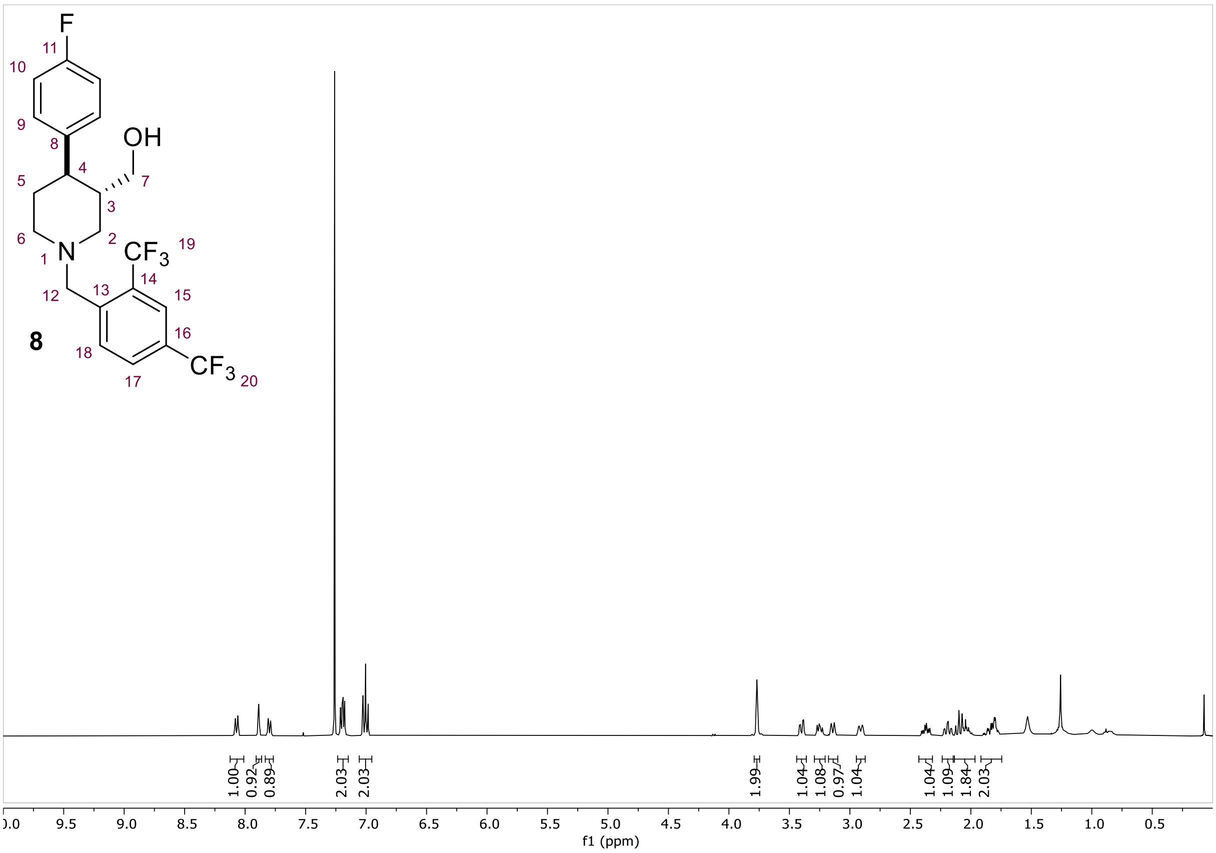

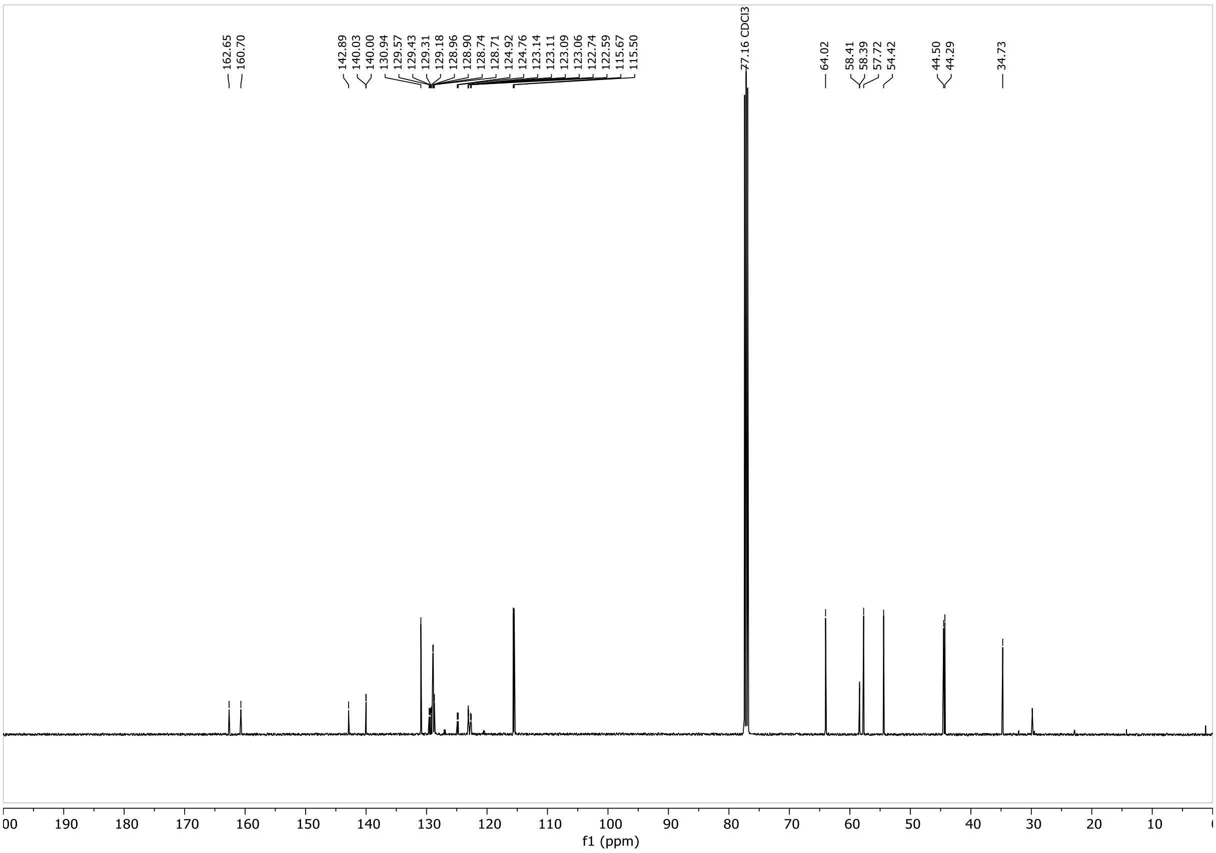

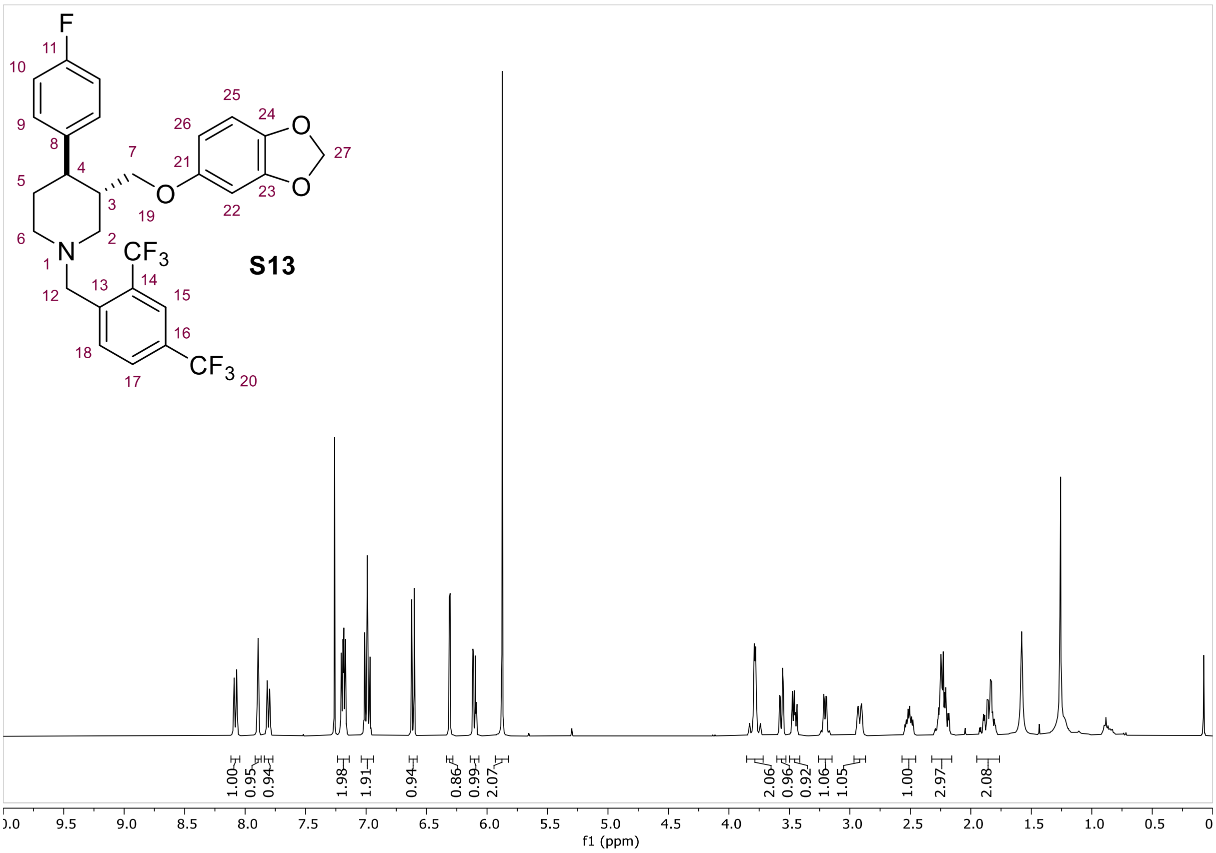
S13
F
CF3
CF3
O
O
O
N
1.00
0.95
0.94
1.98
1.91
0.94
0.86
0.99
2.07
2.06
0.96
0.92
1.06
1.05
1.00
2.97
2.08
f1 (ppm)

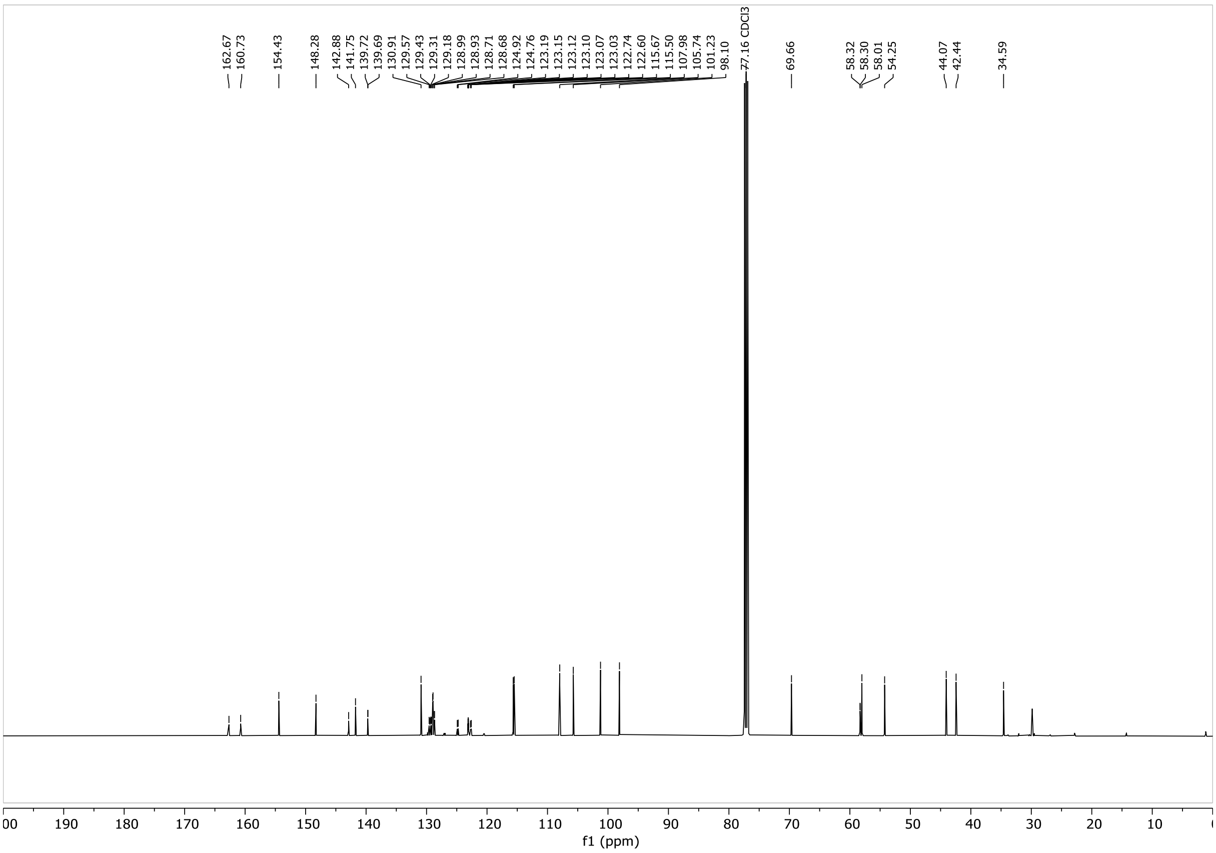
162.67
160.73
154.43
148.28
142.88
141.75
139.72
139.69
130.91
129.57
129.43
129.31
129.18
128.99
128.93
128.71
128.68
124.92
124.76
123.19
123.15
123.12
123.10
123.07
123.03
122.74
122.60
115.67
115.50
107.98
105.74
101.23
98.10
77.16 CDCl3
69.66
58.32
58.30
58.01
54.25
44.07
42.44
34.59
f1 (ppm)

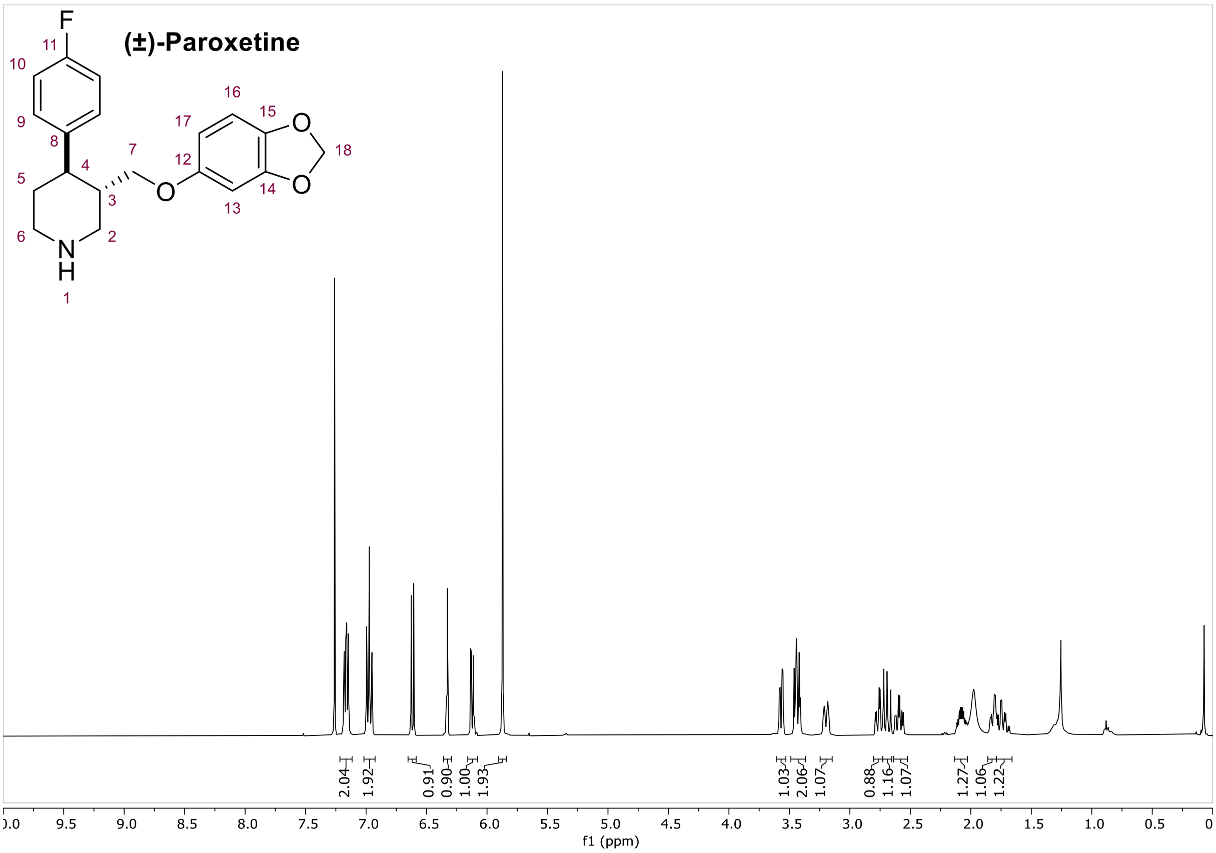
(±)-Paroxetine
F
O
O
O
N
H
1 2 3 4 5 6 7 8 9 10 11 12 13 14 15 16 17 18
2.04
1.92
0.91
0.90
1.00
1.93
1.03
2.06
1.07
0.88
1.16
1.07
1.27
1.06
1.22
9.5 9.0 8.5 8.0 7.5 7.0 6.5 6.0 5.5 5.0 4.5 4.0 3.5 3.0 2.5 2.0 1.5 1.0 0.5 0
f1 (ppm)

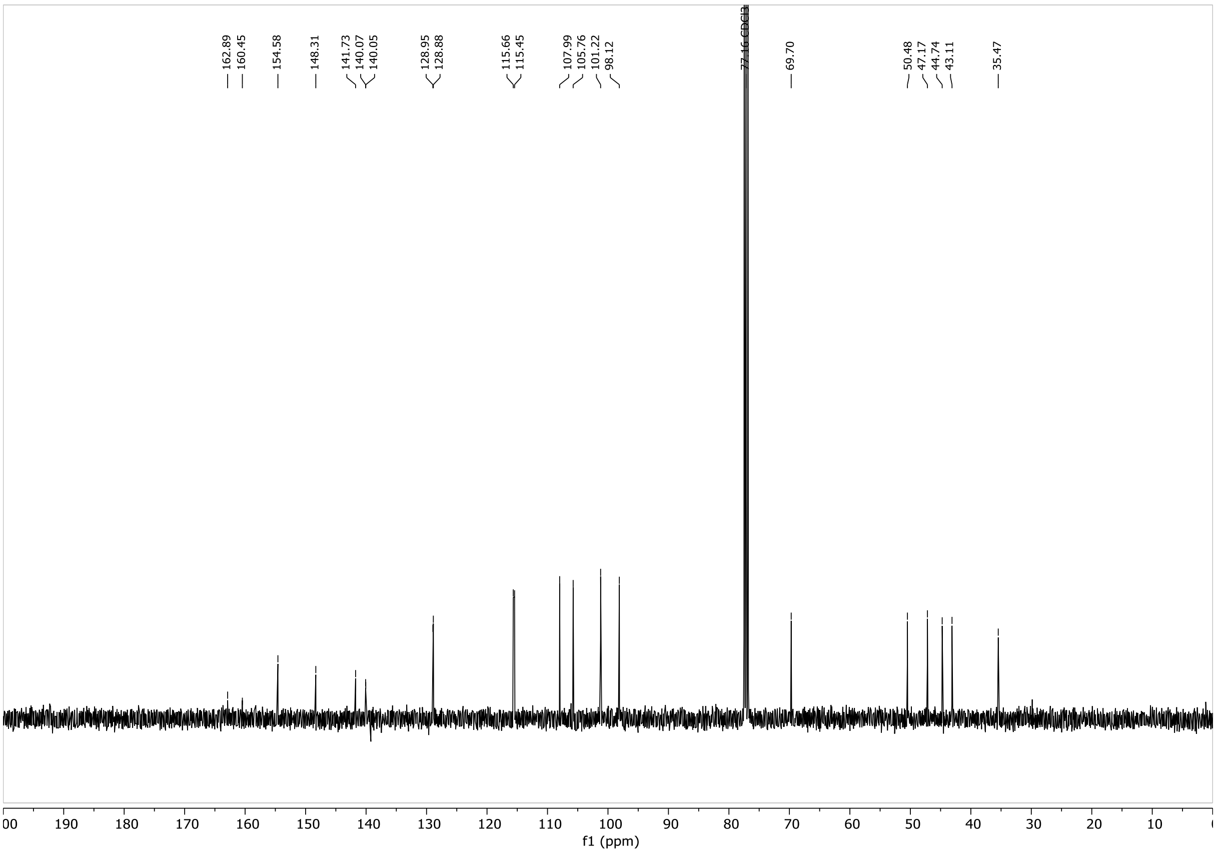
162.89
160.45
154.58
148.31
141.73
140.07
140.05
128.95
128.88
115.66
115.45
107.99
105.76
101.22
98.12
77.16 CDCl3
69.70
50.48
47.17
44.74
43.11
35.47
190 180 170 160 150 140 130 120 110 100 90 80 70 60 50 40 30 20 10
f1 (ppm)
